# Metabolic Interplay in Acute Lung Injury: PARK7 Integrates FADS1/2‐Dependent PUFA Metabolism and H3K14 Lactylation to Attenuate Endothelial Ferroptosis and Dysfunction

**DOI:** 10.1002/advs.202508725

**Published:** 2025-09-30

**Authors:** Jian Xu, Yuhan Wang, Weiqi Mao, Tianchang Wei, Yufan Li, Juan Song, Cuiping Zhang, Xiaoyan Chen, Cuicui Chen, Qingyuan Xu, Xu Wu, Yuanlin Song

**Affiliations:** ^1^ Shanghai Key Laboratory of Lung Inflammation and Injury Shanghai Respiratory Research Institute Department of Pulmonary Medicine Zhongshan Hospital Fudan University Shanghai 200032 China; ^2^ Shanghai Institute of Infectious Disease and Biosecurity Fudan University Shanghai Key Laboratory of Lung Inflammation and Injury Shanghai Respiratory Research Institute Department of Pulmonary Medicine Shanghai Institute of Infectious Disease and Biosecurity Fudan University Shanghai 200032 China; ^3^ National and Shanghai Clinical Research Center for Aging and Medicine Huashan Hospital Fudan University Shanghai 200040 China; ^4^ Key Laboratory of Chemical Injury Emergency and Critical Medicine of Shanghai Municipal Health Commission Center of Emergency and Critical Medicine Jinshan Hospital of Fudan University Shanghai 201508 China

**Keywords:** acute lung injury (ALI), endothelial cell, ferroptosis, histone lactylation, PARK7, polyunsaturated fatty acid (PUFA)

## Abstract

Acute respiratory distress syndrome (ARDS) is a severe clinical condition characterized by widespread inflammation and fluid accumulation in the lungs. Endothelial cell (EC) metabolic changes in acute lung injury (ALI) and their relationship to injury remain unclear. Transcriptomic and lipidomic analyses revealed downregulation of PUFA synthesis pathways, particularly omega‐3 PUFAs, in pulmonary ECs during LPS‐induced ALI. Activation of the PUFA metabolic pathway, through FADS1/2 overexpression or omega‐3 fatty acid supplementation, protected ECs from ferroptosis and restored barrier function. In vivo, pulmonary EC‐specific overexpression of FADS1/2 contributed to the alleviation of ALI. Overexpression of whole lung FADS1/2, combined with alpha‐linolenic acid (ALA) supplementation, also significantly mitigated ALI. PARK7 is identified as an endogenous regulator of FADS1/2, acting through the BMP‐BMPR‐SMAD1/5/9 signaling. Driven by histone H3K14 lactylation, which is also promoted by the downregulation of FADS1/2, PARK7 upregulation restored FADS1/2 expression and counteracted ferroptosis, thereby forming a protective feedback loop. This study elucidates a novel regulatory axis involving the two major metabolic changes—downregulation of PUFA synthesis and upregulation of histone lactylation—in ALI pathogenesis, which are interconnected through the PARK7‐BMP signaling pathway. Targeting this axis offers potential therapeutic strategies for mitigating endothelial dysfunction and ferroptosis in ARDS/ALI.

## Graphical abstract

SCAP/ARDS patients show reduced unsaturated fatty acids (UFAs) but higher PARK7 and lactate in plasma. PARK7 correlates positively with lactate and negatively with UFAs. Pulmonary endothelial cell multi‐omics show FADS1/2 downregulation and impaired PUFA metabolism. Lactate boosts PARK7 via H3K14 lactylation, and PARK7 then restores FADS1/2. Recovery of FADS1/2 and supplementation of omega‐3 fatty acids alleviate acute lung injury.

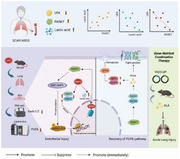

## 1 Introduction

Acute respiratory distress syndrome (ARDS) is a severe clinical condition characterized by widespread inflammation and fluid accumulation in the lungs, leading to severe hypoxemia and respiratory failure. ARDS poses a significant therapeutic challenge due to its high mortality and the lack of effective pharmacotherapy.^[^ ^1^, ^2^, ^3^ ^]^ Acute lung injury (ALI) is a milder form of ARDS, also involving lung inflammation and impaired gas exchange. Damage to the respiratory membrane is a critical pathophysiological process in the development of ARDS. Endothelial cells (ECs) serve as the primary barrier against the leakage of fluid, proteins, and inflammatory cells into the alveolar space.^[^ ^4^, ^5^ ^]^ Preventing endothelial injury and preserving the integrity of the respiratory membrane may thus represent a key therapeutic strategy to halt the progression of inflammatory cascades in ARDS. While the mechanisms of endothelial injury in ARDS/ALI have been extensively studied—particularly involving processes such as inflammation, glycocalyx degradation, oxidative stress, and pyroptosis—the precise patterns and underlying causes of post‐injury endothelial dysfunction remain poorly understood.^[^ ^6^, ^7^ ^]^

Lipid metabolism plays a critical role in maintaining cellular homeostasis and functional integrity. Various lipid metabolic processes in pulmonary cells contribute to essential functions including membrane stabilization and surfactant production.^[^ ^8^ ^]^ Under ARDS/ALI conditions, significant alterations occur in pulmonary lipid profiles, including changes in fatty acids, phospholipids, and sphingolipids, with a notable reduction in omega‐3 fatty acid levels.^[^ ^9^, ^10^, ^11^ ^]^ Unsaturated fatty acids (UFAs) are a class of fatty acids containing carbon–carbon double bonds in their molecular chains. These double bonds create “kinks” in the molecular structure, which reduce intermolecular packing density and enhance the fluidity of biological membranes. Based on their degree of unsaturation, UFAs can be classified into two main categories: monounsaturated fatty acids (MUFAs) and polyunsaturated fatty acids (PUFAs). Omega‐3 PUFAs, including α‐linolenic acid (ALA), eicosapentaenoic acid (EPA), docosahexaenoic acid (DHA), etc., play critical roles in various physiological and pathological processes and are implicated in multiple diseases such as metabolic disorders, cardiovascular diseases, and inflammatory conditions.^[^ ^12^ ^]^ In the PUFA synthesis pathway, ELOVL6, the enzyme responsible for PUFA elongation, was downregulated in bleomycin‐induced pulmonary injury, and its deficiency promoted oxidative damage.^[^ ^13^ ^]^ FADS1 and FADS2 (fatty acid desaturase 1 and 2) are rate‐limiting enzymes that catalyze the biosynthesis of PUFAs by introducing double bonds into fatty acid chains. Their roles in cellular damage responses have attracted increasing research attention in recent years. In tumor cells, FADS2 and SCD1 (a MUFA desaturase) are aberrantly upregulated and confer resistance to ferroptosis.^[^ ^14^, ^15^ ^]^ Moreover, additional overexpression of FADS2 contributes to suppressing inflammatory responses in Crohn's disease.^[^ ^16^ ^]^ Therefore, PUFA desaturase dysfunction may be associated with cellular damage and inflammatory responses, providing important insights into the mechanisms of cell injury in ALI.

Ferroptosis is an iron‐dependent form of regulated cell death driven by lipid peroxidation and glutathione depletion.^[^ ^17^ ^]^ In recent years, the role of ferroptosis in ALI has garnered increasing attention. Lipid peroxidation, iron deposition, and related processes trigger inflammatory responses, mitochondrial dysfunction, and oxidative stress, thereby exacerbating pulmonary damage.^[^ ^18^ ^]^ Based on the molecular mechanisms of ferroptosis, an increasing number of biomarkers and therapeutic targets (e.g., NRF2) have been identified, offering potential strategies to mitigate lung injury through ferroptosis intervention.^[^ ^19^ ^]^ The PUFA metabolic pathway is directly linked to ferroptosis. Notably, PUFAs with higher degrees of unsaturation undergo faster oxidation rates. Moreover, the oxidation of PUFA‐containing phospholipids directly drives the initiation and progression of ferroptosis.^[^ ^20^ ^]^ Omega‐6 PUFA arachidonic acid (AA) metabolism also promotes ferroptosis.^[^ ^20^ ^]^ As key enzymes in PUFA biosynthesis, FADS1 and FADS2 have also been implicated in promoting ferroptosis according to emerging studies.^[^ ^21^, ^22^, ^23^ ^]^ This appears to present a paradox: PUFAs serve dual roles as both crucial components for antioxidation and cellular homeostasis maintenance, while simultaneously acting as a reservoir for ferroptosis execution. This suggests that desaturases and PUFAs may exert divergent effects depending on cellular contexts and disease models. The specific roles of FADS1/2 and PUFAs in lung injury require urgent experimental investigation. NRF2 is a critical regulator of ferroptosis. PARK7 (DJ‐1) represents another critical antioxidant molecule that functions as a reactive oxygen species (ROS) scavenger and exhibits close interplay with NRF2 signaling.^[^ ^24^, ^25^ ^]^ Previous studies have identified PARK7 as both a potential biomarker and protective molecule in ARDS/ALI.^[^ ^26^, ^27^ ^]^ Our preliminary studies have demonstrated that endothelial PARK7 serves as a critical mediator against oxidative damage and pyroptosis.^[^ ^28^ ^]^ In other disease models, such as cancer, PARK7 has been shown to alleviate cellular ferroptosis.^[^ ^29^, ^30^, ^31^ ^]^ However, its relationship with PUFA metabolism and ferroptosis in ALI remains to be further investigated and elucidated.

Lactylation is a novel post‐translational modification where lactate‐derived acyl groups are covalently attached to lysine residues, linking cellular metabolism to epigenetic regulation, participating in different disease conditions.^[^ ^32^, ^33^ ^]^ In critically ill patients, elevated blood lactate levels resulting from enhanced anaerobic respiration undergo functional repurposing through lactylation, transforming metabolic waste into regulators of biological functions. Histone H3 and H4 lactylation represent prevalent forms of lactylation modifications. In pulmonary diseases, histone lactylation modulates critical cellular processes—including cell cycle progression, cell death, and cellular metabolism—through transcriptional regulation.^[^ ^34^ ^]^ Recent studies have revealed that endothelial H3K14 lactylation (H3K14la) promotes ferroptosis, thereby exacerbating endothelial dysfunction in ARDS and mechanistically linking ARDS/ALI pathogenesis to ferroptotic cell death.^[^ ^35^ ^]^ Moreover, in ARDS, endothelial H3K18la accelerates glycocalyx degradation.^[^ ^36^ ^]^ Consequently, endothelial histone lactylation emerges as a promising research target in ARDS/ALI, with its interplay with PUFA metabolism awaiting further exploration.

## 2 Results

### 2.1 Omega‐3 PUFA Biosynthesis is Impaired in Pulmonary ECs During Lipopolysaccharides (LPS)‐Induced ALI

**Figure 1:**
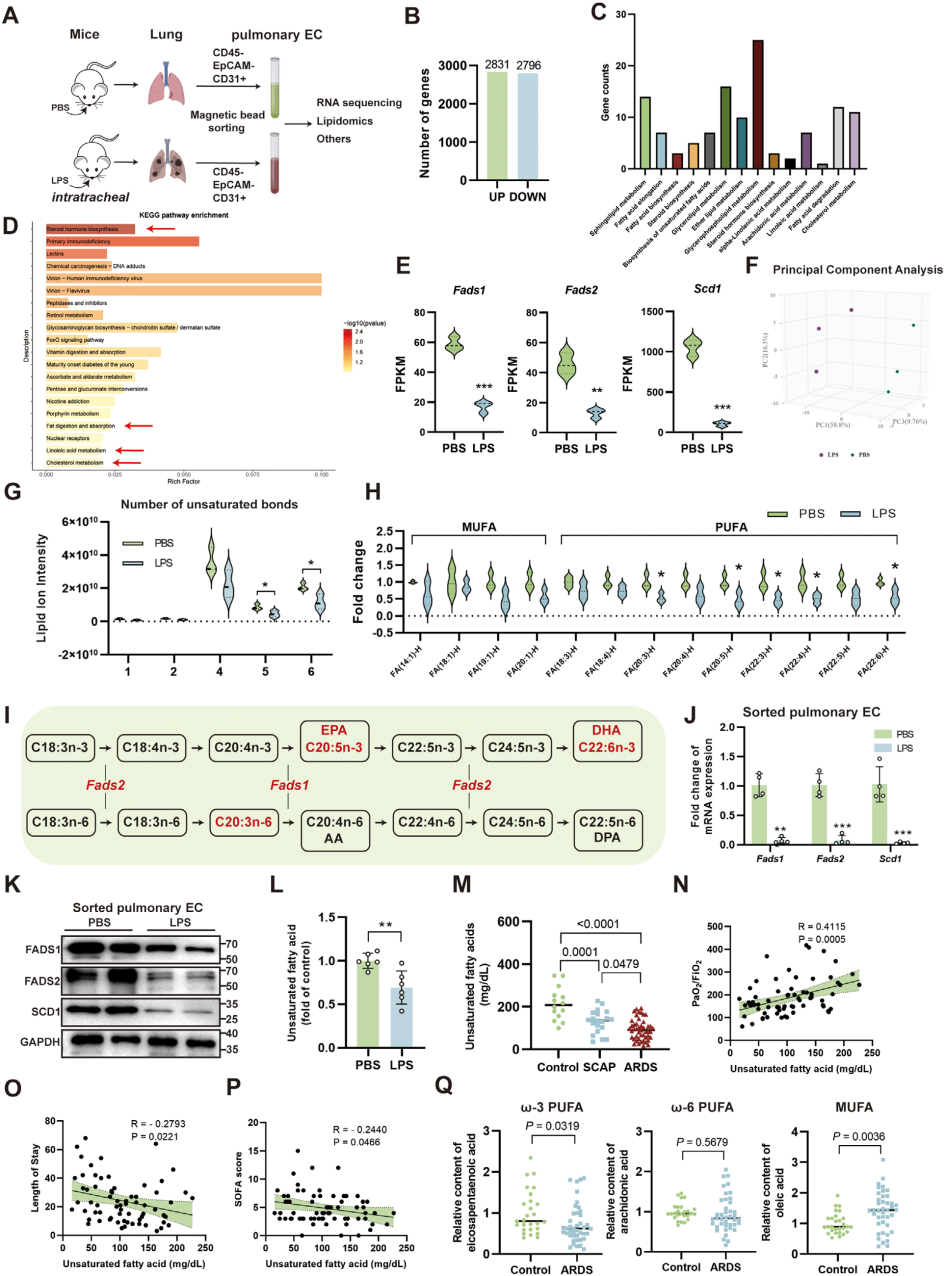
Pulmonary endothelial cells (ECs) exhibit impaired polyunsaturated fatty acid (PUFA) biosynthesis pathways during lipopolysaccharide (LPS)‐induced acute lung injury (ALI). A) Schematic diagram of pulmonary EC sorting and investigation. B) Number of differentially expressed genes (DEGs) from transcriptome sequencing. C) Number of lipid metabolism‐associated DEGs. D) KEGG pathway enrichment analysis of the top 200 DEGs. Red arrows indicate pathways associated with lipid metabolism. E) Changes in fatty acid desaturase expression levels revealed by transcriptome sequencing (n = 3). F) Principal component analysis (PCA) of lipidomics data in pulmonary ECs. G) Changes in the number of unsaturated bonds in fatty acids of pulmonary ECs (n = 3). H) Changes in the content of various unsaturated fatty acid (UFA) species in pulmonary ECs (n = 3). I) Schematic diagram of downregulated desaturase genes and fatty acid species in the PUFA metabolic pathway of pulmonary ECs after LPS‐ALI. Red indicates downregulated genes or lipid species. J) Changes in desaturase mRNA expression levels in sorted pulmonary ECs (n = 3). K) Changes in desaturase expression levels detected by Western blot in sorted pulmonary ECs. L) Changes in relative content of total UFAs in lung tissue after LPS‐induced ALI (n = 6). M) UFA levels in plasma of control group (n = 14), SCAP (n = 20), and ARDS (n = 47) patients. N–P) Correlation analysis between plasma UFA levels and clinical parameters for SCAP and ARDS patients: N) PaO_2_/FiO_2_ ratio, O) length of stay, and P) SOFA score. Q) Plasma eicosapentaenoic acid, arachidonic acid, and oleic acid levels in healthy controls versus ARDS patients from the external validation dataset. This subfigure was recreated and analyzed using publicly available data from doi: 10.1186/s12931‐020‐01364‐6. The use of the original data complies with the Creative Commons Attribution 4.0 International License (CC BY 4.0) of the source article. ARDS, acute respiratory distress syndrome; SCAP, severe community‐acquired pneumonia. *, *P* < 0.05; **, *P* < 0.01; ***, *P* < 0.001.

To investigate the alterations in lipid metabolism in pulmonary ECs during LPS‐induced ALI, magnetic bead sorting was employed to isolate endothelial cells (EpCAM^−^, CD45^−^, CD31⁺) (**Figure** **1**A). The sorted cells were subsequently used for omics analyses or validation experiments. RNA sequencing (RNA‐seq) identified a total of 5627 differentially expressed genes (DEGs) (Figure 1B), with 117 genes associated with lipid metabolism distributed across different lipid metabolic pathways (Figure 1C; Figure S1A, Supporting Information). KEGG enrichment analysis of the top 200 DEGs highlighted significant alterations of lipid‐related pathways (red arrows, Figure 1D). Notably, key desaturases (*Fads1/2* and *Scd1*) were downregulated at the mRNA level (Figure 1E). To further validate these metabolic differences, parallel lipidomics were conducted separately. Lipidomics analysis revealed significant differences in total lipid content, lipid composition ratios, and individual lipid species in pulmonary ECs following LPS‐induced ALI (Figure S1B—F, Supporting Information). Principal component analysis (PCA) demonstrated significant alterations in the lipid profile following LPS‐induced ALI (Figure 1F). Notably, total fatty acid content showed a non‐significant decreasing trend after LPS‐ALI (Figure S1G, Supporting Information), while fatty acid unsaturation was significantly reduced, particularly in highly unsaturated fatty acids (Figure 1G). At the individual lipid species level, PUFAs, but not MUFAs, exhibited significant reductions, with notable decreases in the omega‐3 fatty acids EPA^[^(FA(20:5)] and DHA^[^(FA(22:6)] (Figure 1H). In summary, the UFA biosynthesis pathway was downregulated in pulmonary ECs following LPS‐induced ALI, with particularly pronounced reductions in omega‐3 PUFAs (Figure 1I). Real‐time quantitative PCR (qPCR) and western blot (WB) analyses validated the downregulation of desaturases FADS1/2 and SCD1 at both mRNA and protein levels in sorted pulmonary ECs (Figure 1J,K; Figure S1H, Supporting Information). In vitro endothelial injury models and in vivo immunohistochemistry (IHC) analyses of lung tissues consistently confirmed this downregulation trend (Figure S1I—M, Supporting Information). The relative content of total UFAs was reduced in lung tissue following injury (Figure 1L). To further validate this finding, we collected plasma samples from 14 control subjects (patients with other non‐critical respiratory diseases such as asthma and interstitial lung disease), 20 non‐ARDS severe community‐acquired pneumonia (SCAP) patients, and 47 ARDS patients caused by SCAP. Table S1 (Supporting Information) presents the baseline characteristics of SCAP and ARDS patients. SCAP patients exhibited reduced plasma UFA levels, while ARDS patients showed even lower UFA concentrations (Figure 1M). Moreover, plasma UFA levels in SCAP and ARDS patients showed a positive correlation with the PaO_2_/FiO_2_ ratio, while demonstrating a negative correlation with both length of hospital stay and SOFA score (Figure 1N–P). Statistical analysis of previously published metabolomics data^[^ ^37^ ^]^ from healthy controls and ARDS patients revealed a significant decrease in EPA and an increase in oleic acid in theplasma of ARDS patients, while arachidonic acid remained unchanged (Figure 1Q). These findings suggest that downregulation of omega‐3 PUFAs is a characteristic feature of ARDS/ALI.

### 2.2 PUFA Metabolic Pathway Activation Protects Against Endothelial Ferroptosis

**Figure 2:**
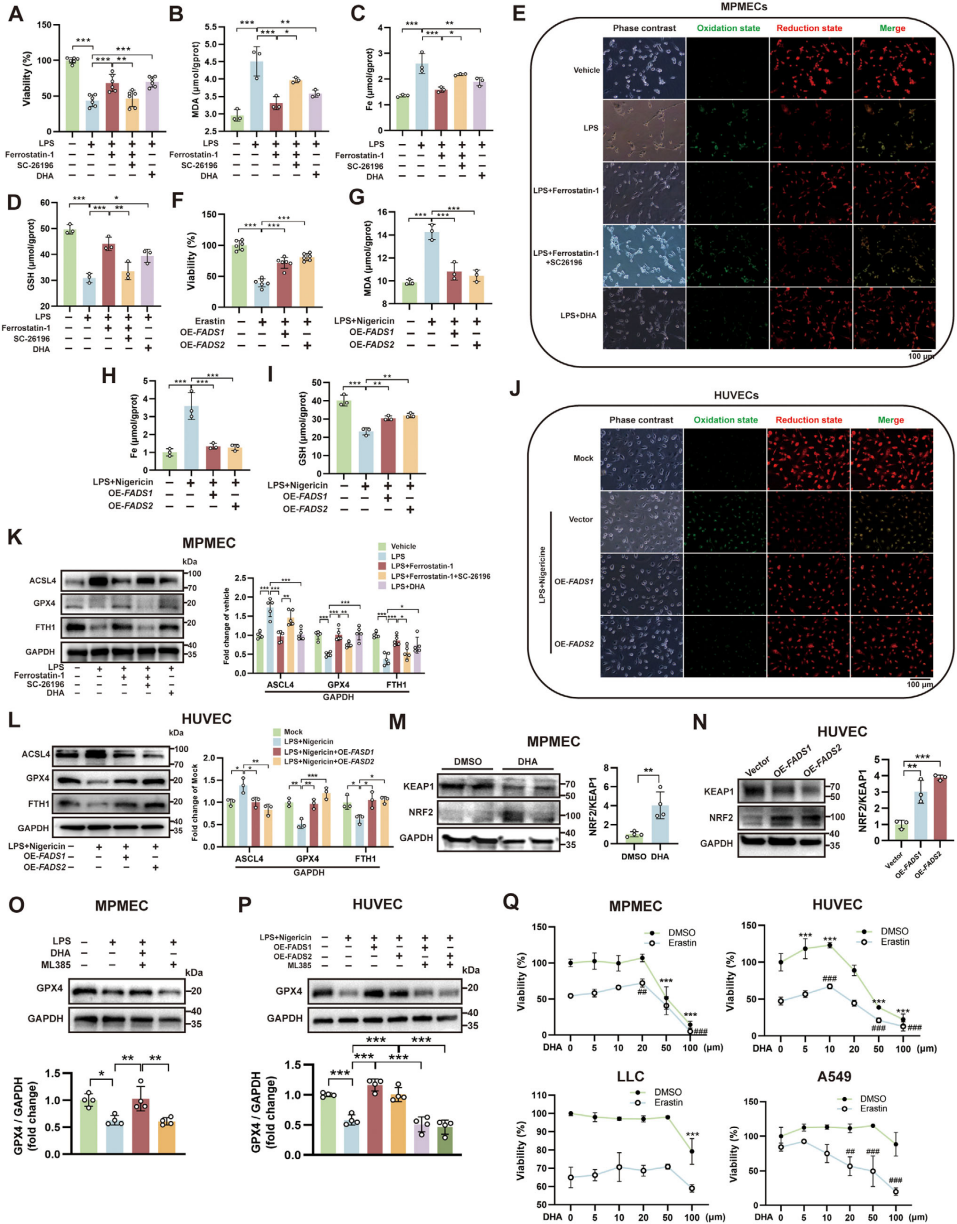
Activation of the polyunsaturated fatty acid (PUFA) metabolic pathway alleviates endothelial cell (EC) ferroptosis. A–D) Changes in cell viability (A, n = 6), malondialdehyde (MDA) levels (B, n = 3), total iron content (C, n = 3), and reduced glutathione (GSH) levels (D, n = 3) in mouse pulmonary microvascular endothelial cells (MPMECs) treated with lipopolysaccharide (LPS), Ferrostatin‐1, SC‐26196 or docosahexaenoic acid (DHA). E) Detection of lipid peroxidation (LPO) in MPMECs treated with LPS, Ferrostatin‐1, SC‐26196 or DHA. F–I) Changes in cell viability (F, n = 6), MDA levels (G, n = 3), total iron content (H, n = 3), and reduced GSH levels (I, n = 3) in human umbilical vein endothelial cells (HUVECs) following treatment with erastin, LPS, nigericin, and transfection with *FADS1/2* overexpression plasmids. J) Detection of LPO in HUVECs following treatment with erastin, LPS, nigericin, and transfection with *FADS1/2* overexpression plasmids. K) Western blot (WB) analysis of ferroptosis‐related gene expression changes and quantification in MPMECs (n = 5). L) WB analysis of ferroptosis‐related gene expression changes and quantification in HUVECs (n = 3). M) WB analysis of KEAP1/NRF2 expression changes in MPMECs after DHA treatment (n = 4). N) WB analysis of KEAP1/NRF2 expression changes in HUVECs after overexpression of *FADS1* or *FADS2* (n = 3). O) WB analysis of GPX4 expression in MPMECs after LPS injury, DHA treatment, and ML385 administration (n = 4). P) WB analysis of GPX4 expression in HUVECs after injury, DHA treatment, and ML385 administration (n = 4). Q) Changes in cell viability measured by CCK‐8 assay after erastin injury and treatment with different concentrations of DHA in various cell lines (n = 6). *, compared with the DMSO + 0 µM DHA group; #, compared with the Erastin + 0 µM DHA group. OE, overexpression; Vehicle, addition of corresponding solvents to the culture system; Vector, transfection with vector plasmids; Mock, transfection with vector plasmids and addition of corresponding solvents; LLC, Lewis lung carcinoma. *, #, *P* < 0.05; **, ##, *P* < 0.01; ***, ###, *P* < 0.001.

Lipid metabolism is closely associated with ferroptosis. However, the specific roles of the PUFA pathway and FADS1/2 in EC ferroptosis remain unclear. To validate the impact of the PUFA metabolic pathway on endothelial cell ferroptosis, mouse pulmonary microvascular endothelial cells (MPMECs) and human umbilical vein endothelial cells (HUVECs) were utilized to establish in vitro models. In MPMECs, LPS treatment significantly decreased cell viability, increased malondialdehyde (MDA) levels and total iron content, decreased reduced glutathione (GSH) levels, and promoted lipid peroxidation, indicative of ferroptosis activation (**Figure** **2**A–E). These effects were reversed by the ferroptosis inhibitor Ferrostatin‐1. However, co‐treatment with the FADS2 inhibitor SC‐26196 abolished this protective effect (Figure 2A–E). Notably, DHA pretreatment effectively attenuated LPS‐induced ferroptosis (Figure 2A–E). In the HUVEC model, the ferroptosis inducer erastin significantly reduced cell viability, while overexpression of *FADS1/2* effectively counteracted this cytotoxic effect, demonstrating that FADS1/2 directly protects endothelial cells against ferroptosis (Figure 2F). Validation of *FADS1/2* overexpression is shown in Figure S2A—D (Supporting Information). Subsequently, combined treatment with LPS and nigericin was used to simulate HUVEC injury, resulting in increased MDA levels and total iron content, decreased reduced GSH, and enhanced lipid peroxidation. Overexpression of either *FADS1* or *FADS2* attenuated the activation of ferroptosis under these conditions (Figure 2G–J). Furthermore, WB analysis revealed differential expression of key ferroptosis regulators during LPS‐induced injury: ACSL4 was upregulated while FTH1 and GPX4 were downregulated (Figure 2KL). In MPMECs, both Ferrostatin‐1 and DHA treatment suppressed these molecular alterations, whereas the FADS2 inhibitor SC‐26196 abolished the therapeutic effects of Ferrostatin‐1 (Figure 2K). Notably, *FADS1/2* overexpression in HUVECs reversed LPS/nigericin‐induced activation of the ferroptosis pathway (Figure 2L). Further WB analysis demonstrated that both DHA pretreatment and *FADS1/2* overexpression increased the NRF2/KEAP1 ratio, which was associated with ferroptosis protection (Figure 2M,N). Administration of the NRF2 inhibitor ML385 during DHA treatment or *FADS1/2* overexpression in injured MPMECs/HUVECs reversed the therapy‐induced recovery of cell viability (Figure S3A,B) and of GPX4 expression (Figure 2O,P). To investigate the dual effects of PUFAs on ferroptosis, we treated different cell lines with erastin to induce injury and administered varying doses of DHA (Figure 2Q). CCK‐8 assays revealed that in two endothelial cell lines, low concentrations of DHA (<20 or 10 µM) partially rescued ferroptosis, while high concentrations of DHA itself induced cell death. In contrast, two lung tumor cell lines (LLC and A549) showed stronger resistance to high‐dose DHA but exhibited minimal therapeutic response to DHA in ferroptosis mitigation. After treatment with different concentrations of DHA, GPX4 expression in MPMECs showed a trend of upregulation at low concentrations and downregulation at high concentrations (Figure S3C,D, Supporting Information). These results suggest that the PUFA pathway can protect endothelial cells from LPS‐induced ferroptosis through modulation of NRF2‐GPX4 signaling.

### 2.3 PUFA Metabolic Pathway Activation Improves Endothelial Function After Injury

**Figure 3:**
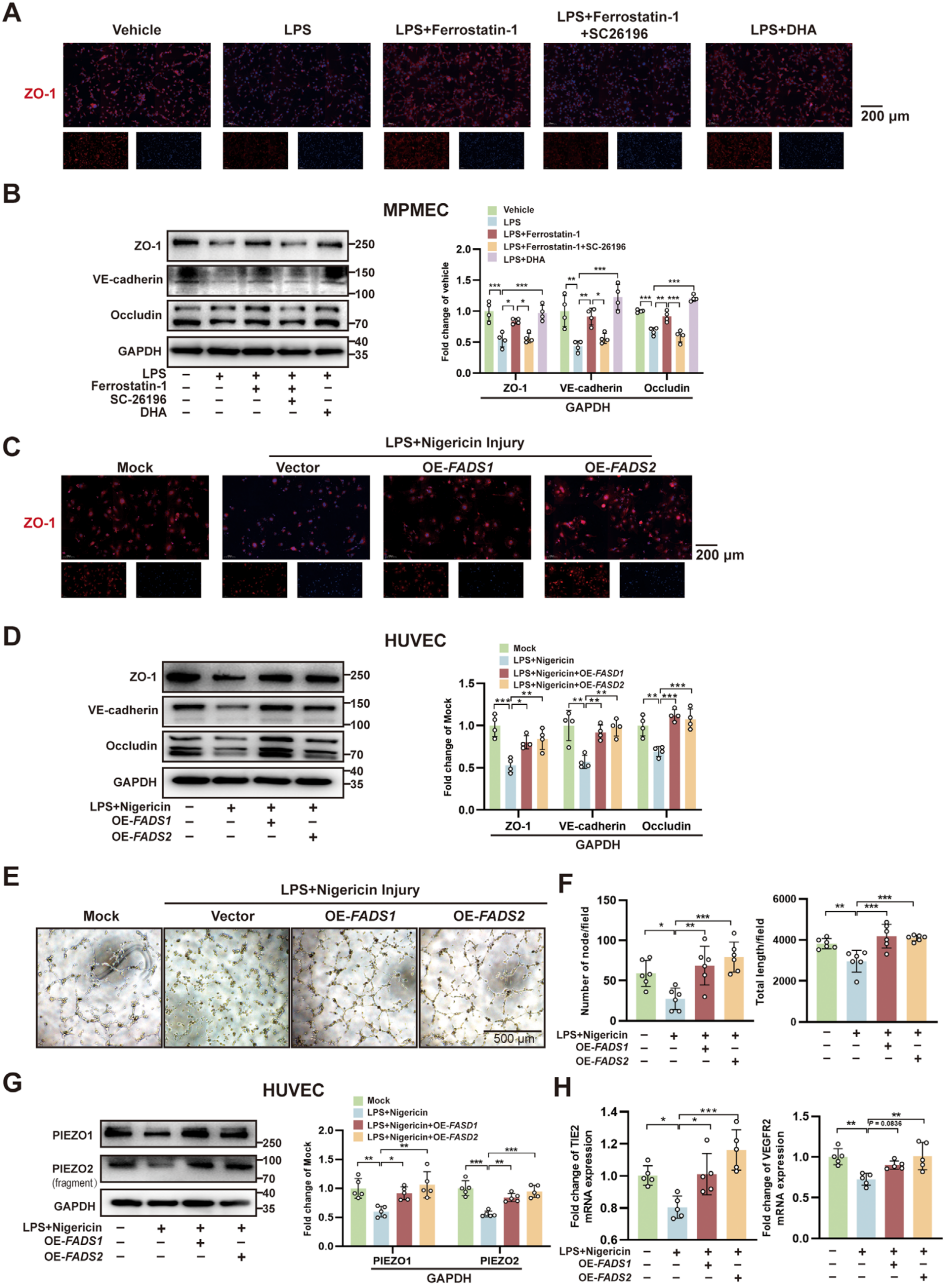
Activation of the polyunsaturated fatty acid (PUFA) metabolic pathway ameliorates endothelial dysfunction post‐injury. A) Immunofluorescence (IF) analysis of ZO‐1 expression changes in mouse pulmonary microvascular endothelial cells (MPMECs) treated with lipopolysaccharide (LPS), Ferrostatin‐1, SC‐26196, or docosahexaenoic acid (DHA). B) Western blot (WB) analysis of tight junction proteins and quantification of MPMECs treated with LPS, Ferrostatin‐1, SC‐26196, or DHA (n = 4). C) IF analysis of ZO‐1 expression changes in human umbilical vein endothelial cells (HUVECs) following treatment with LPS, nigericin, and transfection with *FADS1/2* overexpression plasmids. D) WB analysis of tight junction proteins and quantification of HUVECs following treatment with LPS, nigericin, and transfection with *FADS1/2* overexpression plasmids (n = 4). E) Tube formation assay images of HUVECs. F) Quantitative analysis of node number and tube length in HUVEC tube formation assay (n = 6). G) WB analysis and quantification of Piezo1 and Piezo2 protein expression in HUVECs (n = 5). H) Quantitative real‐time PCR analysis of endothelial markers TIE2 and VEGFR2 expression levels (n = 5). OE, overexpression; Vehicle, addition of corresponding solvents to the culture system; Vector, transfection with vector plasmids; Mock, transfection with vector plasmids and addition of corresponding solvents. *, *P* < 0.05; **, *P* < 0.01; ***, *P* < 0.001.

The potential of the PUFA pathway to enhance post‐injury endothelial cell function was further validated in vitro. In MPMECs, immunofluorescence (IF) and WB analyses demonstrated that ferrostatin‐1 and DHA treatment preserved ZO‐1 expression and other tight junction proteins, while FADS2 inhibitor SC‐26196 exacerbated LPS‐induced junctional disruption (**Figures** **3**A,B). Similar results were observed in HUVECs, where *FADS1/2* overexpression reversed the downregulation of ZO‐1 and other junctional proteins after LPS/nigericin challenge (Figure 3C,D). Transmission electron microscopy (TEM) further corroborated these findings, demonstrating that ferrostatin‐1, DHA, and *FADS1/2* overexpression preserved intact tight junction ultrastructure in both MPMECs (Figure S3E, Supporting Information) and HUVECs (Figure S3F, Supporting Information) under inflammatory conditions, whereas the FADS2 inhibitor SC‐26196 exacerbated junctional disruption in MPMECs. Functional assays revealed that PUFA pathway activation enhanced angiogenic capacity, as evidenced by increased node formation and tube length in HUVEC tube formation assays (Figures 3E,F). Mechanistically, both Piezo1/2 mechanosensitive channels (Figure 3G) and endothelial markers (TIE2, VEGFR2; Figure 3H) were upregulated. These data collectively indicate that activation of the PUFA metabolic pathway significantly improved endothelial barrier integrity and function following injury.

### 2.4 EC‐Specific Overexpression of Fads1/2 Alleviates LPS‐Induced ALI

**Figure 4:**
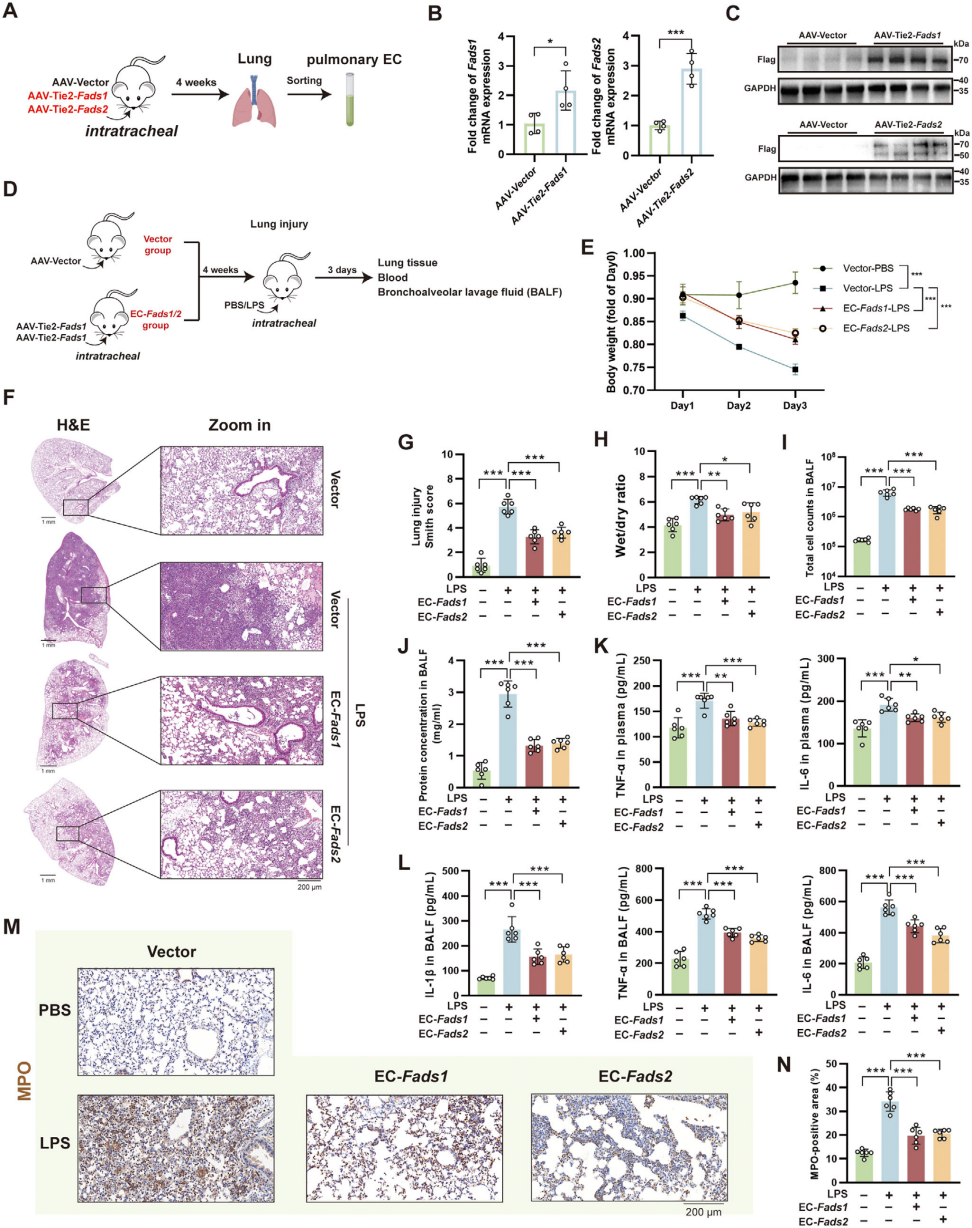
Lung endothelial cell (EC) ‐specific overexpression of *Fads1/2* via AAV infection alleviates lipopolysaccharide (LPS)‐induced acute lung injury (ALI) in mice. A) Schematic diagram of AAV infection and validation of sorted endothelial cells. B) Quantitative real‐time PCR (qPCR) analysis of sorted endothelial cells confirming *Fads1/2* overexpression at the mRNA level (n = 4). C) Western blot detection of Flag‐tagged proteins in sorted endothelial cells, validating FADS1/2 protein overexpression (n = 4). D) Schematic diagram of the experimental model for validating the impact of EC‐specific Fads1/2 overexpression on ALI; E) Relative changes in body weight of mice over time following LPS‐induced ALI (n = 6). F) Hematoxylin and eosin (H&E) staining of harvested lung tissue. G) Lung injury scoring based on H&E staining (n = 6). H) Wet‐to‐dry weight ratio of lung tissue (n = 6). I–J) Total cell counts (I) and protein concentrations (J) in bronchoalveolar lavage fluid (BALF) (n = 6). K) Concentrations of TNF‐α and IL‐6 in plasma. L) Concentrations of IL‐1β, TNF‐α, and IL‐6 in BALF (n = 6). M–N) Immunohistochemical staining images of MPO in lung tissues (M) and quantitative analysis of positive area (N) (n = 6). AAV, adeno‐associated virus. *, *P* < 0.05; **, *P* < 0.01; ***, *P* < 0.001.

To confirm the protective role of pulmonary endothelial cells in ALI in vivo, we intratracheally administered adeno‐associated virus (AAV, serotype Vec) carrying a Tie2‐promoted *Fads1* or *Fads2* overexpression vector (**Figure** **4**A). The vectors contained a Flag tag and ZsGreen to validate overexpression efficiency (Figure S4A,B, Supporting Information). Four weeks after AAV infection, pulmonary endothelial cells were sorted and subjected to qPCR analysis, which demonstrated upregulation of *Fads1/2* at the mRNA level (Figure 4B). WB analysis further confirmed strong positivity for the Flag tag (Figure 4C). To verify expression specificity, WB analysis confirmed that the sorted and retained endothelial cells expressed both CD31 and Flag, while the discarded cells showed minimal expression of CD31 and Flag (Figure S4D,E, Supporting Information). Immunofluorescence results indicated that ZsGreen was primarily distributed in vascular endothelium, pericytes, and alveolar microvascular networks (Figure S4F, Supporting Information). Following validation, mice were intratracheally administered either control AAV or EC‐specific *Fads1/2*‐overexpressing AAV. Four weeks later, ALI was induced via airway delivery of 5mgkg^−1^ LPS or PBS control (Figure 4D). EC‐specific overexpression of *Fads1/2* attenuated body weight loss in mice following lung injury (Figure 4E). H&E staining with lung injury scoring and the lung wet‐to‐dry ratio demonstrated that *Fads1/2* overexpression of EC alleviated pathological manifestations, including tissue damage, inflammatory infiltration, and edema (Figure 4F,H). EC‐ *Fads1/2* overexpression reduced total cell count and protein concentration in bronchoalveolar lavage fluid (BALF) (Figure 4I,J). The elevated concentrations of pro‐inflammatory cytokines tumor necrosis factor‐alpha (TNF‐α) and interleukin‐6 (IL‐6) in plasma, as well as interleukin‐1 beta (IL‐1β), TNF‐α, and IL‐6 in BALF, were also reduced (Figure 4K,L). IHC analysis demonstrated that EC‐specific *Fads1/2* overexpression reduced the positive area of myeloperoxidase (MPO), indicating attenuated neutrophil infiltration following ALI (Figure 4M,N). In summary, in vivo experiments utilizing AAV‐mediated EC‐specific overexpression of Fads1/2 demonstrate that enhanced *Fads1/2* expression in pulmonary ECs confers protection against ALI.

### 2.5 Comprehensive Fads1/2 Overexpression with ALA Supplementation Attenuates LPS‐Induced ALI In Vivo

**Figure 5:**
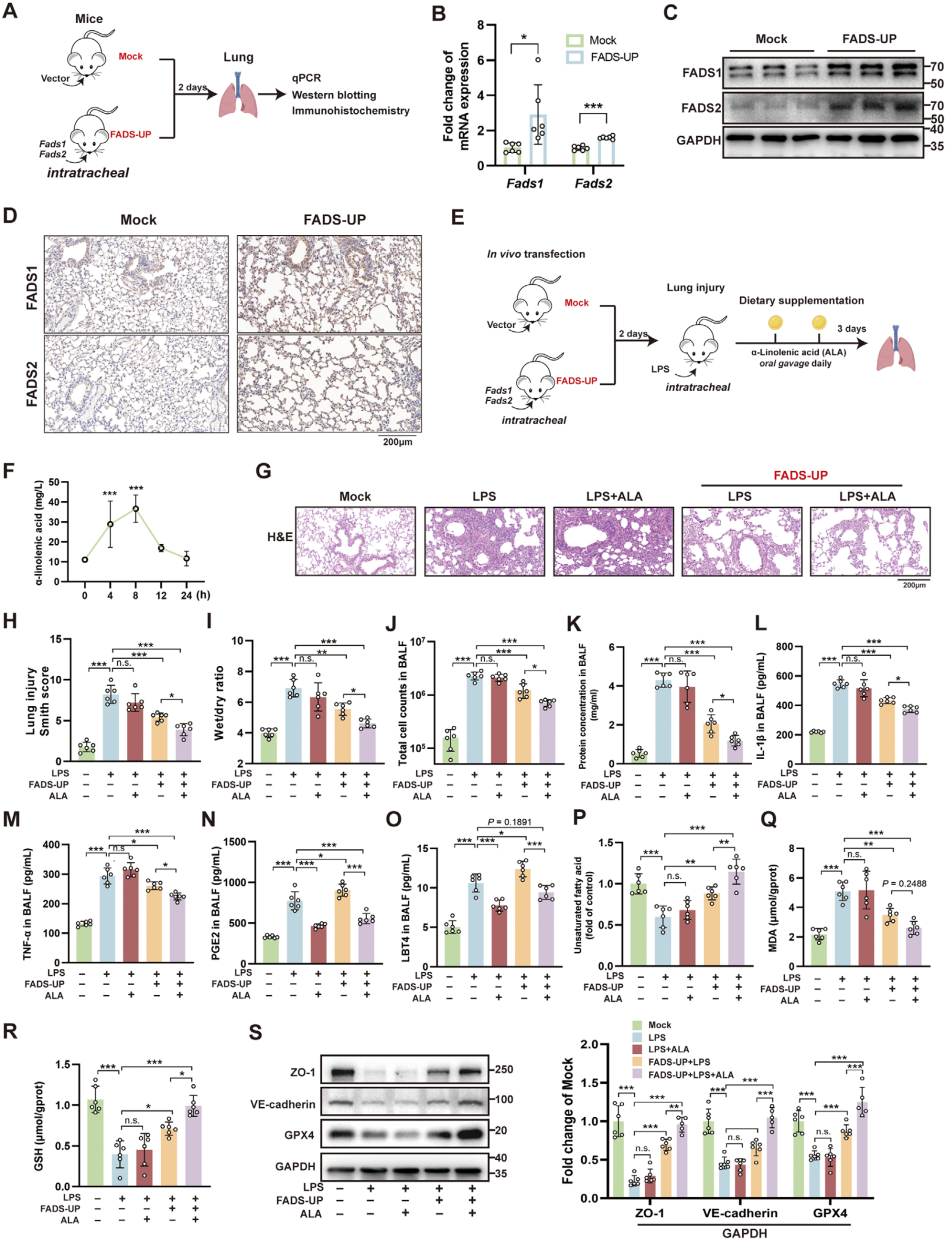
Overexpression of *Fads1/2* in mouse lungs with α‐linolenic acid (ALA) supplementation alleviates lipopolysaccharide (LPS)‐induced acute lung injury (ALI). A) Schematic diagram of the in vivo transfection procedure. B–D) Validation of *FADS1/2* upregulation after in vivo transfection by quantitative real‐time PCR (B), Western blot (WB) (C), and immunohistochemistry (D) (n = 6). E) Schematic illustration of the *Fads1/2* overexpression combined with ALA supplementation therapeutic strategy in the LPS‐induced acute lung injury (ALI) animal model. Following *Fads1/2* overexpression for two days, mice received intratracheal administration of LPS (5 mgkg^−1^). For ALA‐supplemented groups, ALA (100 mgkg^−1^) was administered via oral gavage at 24 h and 48 h post‐LPS challenge. All mice subjected to intratracheal LPS/phosphate‐buffered saline (PBS) instillation were subsequently euthanized for tissue collection. F) Changes in plasma ALA concentration in mice after oral gavage of 100 mgkg^−1^ ALA (n = 8). G) Hematoxylin and eosin (H&E) staining of harvested lung tissue. H) Lung injury scoring based on H&E staining (n = 6). I) Wet‐to‐dry weight ratio of lung tissue (n = 6). J–K) Total cell counts (J) and protein concentrations (K) in bronchoalveolar lavage fluid (BALF) (n = 6). L–O) Concentrations of IL‐1β (L), TNF‐α (M), PGE_2_ (N), and LTB_4_ (O) in BALF (n = 6). P) Relative content of unsaturated fatty acids per unit mass of lung tissue (n = 6). Q–R) Content of malondialdehyde (MDA) (Q) and reduced glutathione (GSH) (R) per unit protein mass in lung tissue (n = 6). S) WB analysis and quantification of ZO‐1, VE‐cadherin, and GPX4 expression levels in sorted endothelial cells from lung tissue (n = 6). Mock, intrapulmonary transfection with vector plasmid plus corresponding solvent treatment; FADS‐UP: Intrapulmonary transfection with *Fads1/2* overexpression plasmid. *, *P* < 0.05; **, *P* < 0.01; ***, *P* < 0.001.

To validate the therapeutic effect of restoring desaturase expression of the whole lung in LPS‐induced ALI, we also established a pulmonary *Fads1/2* overexpression mouse model (FADS‐UP) via intrapulmonary transfection, as detailed in the Methods section (**Figure** **5**A). qPCR, WB, and IHC collectively confirmed successful co‐overexpression of *Fads1/2* in mouse lung tissues (Figure 5B–D). In the therapeutic efficacy validation model, different experimental groups were established as follows: the Mock group received solvent treatment combined with intrapulmonary vector transfection; the LPS group underwent intratracheal instillation of LPS (5 mgkg^−1^) to induce acute lung injury; the LPS+ALA group received additional ALA supplementation post‐injury (100 mgkg^−1^ via oral gavage at 24 and 48 h post‐LPS administration); while the FADS‐UP groups were subjected to LPS challenge with or without ALA treatment following prior *Fads1/2* overexpression (achieved through intrapulmonary transfection 2 days before LPS administration) (Figure 5E). Gas chromatography results showed that ALA levels peaked at 8 h after gavage and returned to baseline within 24 hours in mouse plasma (Figure 5F). H&E staining and lung injury scoring demonstrated that ALA supplementation alone failed to alleviate ALI in non‐FADS‐UP conditions, whereas FADS‐UP significantly mitigated lung injury. Notably, additional ALA administration provided further protective effects in FADS‐UP mice (Figure 5G,H). Consistent with these findings, measurements of lung wet‐to‐dry weight ratio, total cell counts, and protein concentration in BALF revealed parallel improvements in pulmonary edema, cellular infiltration, and protein extravasation following this combined therapeutic approach (Figure 5I–K). In BALF, tumor necrosis factor‐alpha (TNF‐α) and interleukin‐1 beta (IL‐1β) were significantly reduced following FADS‐UP intervention, with further decreases observed after additional ALA supplementation (Figure 5L,M). However, for the omega‐6 fatty acid AA derivatives prostaglandin E_2_ (PGE_2_) and leukotriene B_4_ (LTB_4_), FADS‐UP increased their levels due to PUFA metabolic activation, though this effect was attenuated by ALA supplementation (Figure 5N,O). Furthermore, FADS‐UP significantly increased UFA content in lung tissue while reducing MDA levels and elevating reduced GSH levels (Figure 5P,R). These beneficial changes were further enhanced by ALA supplementation, although the additional reduction in MDA did not reach statistical significance. WB analysis of sorted pulmonary ECs revealed that FADS‐UP intervention restored the LPS‐ALI‐induced downregulation of ZO‐1, VE‐cadherin, and GPX4, with additional upregulation observed in the FADS‐UP+ALA co‐treatment group (Figure 5S). In summary, restoring pulmonary *Fads1/2* expression alleviates LPS‐induced ALI, with ALA providing additional protection and counteracting arachidonic acid (AA)‐mediated effects when desaturase activity is reconstituted.

### 2.6 PARK7 Exerts Endogenous Regulation on FADS1/2 Expression

**Figure 6:**
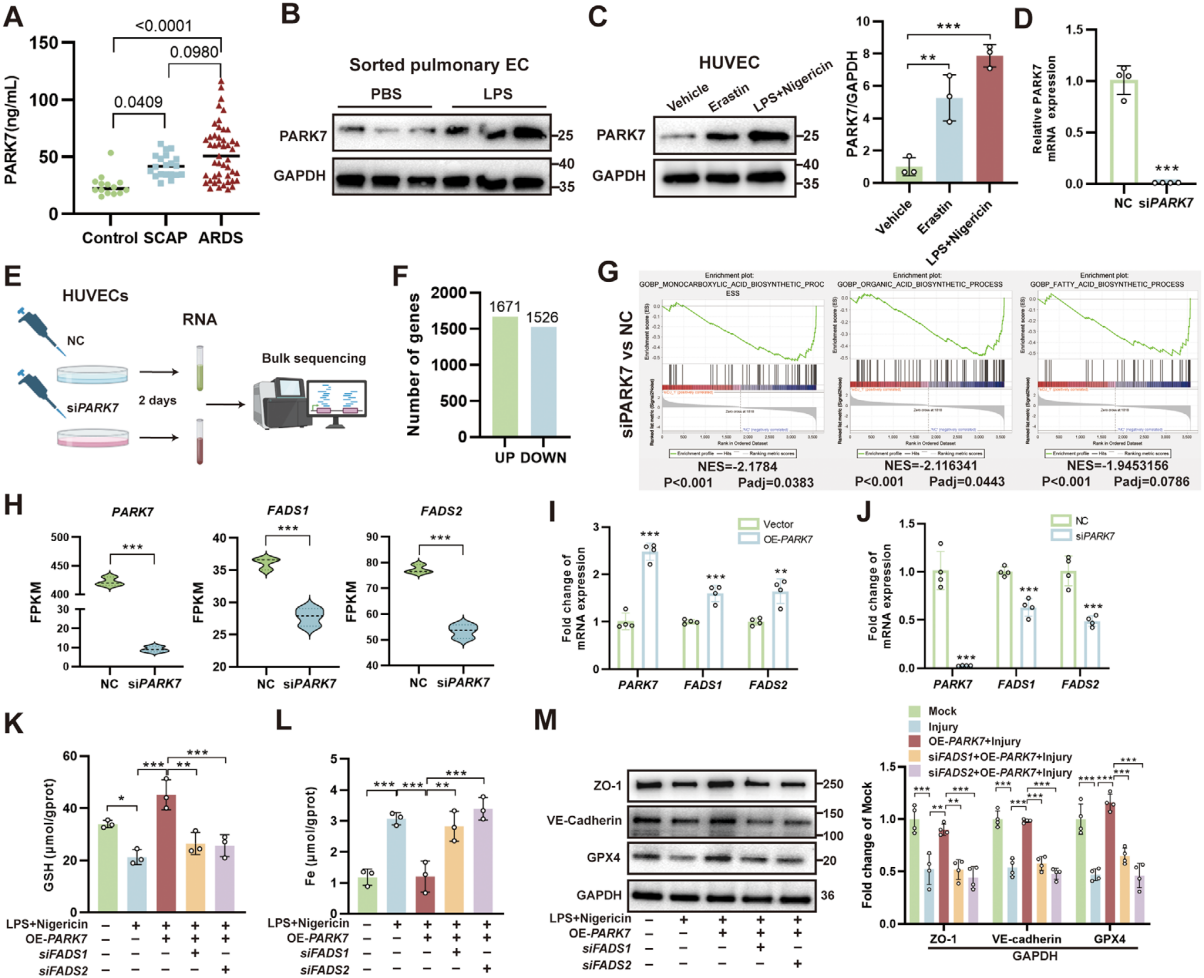
PARK7 acts as an endogenous regulator of FADS1/2 in endothelial cells. A) Plasma PARK7 concentrations of the control group (n = 14), SCAP (n = 20), and ARDS (n = 47) patients. B) Western blot (WB) analysis of PARK7 in sorted pulmonary endothelial cells. C) WB analysis and quantification of PARK7 expression in human umbilical vein endothelial cells (HUVECs) following different stimulations (n = 3). D) Quantitative real‐time PCR (qPCR) analysis of *PARK7* knockdown efficiency by specific siRNA (n = 4). E) Schematic diagram of RNA sequencing (RNA‐seq) in HUVECs transfected with negative control or PARK7‐specific siRNA. F) Number of differentially expressed genes (DEGs) from RNA‐seq. G) Gene set enrichment analysis (GSEA) of fatty acid metabolism‐related pathways in biological process pathway sets. H) Expression levels of *PARK7*, *FADS1*, and *FADS2* in RNA‐seq data (n = 3). I) qPCR analysis of *PARK7*, *FADS1*, and *FADS2* expression changes after *PARK7* overexpression plasmid transfection (n = 4). J) qPCR analysis of *PARK7*, *FADS1*, and *FADS2* expression changes after *PARK7* knockdown (n = 4). K–L) Changes in reduced glutathione (GSH) (K) and total iron content (L) in HUVECs under injury, *PARK7* overexpression, and *FADS1/FADS2* knockdown conditions (n = 3). M) WB analysis and quantification of ZO‐1, VE‐cadherin, and GPX4 expression in HUVECs under injury, *PARK7* overexpression, and *FADS1/FADS2* knockdown conditions (n = 4). ARDS, acute respiratory distress syndrome; SCAP, severe community‐acquired pneumonia; OE, overexpression; NC, negative control; Vector, transfection with vector plasmids; Mock, transfection with vector plasmids and addition of corresponding solvents. *, *P* < 0.05; **, *P* < 0.01; ***, *P* < 0.001.

To investigate the endogenous regulatory pathways of FADS1/2, we focused on the antioxidant protein PARK7, based on our previous findings demonstrating its critical role in mitigating endothelial oxidative stress and pyroptosis.^[^ ^28^ ^]^ Plasma PARK7 concentrations were significantly elevated in patients with SCAP and ARDS compared to the control group (**Figure** **6**A). Both sorted pulmonary ECs following LPS‐induced ALI and cultured HUVECs post‐ferroptosis or injury showed significant upregulation of PARK7 expression (Figure 6B,C). To investigate the role of PARK7 in lipid metabolism, siRNA was used to knock down PARK7, and the efficiency was validated by qPCR (Figure 6D). RNA extracted from HUVECs transfected with either negative control or PARK7‐targeting siRNA was subjected to RNA‐seq (Figure 6E). RNA‐seq analysis revealed a total of 1671 upregulated and 1526 downregulated DEGs following PARK7 knockdown (Figure 6F). KEGG pathway annotation analysis demonstrated that DEGs were significantly enriched in lipid metabolism and carbohydrate metabolism pathways (Figure S5A, Supporting Information). Specifically, DEGs associated with multiple distinct lipid metabolic pathways were identified (Figure S5B,C, Supporting Information). Gene Set Enrichment Analysis (GSEA) revealed significant downregulation of multiple fatty acid metabolism‐related biological processes following PARK7 knockdown, including monocarboxylic acid biosynthesis, organic acid biosynthesis, and fatty acid biosynthesis (Figure 6G; Figure S5D, Supporting Information). There were 20 overlapping genes between PARK7‐knockdown‐induced and ALI‐induced endothelial lipid metabolism‐related DEGs, including *FADS1* and *FADS2*, suggesting a potential regulatory relationship between PARK7 and these desaturases (Figure S5E, Supporting Information). RNA‐seq analysis demonstrated coordinated downregulation of *FADS1*, *FADS2*, and *PARK7* expression levels (Figure 6H). Further validation by qPCR and WB confirmed that overexpression or knockdown of *PARK7* in HUVECs led to corresponding upregulation or downregulation of both FADS1 and FADS2 at mRNA and protein levels (Figure 6I,J; Figure S5F,G, Supporting Information). Additionally, sodium phenylbutyrate (Na‐PBA), a PARK7 activator, concurrently upregulated both FADS1 and FADS2 expression in MPMECs (Figure S5H, Supporting Information). Subsequently, to validate that PARK7 modulates endothelial ferroptosis and function through FADS1/2, we generated and validated *FADS1/2*‐targeting siRNAs (Figure S2E—H, Supporting Information). PARK7 overexpression was found to directly increase reduced GSH levels, decrease iron content, and restore the LPS‐induced downregulation of ZO‐1, VE‐cadherin, and GPX4 in HUVECs (Figure 6K,M). Notably, these therapeutic effects were abolished by concurrent knockdown of either FADS1 or FADS2. These results demonstrate that PARK7 serves as a critical regulator of endothelial lipid metabolism and may influence endothelial ferroptosis through modulation of FADS1/2.

### 2.7 PARK7 Regulates FADS1/2 Transcription via a Dual‐Pathway Mechanism Targeting the BMP‐BMPR‐SMAD1/5/9 Signaling Cascade

**Figure 7:**
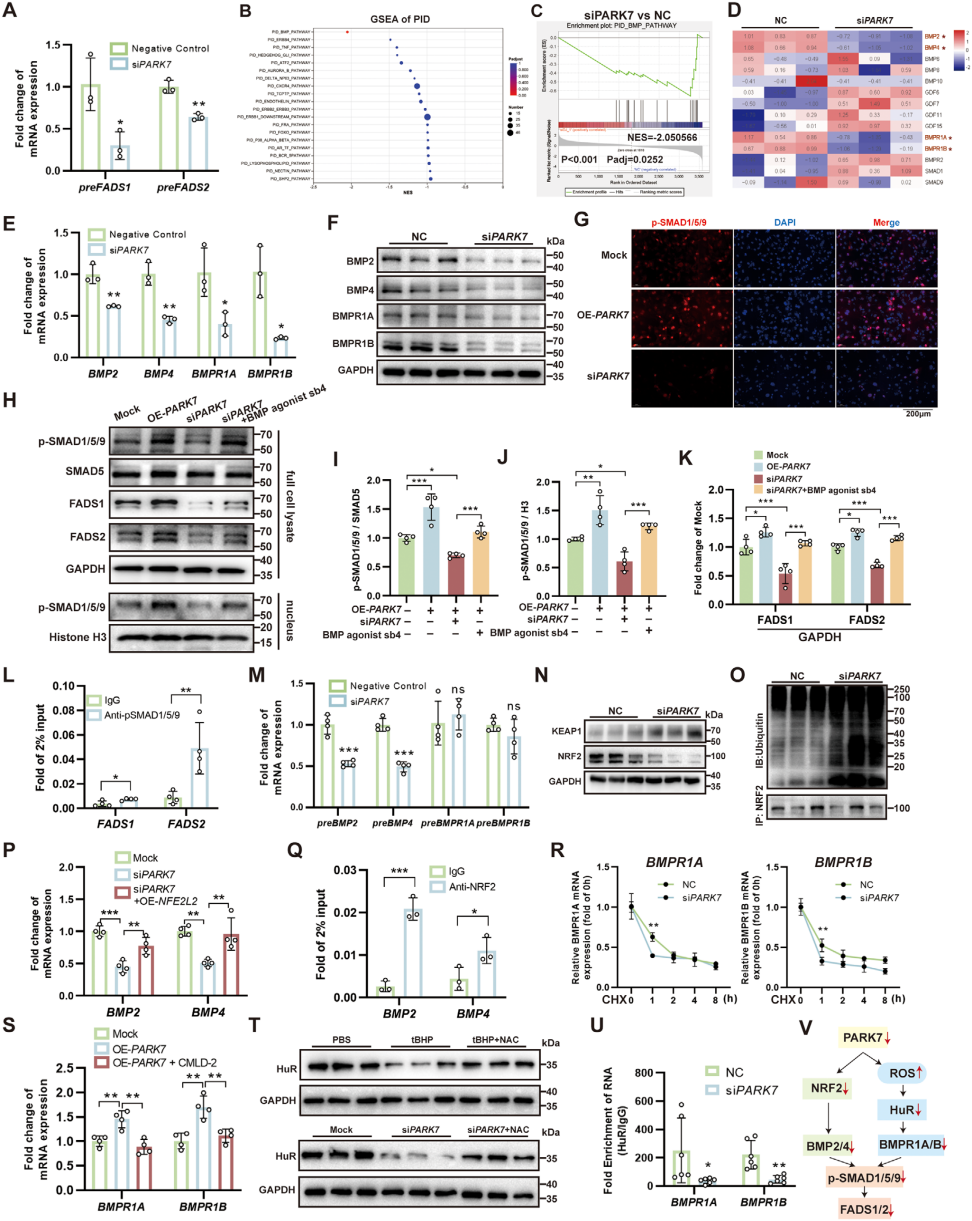
PARK7 regulates FADS1/2 expression by upregulating bone morphogenetic protein (BMP)‐SMAD1/5/9 signaling. A) Quantitative real‐time PCR (qPCR) analysis of the expression of *FADS1/2* precursor mRNA (n = 3). B) Gene set enrichment analysis (GSEA) of signaling pathways from the Pathway Interaction Database (PID). C) GSEA analysis of BMP signaling. D) Heatmap of expression levels for key BMP signaling molecules in human umbilical vein endothelial cells (HUVECs). E,F) qPCR (E) and Western blot (WB) (F) analysis of BMP2, BMP4, BMPR1A, and BMPR1B expression changes after *PARK7* knockdown in HUVECs (n = 3). G) Immunofluorescence detection of phosphorylated SMAD1/5/9 (p‐SMAD1/5/9) expression in HUVECs with *PARK7* overexpression or knockdown. H) WB analysis of expression levels of FADS1 and FADS2, the phosphorylation levels of SMAD1/5/9 in whole‐cell lysates, and the content of p‐SMAD1/5/9 in nuclear proteins were detected by WB in HUVECs overexpressing *PARK7*, knocking down *PARK7*, and treated with the BMP agonist sb4. I) WB quantification of the p‐SMAD1/5/9 to total SMAD5 ratio in HUVEC whole cell lysates (n = 4). J) WB quantification of nuclear p‐SMAD1/5/9 in HUVECs (n = 4). K) WB quantification of FADS1 and FADS2 in whole cell lysates of HUVECs (n = 4). L) ChIP‐qPCR validation of p‐SMAD1/5/9 binding to *FADS1/2* gene promoters (n = 4). M) qPCR analysis of the expression of *BMP2/4* and *BMPR1A/B* precursor mRNA (n = 4). N) WB analysis of NRF2 and KEAP1 after *PARK7* knockdown. O) CoIP analysis of NRF2‐ubiquitin conjugation status following *PARK7* knockdown. P) qPCR analysis of the expression of *BMP2/4* after *PARK7* knockdown and *NEF2L2* overexpression (n = 4). Q) ChIP‐qPCR validation of NRF2 binding to *BMP2/4* gene promoters (n = 3). R) Changes in BMPR1A/B mRNA levels over time after cycloheximide (CHX) administration (n = 3). S) qPCR analysis of the expression of *BMPR1A/B* after *PARK7* knockdown and CMLD‐2 administration (n = 4). T) Western blot analysis of HuR expression changes following tBHP stimulation or *PARK7* knockout, and the restorative effect of NAC. U) RIP assay validating changes in the binding capacity of HuR to BMPR1A/B mRNA (n = 5). V) Schematic diagram illustrating the dual pathways through which PARK7 regulates BMP/BMPR expression. CHX, cycloheximide; NAC, N‐Acetyl‐cysteine; tBHP, t‐Butyl hydroperoxide; ChIP, chromatin immunoprecipitation; CoIP, Co‐Immunoprecipitation; RIP, RNA immunoprecipitation; OE, overexpression; NC, negative control; Mock, transfection with vector plasmids and addition of corresponding solvents. *, *P* < 0.05; **, *P* < 0.01; ***, *P* < 0.001.

To further investigate the mechanisms through which PARK7 regulates FADS1/2 expression, we conducted qPCR analysis of *FADS1/2* precursor mRNA using HUVECs. The results indicated that *PARK7* knockdown led to reduced transcription of *FADS1/2* genes (**Figure** **7**A). However, there is no unambiguous evidence supporting PARK7's function as a transcription factor. To elucidate the regulatory mechanism of PARK7 on FADS1/2 expression, GSEA was performed on the RNA‐seq data using the Pathway Interaction Database (PID) gene set collection,^[^ ^38^ ^]^ revealing significant downregulation of the bone morphogenetic protein (BMP) signaling pathway (Figure 7B,C). Following *PARK7* knockdown, significant downregulation was observed in key BMP signaling components, including BMP2/4 ligands and BMPR1A/B receptors, which was further validated by qPCR and WB (Figure 7D–F). Subsequent IF analysis in HUVECs demonstrated that *PARK7* overexpression enhanced, whereas *PARK7* knockdown suppressed, nuclear localization of p‐SMAD1/5/9, which is the downstream effector of BMP signaling (Figure 7G). WB analysis revealed that *PARK7* overexpression enhanced both global SMAD1/5/9 phosphorylation and FADS1/2 expression, while concurrently increasing nuclear p‐SMAD1/5/9 levels (Figure 7H,K). Conversely, PARK7 knockdown suppressed these signaling events, an effect that was rescued by the BMP agonist sb4. These findings demonstrate that PARK7 regulates FADS1/2 expression through the BMP‐SMAD1/5/9 pathway. To further validate that BMP signaling directly regulates *FADS1/2*, we interrogated the chromatin immunoprecipitation (ChIP)‐seq based Cistome DB, which revealed that SMAD1/5 had been previously identified as potential transcriptional regulators of *FADS1/2* with comparable prediction scores in existing studies (Figure S6A, Supporting Information). To validate the direct binding of SMAD proteins to *FADS1/2* genes, the JASPAR database was employed to identify potential binding sites of SMAD5 on *FADS1/2* promoters (Table S2, Supporting Information). Finally, ChIP‐qPCR confirmed direct binding of p‐SMAD1/5/9 to specific promoter regions of *FADS1/2* (Figure 7L).

To further investigate how PARK7 regulates the expression of BMP‐BMPR pathway proteins, precursor mRNA levels were examined. The results revealed that *BMP2/4* transcription was affected, whereas *BMPR1A/B* transcription remained unchanged (Figure 7M). NRF2 is a previously confirmed transcription factor regulated by PARK7.^[^ ^25^ ^]^ We demonstrated that *PARK7* knockdown led to downregulation of NRF2/KEAP1 ratio, and co‐immunoprecipitation (CoIP) assays indicated increased ubiquitin binding to NRF2 (Figure 7N,O). Subsequently, overexpression of NRF2 (*NFE2L2* gene) resulted in upregulation of *BMP2/4*, while no synchronous upregulation was observed for *BMPR1A/B* or *FADS1/2* (Figure S6B,C, Supporting Information). Importantly, overexpression of NRF2 reversed the downregulation of *BMP2/4* induced by *PARK7* knockdown (Figure 7P). JASPAR predicted potential NRF2 binding sites on the *BMP2/4* promoters (Table S2, Supporting Information), and ChIP‐qPCR validation confirmed direct binding of NRF2 to these promoter regions (Figure 7Q). Furthermore, cycloheximide (CHX) treatment to inhibit mRNA synthesis revealed that *BMPR1A/B* mRNA degradation was accelerated during the early administration phase following *PARK7* knockdown, suggesting that PARK7 may influence the stability of these transcripts (Figure 7R). RBPDB (http://rbpdb.ccbr.utoronto.ca/) prediction of RNA‐binding protein sites on *BMPR1A/B* mRNA revealed abundant HuR binding motifs in both transcripts (Figure S6D, Supporting Information). Notably, HuR is an RNA‐stabilizing protein that typically enhances mRNA stability and translational efficiency by binding to AU‐rich elements (AREs) in target transcripts.^[^ ^39^ ^]^ Treatment with the HuR‐ARE inhibitor CMLD‐2 reduced the expression of mature *BMPR1A/B* mRNA and reversed the upregulation of *BMPR1A/B* induced by *PARK7* overexpression (Figure 7S; Figure S6E, Supporting Information). As PARK7 functions as a ROS scavenger, we found that its knockdown led to increased ROS burden, which could be reversed by the antioxidant N‐acetylcysteine (NAC) (Figure S6F, Supporting Information). Further experiments demonstrated that oxidative stress induced by tBHP (tert‐butyl hydroperoxide) treatment or *PARK7* knockdown led to downregulation of HuR, which could be rescued by NAC (Figure 7T). This indicates that PARK7 knockdown may regulate HuR expression through ROS‐mediated mechanisms. Additionally, RNA Immunoprecipitation (RIP) assays demonstrated that *PARK7* knockdown significantly attenuated the binding capacity of HuR to *BMPR1A/B* mRNA (Figure 7U). Collectively, these results indicate that PARK7 regulates BMP‐BMPR expression through dual mediators, NRF2 and HuR, thereby influencing SMAD1/5/9 phosphorylation and ultimately modulating *FADS1/2* expression (Figure 7V).

### 2.8 Endothelial Histone H3K14 Lactylation Directly Activates PARK7 Transcription Upon Injury

**Figure 8:**
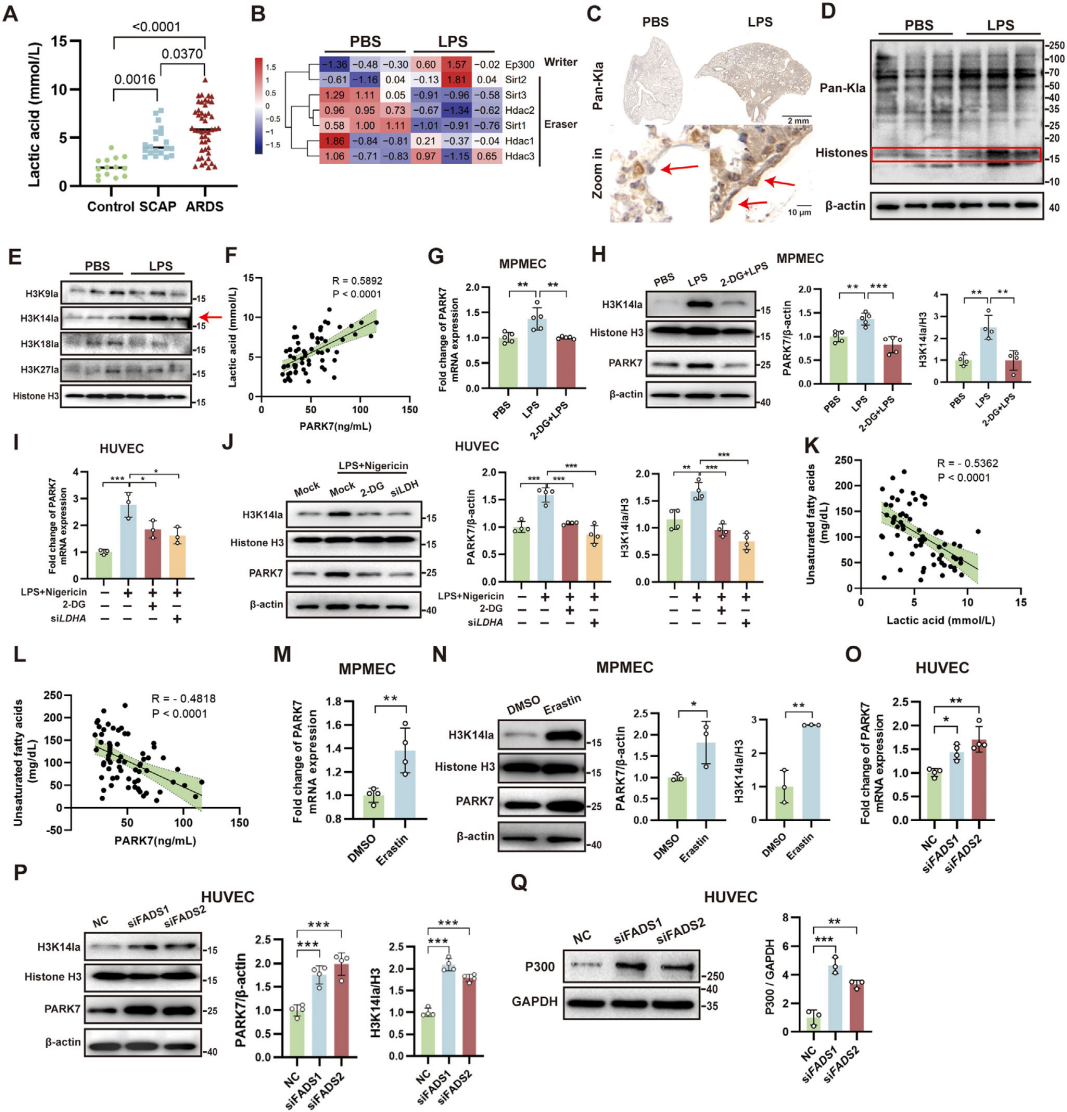
Histone lactylation upregulates PARK7 expression in endothelial cells (ECs) under injury conditions. A) Plasma lactate concentrations in the control group (n = 14), SCAP (n = 20), and ARDS (n = 47) patients. B) Changes in lactylation "Writer" and "Eraser" enzyme expression in sorted pulmonary endothelial RNA‐seq data. C) Immunohistochemical (IHC) staining of pan‐lysine lactylation (pan‐Kla) in lipopolysaccharide (LPS)‐induced lung injury. Red arrows indicate endothelial cells. D) Western blot (WB) analysis of pan‐Kla in sorted pulmonary ECs. The red box marks the localization of histones. E) WB analysis of histone H3 lysine lactylation at specific sites in sorted pulmonary ECs. The red arrow highlights the most significantly upregulated lactylation site. F) Correlation analysis between plasma lactate concentration and PARK7 levels in SCAP and ARDS patient samples. G) Quantitative real‐time PCR (qPCR) analysis of PARK7 expression in mouse pulmonary microvascular endothelial cells (MPMECs) after LPS and 2‐deoxy‐D‐glucose (2‐DG) stimulation (n = 5). H) WB analysis of H3K14 lactylation and PARK7 expression in MPMECs after LPS and 2‐deoxy‐D‐glucose (2‐DG) stimulation (n = 5). I) qPCR analysis of PARK7 expression in human umbilical vein endothelial cells (HUVECs) after LPS, nigericin, and 2‐DG stimulation or *LDHA* knockdown (n = 3). J) WB analysis of H3K14 lactylation and PARK7 expression in HUVECs after LPS, nigericin, and 2‐DG stimulation or *LDHA* knockdown (n = 4). K–L) Correlation analysis of plasma unsaturated fatty acids with lactate levels (K) and PARK7 (L) in SCAP and ARDS patients. M) qPCR analysis of PARK7 expression in MPMECs after erastin stimulation (n = 4). N) WB analysis of H3K14 lactylation and PARK7 expression in MPMECs after erastin stimulation (n = 3). O) qPCR analysis of PARK7 expression in HUVECs after *FADS1* or *FADS2* knockdown (n = 4). P) WB analysis of H3K14 lactylation and PARK7 expression in HUVECs after *FADS1* or *FADS2* knockdown (n = 4). Q) WB analysis of P300 expression in HUVECs after *FADS1* or *FADS2* knockdown (n = 3). ARDS, acute respiratory distress syndrome; SCAP, severe community‐acquired pneumonia; NC, negative control; Mock, transfection with vector plasmids and addition of corresponding solvents. *, *P* < 0.05; **, *P* < 0.01; ***, *P* < 0.001.

To investigate the mechanism underlying PARK7 upregulation following endothelial injury, we examined lactate production and lactylation modifications under inflammation conditions. Patients with SCAP and ARDS exhibited significantly elevated plasma lactate concentrations (**Figure** **8**A). RNA‐seq data of pulmonary ECs revealed upregulation of the lactylation “writer” gene *Ep300* alongside downregulation of “eraser” genes (*Sirt1/3*, *Hdac2*) under inflammatory conditions (Figure 8B). Immunohistochemical analysis demonstrated enhanced lysine lactylation in both whole lung tissues and endothelial cells (Figure 8C). Subsequent WB analysis of sorted pulmonary ECs demonstrated a global increase in protein lactylation post‐ALI, with elevated modification levels observed at 17 kDa (histone fraction), 50, and 70 kDa molecular weights (Figure 8D). Further targeted analysis of histone H3 lactylation sites revealed particularly pronounced upregulation of H3K14 lactylation (H3K14la) (Figure 8E). Correlation analysis revealed a statistically significant positive association between plasma PARK7 levels and lactate concentrations in patients (r = 0.5892, *P*<0.0001) (Figure 8F). To further elucidate the impact of histone lactylation on PARK7 expression, we employed 2‐Deoxy‐D‐glucose (2‐DG) treatment or *LDHA* knockdown to inhibit lactate production. In MPMECs, 2‐DG treatment reversed the LPS‐induced upregulation of both H3K14la and PARK7 expression (Figure 8G,H). Similarly, either 2‐DG administration or *LDHA* knockdown abolished the LPS injury‐triggered increase in H3K14la and PARK7 levels in HUVECs (Figure 8I,J). Furthermore, in SCAP and ARDS patients, plasma UFA levels showed significant negative correlations with both lactate concentrations (r = ‐0.4818, *P*<0.0001) and PARK7 levels (r = ‐0.5362, *P*<0.0001) (Figure 8K,L). In MPMECs, erastin‐induced ferroptosis triggered significant upregulation of both H3K14la and PARK7 expression (Figure 7M,N). In HUVECs, knockdown of either *FADS1* or *FADS2* induced upregulation of H3K14la and PARK7 expression (Figure 8O,P). *FADS1/2* knockdown also upregulated the expression of lactylation “writer” P300 (Figure 8Q). These results demonstrate that H3K14la upregulation in ECs induced by LPS‐ALI drives PARK7 overexpression and impairment of the PUFA metabolic pathway may trigger a negative feedback loop.

**Figure 9:**
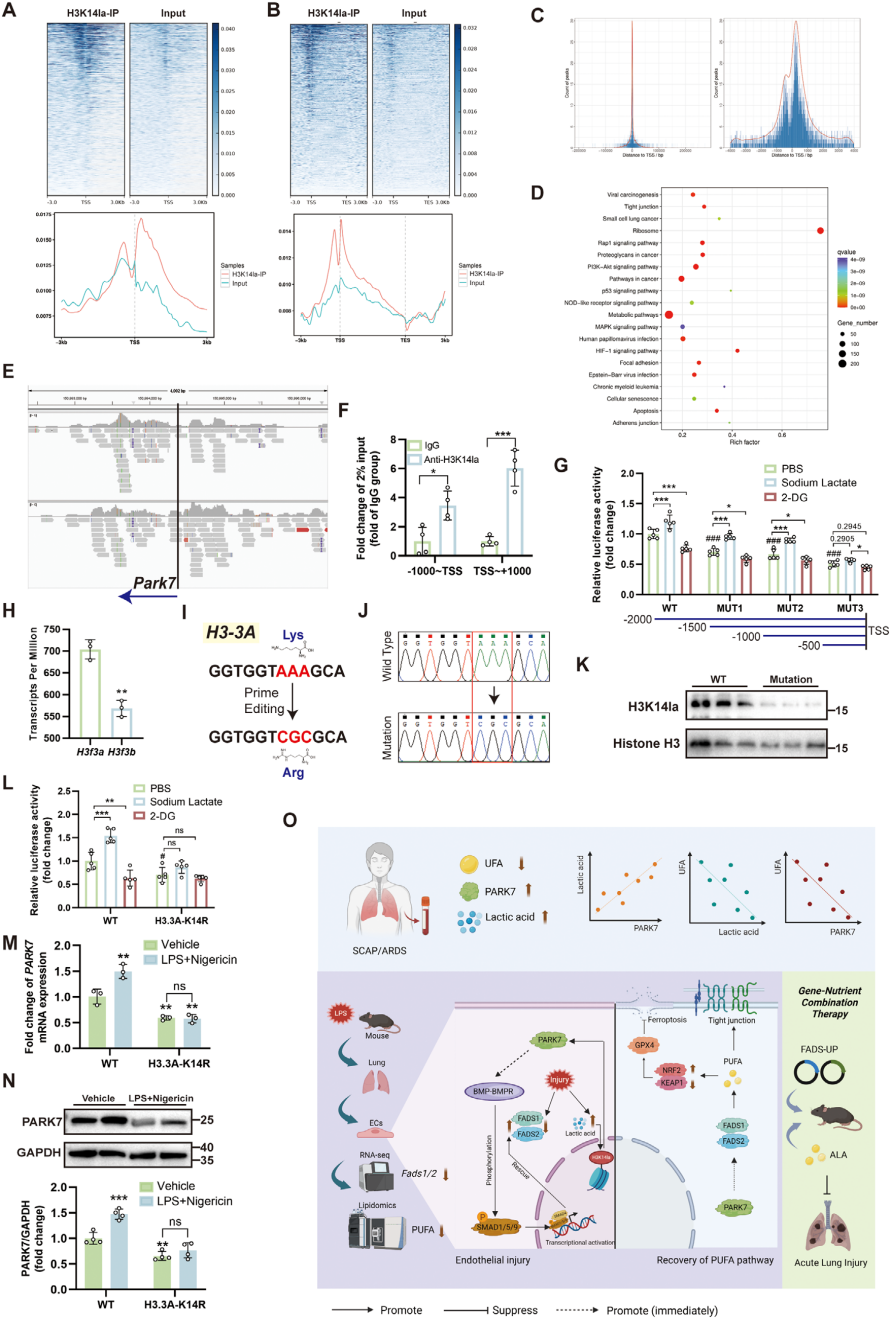
H3K14 lactylation directly regulates PARK7 transcription by binding to sequences near its TSS. A,B) Distribution of reads relative to gene TSS (A) and gene body (B) in H3K14la‐based ChIP‐sequencing (ChIP‐seq). C) Genomic distribution of ChIP‐seq peaks relative to gene TSS positions. D) KEGG enrichment analysis of ChIP‐seq peak‐associated genes. E) Distribution of ChIP‐seq reads near the TSS region of the mouse *Park7* gene. F) ChIP‐qPCR validation of H3K14la binding to the genomic regions spanning ‐1000 to TSS and TSS to +1000 of the *PARK7* gene in human umbilical vein endothelial cells (HUVECs) (n = 4). G) Dual‐luciferase reporter assay validating changes in lactylation regulatory capacity upon segmental deletion of the *PARK7* promoter sequence in 293T cells (n = 5). #, compared with the wild‐type PBS‐treated group. H) Comparison of transcripts per million (TPM) values between *H3f3a* and *H3f3b* in primary pulmonary endothelial cells (n = 3). I) Schematic diagram of introducing a point mutation at lysine 14 (K14) of the H3‐3A gene in 293T cells using a prime editing strategy. J) Third‐generation sequencing verification of monoclonal mutation status. K) Western blot (WB) analysis of H3K14la expression after mutation. L) Dual‐luciferase reporter assay validating changes in lactylation regulatory capacity upon H3.3A K14R mutation. #, compared with the wild‐type PBS‐treated group (n = 5). M,N) qPCR (M, n = 3) and WB (N, n = 4) analysis of PARK7 expression in wild‐type and mutant 293T cells under normal and injured conditions. *, compared with the wild‐type vehicle‐treated group. O) The schematic diagram of this study. Patients with ARDS show decreased UFAs, elevated PARK7, and lactate, which are correlated. Pulmonary endothelial cells exhibit downregulated PUFA metabolism. Injury triggers a H3K14la‐PARK7 feedback loop by suppressing FADS1/2, while PARK7 restores FADS1/2 via BMP‐BMPR‐SMAD1/5/9 activation. PUFA supplementation or FADS1/2 overexpression reduces ferroptosis and junction disruption, alleviating lung injury. This image was created using BioRender. TSS, transcription start site; WT, wild type; 2‐DG, 2‐deoxy‐d‐glucose; qPCR, real‐time quantitative PCR; UFA, unsaturated fatty acid; PUFA, polyunsaturated fatty acid. #, *, *P* < 0.05; ##, **, *P* < 0.01; ###, ***, *P* < 0.001.

To further investigate the direct regulatory role of H3K14la on PARK7 gene expression, we performed H3K14la ChIP‐seq analysis using MPMECs (n = 1). The read lengths of both IP and input samples ranged from 100 to 500 bp and were distributed across the entire mouse genome (Figure S7A,B, Supporting Information). Compared to the Input, reads from the IP samples were predominantly enriched in proximal promoter regions and the upstream sections of gene bodies, primarily localized within a ±3000 bp window around transcription start sites (TSS) (**Figure** **9**A,B). The peaks obtained from ChIP‐seq were primarily located within a ±1000 bp region around gene TSS and were distributed across all autosomes (Figure 9C; Figure S7C, Supporting Information). Moreover, these peaks were predominantly distributed within promoter regions (≤1000 bp from TSS), accounting for 37.15% of all peaks (Figure S7D, Supporting Information). The predicted motifs for H3K14la binding are presented in Figure S7E (Supporting Information). KEGG enrichment analysis of peak‐associated genes revealed that H3K14la may participate in regulating critical pathways and biological processes, including hypoxia response, metabolism, cell death, and cell junction organization, which are implicated in important diseases such as cancer and infections (Figure 9D). By locating the TSS of the mouse *Park7* gene, we observed that reads from the IP sample were primarily distributed within a ±1000 bp window around the *Park7* TSS^[^NC_000070.7 (150 994 378) in Chr 4] (Figure 9E). Based on this finding, we designed primers targeting two regions flanking the TSS (upstream and downstream within 1000 bp) and performed ChIP‐qPCR validation in HUVECs. The results confirmed that H3K14la binds to both the upstream and downstream regions within 1000 bp of the *PARK7* gene TSS (Figure 9F). Subsequently, we confirmed that sodium lactate and 2‐DG could upregulate and downregulate H3K14la levels in 293T cells, respectively (Figure S7E, Supporting Information). Using dual‐luciferase reporter assays in 293T cells, we validated the transcriptional activation activity of progressively truncated *PARK7* promoter regions. The results demonstrated that lactylation‐level modulation within the retained ‐1000 to 0 region significantly altered transcriptional activity, whereas truncation to the −500 to 0 region markedly impaired this lactylation‐dependent transcriptional regulation (Figure 9G). Then, to demonstrate the specificity of H3K14 lactylation in regulating PARK7 expression, we conducted prime editing‐mediated amino acid point mutation assays. Histone H3 exists in multiple variants, with H3.3 being a replication‐independent histone that is highly expressed during the G0 phase. Sequencing analysis of pulmonary ECs revealed higher expression of the *H3f3a* gene (encoding H3.3A) compared to *H3f3b* (encoding H3.3B). Therefore, the human ortholog *H3‐3A* gene was selected for mutagenesis (Figure 9H). We introduced a lysine‐to‐arginine mutation at the target site by co‐transfecting plasmids encoding the pegRNA vector and the prime editing system (Figure 9I; Figure S7G, Supporting Information). Following transfection in 293T cells, puromycin selection, and monoclonal isolation, third‐generation sequencing identified successfully mutated cells for subsequent experiments (Figure 9J; Figure S7H, Supporting Information). Following the mutation, H3K14la expression was significantly downregulated (Figure 9K). Moreover, dual‐luciferase reporter assays demonstrated that the regulatory effect of lactylation levels on *PARK7* promoter activity was markedly reduced post‐mutation (Figure 9L). qPCR and WB analyses revealed that PARK7 expression decreased but was not completely abolished after mutation (Figure 9M,N). However, the upregulation of PARK7 in response to cellular injury was impaired, indicating that H3K14la is a critical factor for injury‐induced PARK7 upregulation.

## 3 Discussion

The present study elucidates a multifaceted regulatory network involving PUFA metabolism, ferroptosis, and lactylation‐driven epigenetic reprogramming in the pathogenesis of ARDS/ALI. Following ALI, ECs exhibit two key metabolic alterations: the inhibition of PUFA synthesis and the enhancement of lactylation. These changes are interconnected and regulated through PARK7. Specifically, the suppression of FADS1/2 activity leads to an increase in H3K14 lactylation. In turn, this elevated lactylation upregulates PARK7, which subsequently restores FADS1/2 expression and rescues PUFA synthesis. Through integrated ChIP‐seq and point mutation analyses, we confirmed the direct transcriptional regulatory role of H3K14la on PARK7. Our findings reveal that endothelial dysfunction in ALI is mechanistically linked to desaturase‐mediated lipid metabolic perturbations, ferroptotic cell death, and lactate‐dependent histone modifications, with PARK7 serving as a pivotal node integrating these pathways. The schematic diagram summarizing the key findings of this study is presented in Figure 9O.

Transcriptomic sequencing and lipidomics of pulmonary ECs after ALI confirmed the downregulation of PUFA content and FADS1/2 expression. In clinical patient plasma, the levels of UFAs were significantly reduced in SCAP and ARDS patients and were clearly correlated with the degree of severity. These findings are consistent with previous clinical research conclusions that the levels of unsaturated fatty acids in the plasma of ARDS patients are reduced and correlated with disease severity.^[^ ^11^ ^]^ Additionally, different types of endothelial cells (MPMEC, HUVEC) were used to validate the downregulation of FADS1/2 following injury. To confirm the roles of FADS1/2 and PUFA in endothelial cells during injury, we conducted pharmacological treatment experiments using MPMECs and performed gene transfection intervention experiments using HUVECs. In MPMECs, LPS directly induces cellular injury. Subsequent to injury, cell viability was significantly decreased, while GSH levels were reduced. Additionally, MDA and total iron levels were elevated, resulting in enhanced lipid peroxidation. Notably, these alterations were effectively reversed by the ferroptosis inhibitor ferrostatin‐1, thereby implicating ferroptosis as a key contributor to LPS‐induced endothelial injury. SC‐26196, an inhibitor of FADS2, reduces the production of PUFAs such as DHA.^[^ ^40^ ^]^ Administration of SC‐26196 exacerbated various ferroptosis‐related phenotypes, whereas pretreatment with DHA alleviated LPS‐induced ferroptosis. Similarly, we overexpressed *FADS1* or *FADS2* in HUVECs and found that this directly alleviated ferroptosis and reduced various ferroptosis‐related phenotypes induced by co‐treatment with LPS and nigericin. Thus, we have demonstrated that FADS1/2 and PUFAs play a protective role against ferroptosis in endothelial cells following injury. Further experiments suggested that FADS1/2 and PUFAs may exert their effects by resisting ferroptosis during injury, thereby restoring endothelial cell junction function, angiogenic capacity, and normal marker expression.

Some studies have reported findings similar to ours. For instance, inhibition of FADS2 has been shown to promote ferroptosis in tumor cells.^[^ ^14^, ^15^ ^]^ Overexpression of *FADS2* in mesenteric adipocytes has been demonstrated to suppress inflammatory responses.^[^ ^16^ ^]^ Conversely, in some cases, the activation of FADS1/2 and PUFAs has been shown to exert cell‐damaging effects. In mesenchymal gastric cancer cells, the upregulation of FADS1 expression leads to sensitization to ferroptosis.^[^ ^21^ ^]^ The dual FADS1/2 inhibitor CP‐24879 exhibits anti‐inflammatory effects in a mouse mastocytoma model.^[^ ^41^ ^]^ Studies on viral hepatitis have shown that increased expression of FADS2 enhances ferroptosis sensitivity and increases susceptibility to antiviral drugs.^[^ ^42^ ^]^ The existence of such contradictions is understandable. On one hand, PUFAs serve as essential substrates for lipid peroxidation, and their increased levels enhance cellular susceptibility to ferroptosis. On the other hand, PUFAs themselves possess reducing properties and participate in fundamental cellular life processes and structures, making them indispensable for cellular homeostasis. To clarify the protective role of the PUFA pathway on the endothelium, we investigated its mechanism in safeguarding against ferroptosis. Previous studies have shown that EPA and DHA supplementation can reduce ferroptosis in neurons of epilepsy conditions by activating NRF2.^[^ ^43^, ^44^ ^]^ This effect may be related to maresin 1, an intermediate metabolite of DHA.^[^ ^45^ ^]^ Our study demonstrates that both direct DHA administration and FADS1/2 overexpression can increase the NRF2/KEAP1 ratio in endothelial cells, indicating NRF2 signaling activation. Therefore, PUFAs in endothelial cells may also inhibit ferroptosis through NRF2 pathway activation. Meanwhile, we compared the responses of endothelial cells and lung epithelium‐derived tumor cell lines (LLC and A549) to DHA treatment. The results showed that both endothelial cell lines were protected from erastin‐induced injury at low DHA concentrations (<20 or 10 µM), whereas high DHA concentrations caused significant cellular damage. In contrast, tumor cells exhibited greater tolerance to high DHA levels. However, A549 cells showed exacerbated damage at concentrations >20 µM. These findings suggest that the dual effects of DHA may depend on cell type and concentration. It remains unclear whether DHA functions through receptor binding or direct interactions, warranting further investigation in future studies.

Subsequently, we conducted in vivo validation through two experimental approaches. First, an animal model with AAV‐mediated endothelial‐specific overexpression of *Fads1/2* was used to confirm the critical role of endothelial FADS1/2 enzymes in protecting against lung injury. Second, a whole‐lung *Fads1/2* overexpression combined with ALA supplementation model was established to lay the groundwork for future therapeutic applications of FADS1/2 activators across the entire lung, while also clarifying the conditions under which ALA supplementation is effective. The results demonstrated that both models significantly alleviated LPS‐induced ALI. Several studies attribute the multifaceted effects of PUFAs on cellular injury to the ratio of omega‐3 to omega‐6 fatty acids, as the AA metabolites derived from omega‐6 fatty acids can significantly promote ferroptosis and inflammatory activation.^[^ ^20^ ^]^ In non‐alcoholic steatohepatitis (NASH) models, an imbalance in the omega‐6 to omega‐3 ratio was observed, while omega‐3 supplementation can alleviate hepatocyte inflammatory responses and lipid peroxidation.^[^ ^46^ ^]^ Paolo et al.^[^ ^47^ ^]^ exposed A549 cells to BALF collected from ARDS patients, supplemented with DHA and AA at different ratios. They found that a 1:2 DHA/AA supplementation ratio provided protection against inflammatory responses in A549 cells compared to the 1:7 ratio. In our study, we overexpressed FADS1/2 in pulmonary tissues via in vivo transfection to validate the impact of PUFA pathway restoration on ALI. Concurrently, we administered ALA supplementation to shift the PUFA metabolic focus from omega‐6 to omega‐3 fatty acids. Our findings demonstrated that *Fads1/2* overexpression alleviated multiple pulmonary injury phenotypes, including endothelial GPX4 downregulation and junctional impairment, with ALA co‐supplementation further enhancing protection against LPS‐induced ALI. Notably, while *Fads1/2* overexpression promoted AA metabolism (evidenced by elevated PGE_2_ and LTB_4_ levels in BALF), ALA supplementation partially normalized these mediators. Consequently, to counteract the pro‐ferroptotic effects of PUFAs, combined strategies involving omega‐3 fatty acid activation/supplementation may serve as an effective therapeutic approach.

However, although the protective role of FADS1/2 in ALI has been established, the lack of specific and effective FADS1/2 activators has led us to investigate its endogenous regulatory factors in greater depth. PARK7/DJ‐1, a Parkinson's disease‐associated protein, exhibits antioxidative properties in multiple pathological conditions, including ALI, and interacts with critical signaling molecules such as Akt and NRF2 that regulate essential biological processes.^[^ ^24^, ^25^, ^26^, ^27^ ^]^ PARK7 negatively regulates ferroptosis by modulating GSH biosynthesis.^[^ ^48^ ^]^ Through genetic knockout and overexpression approaches, we investigated the relationship between PARK7 and FADS1/2. Multiple experimental techniques, including RNA sequencing, qPCR, and WB analysis, demonstrated their coordinated expression. Importantly, the anti‐ferroptotic function of PARK7 was found to be partially dependent on FADS1/2 expression. These findings not only expand the endogenous regulatory network of FADS1/2 but also reveal a novel mechanism by which PARK7 confers resistance to ferroptosis. However, transcription of FADS1/2 was suppressed following PARK7 knockout, necessitating the identification of a relevant transcription factor‐associated pathway to establish the regulatory cascade. Through GSEA, we identified the BMP signaling pathway as the critical link between PARK7 and FADS1/2. The presence of PARK7 was essential for maintaining p‐SMAD1/5/9 levels, which showed a positive correlation with FADS1/2 expression. Subsequent analysis of ChIP‐seq databases followed by ChIP‐qPCR validation demonstrated direct transcriptional regulation of FADS1/2 by this signaling cascade. Recent studies have demonstrated that BMP signaling participates in regulating endothelial cell function in various pulmonary diseases, including pulmonary fibrosis and pulmonary arterial hypertension.^[^ ^49^, ^50^ ^]^ Furthermore, through detailed investigation, we found that PARK7 regulates BMP2/4 and BMPR1A/B through distinct mechanisms: it modulates *BMP2/4* transcription via NRF2, a key interacting partner, while it regulates *BMPR1A/B* mRNA stability through the expression and binding activity of the RNA‐binding protein HuR. Indeed, our previous research revealed that PARK7 not only reduces NRF2 degradation and enhances its expression, but NRF2 can also activate PARK7, forming a positive feedback loop. However, this study did not observe that *NEF2L2* overexpression directly upregulated FADS1/2, leading us to adopt a more comprehensive albeit complex explanatory pathway. Our findings further enrich the understanding of the role of BMP signaling in endothelial cells in acute pulmonary diseases.

Finally, we investigated why PARK7, as a protective factor, is significantly upregulated in ALI, which may imply the existence of a negative feedback regulatory mechanism. Our analysis revealed an inverse correlation between PARK7 and lactate (a hypoxia byproduct), prompting us to explore the potential activation of lactylation modifications in endothelial cells during lung injury. Gong et al.^[^ ^35^ ^]^ discovered that sepsis‐induced ALI is associated with elevated endothelial H3K14 lactylation, which facilitates the transcription of ferroptosis‐related genes and ultimately promotes ferroptosis activation. Lu et al.^[^ ^36^ ^]^ revealed that both H3K18 lactylation and EGR1 lactylation promote glycocalyx degradation in endothelial cells during sepsis‐induced ALI, thereby exacerbating ALI progression. Our study confirmed through WB analysis that H3K14 is a prominently upregulated lactylation site in pulmonary ECs within the ALI model. Furthermore, by inhibiting lactate production (via 2‐DG administration or *LDHA* knockdown), we demonstrated synchronized regulation between PARK7 expression and lactylation levels. Subsequently, we observed a negative correlation between plasma UFA levels and both lactate and PARK7 expression. Further validation experiments revealed that under conditions of ferroptosis induction or *FADS1/2* knockdown, both H3K14la and PARK7 expression were significantly enhanced. Intriguingly, FADS1/2 deficiency upregulated P300, the key enzyme responsible for lactylation modification. These findings demonstrate that downregulation of the PUFA biosynthesis pathway and its induced ferroptosis can reciprocally upregulate PARK7 expression through histone lactylation‐mediated feedback mechanisms.

ChIP‐seq analysis targeting H3K14la revealed that its reads and binding peaks were primarily located flanking gene TSS regions, indicating a role in transcriptional regulation. This pattern held true for the *Park7* gene as well, where we validated specific H3K14la binding to both the ‐1000 to TSS and TSS to +1000 regions via ChIP‐qPCR. Enrichment analysis suggested that H3K14la may regulate additional hypoxia‐ and metabolism‐related molecules, warranting further exploration and validation in human lung ECs. To ensure the specificity of the H3K14 site, we employed prime editing to introduce point mutations. Given the existence of multiple histone H3 variants, previous studies often used universal tools^[^ ^51^ ^]^ or focused on H3.3 mutations,^[^ ^52^ ^]^ a replication‐independent variant. We selected H3.3A, which is more abundant in ECs, for mutagenesis. Following mutation, H3K14la expression was impaired, leading to partial loss of basal PARK7 expression and abolished injury‐induced upregulation. This indicates that while H3K14la is not the sole factor maintaining basal PARK7 expression, it is a major regulator of its upregulation following injury. The dual‐luciferase reporter assay provided cross‐validation of the interaction between H3K14la and the *PARK7* promoter. Both deletion of the ‐1000 to TSS promoter region and point mutation of the H3K14 site resulted in dysregulated lactylation‐dependent modulation, demonstrating the specificity of this interaction. Thus, our findings establish a novel metabolic circuit linking two crucial reprogramming events in ALI‐affected ECs: 1) endothelial injury triggers concurrent PUFA pathway downregulation and lactate overproduction, 2) lactate‐derived histone lactylation upregulates PARK7 to rescue FADS1/2 expression, while 3) FADS1/2 deficiency itself further amplifies PARK7 activation. This self‐reinforcing loop represents a previously unrecognized compensatory mechanism in vascular homeostasis.

While our study elucidates a novel PARK7‐FADS1/2‐lactylation axis in ALI pathogenesis, several limitations warrant consideration. First, while our control group consisted of patients with non‐critical respiratory diseases to ensure clinical relevance, the inclusion of healthy volunteers could have provided a more robust baseline for metabolic comparisons. Second, while we demonstrated the therapeutic potential of omega‐3 PUFA supplementation, the optimal dosing, timing, and formulation (e.g., DHA versus EPA ratios) for clinical intervention need systematic optimization. Moreover, the precise mechanisms by which omega‐3 fatty acids inhibit ferroptosis via the NRF2 pathway remain to be elucidated. Finally, the upstream mechanisms underlying the downregulation of FADS1/2 after lung injury remain unexplained. Addressing these limitations through multi‐center collaborations and advanced omics technologies will be critical for translating these mechanistic insights into effective therapies for ARDS/ALI.

The therapeutic implications of these findings are twofold: First, targeted activation of the PUFA pathway—via FADS1/2 activation or omega‐3 supplementation—effectively mitigates ferroptosis and preserves endothelial integrity. Second, modulation of lactylation or PARK7 activity may offer novel strategies to interrupt the vicious cycle of metabolic dysfunction and inflammation in ARDS. Our results underscore the potential of combined metabolic‐epigenetic interventions as a paradigm for treating acute lung injury. Future studies should explore clinical applications of this axis, including optimized PUFA formulations and small‐molecule activators of PARK7‐BMP signaling, to bridge mechanistic insights into therapeutic breakthroughs.

## 4 Conclusion

In LPS‐induced ALI, endothelial FADS1/2 downregulation reduces omega‐3 PUFAs, exacerbating ferroptosis and barrier dysfunction. PARK7 restores FADS1/2 via BMP‐BMPR‐SMAD1/5/9 signaling, while FADS1/2 deficiency triggers H3K14 lactylation to upregulate PARK7, forming a protective feedback loop. Targeting this PARK7‐FADS1/2‐lactylation axis offers therapeutic potential for ARDS.

## 5 Experimental Section

### Antibodies, Reagents, and Kits

Primary antibodies used in this study included rabbit anti‐FADS1 (A0178; Abcolonal, China), anti‐FADS2 (28034‐1‐AP; Proteintech, China), anti‐SCD1 (28678‐1‐AP; Proteintech, China), anti‐GAPDH (81640‐5‐RR; Proteintech, China), anti‐ACSL4 (22401‐1‐AP; Proteintech, China), anti‐FTH1 (RMAB48857; Bioswamp, China), anti‐GPX4 (A25009; Abcolonal, China), anti‐ZO‐1 (21773‐1‐AP; Proteintech, China), anti‐VE‐cadherin (PAB48175; Bioswamp, China), anti‐Occludin (27260‐1‐AP; Proteintech, China), anti‐Piezo1 (PAB33795; Bioswamp, China), anti‐Piezo2 (26205‐1‐AP;; Proteintech, China), anti‐PARK7 (82913‐2‐RR; Proteintech, China), anti‐MPO (MPO; GB11224; Servicebio, China), anti‐CD31 (PAB46171; Bioswamp, China), anti‐Flag (TAG60274; Bioswamp, China) anti‐BMP2 (PAB30060; Bioswamp, China), anti‐BMP4 (PAB30663; Bioswamp, China); anti‐BMPR1A (PAB33013; Bioswamp, China); anti‐BMPR1B (PAB40430; Bioswamp, China); anti‐HuR (RMAB54755; Bioswamp, China), anti‐pSMAD1/5/9 (13 820; CST, USA), anti‐SMAD5 (12167‐1‐AP; Proteintech, China), anti‐histone H3 (9715; CST, USA), anti‐Pan‐Kla (PTM‐1401RM; PTM, China), anti‐H3K9la (PTM‐1419RM; PTM, China), anti‐H3K14la (PTM‐1414RM; PTM, China), anti‐H3K18la (PAB52620; Bioswamp, China), anti‐H3K27la (A18825; Abcolonal, China), anti‐β‐actin (ZB15001‐HR, Servicebio, China), and anti‐P300 (PAB59979; Bioswamp, China) antibodies.

Main reagents used in this study included LPS (L9143; Sigma–Aldrich, USA), nigericin (HY‐127019; MCE, China), ferrostatin‐1 (T6500; TargetMol, China), SC26196 (T12856; TargetMol, China), DHA (T5369; TargetMol, China), erastin (HY‐15763; MCE, China), ML385 (T4360; TargetMol, China), ALA (T3P2904; TargetMol, China), CMLD‐2 (T36493; TargetMol, China), sodium lactate (HY‐B2227B; MCE, China), 2‐DG(HY‐13966; MCE, China), BMP agonist sb4 (HY‐12469; MCE, China), medium chain triglycerides (MCT, S25953; Yuanye, China), puromycin (HY‐K1057; MCE, China), cycloheximide (HY‐12320; MCE, China) and ROS assay reagent DCFH‐DA (HY‐D0940; MCE, China).

Additionally, the detection kits utilized in this study were as follows: Cell Counting Kit‐8 (CCK8, C0041; Beyotime, China), MDA assay kit (S0131; Beyotime, China), Reduced GSH Colorimetric Assay Kit (E‐BC‐K030; Elabscience, China), Cell Total Iron Colorimetric Assay Kit (E‐BC‐K880‐M; Elabscience, China), Lipid Peroxidation (LPO) Assay Kit (S0043; Beyotime, China), Lactic Acid Colorimetric Assay Kit (E‐BC‐K044‐M; Elabscience, China) and Lipid Assay Kit (ab242305; Abcam, UK). Enzyme‐linked immunosorbent assay kits included Human PARK7 ELISA Kit (SEL059Hu; Cloud‐Clone, China), Mouse TNF‐α ELISA Kit (MU30030; Bioswamp, China), Mouse IL‐1β ELISA Kit (MU30369; Bioswamp, China), Mouse IL‐6 ELISA Kit (MU30044; Bioswamp, China), Mouse LTB4 ELISA Kit (MU30373; Bioswamp, China), and Mouse PGE2 ELISA Kit (MU30391; Bioswamp, China).

### Patient Enrollment and Sample Collection

Plasma samples were collected from 20 non‐ARDS SCAP patients, 47 ARDS patients secondary to SCAP, and 14 non‐critical patients with other respiratory diseases (such as asthma and interstitial lung disease) serving as controls. The diagnoses of SCAP and ARDS were established according to the CURB‐65 criteria and the 2023 global new definition of ARDS, respectively. Baseline characteristics of the SCAP and ARDS patients are presented in Table S1 (Supporting Information). Peripheral blood was collected in EDTA tubes, followed by immediate centrifugation at 3000 x g for 15 min at 4 °C. The supernatant plasma was stored at −80 °C until subsequent analysis. The study protocol was approved by the Ethics Committee of Zhongshan Hospital Fudan University (B2021‐183), with written informed consent obtained from patients or their legal representatives.

### Mice Model and In Vivo Transfection

The animal experiments were approved by the Animal Care and Use Committee of Zhongshan Hospital, Fudan University (Y2021‐425). Male C57BL/6J mice (6–8 weeks old, 20‐25 g) were purchased from Shanghai Shengchang Biological Technology Co., Ltd. (Shanghai, China). The mice were housed in cages in groups, with free access to food and water and a 12‐h dark/light cycle.

For the ALI model, mice were divided into PBS and LPS groups. 5 mgkg^−1^ LPS or equal volume of PBS was administrated intratracheally after anesthetization by intraperitoneal injection of 25 mgkg^−1^ avertin (T48402; Sigma–Aldrich, USA). Mice were humanely sacrificed by overdose of avertin 3 days after intratracheal administration.

For EC‐specific overexpression of *Fads1/2*, AAV (Vec serotype; Hanbio, China) transfected with a *Fads1* or *Fads2* overexpression sequence initiated by the TIE2 promoter was administered intratracheally at a dose of 70 µl (1*10^12 VGml^−1^) per mouse. The vector maps information is presented in Figure S4A (Supporting Information). Experiments were conducted 4 weeks after AAV infection for validation or to establish ALI models.

For pulmonary transfection, we employed the Entranster‐in vivo transfection reagent (Engreen Biosystem, China) according to the manufacturer's protocol. The FADS‐UP treatment group received intratracheal administration containing 16 µL transfection reagent complexed with 4 µg each of *Fads1* and *Fads2* overexpression plasmids (total 8 µg DNA). Mock control mice were transfected with 8 µg of empty vector plasmid using the same volume of transfection reagent (16 µL). All transfection mixtures were prepared in sterile normal saline to a final volume of 50 µL, followed by incubation at room temperature for 15 min to allow complex formation before administration.

To investigate PUFA metabolism in ALI pathogenesis, mice were systematically allocated into five experimental groups with stringent control for confounding factors: 1) Mock group received intrapulmonary transfection of empty vector, followed by PBS instillation and MCT gavage (vehicle control); 2) LPS group underwent identical procedures with 5 mgkg^−1^ LPS instillation replacing PBS; 3) LPS+ALA group received daily ALA supplementation (100 mgkg^−1^ in MCT) post‐LPS challenge; 4) FADS‐UP+LPS group was pretreated with Fads1/2 overexpression plasmid transfection 48 h prior to LPS administration; 5) FADS‐UP+LPS+ALA group combined both genetic and pharmacological interventions. Crucially, MCT was employed as the solvent for ALA to eliminate potential fatty acid interference from conventional oils (e.g., corn oil), ensuring specific assessment of omega‐3 PUFA effects. Plasma, BALF, and lung tissue (split by lobe) were collected from each mouse for subsequent testing. Pulmonary edema was quantified via wet/dry weight ratios after 48 h desiccation at 55 °C. BALF was centrifuged (1500 rpm, 10 min, 4 °C) for protein concentration measurement (BCA kit, ZJ102; Epizyme, China) and cellular analysis by PBS‐resuspension counting.

### Isolation of Pulmonary Endothelial Cells

Primary lung endothelial cells were isolated from mouse lung tissue using magnetic‐activated cell sorting. Briefly, lungs were perfused with 1 mgmL^−1^ dispase II (D4693; Sigma–Aldrich, USA), digested in 1 mgmL^−1^ DNase I (10 104 159 001; Roche, USA) and 1 mgmL^−1^ collagenase IV (C4‐28; Merck, USA), and filtered to obtain single cells. For negative selection, cells were incubated with FcR blocker (BD Pharmingen, 553 142), biotinylated anti‐EpCAM (BioLegend, 118 204), and anti‐CD45 (BioLegend, 103 104), followed by streptavidin‐conjugated magnetic beads (BioLegend, 480 016); unbound CD45^−^EpCAM^−^ cells were retained. For positive selection, these cells were labeled with biotinylated anti‐CD31 (BioLegend, 102 504) and magnetic beads, and CD31⁺ endothelial cells were collected. Isolated cells were used for omics or other experiments.

### Cell Culture, Modeling, and Gene Overexpression or Knockdown

HUVECs were purchased from iCell (Shanghai, China), and MPMECs were purchased from QuiCell (Shanghai, China). Lewis Lung Cancer cell (LLC) and A549 were purchased from Servicebio (Wuhan, China). HEK‐293T cells (SCSP‐502) were purchased from the Chinese Academy of Sciences (Shanghai, China). HUVECs and MPMECs were cultured in endothelial cell medium (1001; Sciencell, USA) with 5% FBS, 1% endothelial cell growth supplement (ECGS), and 1% penicillin/streptomycin solution (P/S). LLC, A549, and HEK‐293T cells were cultured in DMEM medium (C11995500; Gibco, USA) supplemented with 10% FBS (F102; Vazyme, China) and 1% P/S (15 140 122; Gibco, USA). Cells were cultured at 37 °C in a constant temperature incubator with 5% CO2. Cultured cells from passages 3 to 8 were utilized for experimental procedures. HUVECs were injured with 1 µgml^−1^ LPS for 12 h followed by 2 µM nigericin for 4 h. MPMECs were treated with 1 µgml^−1^ LPS for 24 h to establish the injury model. To induce endothelial ferroptosis, cells were treated with 5 µM erastin for 24 h. The following optimized concentrations were used for mechanistic studies: ferroptosis inhibitor ferrostatin‐1 (2 µM), FADS2 inhibitor SC‐26196 (0.2 µM), NRF2 inhibitor ML385 (2 µM), cycloheximide (3 µM), omega‐3 fatty acid DHA (20 µM), BMP signaling agonist sb4 (1 µM), HuR inhibitor CMLD‐2 (30 µM), sodium lactate (7 mmolL^−1^), and glycolysis inhibitor 2‐DG (5 mM). All pharmacological agents were administered 12 h before injury induction to ensure proper cellular uptake and pathway modulation.

For gene overexpression, plasmids were transfected into cells using Lipofectamine 3000 (L3000001; Thermo Fisher, USA) following the manufacturer's protocol. For gene knockdown, siRNA transfection was performed using CALNP RNAi in vitro transfection reagent (DN001; D‐nano, China). All plasmids and siRNAs were custom‐synthesized by Tsingke Biotechnology (Beijing, China), with siRNA target sequences provided in Table S3 (Supporting Information).

### RNA Sequencing

Total RNA was extracted from sorted endothelial cells or HUVECs using TRIzol reagent, with quality assessed by NanoDrop 2000 and Agilent 2100/4200. PolyA‐enriched mRNA libraries were prepared and sequenced on an Illumina NovaSeq 6000 (150 bp paired‐end). After rRNA removal (silva database) and quality filtering (in‐house scripts), clean reads were aligned to the reference genome and quantified. Differential expression analysis (|log2FC|>1, q<0.05) was followed by functional enrichment (GO/KEGG/GSEA).

### Untargeted Lipidomics Analysis

Lipidomics profiling was performed on sorted pulmonary endothelial cells using methyl‐tert‐butyl ether (MTBE)‐based extraction. Briefly, thawed samples were homogenized in cold methanol/water (1:1, v/v), followed by lipid extraction with 800 µL MTBE (4 °C ultrasonication for 20 min) and centrifugation (14000 x g, 15 min, 10 °C). The organic phase was dried under nitrogen and reconstituted in 200 µL 90% isopropanol/acetonitrile for LC‐MS analysis. Chromatographic separation was achieved using a UHPLC Nexera LC‐30A system (Shimadzu) coupled to a Q Exactive Plus mass spectrometer (Thermo Scientific). Data were processed with LipidSearch 4.1 for lipid identification/quantification. Multivariate analyses included Pareto‐scaled PCA, PLS‐DA, and OPLS‐DA. Univariate significance (p < 0.05) was determined by Student's t‐test.

### Western Blot (WB)

Total protein was extracted from lung tissues and different endothelial cells using lysis buffer (P0013; Beyotime, China) supplemented with protease inhibitors (P1005; Beyotime, China). Protein concentrations were determined by BCA assay (ZJ102; Epizyme, China). Samples were separated by SDS‐PAGE (PG112; Epizyme, China) and transferred to PVDF membranes (ISEQ00010; Millipore, USA). After blocking with 5% skim milk in PBST for 1 h, membranes were incubated with primary antibodies at 4 °C overnight and HRP‐conjugated secondary antibodies at room temperature for 1 h. Protein bands were visualized using enhanced chemiluminescence (P10300; NCM, China).

### Quantitative Real‐Time Polymerase Chain Reaction (qPCR)

Total RNA was extracted using a total RNA isolation kit (RC112; Vazyme, China), and the concentration and purity were assayed with Nanodrop 2000. The extracted RNA was reverse transcribed by a 1st strand cDNA synthesis kit (R323; Vazyme, China). Quantitative PCR was performed using the universal SYBR qPCR master mix (Q312; Vazyme, China), referring to the manufacturer's instructions in the qPCR instrument (Lepgen‐96; LEPU TECHNOLOGY, China). The relative expression of the target genes was calculated using the 2^‐△△CT^ method. The primers used in this study are listed in Table S4 (Supporting Information).

### Histopathological and Immunohistochemical (IHC) Analysis

For histological evaluation, lung tissue samples were fixed in 4% paraformaldehyde (PFA) for 24 h at room temperature, followed by paraffin embedding and sectioning. Tissue sections were stained with hematoxylin and eosin (H&E) for assessment of lung injury using Smith's scoring system (0‐4 points per parameter), evaluating pulmonary edema, atelectasis, hyaline membrane formation, inflammation, and hemorrhage. Immunohistochemical staining was performed to examine the expression of FADS1, FADS2, and pan‐Kla in lung tissues. The IHC protocol included antigen retrieval, blocking, and incubation with primary antibodies overnight at 4 °C, followed by appropriate secondary antibody detection.

### Immunofluorescence (IF)

Cells were fixed with 4% PFA (15 min, room temperature), permeabilized with 0.1% Triton X‐100 (10 min), and blocked with 5% serum (1 h). After overnight incubation with primary antibodies at 4 °C, samples were stained with fluorescent secondary antibodies (1 h, room temperature) and DAPI (5 min). Paraffin tissue slices were deparaffinized, antigenically repaired, and blocked before staining. Fluorescent images were captured using a high‐resolution fluorescence scanning system (KFBIO, China) with consistent exposure settings across all samples.

### Chromatin Immunoprecipitation (ChIP), Co‐Immunoprecipitation (Co‐IP), and RNA Immunoprecipitation (RIP)

ChIP of HUVECs was performed using the SimpleChIP Enzymatic Chromatin IP Kit (9003; CST, USA) according to the manufacturer's instructions. Anti‐H3K14la and anti‐p‐SMAD1/5/9 antibodies were used to immunoprecipitate protein‐bound DNA fragments, with normal IgG serving as the negative control. The precipitated DNA was then purified and analyzed by qPCR. The primers used for ChIP are listed in Table S4 (Supporting Information). Co‐IP of HUVEC proteins was performed using the Pierce Classic IP Kit (26 146; Thermo Fisher, USA). In particular, VeriBlot for IP Detection Reagent (ab131366; Abcam, UK) was used to detect protein bands bound by primary antibodies to remove the effect of light and heavy chains. The RIP assay was performed using the RIP Assay Kit (P1801; Beyotime, China) according to the manufacturer's instructions.

### HUVEC Angiogenesis Assay

HUVECs (passage 3‐5) were seeded (1×10^4 cells/well) on Matrigel (354 234; Corning, USA) in 96‐well plates with basic medium. After 6 h incubation (37 °C, 5% CO_2_), tube networks were imaged (4× objective) and quantified by measuring total tube length (mm) and nodes per field using ImageJ (Angiogenesis Analyzer plugin).

### Transmission Electron Microscopy (TEM)

Cell samples were fixed in 2.5 % glutaraldehyde and post‐fixed in 1 % osmium tetroxide. After dehydration through a graded ethanol series, samples were penetrated, embedded in EMBed 812, and polymerized. Ultrathin sections (70 nm) were cut using an ultramicrotome, stained with uranyl acetate and lead citrate, and then imaged using a TEM (HT7800; HITACHI, Japan).

### ChIP‐Sequencing

The ChIP‐seq libraries were sequenced on the Illumina sequencing platform by Novogene Co., Ltd.(Beijing, China). ChIP‐seq libraries were constructed from end‐repaired and adapter‐ligated DNA, followed by size selection and PCR amplification. Sequencing generated 150‐bp paired‐end reads on Illumina platforms. Data were quality‐controlled (fastp), aligned to the reference genome (BWA), and peaks were called (MACS2). Motif analysis (HOMER), peak annotation (ChIPseeker), and functional enrichment (GO/KEGG) were performed.

### Dual‐Luciferase Reporter Gene Assay

Dual‐luciferase assays were performed on HEK293T cells using the Dual Luciferase Reporter Gene Assay Kit (RG029; Beyotime, China). Firefly luciferase vectors containing *PARK7* promoters of varying lengths and Renilla luciferase control vectors were purchased from Genepharma (Shanghai, China).

### Prime Editing

293T cells were co‐transfected with the pLVX‐Puro‐ZsGreen vector containing the pegRNA sequence (ACTGCCCGCAAATCGACCGGGTTTTAGAGCTAGAAATAGCAAGTTAAAATAAGGCTAGTCCGTTATCAACTTGAAAAAGTGGCACCGAGTCGGTGCGTGCGCGACCACCGGTCGATTTGCG) and the prime editor (PE) vector (Tsingke; Beijing, China). Three days post‐transfection, 2 µgmL^−1^ puromycin selection was applied for 5 days. Monoclonal colonies were isolated by limited dilution, expanded, and validated by third‐generation sequencing. Successfully mutated clones were used for subsequent experiments.

### Gas Chromatography

The method involved extracting lipids from samples, followed by methylation. ALA methyl esters were separated and quantified using an Agilent 6890 GC with a DB‐23 column and FID detector under optimized temperature programming.

### Statistical Analysis

Western blot band intensities were quantified using ImageJ software (NIH, USA). All statistical analyses were conducted with GraphPad Prism 9.0 (GraphPad Software, USA), with data presented as mean ± standard deviation (SD). Student's t‐test was employed for two‐group comparisons, while one‐way or two‐way ANOVA with appropriate post‐hoc tests was used for multi‐group comparisons. For external data validation in Figure 1Q, the valid value range was defined as [Q1 ‐ 1.5×interquartile range (IQR), Q3 + 1.5×IQR]. Any data points falling outside this range were identified as outliers and excluded from subsequent analysis. Statistical significance was defined as *P < 0.05, **P < 0.01, and ***P < 0.001.

### Ethical Approval

The protocol of plasma collection was approved by the Ethics Committee of Zhongshan Hospital Fudan University (B2021‐183) in accordance with the Declaration of Helsinki, with written informed consent obtained from patients or their legal representatives. The animal experiments were approved by the Animal Care and Use Committee of Zhongshan Hospital Fudan University (Y2021‐425).

## Conflict of Interest

The authors declare no conflict of interest.

## Author Contributions

J.X, Y.W., and W.M. contributed equally to this work. J.X., Y.W., and W.M. performed the experiments, conducted data analysis, and drafted the initial manuscript along with figure preparation. T.W., Y.L., J.S., C.Z., X.C., C.C., and Q.X. assisted in conducting experiments and revising the manuscript. X.W. and Y.S. conceived and designed the study, provided methodological guidance, and supervised the research. Y.S. acquired funding.

## Supporting information



Supporting Information

Supporting Information

Supporting Information

## Data Availability

The raw RNA‐seq data and ChIP‐seq have been uploaded to the GEO database (GSE285730, GSE306293, and GSE306296). The data that support the findings of this study are available from the corresponding author upon reasonable request.

## References

[1] N. J. Meyer , L. Gattinoni , C. S. Calfee , Lancet 2021, 398, 622. 34217425 10.1016/S0140-6736(21)00439-6 PMC8248927

[2] L. D. J. Bos , L. B. Ware , Lancet 2022, 400, 1145. 36070787 10.1016/S0140-6736(22)01485-4

[3] E. A. Gorman , C. M. O'Kane , D. F. McAuley , Lancet 2022, 400, 1157. 36070788 10.1016/S0140-6736(22)01439-8

[4] M. I. Townsley , Compr Physiol 2012, 2, 675. 23606929 10.1002/cphy.c100081 PMC3630377

[5] J. N. Gonzales , R. Lucas , A. D. Verin , Austin J Vasc Med 2015, 2, 1009. 26973981 PMC4786180

[6] Y. Su , R. Lucas , D. J. R. Fulton , A. D. Verin , Chin Med J Pulm Crit Care Med 2024, 2, 80. 39006829 10.1016/j.pccm.2024.04.002 PMC11242916

[7] T. Zhou , K. Long , J. Chen , L. Zhi , X. Zhou , P. Gao , Front Physiol 2024, 15, 1326392. 38774649 10.3389/fphys.2024.1326392 PMC11107300

[8] G. Liu , R. Summer , Annu. Rev. Physiol. 2019, 81, 403. 30485759 10.1146/annurev-physiol-020518-114640 PMC6853603

[9] R. Batra , W. Whalen , S. Alvarez‐Mulett , L. G. Gomez‐Escobar , K. L. Hoffman , W. Simmons , J. Harrington , K. Chetnik , M. Buyukozkan , E. Benedetti , M. E. Choi , K. Suhre , E. Schenck , A. M. K. Choi , F. Schmidt , S. J. Cho , J. Krumsiek , PLoS Pathog. 2022, 18, 1010819. 10.1371/journal.ppat.1010819 PMC9484674 36121875

[10] S. Y. Zhang , D. Shao , H. Liu , J. Feng , B. Feng , X. Song , Q. Zhao , M. Chu , C. Jiang , W. Huang , X. Wang , Redox Biol. 2017, 13, 459. 28715731 10.1016/j.redox.2017.07.001 PMC5512213

[11] N. Alipanah‐Lechner , L. Neyton , E. Mick , A. Willmore , A. Leligdowicz , K. Contrepois , A. Jauregui , H. Zhuo , C. Hendrickson , A. Gomez , P. Sinha , K. N. Kangelaris , K. D. Liu , M. A. Matthay , A. J. Rogers , C. S. Calfee , Am J Physiol Lung Cell Mol Physiol 2023, 324, L297. 36648136 10.1152/ajplung.00278.2022 PMC9988532

[12] H. Poudyal , S. K. Panchal , V. Diwan , L. Brown , Prog Lipid Res 2011, 50, 372. 21762726 10.1016/j.plipres.2011.06.003

[13] H. Sunaga , H. Matsui , M. Ueno , T. Maeno , T. Iso , M. R. Syamsunarno , S. Anjo , T. Matsuzaka , H. Shimano , T. Yokoyama , M. Kurabayashi , Nat. Commun. 2013, 4, 2563. 24113622 10.1038/ncomms3563

[14] Y. Xuan , H. Wang , M. M. Yung , F. Chen , W. S. Chan , Y. S. Chan , S. K. Tsui , H. Y. Ngan , K. K. Chan , D. W. Chan , Theranostics 2022, 12, 3534. 35547771 10.7150/thno.70194 PMC9065188

[15] Y. Jiang , C. Mao , R. Yang , B. Yan , Y. Shi , X. Liu , W. Lai , Y. Liu , X. Wang , D. Xiao , H. Zhou , Y. Cheng , F. Yu , Y. Cao , S. Liu , Q. Yan , Y. Tao , Theranostics 2017, 7, 3293. 28900510 10.7150/thno.19988 PMC5595132

[16] R. Liu , S. Qiao , W. Shen , Y. Liu , Y. Lu , H. Liangyu , Z. Guo , J. Gong , G. Shui , Y. Li , W. Zhu , J Crohns Colitis 2020, 14, 1581. 32365195 10.1093/ecco-jcc/jjaa086

[17] X. Jiang , B. R. Stockwell , M. Conrad , Nat. Rev. Mol. Cell Biol. 2021, 22, 266. 33495651 10.1038/s41580-020-00324-8 PMC8142022

[18] Y. Wen , Y. Liu , W. Liu , W. Liu , J. Dong , Q. Liu , Z. Yu , H. Ren , H. Hao , Inflamm Res 2024, 73, 1615. 39152299 10.1007/s00011-024-01919-z

[19] Y. Wang , Z. Zhao , Z. Xiao , J Inflamm Res 2023, 16, 4073. 37727372 10.2147/JIR.S420676 PMC10506607

[20] J. Y. Lee , W. K. Kim , K. H. Bae , S. C. Lee , E. W. Lee , Biology (Basel) 2021, 10, 184.33801564

[21] J. Y. Lee , M. Nam , H. Y. Son , K. Hyun , S. Y. Jang , J. W. Kim , M. W. Kim , Y. Jung , E. Jang , S. J. Yoon , J. Kim , J. Kim , J. Seo , J. K. Min , K. J. Oh , B. S. Han , W. K. Kim , K. H. Bae , J. Song , J. Kim , Y. M. Huh , G. S. Hwang , E. W. Lee , S. C. Lee , Proc Natl Acad Sci U S A 2020, 117, 32433. 33288688 10.1073/pnas.2006828117 PMC7768719

[22] R. Wang , J. Zhang , H. Ren , S. Qi , L. Xie , H. Xie , Z. Shang , C. Liu , Cell. Mol. Life Sci. 2024, 81, 85. 38345762 10.1007/s00018-024-05145-y PMC10861707

[23] M. Zhu , J. Yu , Immun. Inflamm. Dis. 2024, 12, 70059. 10.1002/iid3.70059 PMC11647999 39679976

[24] L. Zhang , J. Wang , J. Wang , B. Yang , Q. He , Q. Weng , Front. Immunol. 2020, 11, 994. 32612601 10.3389/fimmu.2020.00994 PMC7308417

[25] M. Neves , M. Grãos , S. I. Anjo , B. Manadas , Redox Biol. 2022, 51, 102283. 35303520 10.1016/j.redox.2022.102283 PMC8928136

[26] H. Amatullah , T. Maron‐Gutierrez , Y. Shan , S. Gupta , J. N. Tsoporis , A. K. Varkouhi , A. P. Teixeira Monteiro , X. He , J. Yin , J. C. Marshall , P. R. M. Rocco , H. Zhang , W. M. Kuebler , C. C. Dos Santos , Redox Biol. 2021, 38, 101796. 33246293 10.1016/j.redox.2020.101796 PMC7695876

[27] X. W. Liu , T. Ma , Q. Cai , L. Wang , H. W. Song , Z. Liu , J. Intensive Care Med. 2019, 34, 662. 28506137 10.1177/0885066617709689

[28] J. Xu , T. Wei , Y. Wang , C. Zhang , X. Chen , Y. Li , J. Song , W. Mao , Q. Xu , X. Wu , Y. Song , Int. Immunopharmacol. 2025, 164, 115386. 40834532 10.1016/j.intimp.2025.115386

[29] J. Cao , X. Chen , L. Jiang , B. Lu , M. Yuan , D. Zhu , H. Zhu , Q. He , B. Yang , M. Ying , Nat. Commun. 2020, 11, 1251. 32144268 10.1038/s41467-020-15109-y PMC7060199

[30] S. Chen , H. Xia , L. Sheng , Dig. Liver Dis. 2023, 55, 967. 36586770 10.1016/j.dld.2022.12.005

[31] J. Pan , W. Xiong , A. Zhang , H. Zhang , H. Lin , L. Gao , J. Ke , S. Huang , J. Zhang , J. Gu , A. C. Y. Chang , C. Wang , Adv. Sci. (Weinh) 2023, 10, 2206007. 36967569 10.1002/advs.202206007 PMC10214246

[32] L. Chen , L. Huang , Y. Gu , W. Cang , P. Sun , Y. Xiang , Int. J. Mol. Sci. 2022, 23, 11943. 36233246 10.3390/ijms231911943 PMC9569569

[33] F. Jing , J. Zhang , H. Zhang , T. Li , Biol. Rev. Camb. Philos. Soc. 2025, 100, 172. 39279350 10.1111/brv.13135

[34] S. Wang , H. Zheng , J. Zhao , J. Xie , Int. J. Mol. Med. 2025, 55, 1. 40052587 10.3892/ijmm.2025.5512 PMC11913435

[35] F. Gong , X. Zheng , W. Xu , R. Xie , W. Liu , L. Pei , M. Zhong , W. Shi , H. Qu , E. Mao , Z. Yang , R. Li , E. Chen , Y. Chen , Med. Comm. 2020, 6, 70049. 10.1002/mco2.70049 PMC11733091 39822760

[36] Z. Lu , P. Fang , S. Li , D. Xia , J. Zhang , X. Wu , J. Pan , H. Cai , L. Fu , G. Sun , Q. You , Adv. Sci. (Weinh) 2025, 12, 2407064. 39721014 10.1002/advs.202407064 PMC11831459

[37] J. Xu , T. Pan , X. Qi , R. Tan , X. Wang , Z. Liu , Z. Tao , H. Qu , Y. Zhang , H. Chen , Y. Wang , J. Zhang , J. Wang , J. Liu , Respir. Res. 2020, 21, 99. 32354336 10.1186/s12931-020-01364-6 PMC7193408

[38] C. F. Schaefer , K. Anthony , S. Krupa , J. Buchoff , M. Day , T. Hannay , K. H. Buetow , Nucleic Acids Res. 2009, 37, D674. 18832364 10.1093/nar/gkn653 PMC2686461

[39] J. M. Finan , T. L. Sutton , D. A. Dixon , J. R. Brody , Cancer Res. 2023, 83, 3507. 37683260 10.1158/0008-5472.CAN-23-0972 PMC13449165

[40] S. D. Harmon , T. L. Kaduce , T. D. Manuel , A. A. Spector , Lipids 2003, 38, 469. 12848296 10.1007/s11745-003-1086-9

[41] M. G. Obukowicz , A. Raz , P. D. Pyla , J. G. Rico , J. M. Wendling , P. Needleman , Biochem. Pharmacol. 1998, 55, 1045. 9605428 10.1016/s0006-2952(97)00665-5

[42] D. Yamane , Y. Hayashi , M. Matsumoto , H. Nakanishi , H. Imagawa , M. Kohara , S. M. Lemon , I. Ichi , Cell Chem. Biol. 2022, 29, 799.e4. 34520742 10.1016/j.chembiol.2021.07.022 PMC8913804

[43] X. Wang , A. Xiao , Y. Yang , Y. Zhao , C. C. Wang , Y. Wang , J. Han , Z. Wang , M. Wen , Mol. Nutr. Food Res. 2022, 66, 2200275. 10.1002/mnfr.202200275 36099650

[44] Y. Yang , X. Wang , L. Chen , S. Wang , J. Han , Z. Wang , M. Wen , Mar Drugs 2023, 21, 464. 37755077 10.3390/md21090464 PMC10533149

[45] P. Fang , S. Cheng , Y. Lai , X. Ma , K. Lu , J. Lu , G. Li , E. Yang , N. Yang , W. Gao , R. Jiang , Eur. J. Pharm. Sci. 2024, 203, 106923. 39368783 10.1016/j.ejps.2024.106923

[46] C. López‐Vicario , A. González‐Périz , B. Rius , E. Morán‐Salvador , V. García‐Alonso , J. J. Lozano , R. Bataller , M. Cofán , J. X. Kang , V. Arroyo , J. Clària , E. Titos , Gut 2014, 63, 344. 23492103 10.1136/gutjnl-2012-303179

[47] P. Cotogni , A. Trombetta , G. Muzio , M. Maggiora , R. A. Canuto , Biomed Res. Int. 2015, 2015, 642520. 26301250 10.1155/2015/642520 PMC4537738

[48] E. Olivo , M. La Chimia , J. Ceramella , A. Catalano , F. Chiaradonna , M. S. Sinicropi , G. Cuda , D. Iacopetta , D. Scumaci , Cells. 2022, 11, 1432. 35563738 10.3390/cells11091432 PMC9103122

[49] E. C. Kirkpatrick , S. Handler , M. Liegl , A. Y. Pan , G. G. Konduri , T. M. Gudausky , A. J. Afolayan , Pulm. Circ. 2025, 15, 70068. 10.1002/pul2.70068 PMC11964942 40182212

[50] Q. Shi , H. Liu , H. Wang , L. Tang , Q. Di , D. Wang , Respir. Res. 2025, 26, 142. 40223052 10.1186/s12931-025-03215-8 PMC11995649

[51] A. Sankar , F. Mohammad , A. K. Sundaramurthy , H. Wang , M. Lerdrup , T. Tatar , K. Helin , Nat. Genet. 2022, 54, 754. 35668298 10.1038/s41588-022-01091-2

[52] G. L. Brien , R. B. Bressan , C. Monger , D. Gannon , E. Lagan , A. M. Doherty , E. Healy , H. Neikes , D. J. Fitzpatrick , O. Deevy , V. Grant , M. A. Marqués‐Torrejón , N. Alfazema , S. M. Pollard , A. P. Bracken , Nat. Genet. 2021, 53, 1221. 34294917 10.1038/s41588-021-00897-w

