# Supplementary material for: Metabolic Interplay in Acute Lung Injury: PARK7 Integrates FADS1/2‐Dependent PUFA Metabolism and H3K14 Lactylation to Attenuate Endothelial Ferroptosis and Dysfunction
Source: Adv Sci (Weinh). 2025 Sep 30;12(46):e08725. doi: 10.1002/advs.202508725 (PMC12697827; doi:10.1002/advs.202508725)
Supplement: Supplementary file 1 — Supporting Information [file ADVS-12-e08725-s002.docx]

**Table S1 Baseline characteristics in** **SCAP patients and ARDS patients**

| **Variables** | **SCAP (n=20)** | **ARDS (n=47)** | ***P*-value** |
| --- | --- | --- | --- |
| **Age (yrs)** | 73.00 (61.50–77.25) | 72.00 (58.00–79.50) | 0.864 |
| **Sex (% men)** | 12 (60%) | 38 (81%) | 0.073 |
| **Comorbidity** |  |  |  |
| Malignancy | 4 (20%) | 13 (28%) | 0.760 |
| Hypertension | 7 (35%) | 28 (60%) | 0.065 |
| Diabetes | 7 (35%) | 7 (15%) | 0.064 |
| Cardio/Cerebrovascular disease | 5 (25%) | 14 (30%) | 0.691 |
| Arrhythmia | 5 (25%) | 2 (4%) | 0.021 |
| Other respiratory disease | 1 (5%) | 4 (9%) | 1.000 |
| Non-vascular neurological disorder | 2 (10%) | 5 (11%) | 1.000 |
| **APACHE II score** | 13.05 ± 5.81 | 19.49 ± 6.15 | <0.001 |
| **SOFA score** | 3.00 (2.00–4.00) | 5.00 (3.00–7.00) | <0.001 |
| **PSI score** | 93.00 ± 24.99 | 107.81 ± 27.55 | 0.038 |
| **PaO2/FiO2 ratio** | 262.40 ± 87.34 | 155.47 ± 50.19 | <0.001 |
| **Laboratory tests** |  |  |  |
| Hemoglobin (g/L) | 96.45 ± 25.08 | 105.85 ± 25.80 | 0.172 |
| Platelet (10^9/L) | 206.50 (139.75–293.50) | 162.00 (92.50–237.00) | 0.164 |
| WBC (10^9/L) | 8.48 (7.00–9.68) | 11.45 (8.67–15.02) | 0.007 |
| N% | 78.20 (72.80–86.95) | 87.90 (84.30–93.25) | 0.001 |
| L% | 11.20 (7.62–16.88) | 5.90 (2.85–8.20) | <0.001 |
| CRP (mg/L) | 57.70 (29.25–128.88) | 90.00 (33.05–146.60) | 0.435 |
| PCT (ng/mL) | 0.47 (0.12–1.03) | 0.45 (0.16–1.04) | 0.779 |
| Fibrinogen (g/L) | 4.45 ± 2.27 | 4.61 ± 2.12 | 0.792 |
| D-dimer (mg/L) | 1.54 (1.28–3.72) | 4.95 (2.63–9.44) | 0.008 |
| Total bilirubin (µmol/L) | 9.75 (6.80–14.45) | 11.20 (8.35–15.20) | 0.311 |
| Albumin (g/L) | 33.40 ± 5.50 | 33.60 ± 5.72 | 0.896 |
| Globulin (g/L) | 27.00 (23.75–34.00) | 25.00 (22.00–29.50) | 0.219 |
| LDH (U/L) | 233.50 (206.25–273.50) | 323.00 (257.00–544.50) | <0.001 |
| BUN (mmol/L) | 6.45 (5.28–8.92) | 9.30 (6.60–15.80) | 0.025 |
| Creatinine (µmol/L) | 65.00 (43.00–71.00) | 66.00 (50.00–148.00) | 0.233 |
| **Death (in-hospital)** | 4 (20%) | 11 (23%) | 1.000 |
| **Invasive ventilation** | 10 (50%) | 35 (74%) | 0.051 |
| **Length of stay (days)** | 9.50 (7.75–16.50) | 24.00 (17.50–39.00) | <0.001 |

Binary variables: n (%) and Pearson Chi-square; Fisher's exact when any expected count <5.

Continuous variables: Shapiro–Wilk normality test per group; if both p≥0.05 use Welch t-test and report mean ± SD; otherwise use Mann–Whitney U and report median (IQR).

Abbreviations: SCAP, severe community-acquired pneumonia; ARDS, acute respiratory distress syndrome; APACHE II, acute physiology and chronic health evaluation II; SOFA, sequential organ failure assessment; PSI, pneumonia severity index; WBC, white blood cell count; N%, neutrophil percentage; L%, lymphocyte percentage; CRP, C-reactive protein; PCT, procalcitonin; LDH, lactate dehydrogenase; BUN, blood urea nitrogen; SD, standard deviation; IQR, interquartile range.

**Table S2 Binding sites of transcription factors and gene promoters predicted by JASPAR (https://jaspar.elixir.no/)**

| **Transcription factor** | **Target gene** | **Score** | **Relative score** | **Sequence ID** | **Start** | **End** | **Strand** | **Predicted sequence** |
| --- | --- | --- | --- | --- | --- | --- | --- | --- |
| SMAD5 | *FADS1* | 5.507673 | 0.821339 | NC_000011.10:c61819003-61817004 | 820 | 829 | + | AATCTAGCCA |
|  |  | 5.006398 | 0.8135859 |  | 820 | 829 | - | TGGCTAGATT |
|  |  | 4.93732 | 0.8125175 |  | 375 | 384 | - | TGGCTAGAAT |
|  |  | 4.3573627 | 0.8035474 |  | 375 | 384 | + | ATTCTAGCCA |
|  |  | 4.265565 | 0.80212754 |  | 1702 | 1711 | + | CGTCTGGAGA |
|  | *FADS2* | 9.342868 | 0.8806575 | NC_000011.10:61814203-61816202 | 1906 | 1915 | - | CGTCTGGACC |
|  |  | 7.3984566 | 0.85058355 |  | 1938 | 1947 | + | CATCGAGACA |
|  |  | 7.297299 | 0.84901893 |  | 1487 | 1496 | + | GGTCTAGAAA |
|  |  | 6.737076 | 0.8403541 |  | 1487 | 1496 | - | TTTCTAGACC |
|  |  | 6.579831 | 0.837922 |  | 1906 | 1915 | + | GGTCCAGACG |
|  |  | 4.3777843 | 0.8038632 |  | 1188 | 1197 | - | TGACGCGACC |
| NRF2  (NFE2L2) | *BMP2* | 9.942441 | 0.84882814 | NC_000020.11:6765686-6767685 | 963 | 973 | + | GTGACTTGGCC |
|  |  | 9.21707 | 0.83079857 |  | 1696 | 1706 | + | CTGACCCTGCA |
|  |  | 8.511445 | 0.8132598 |  | 976 | 986 | + | GTGTCACAGCC |
|  | *BMP4* | 9.392886 | 0.83516866 | NC_000014.9:c53958891-53956892 | 803 | 813 | - | AAGACTGAGCA |
|  |  | 8.625281 | 0.81608933 |  | 1285 | 1295 | - | CCGACACAGCA |

**Table S3 siRNA sequences used in this study**

| ***PARK7*** | Sense (5’ to 3’) | Antisense (5’ to 3’) |
| --- | --- | --- |
| siRNA1 | GGUUCUACCAGGAGGUAAU(dT)(dT) | AUUACCUCCUGGUAGAACC(dT)(dT) |
| ***FADS1*** | Sense (5’ to 3’) | Antisense (5’ to 3’) |
| siRNA1 | GGCUAGUGAUCGACCGUAA(dT)(dT) | UUACGGUCGAUCACUAGCC(dT)(dT) |
| siRNA2 | GGAAGAUCCUCUCUGUGGA(dT)(dT) | UCCACAGAGAGGAUCUUCC(dT)(dT) |
| siRNA3 | GCAUAGAGUACCAGUCCAA(dT)(dT) | UUGGACUGGUACUCUAUGC(dT)(dT) |
| ***FADS2*** | Sense (5’ to 3’) | Antisense (5’ to 3’) |
| siRNA1 | CCGGCACAACUUACACAAGAU(dT)(dT) | AUCUUGUGUAAGUUGUGCCGG(dT)(dT) |
| siRNA2 | ACAUCGUCAUGGAGAUUGA(dT)(dT) | UCAAUCUCCAUGACGAUGU(dT)(dT) |
| siRNA3 | CCAUGAUCGUCCAUAAGAA(dT)(dT) | UUCUUAUGGACGAUCAUGG(dT)(dT) |
| ***LDHA*** | Sense (5’ to 3’) | Antisense (5’ to 3’) |
| siRNA1 | GGAGAAAGCCGUCUUAAUU(dT)(dT) | AAUUAAGACGGCUUUCUCC(dT)(dT) |
| siRNA2 | GACUGAUAAAGAUAAGGAA(dT)(dT) | UUCCUUAUCUUUAUCAGUC(dT)(dT) |
| siRNA3 | GAUUAAGGGUCUUUACGGA(dT)(dT) | UCCGUAAAGACCCUUAAUC(dT)(dT) |

**Table S4 Primers used in this study**

| **Genes** | **Forward** **(5’ to 3’)** | **Reverse (5’ to 3’)** |
| --- | --- | --- |
| *Gapdh* (mouse) | GGTCGGTGTGAACGGATTTG | ATGAAGGGGTCGTTGATGGC |
| *Park7* (mouse) | AGCCGGGATCAAAGTCACTG | GGTCCCTGCGTTTTTGCATC |
| *Fads1* (mouse) | ACTTCTTCCTCATCGGACCC | ATGCGGGCATAGAAGCTGAG |
| *Fads2* (mouse) | GGCCACTTAAAGGGTGCCTC | GGCTCTTTATGTCCGGGTCC |
| *Scd1* (mouse) | GTGATGTTCCAGAGGAGGTACT | AGAAGGTGCTAACGAACAGGC |
| *Actb* (mouse) | ATCGCTGCGCTGGTCG | AGTCCTTCTGACCCATTCCC |
| *GAPDH* (human) | AAAGCCTGCCGGTGACTAAC | GCCCAATACGACCAAATCAGA |
| *PARK7* (human) | GAGGCGAGCTGGGATTAAGG | TCTTCAAGGCTGGCATCAGG |
| *LDHA* (human) | CATGGAGATTCCAGTGTGCCT | ACCACCTGCTTGTGAACCTC |
| *ACTB* (human) | AACCGCGAGAAGATGACCCA | GATAGCACAGCCTGGATAGCAA |
| *FADS1* (human) | GTGGCTAGTGATCGACCGTA | CACAAAGGGATCCGTGGCA |
| *FADS2* (human) | ACCTTCAGCTGGGAGGAGATT | CGCGGAAGGCATCCGTTG |
| *SCD1* (human) | TTCCCGACGTGGCTTTTTCT | AGCCAGGTTTGTAGTACCTCC |
| *TIE2* (human) | GGGGAGATGTGTGATCGCTT | GGGTCATCCTCTGTATGCCTTC |
| *VEGFR2* (human) | GAGGGGAACTGAAGACAGGC | GGCCAAGAGGCTTACCTAGC |
| *BMP2* (human) | AGCTTCCACCATGAAGAATCTTTG | TCCTCCGTGGGGATAGAACT |
| *BMP4* (human) | TCTTGAGTATCCTGAGCGCC | GGATGTTCTCCAGATGTTCTTCG |
| *BMPR1A* (human) | CCCCTGTTGTCATAGGTCCG | ACGCAACAGCAGATGACCTT |
| *BMPR1B* (human) | ATGACTCTGGGTTGCCTGTG | GAGGAATGGGAGTGTCCCGA |
| *preFADS1* (human) | AGATTCACGTTGGCAGGTCC | CAGACGAAACAGGCACCAAC |
| *preFADS2* (human) | TCCTCTCTCCTCAGTACGGC | GGTCCCAGAGTGATTTCGGG |
| *preBMP2* (human) | TTCGGCCTGAAACAGAGACC | GCTCCATGCCTCACCTTCAT |
| *preBMP4* (human) | ACCTTTTGCCTCGGTCACAG | CAGGTTCACTGCAACCGTTC |
| *preBMPR1A* (human) | GCCTTCCCAGCGACCTTATT | GGCCGGATATCCCCACTCTC |
| *preBMPR1B* (human) | GTTACGCCCCTCATTCCCAA | AATGAGGCGCCATCACCATC |
| *PARK7* *-1000~TSS* (for ChIP) | AACAGACCAGAGGCGAAACG | GGGTTCCTTAGTTCGCGCT |
| *PARK7* *TSS~+1000* (for ChIP) | TTGGATTTGACTGACCGCCA | GAGGGTTTACCGTCCACGC |
| *FADS1* promoter (for ChIP) | AGGAACAGCGGTTCTAGTGC | GCACGGAAAAACCTCAACCC |
| *FADS2* promoter (for ChIP) | CCGAGTGGTCACTCTGGTTG | AGGTTCCCATAACGTCGCAG |
| *H3-3A* (human) | ATGCTGGTAGGTAAGTAAGGAGG | AAAGCAAAAAGTTTTCCTGTTATCC |

TSS, transcription start site; ChIP, chromatin immunoprecipitation.


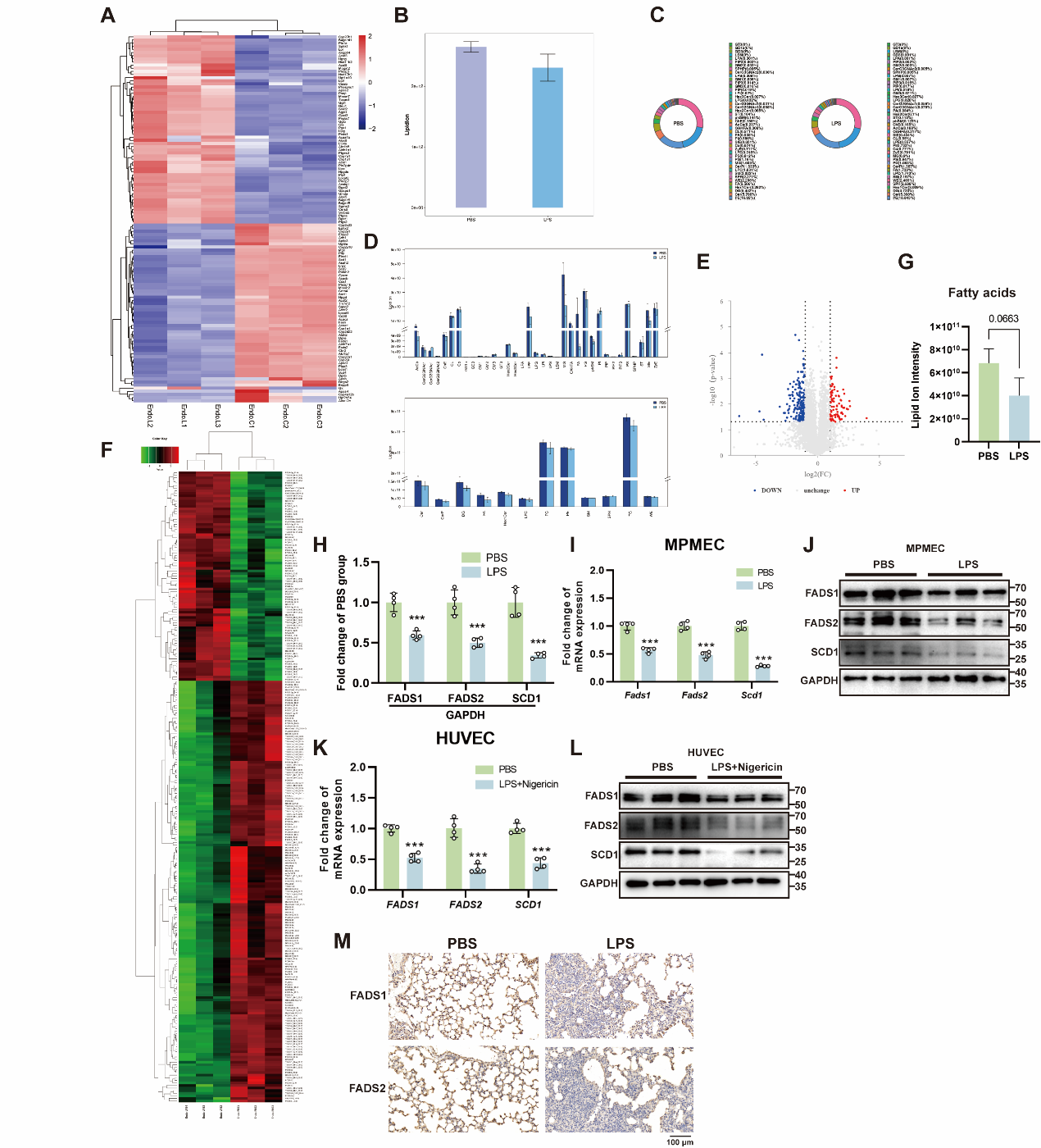


**Figure S1 Supplementary data and validation of RNA sequencing (RNA-seq) and lipidomics in sorted pulmonary endothelial cells (ECs).** (**A**) Heatmap of differentially expressed lipid metabolism-associated genes from RNA-seq in sorted pulmonary ECs. (**B**) Total lipid content of sorted pulmonary ECs identified by lipidomics (n = 3). (**C-D**) Changes in lipid composition (C) and content of various lipid classes (D) in sorted pulmonary ECs after PBS and LPS administration (n = 3). (**E-F**) Volcano plot (E) and heatmap (F) of differential lipid species identified by lipidomics. (**G**) Changes in total fatty acid content in pulmonary ECs (n = 3). (**H**) Quantitative statistics of lung tissue Western blot (WB) corresponding to **Figure 1K** (n = 4). (**I-J**) Real-time quantitative PCR (qPCR) (I, n = 4) and WB (J) analysis of desaturase expression changes in mouse pulmonary microvascular endothelial cells (MPMECs). (**K-L**) qPCR (I, n = 4) and WB (J) analysis of desaturase expression changes in human umbilical vein endothelial cells (HUVECs). (**M**) Immunohistochemical (IHC) analysis of FADS1 and FADS2 in mouse lung tissues.

*, P < 0.05; **, P < 0.01; ***, P < 0.001.


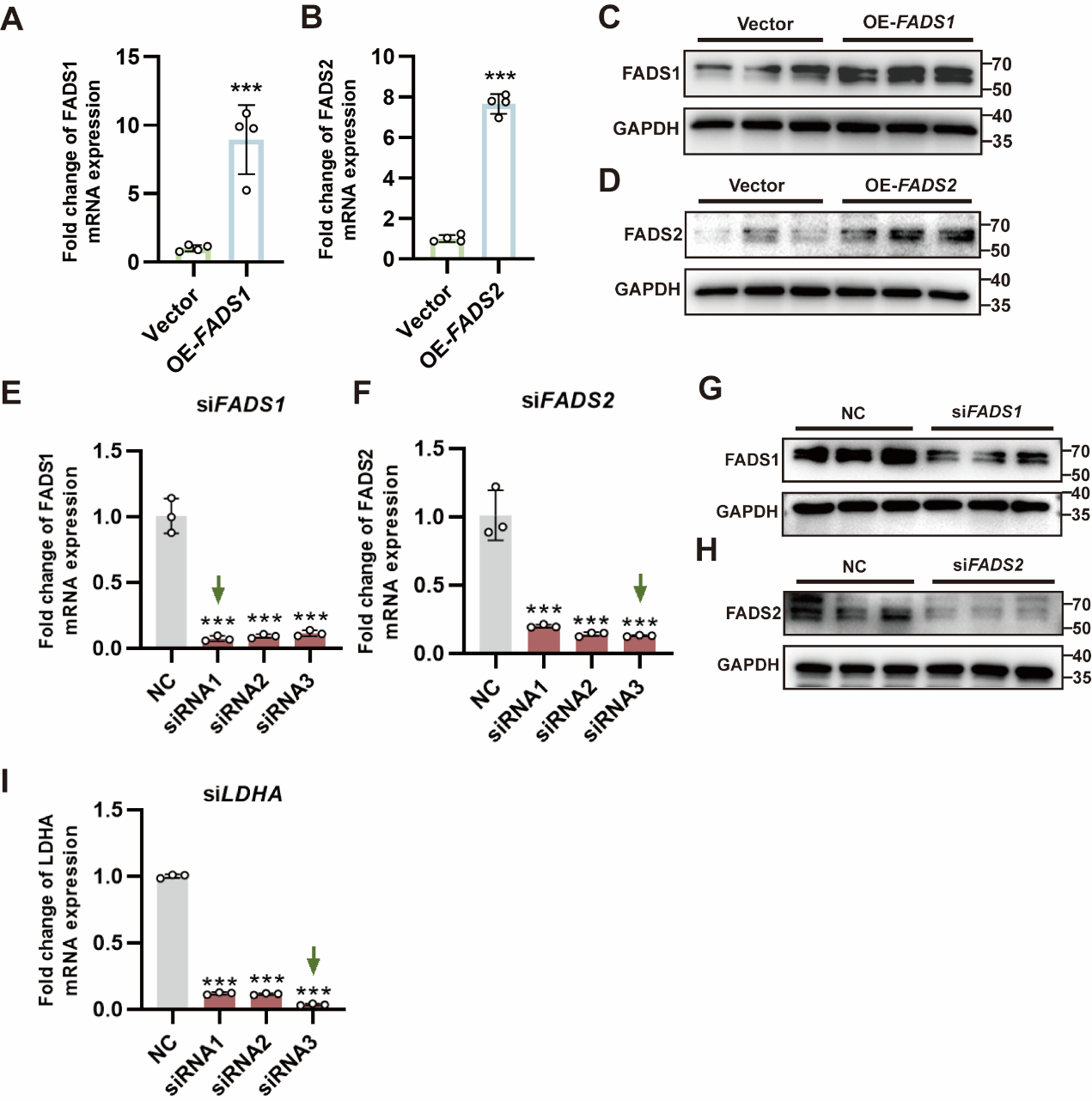


**Figure S2** **Validation of overexpression and knockout effects for selected genes in human umbilical vein endothelial cells (HUVECs) *in vitro* in this study**. (**A-B**) Real-time quantitative PCR (qPCR) validation of *FADS1* (A) and *FADS2* (B) overexpression (n = 4). (**C-D**) Western blot (WB) validation of FADS1 (C) and FADS2 (D) overexpression. (**E-F**) qPCR validation of *FADS1* (E) and *FADS2* (F) knockdown (n = 3). The green arrow indicates the siRNA used in subsequent experiments. (**G-H**) WB validation of FADS1 (G) and FADS2 (H) knockdown. (**I**) qPCR validation of *LDHA* knockdown. The green arrow indicates the siRNA used in subsequent experiments (n = 3).

OE, overexpression; NC, negative control. *, *P* < 0.05; **, *P* < 0.01; ***, *P* < 0.001.


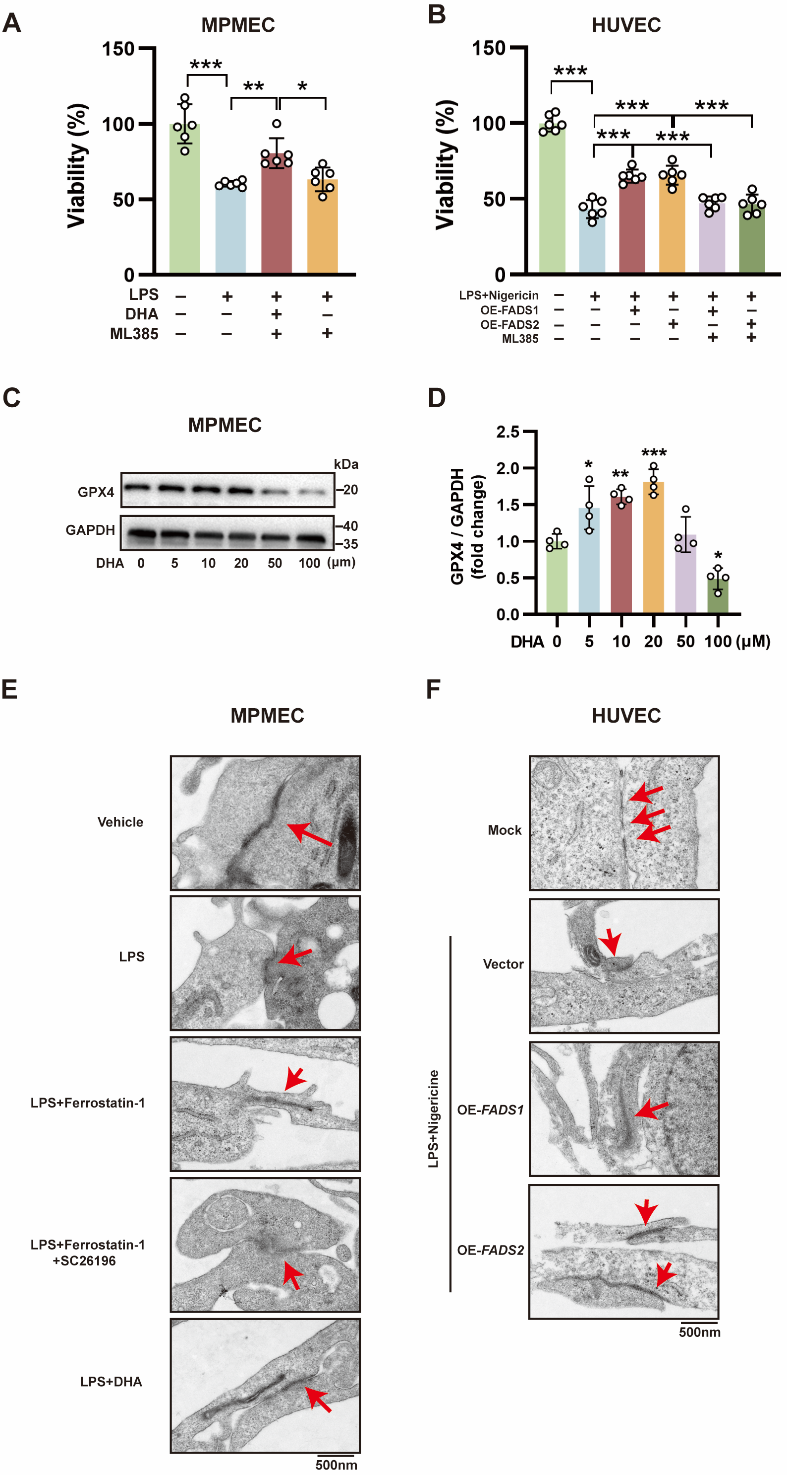


**Figure S3 Supplementary data on regulating the PUFA pathway to alleviate endothelial cell ferroptosis and tight junction dysfunction.** (**A**) CCK8 assay of mouse pulmonary microvascular endothelial cells (MPMECs) viability after lipopolysaccharide (LPS) injury, docosahexaenoic acid (DHA) treatment and ML385 administration (n = 6). (**B**) CCK8 assay of human umbilical vein endothelial cells (HUVECs) viability after injury, *FADS1/2* overexpression and ML385 administration (n = 6). (**C-D**) Western blot detection and quantification of GPX4 expression changes in response to varying DHA concentrations in MPMECs (n = 4). (**E**) Observation of tight junction morphology under TEM in MPMECs treated with LPS, Ferrostatin-1, SC-26196, or DHA. (**F**) Observation of tight junction morphology in HUVECs under TEM in following treatment with LPS, nigericin, and transfection with *FADS1/2* overexpression plasmids.

OE, overexpression; Vehicle, addition of corresponding solvents to the culture system; Vector, transfection with vector plasmids; Mock, transfection with vector plasmids and addition of corresponding solvents; TEM, transmission electron microscopy. *, *P* < 0.05; **, *P* < 0.01; ***, *P* < 0.001.


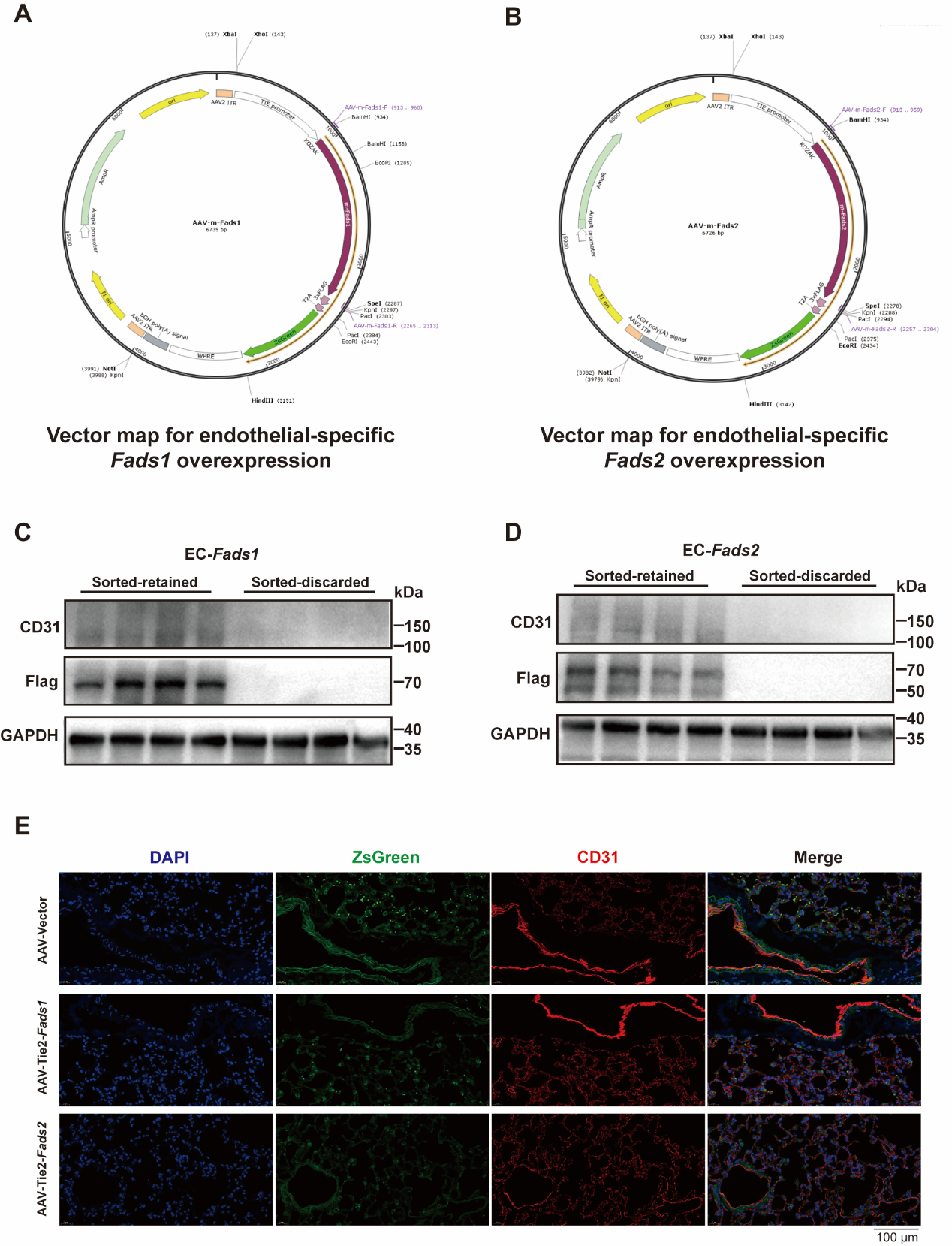


**Figure S4 Supplementary data on endothelial cell-specific overexpression via AAV.** (**A-B**) Maps of *pHBAAV-TIE2-3flag-T2A-ZsGreen* vectors for endothelial cell-specific overexpression of *Fads1* (A) and *Fads2* (B), respectively. (**C-D**) Western blot validation of CD31 and Flag expression in sorted retained cells versus sorted discarded cells in EC-*Fads1* (C) and EC-*Fads2* (D) groups. (**E**) Immunofluorescence validation of ZsGreen localization following AAV infection.

AAV, adeno-associated virus.


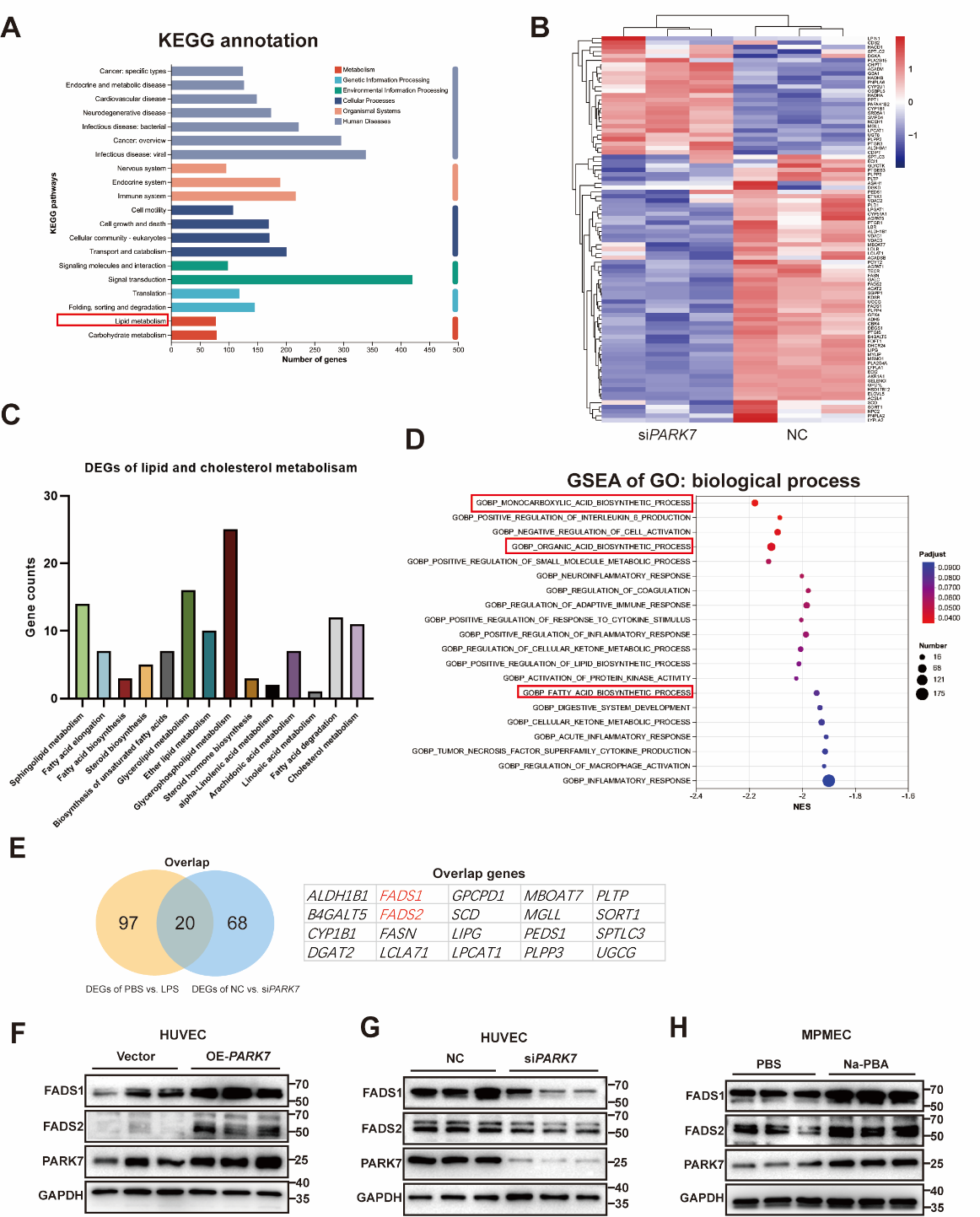


**Figure S5** **Supplementary data of RNA sequencing (RNA-seq) in human umbilical vein endothelial cells (HUVECs) and validation.** (**A**) KEGG pathway annotation analysis of differentially expressed genes (DEGs) from RNA-seq in PARK7-knockdown HUVECs. The red box highlights lipid metabolism. (**B**) Heatmap of expression levels of lipid metabolism-related DEGs. (**C**) Number of lipid metabolism-related DEGs involved in different metabolic pathways. (**D**) Gene set enrichment analysis (GSEA) of pathways in the GO biological process category. The red box denotes fatty acid-related pathways. (**E**) Venn diagram showing overlapping lipid metabolism-related DEGs between PARK7-knockout HUVECs and sorted lung endothelial cells post-lung injury. The table displays all overlapping genes (annotated with human gene symbols). (**F-G**) WB analysis of alterations in PARK7, FADS1, and FADS2 expression upon *PARK7* overexpression (F) or knockdown (G) in HUVECs. (**H**) WB analysis of alterations in PARK7, FADS1, and FADS2 expression in mouse pulmonary microvascular endothelial cells (MPMECs) following sodium phenylbutyrate (Na-PBA) treatment.

OE, overexpression; Vector, transfection with vector plasmids; NC, negative control.


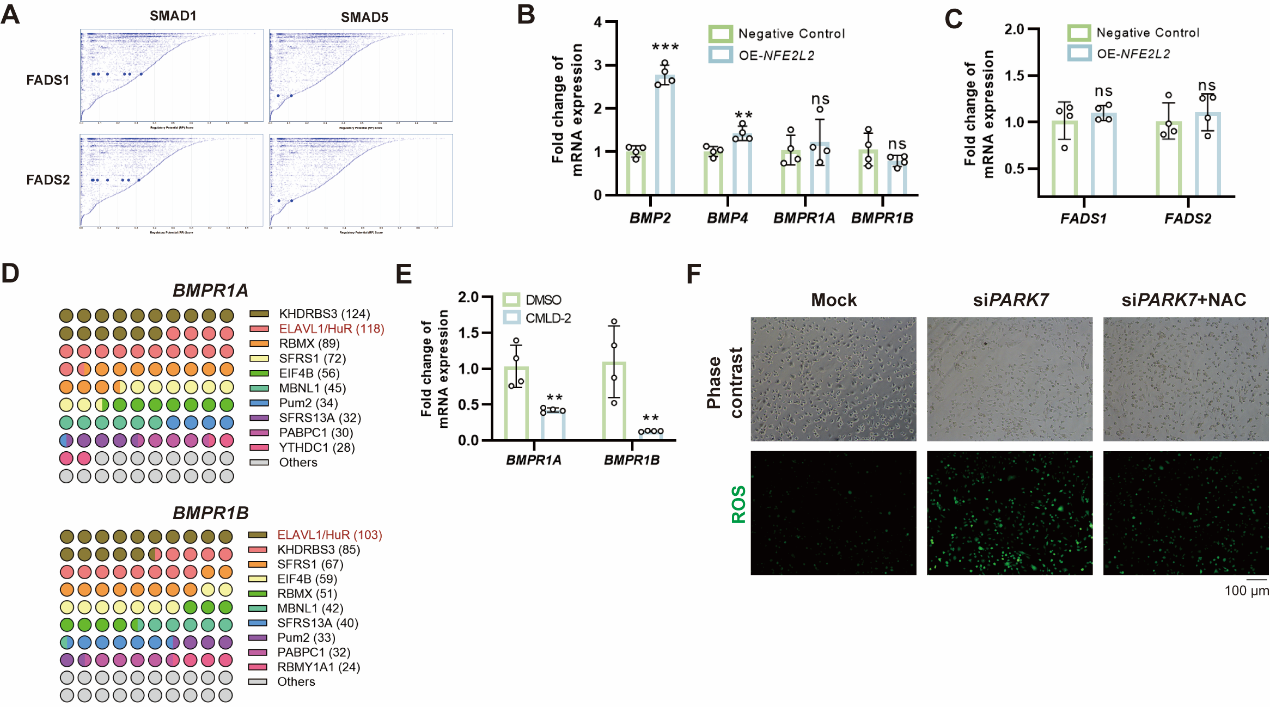


**Figure S6 Supplementary data on PARK7-mediated regulation of FADS1/2 expression through the BMP-SMAD1/5/9 signaling pathway.** (**A**) Prediction of transcriptional regulation of FADS1 and FADS2 by SMAD1/5 based on Cistrome DB database. The larger blue dots represent previously published ChIP-seq results for other cell types in the database. (**B**) Quantitative real-time PCR (qPCR) analysis of *BMP2/4* and *BMPR1A/B* expression after *NFE2L2* overexpression (n = 4). (**C**) qPCR analysis of *FADS1/2* expression after *NFE2L2* overexpression (n = 4). (**D**) Statistical dot plot of RNA-binding protein sites on BMPR1A/B RNA. (**E**) qPCR analysis of *BMPR1A/B* expression after CMLD-2 expression (n = 4). (**F**) Detection of ROS following *PARK7* knockdown and NAC treatment.

ROS, reactive oxygen species; NAC; N-Acetyl-cysteine. *, *P* < 0.05; **, *P* < 0.01; ***, *P* < 0.001.


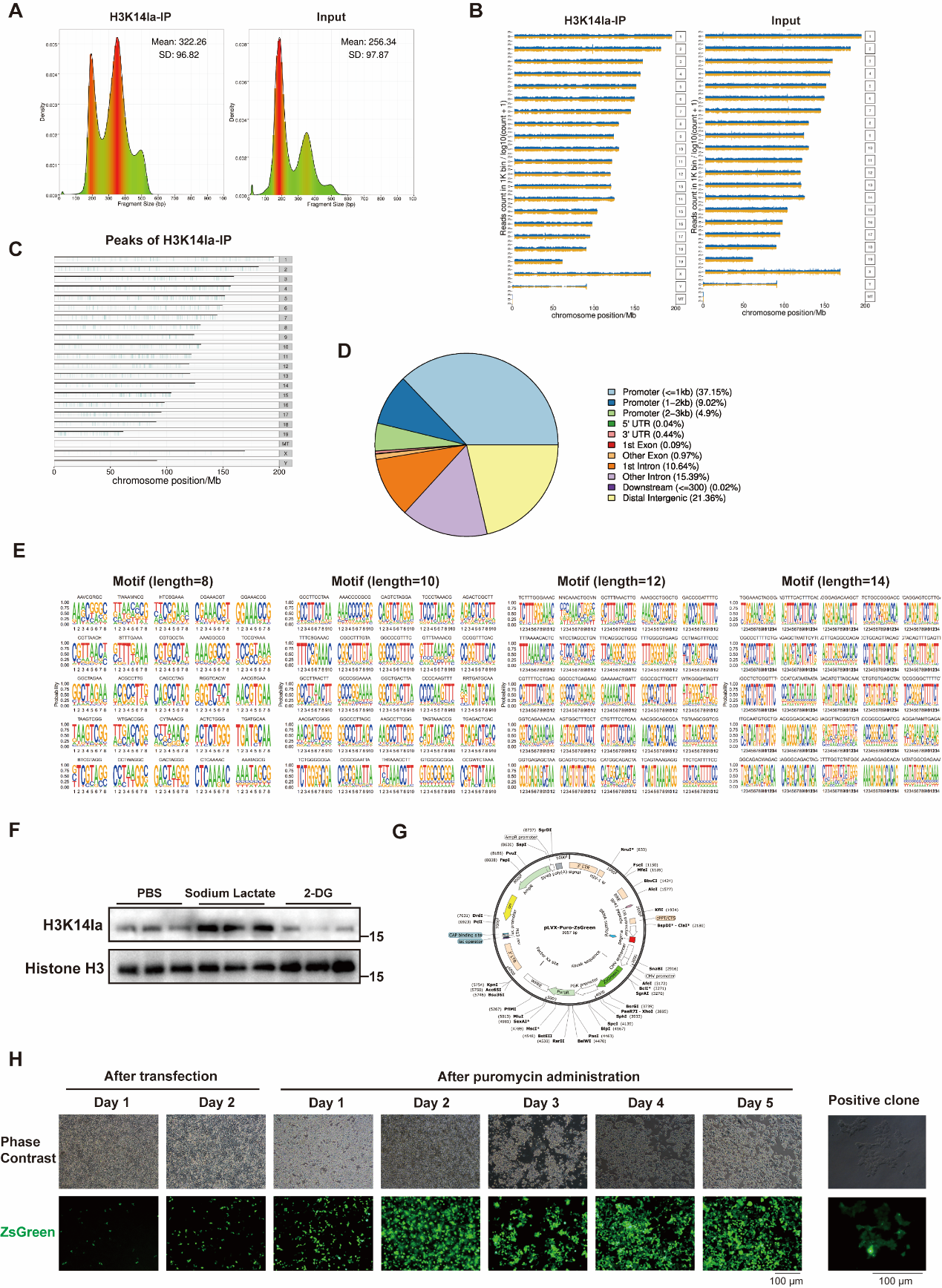


**Figure S7 Supplementary data on H3K14la-mediated regulation of *PARK7* gene transcription.** (**A**) Read length distribution in ChIP-sequencing (ChIP-seq). (**B**) Genome-wide distribution of reads in ChIP-seq. (**C**) Genome-wide distribution of peaks in ChIP-seq. (**D**) Peak distribution across genomic functional regions. (**E**) Predicted motif of H3K14la binding. (**F**) Western blot analysis of the regulatory effects of sodium lactate and 2-DG on H3K14la levels. (**G**) The map of the *pLVX-Puro-ZsGreen* plasmid carrying pegRNA for prime editing. (**H**) Observation of ZsGreen-positive 293T cells following transfection, and puromycin selection, with subsequent isolation of positive monoclonal colonies.

2-DG, 2-deoxy-d-glucose.
