# Supplementary material for: Metabolic Interplay in Acute Lung Injury: PARK7 Integrates FADS1/2‐Dependent PUFA Metabolism and H3K14 Lactylation to Attenuate Endothelial Ferroptosis and Dysfunction
Source: Adv Sci (Weinh). 2025 Sep 30;12(46):e08725. doi: 10.1002/advs.202508725 (PMC12697827; doi:10.1002/advs.202508725)

The order of all samples displayed in the original images is **consistent** with that in the figures of the article. The **red boxes** indicate the representative images used in the article, while the **blue boxes** denote the images used for replication or quantification. Each group included at least **three biological replicates**, even when detected on different membranes. To ensure the reliability of Western blot trends, the target protein and internal reference protein were incubated on the same membrane after stripping (unless otherwise specified).

Figure 1K

FADS1

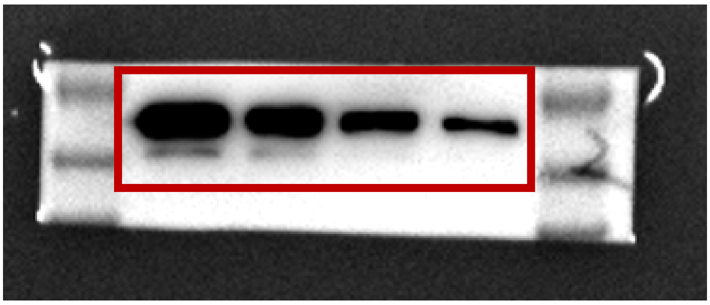

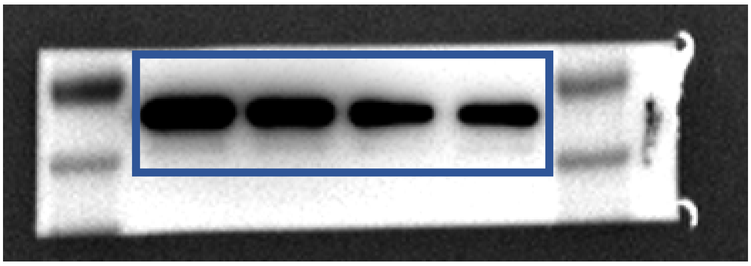

FADS2

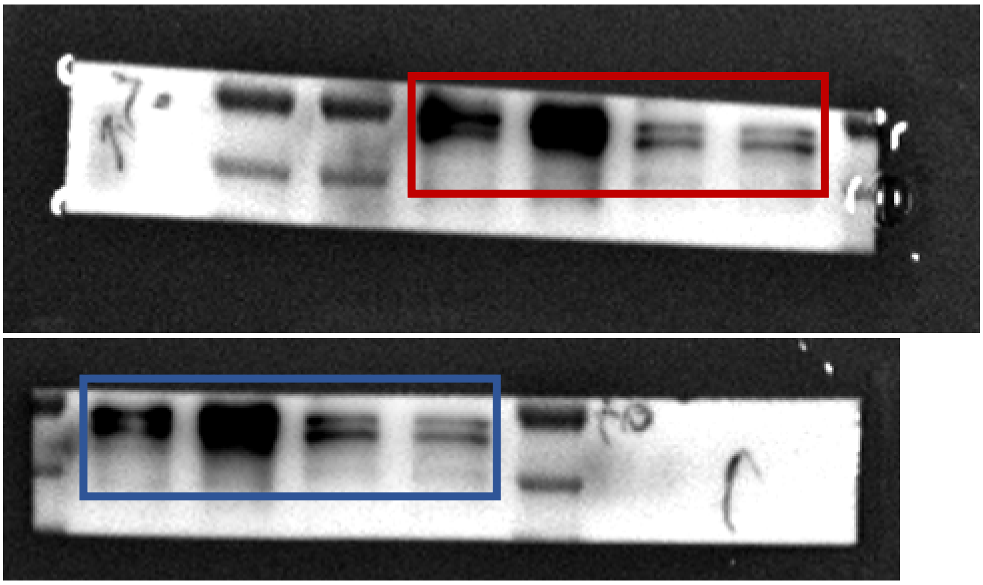

SCD1

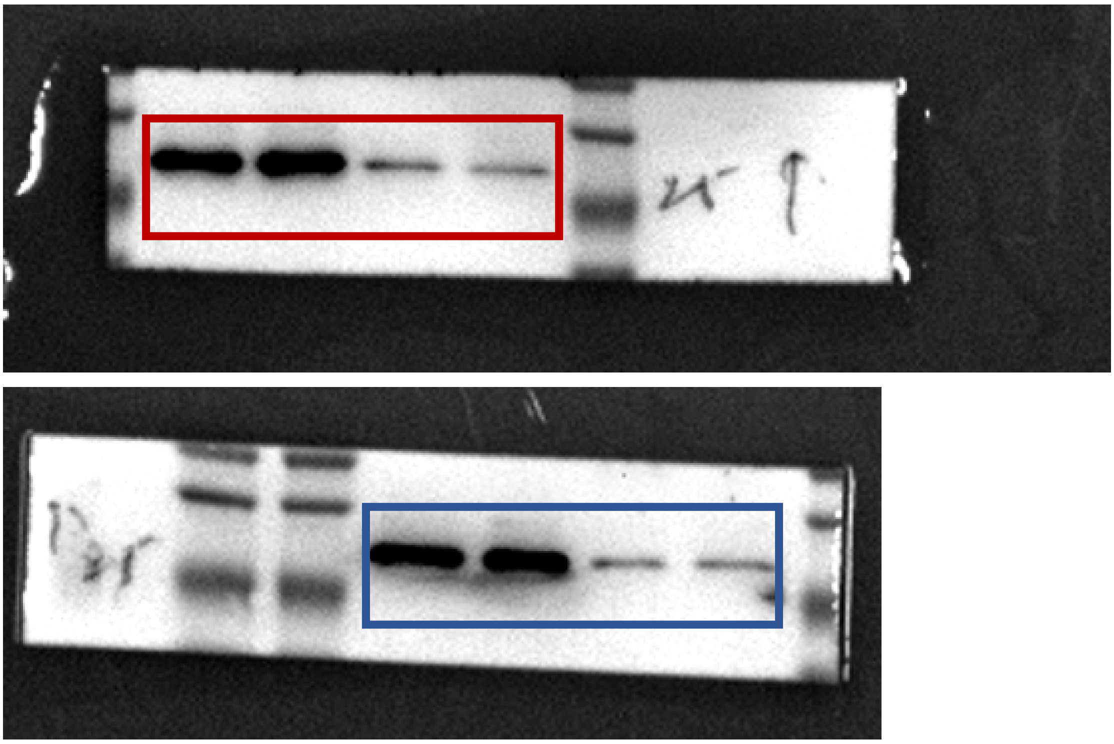

GAPDH

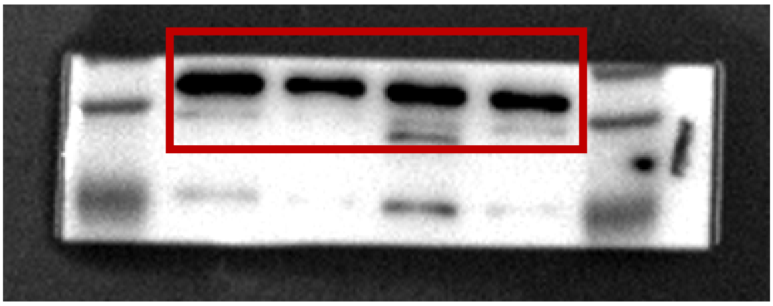

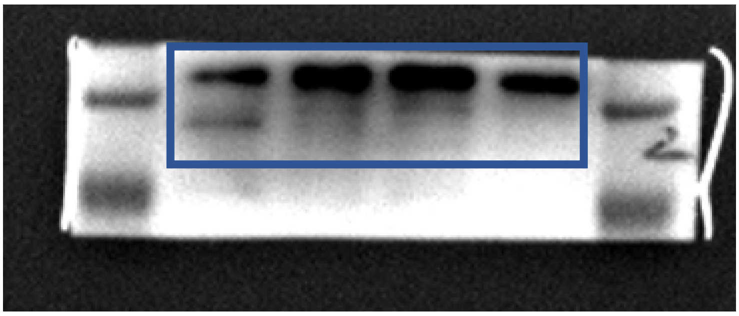

Important Technical Note: In this experiment, the same set of samples was divided and detected across two membranes: SCD1 and FADS2 on one membrane, and GAPDH and FADS1 on the other, with identical loading patterns.

Figure S1J

FADS1

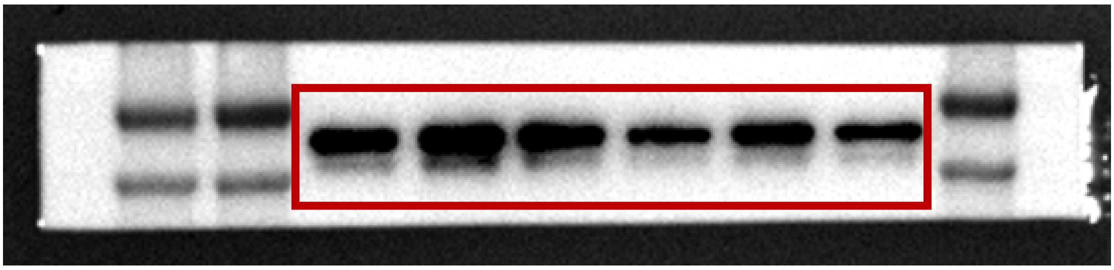

FADS2

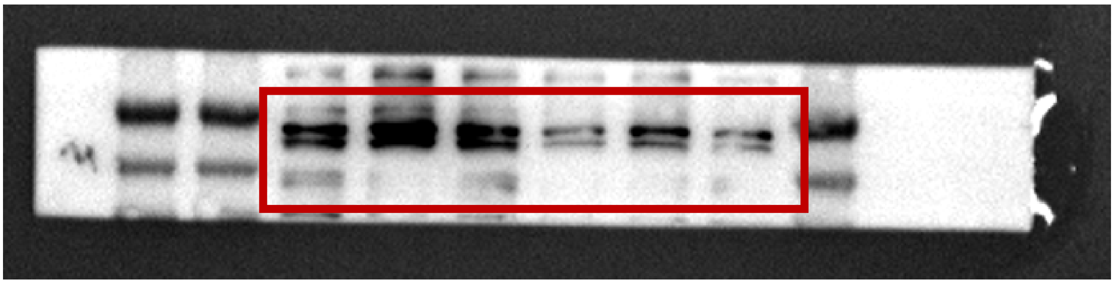

SCD1

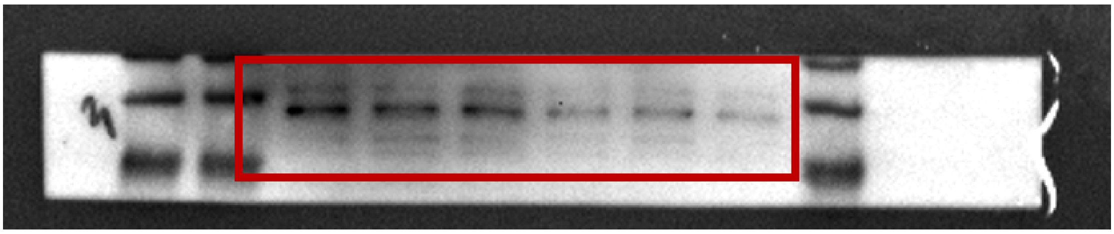

GAPDH

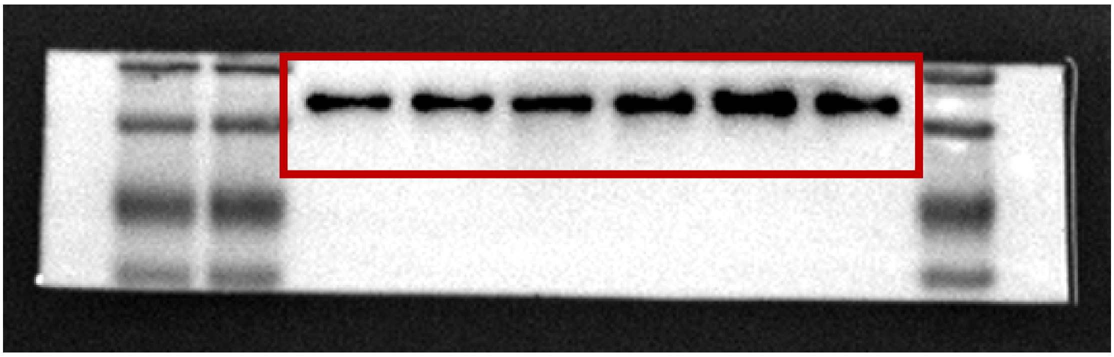

Important Technical Note: In this experiment, the same set of samples was divided and detected across two membranes: SCD1

and FADS2 on one membrane, and GAPDH and FADS1 on the other, with identical loading patterns.

Figure S1L

FADS1

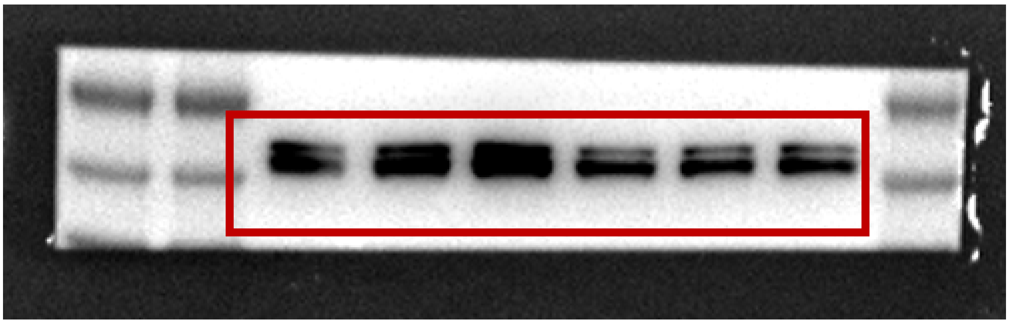

FADS2

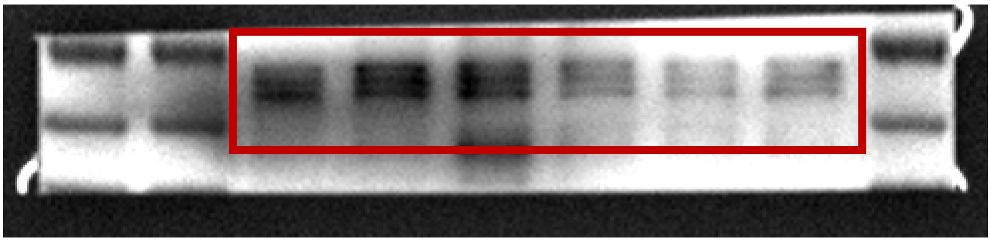

SCD1

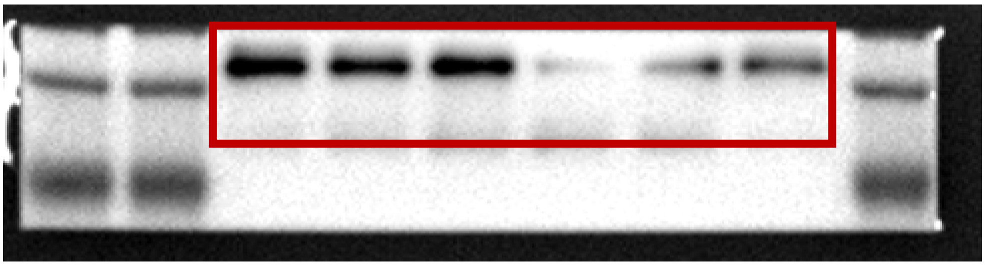

GAPDH

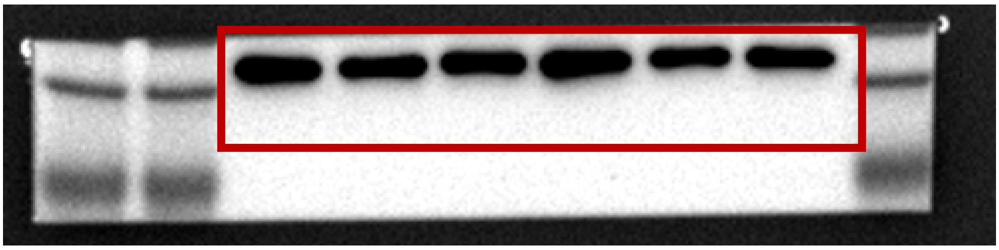

Important Technical Note: In this experiment, FADS1 was detected on a separate membrane.

Figure 2K

ASCL4

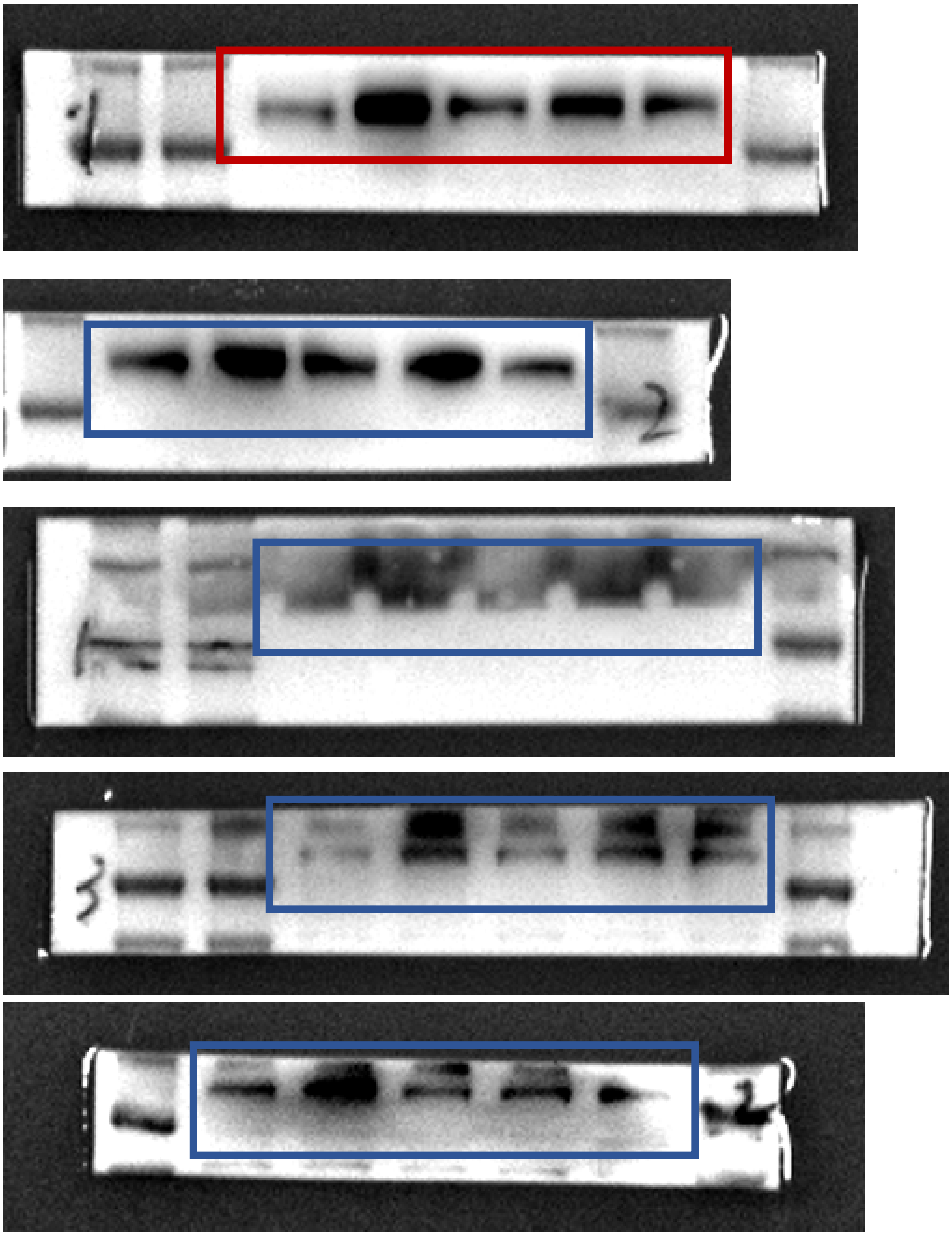

GPX4

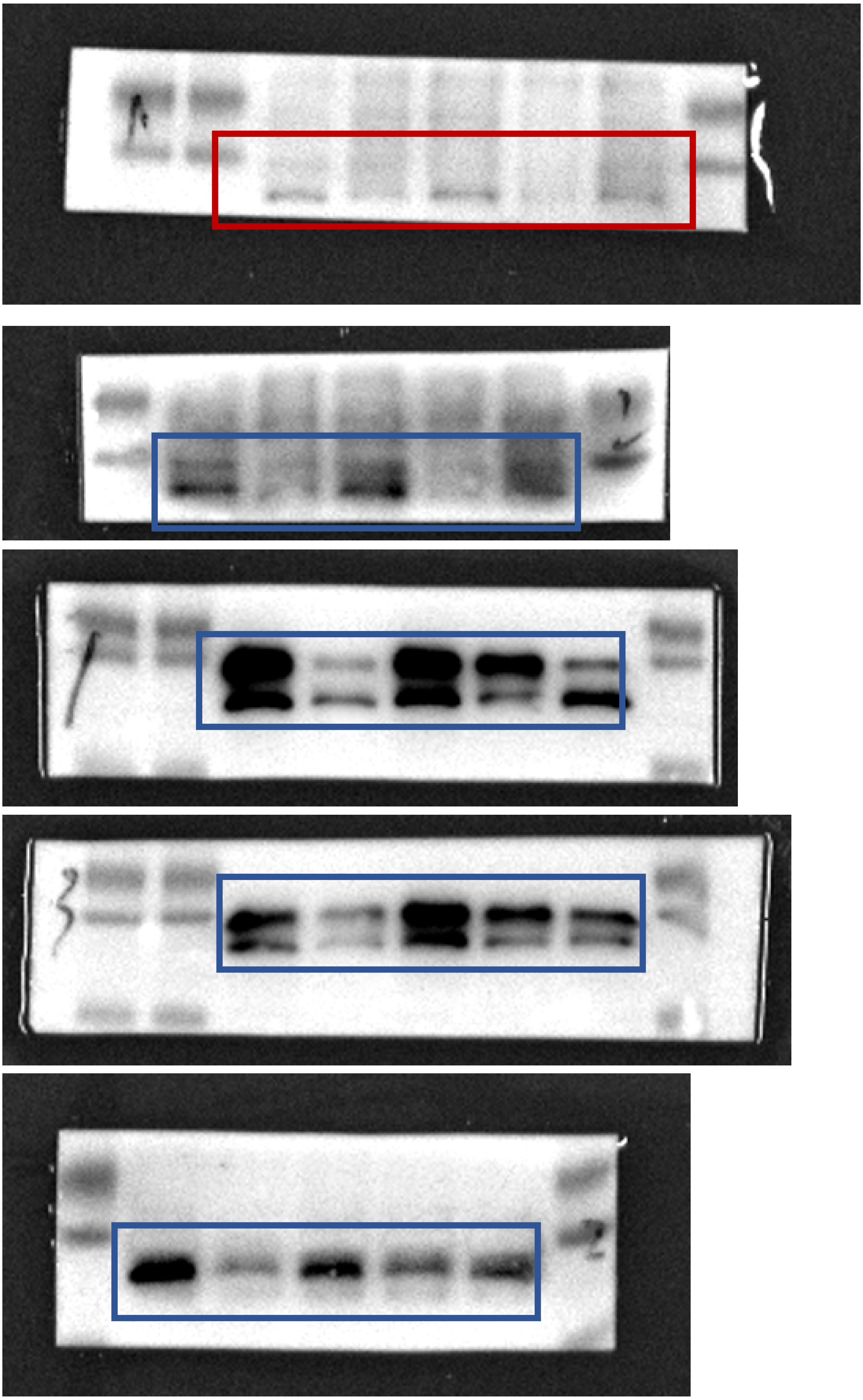

FTH1

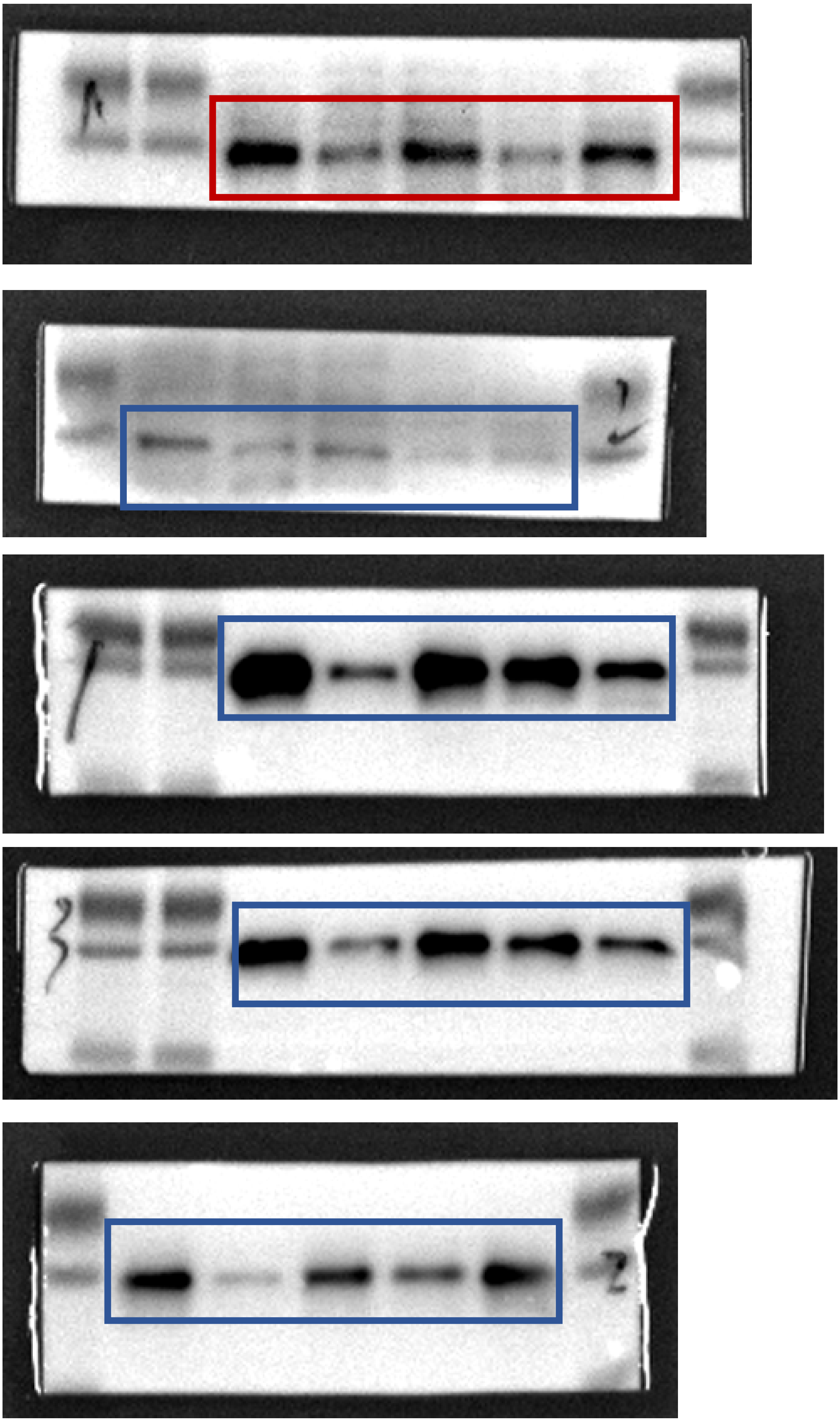

GAPDH

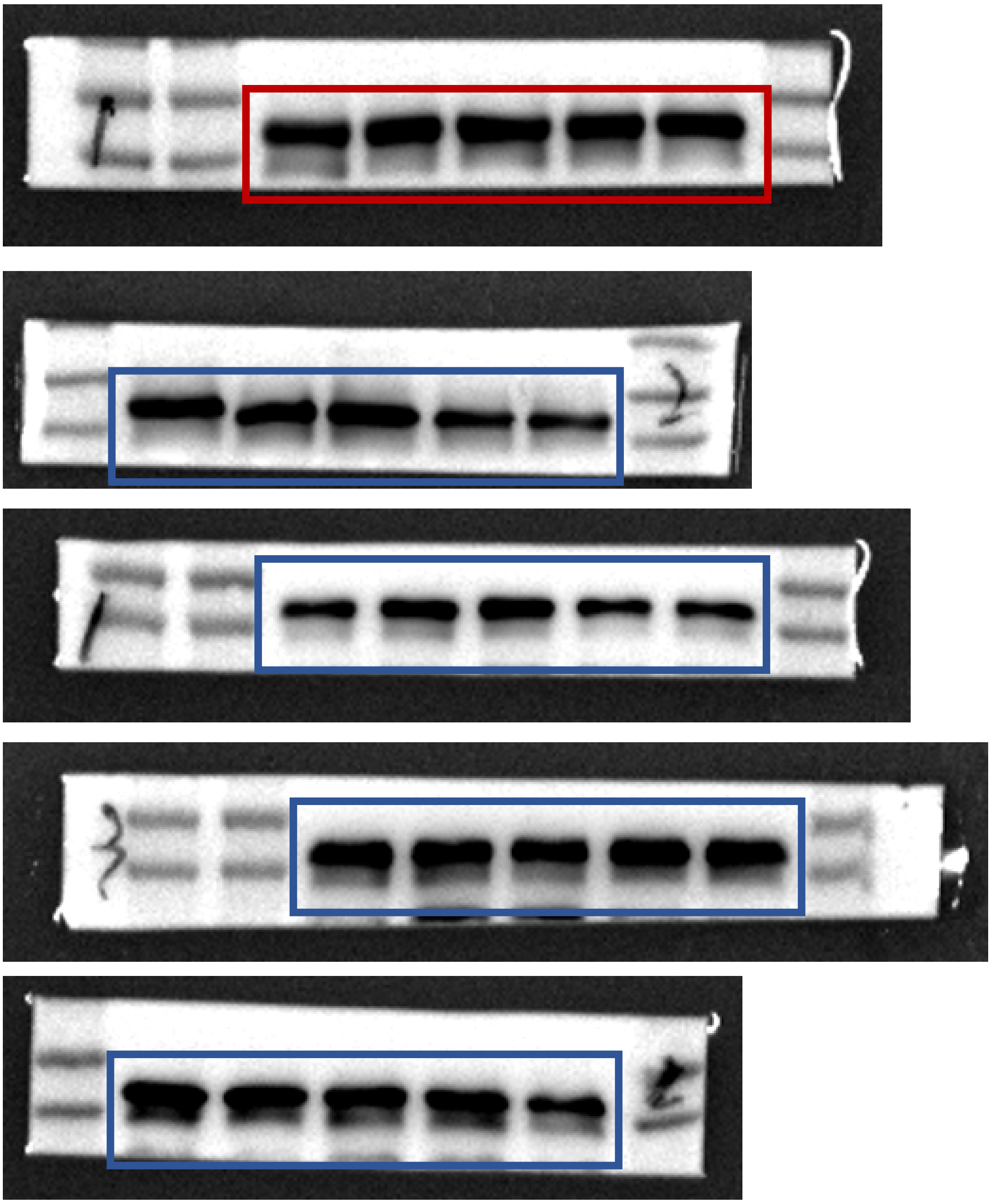

Figure 2L

ASCL4

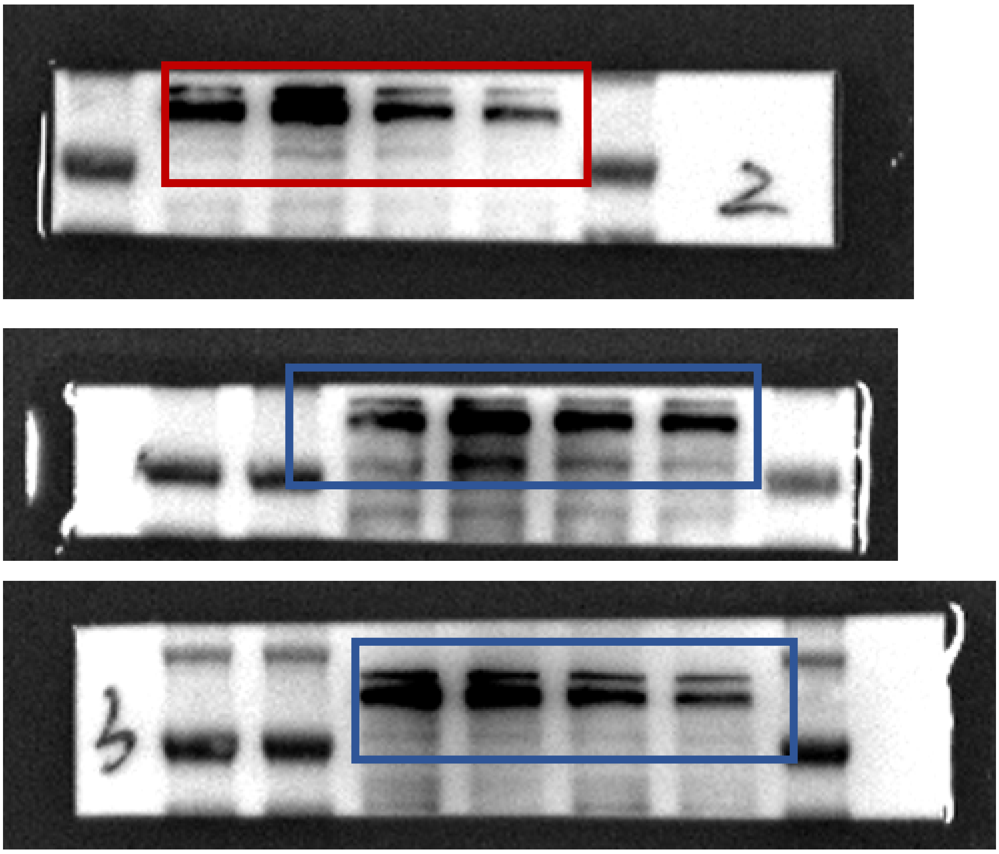

GPX4

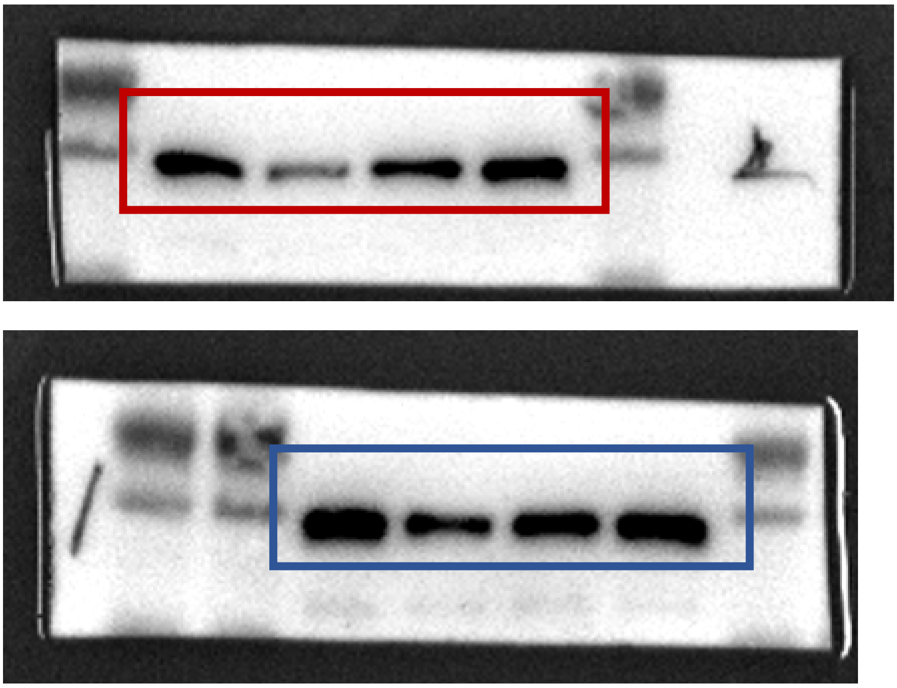

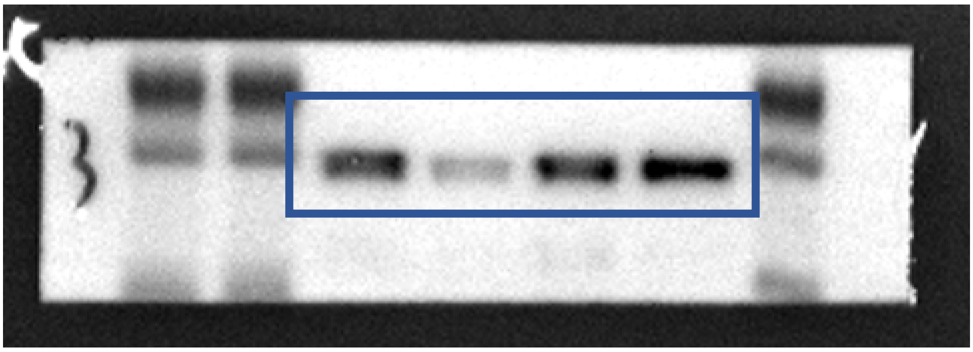

FTH1

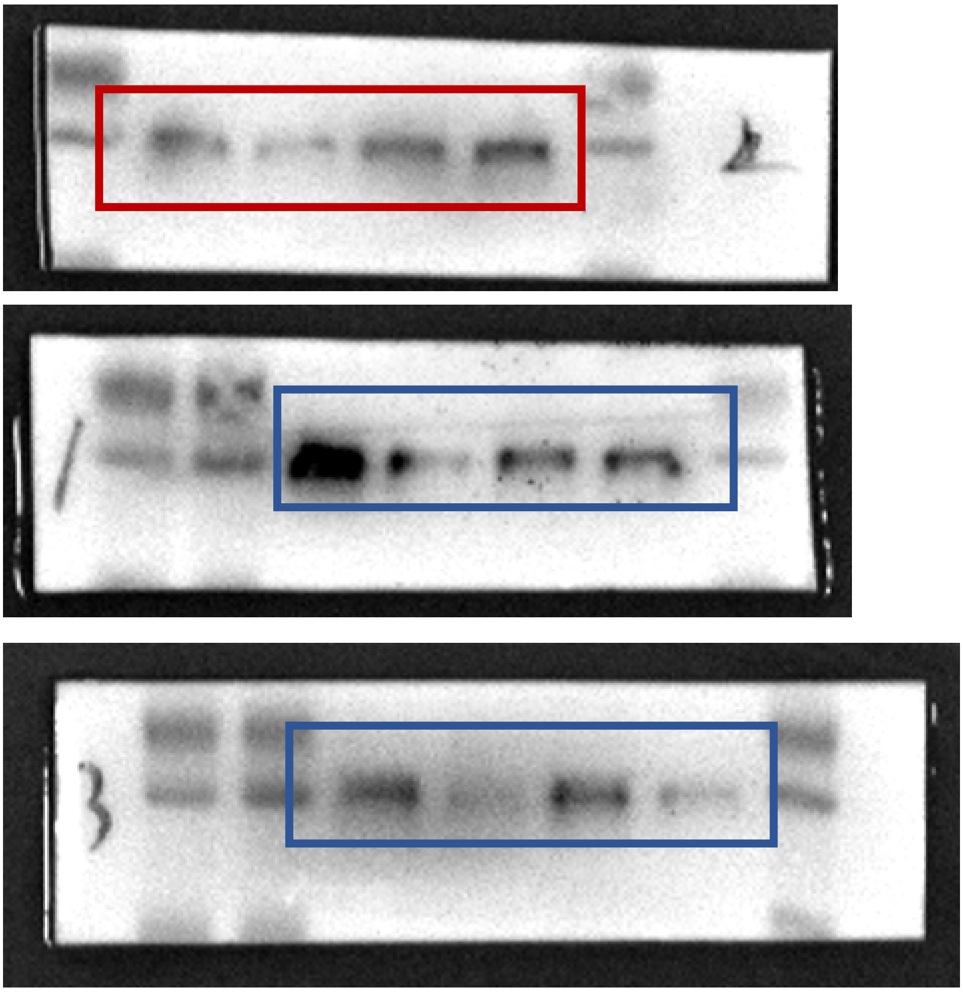

GAPDH

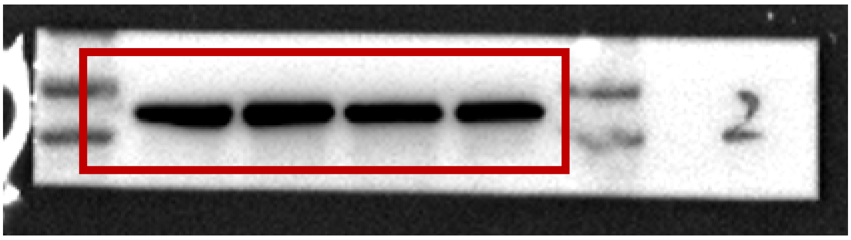

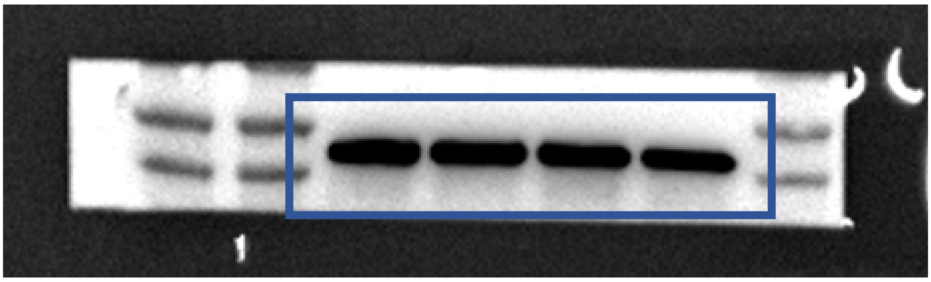

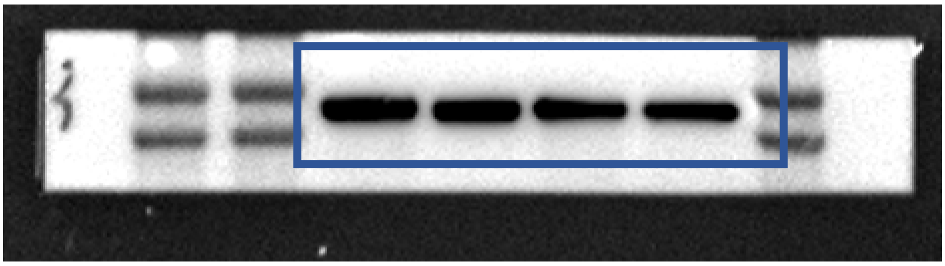

Figure 2M

KEAP1

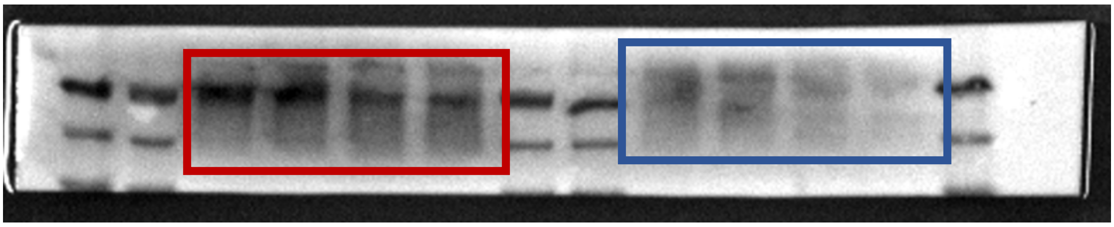

NRF2

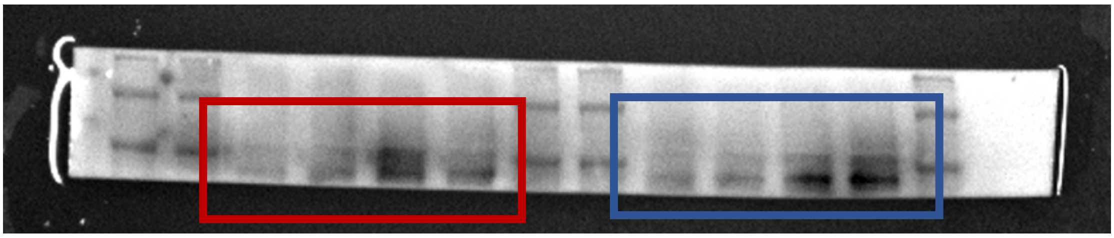

GAPDH

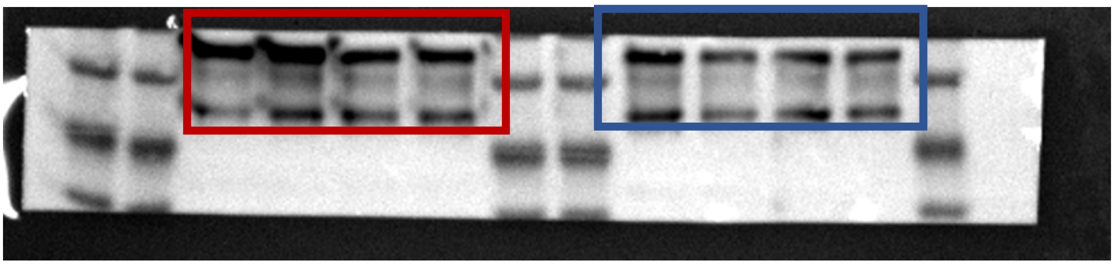

Figure 2N

KEAP1

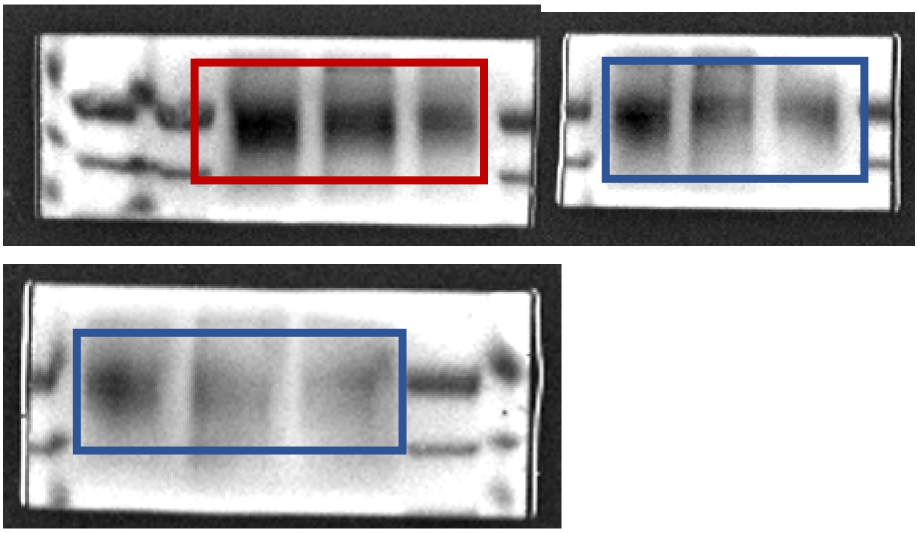

NRF2

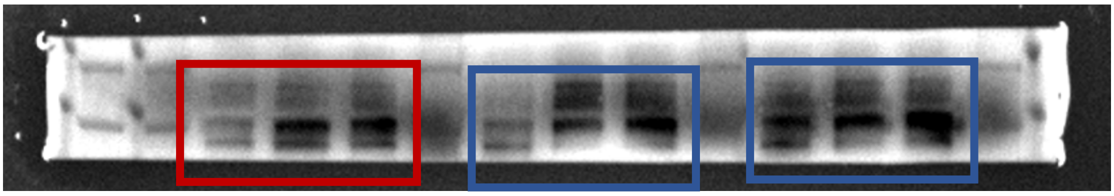

GAPDH

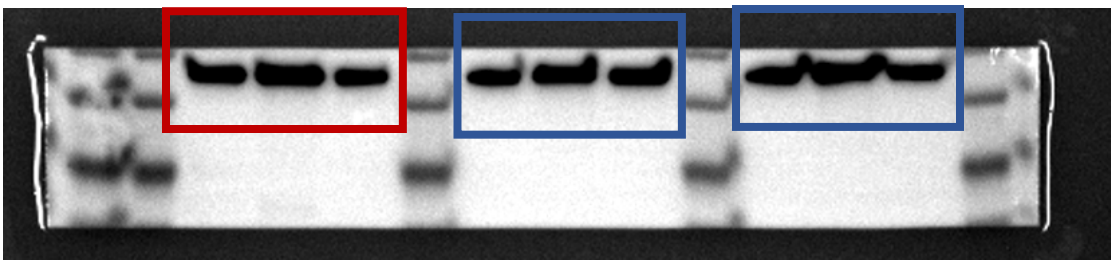

Note: These bands originated from the same membrane, even though KEAP1 was detected separately.

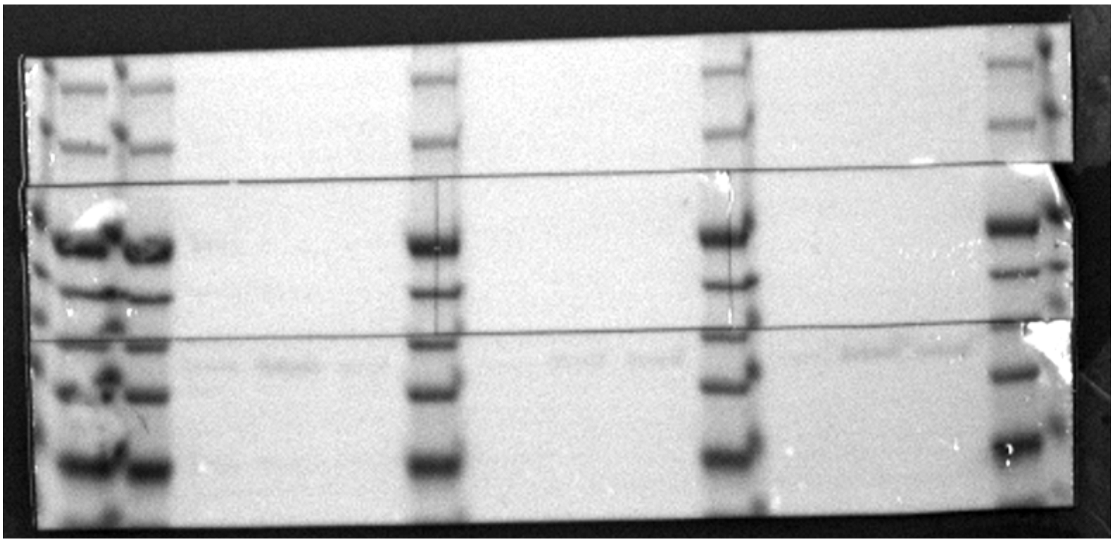

Figure 2O

GPX4

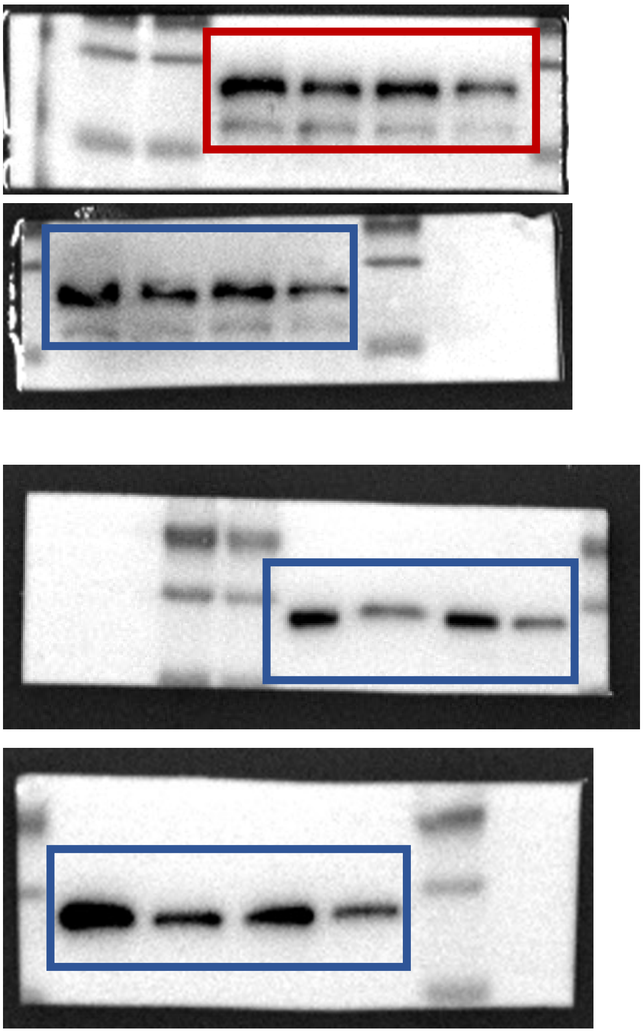

GAPDH

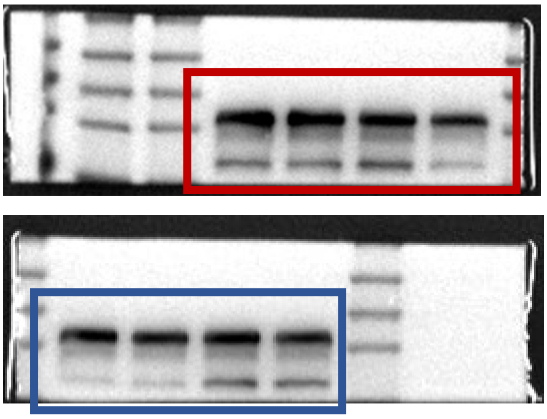

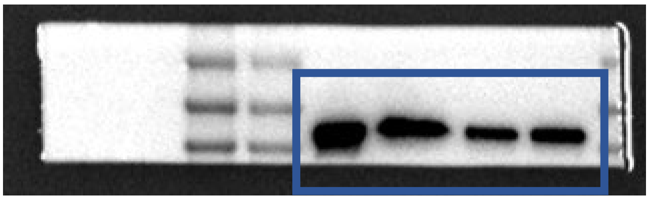

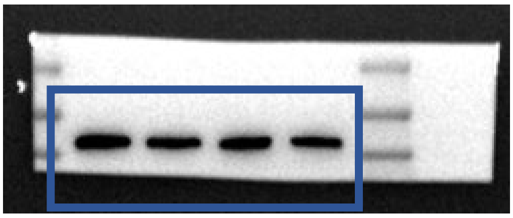

Figure 2P

GPX4

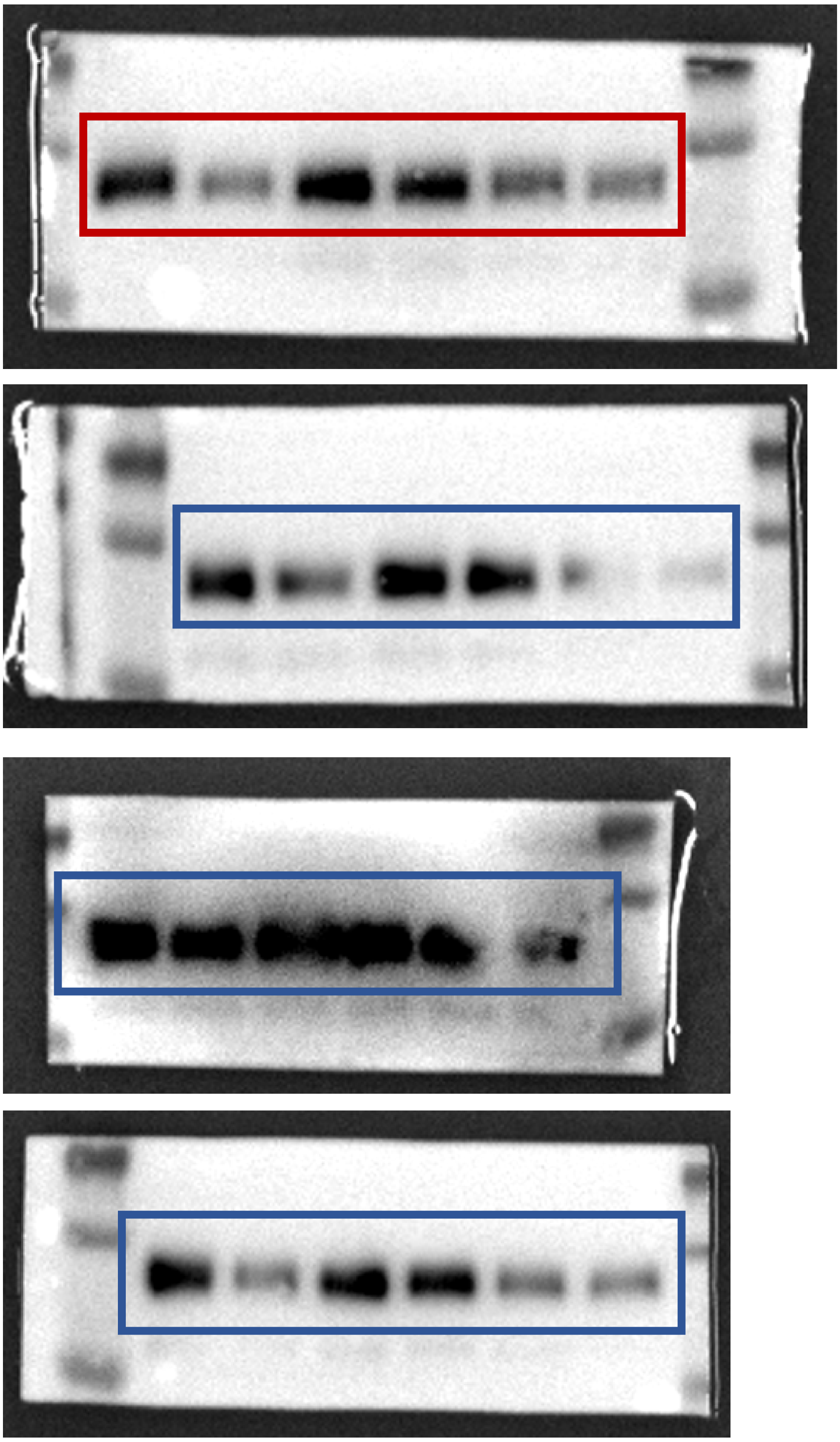

GAPDH

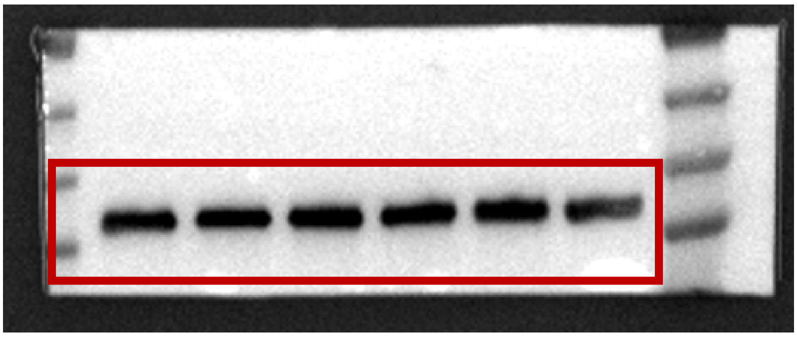
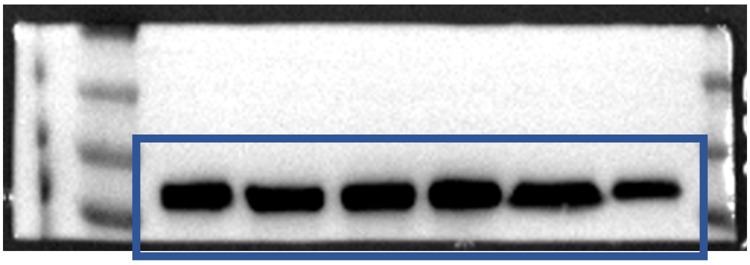
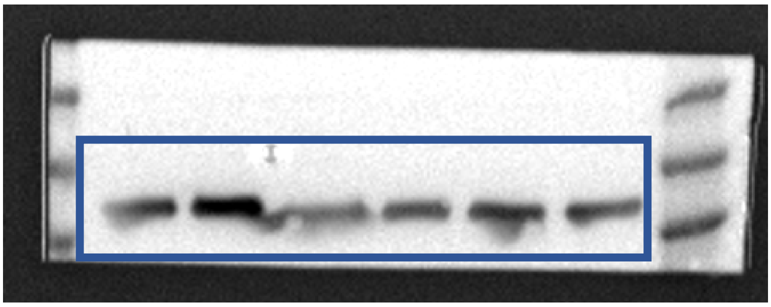
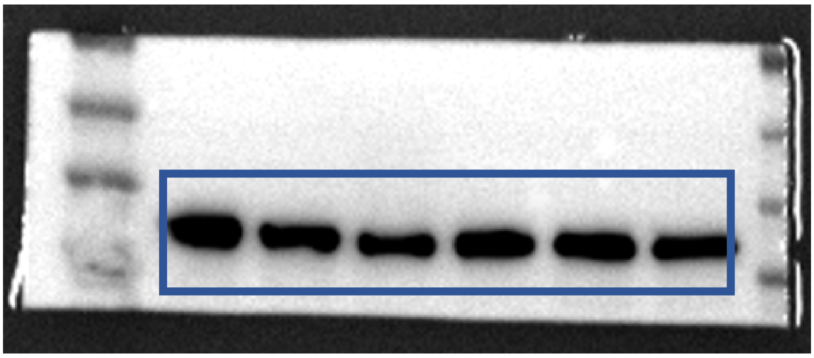

Figure S2C

FADS1

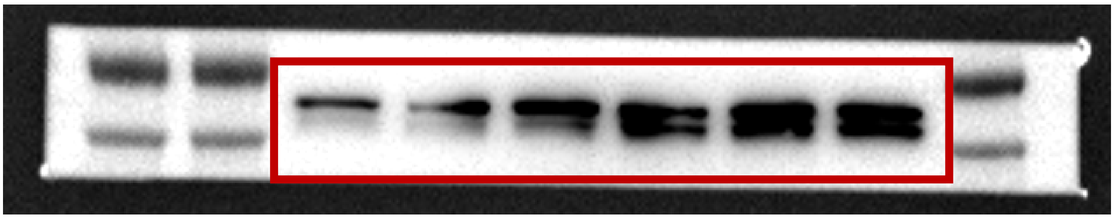

GAPDH

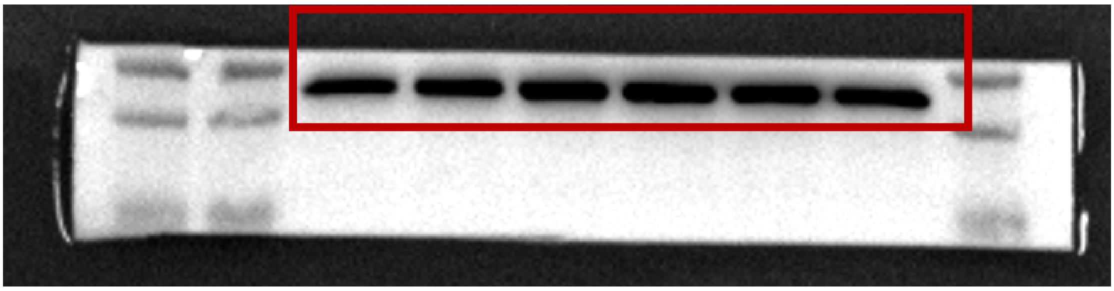

Figure S2D

FADS2

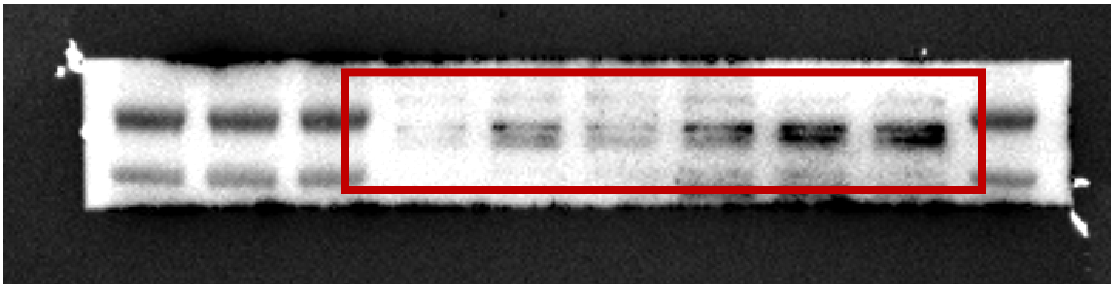

GAPDH

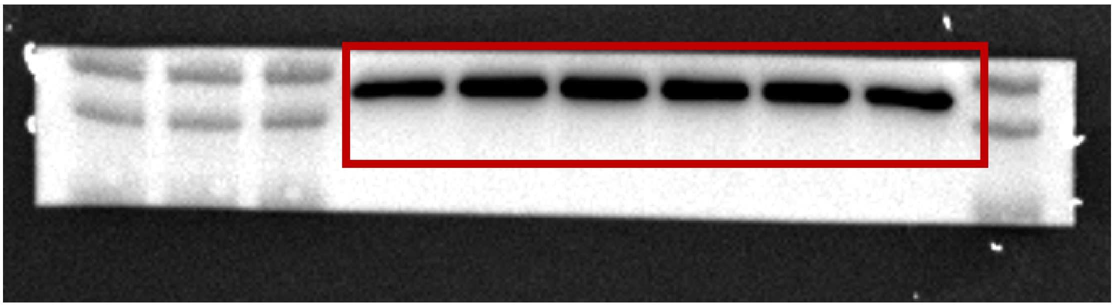

Figure S2G

FADS1

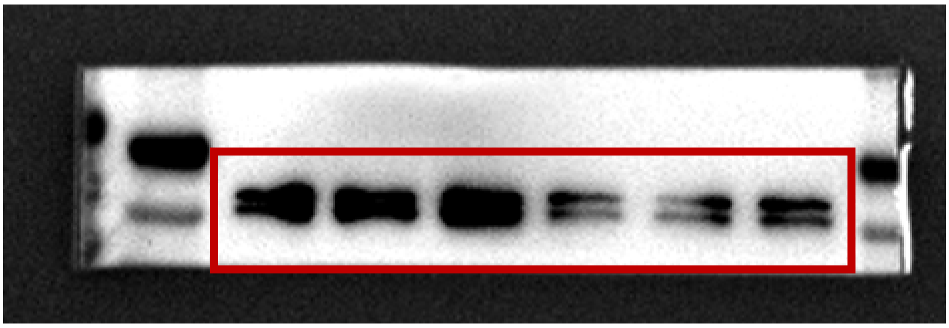

GAPDH

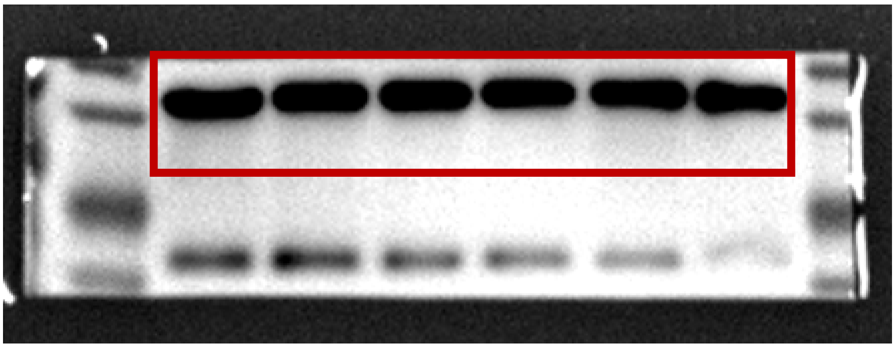

Figure S2H

FADS2

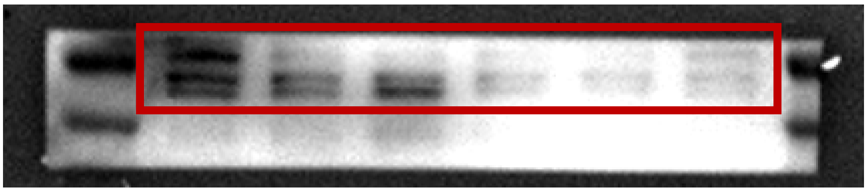

GAPDH

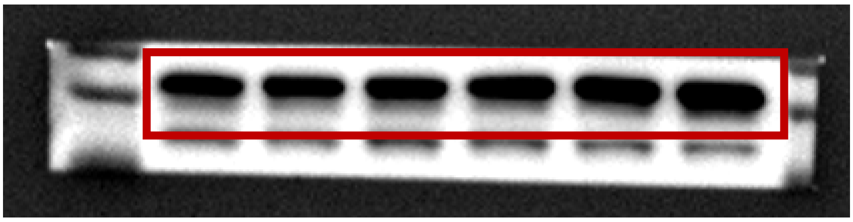

Figure S3C

GPX4

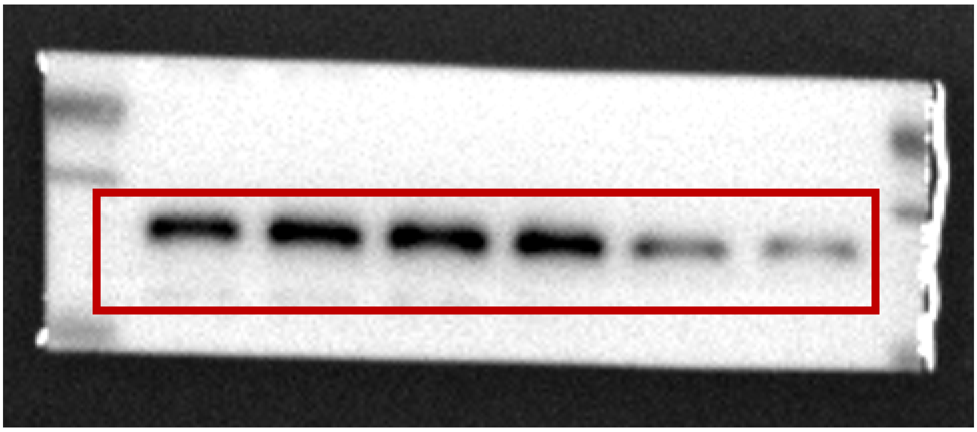

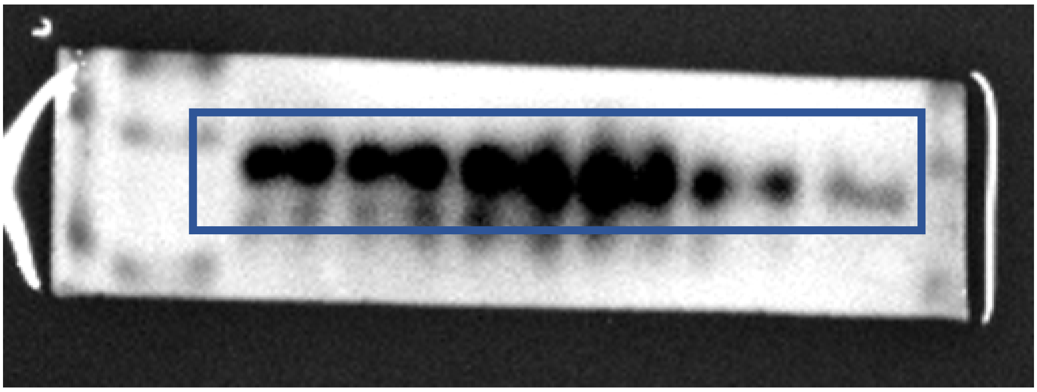

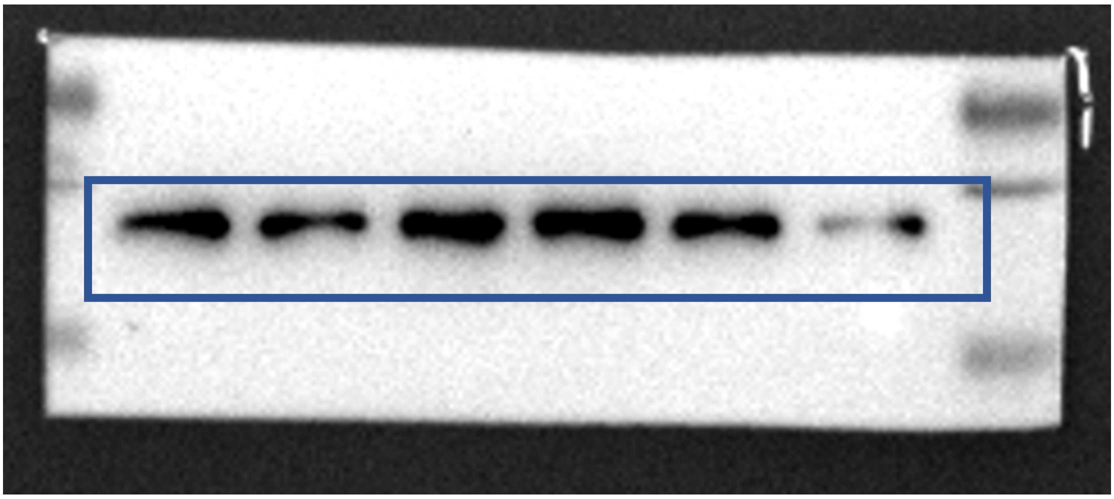

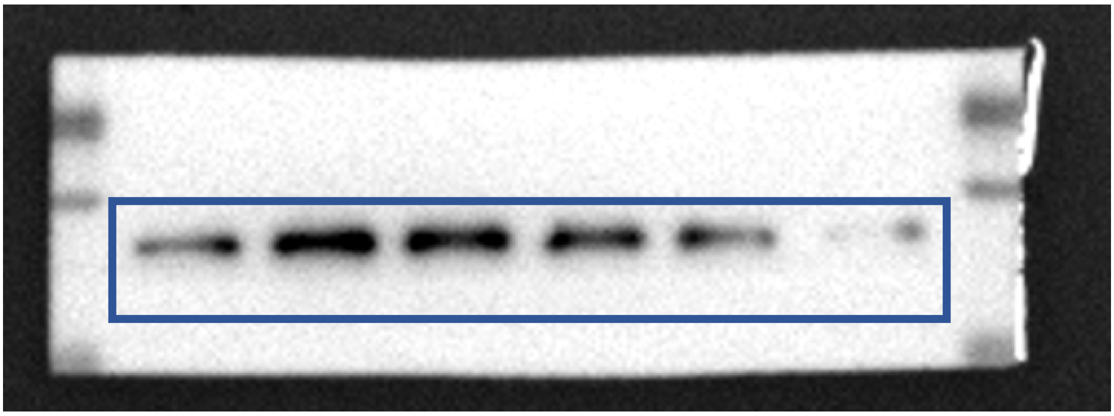

GAPDH

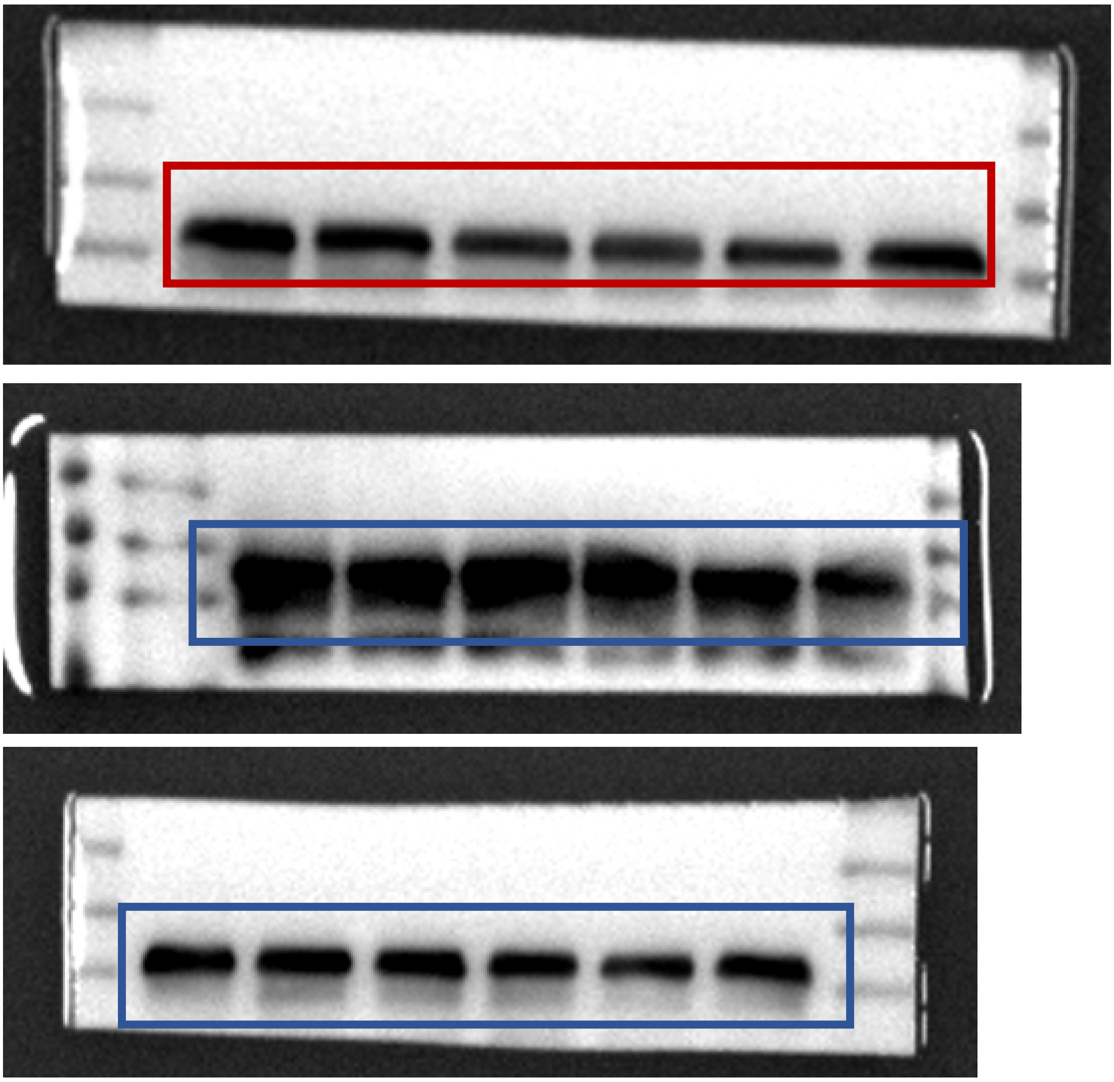

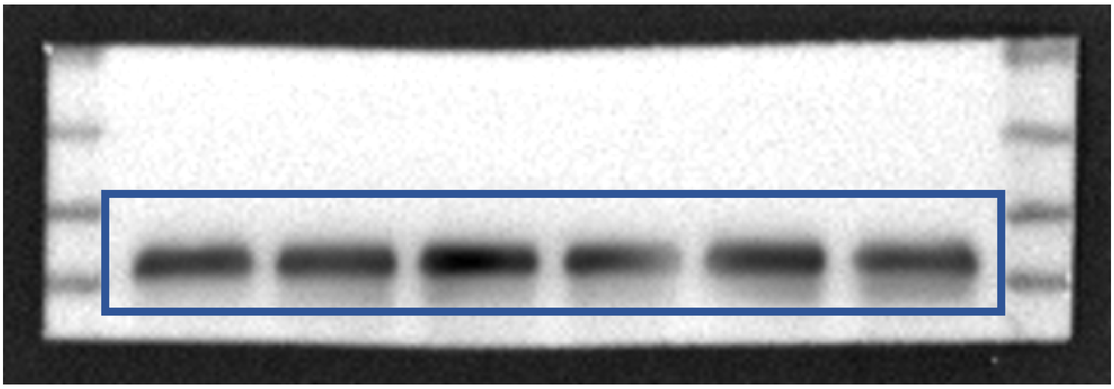

Figure 3B

ZO-1

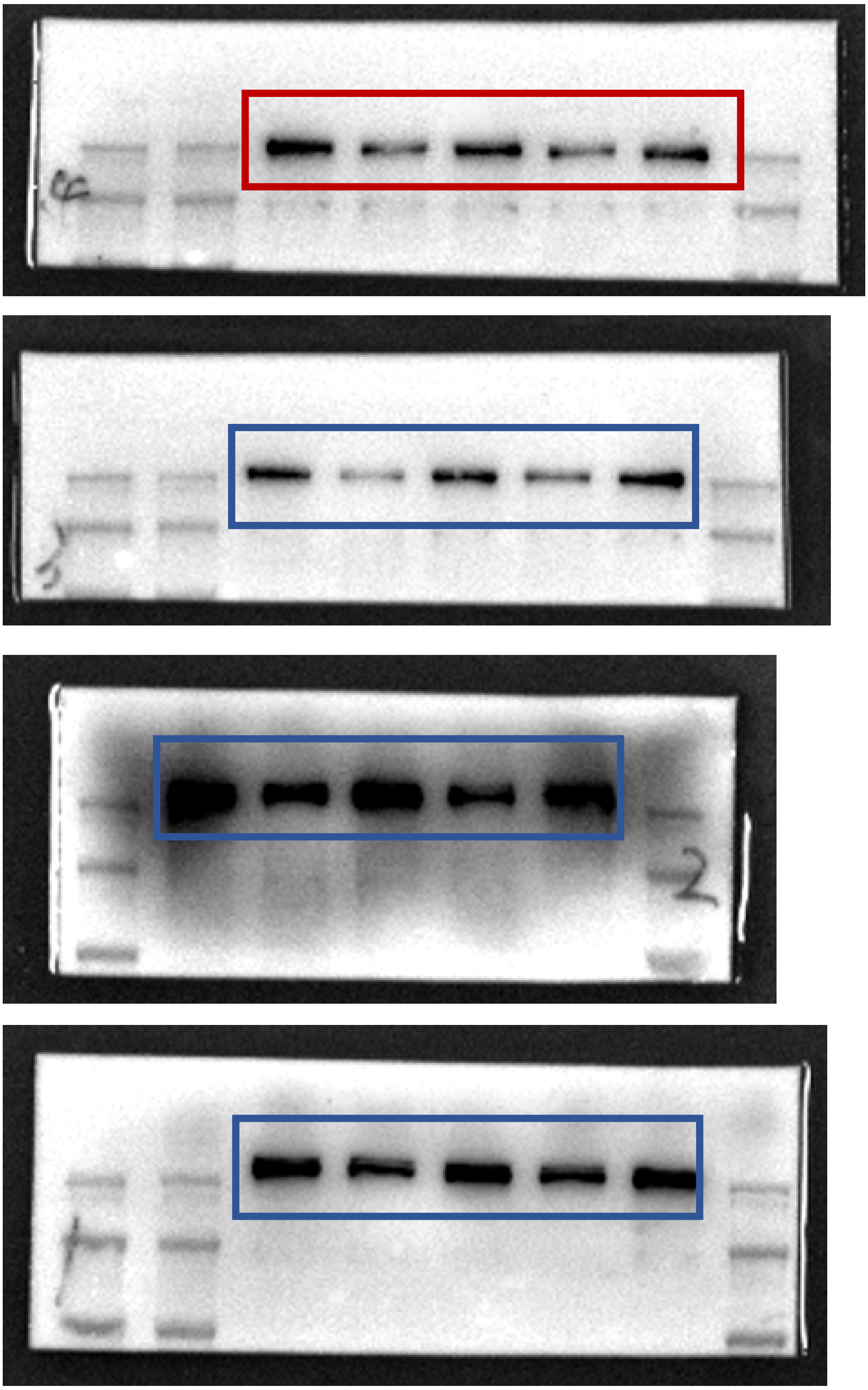

Occludin

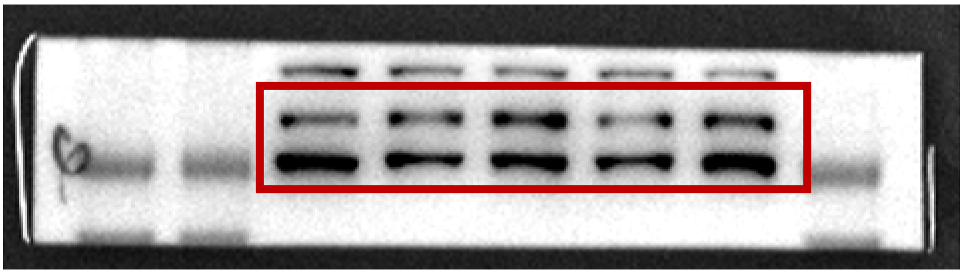

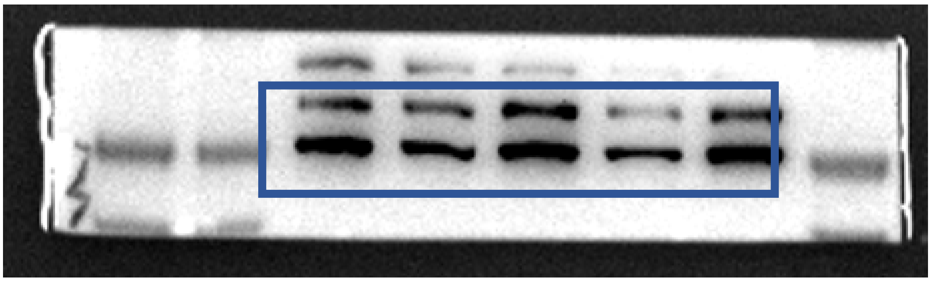

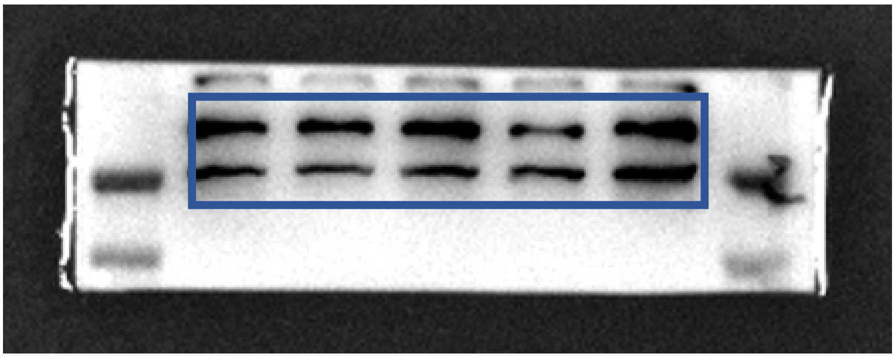

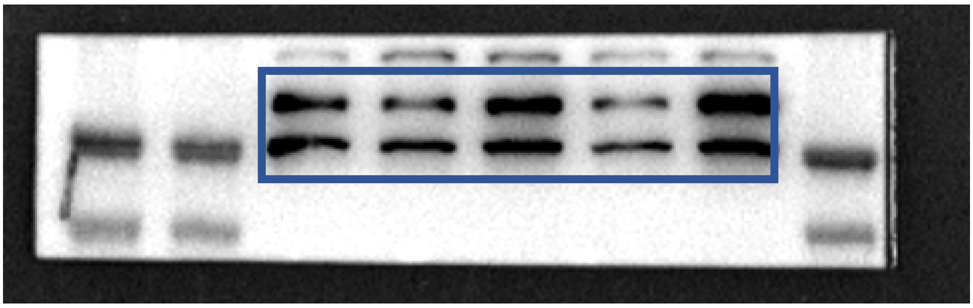

GAPDH (for ZO-1 & Occludin)

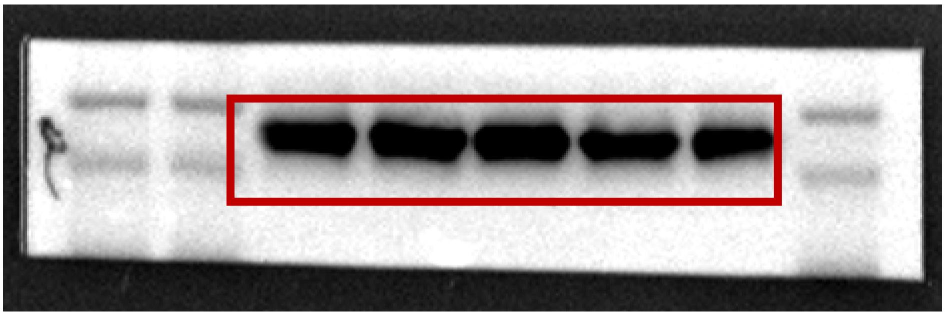

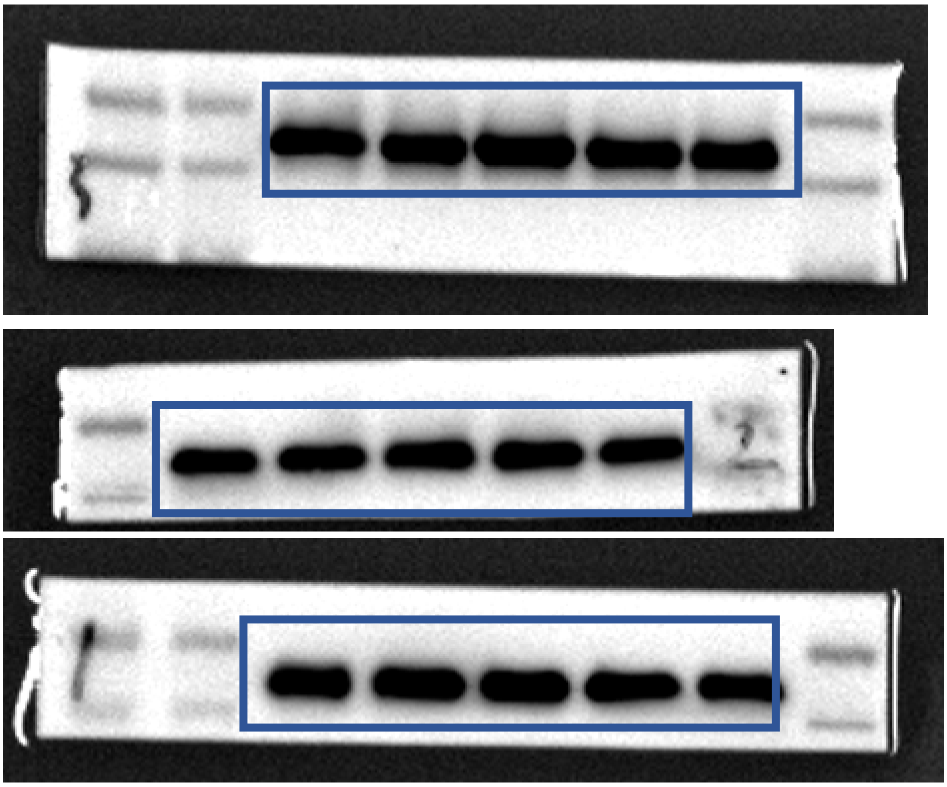

VE-cadherin

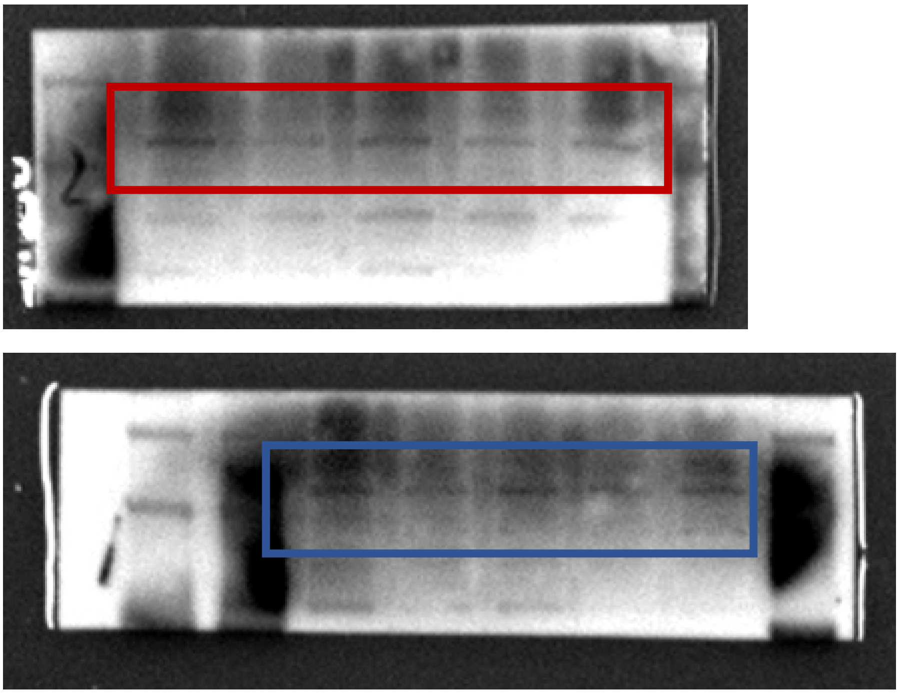

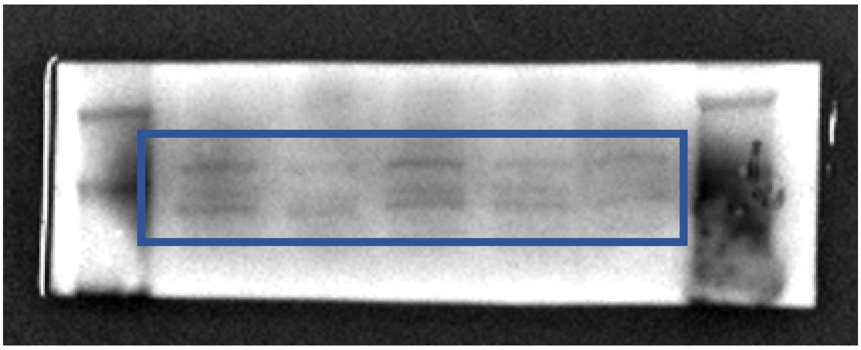

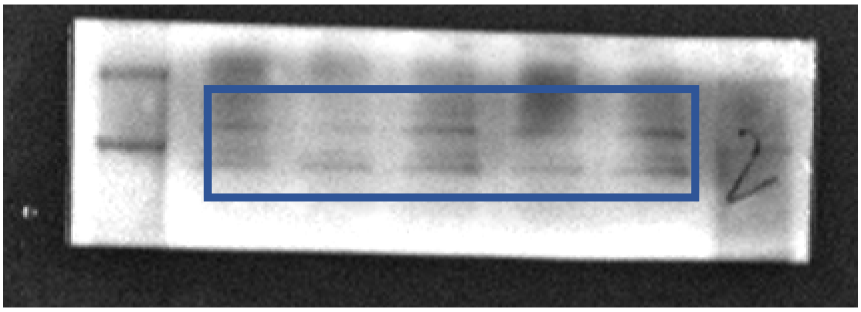

GAPDH (for VE-cadherin)

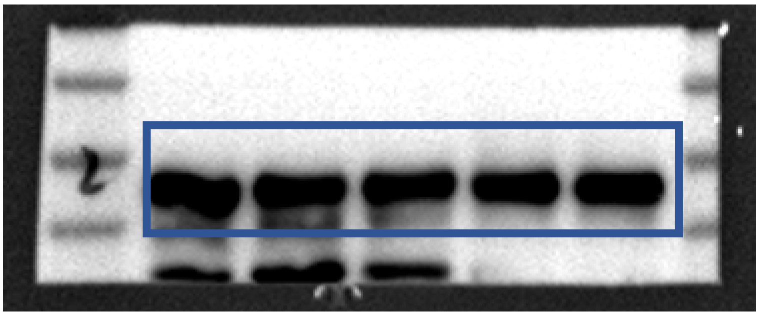

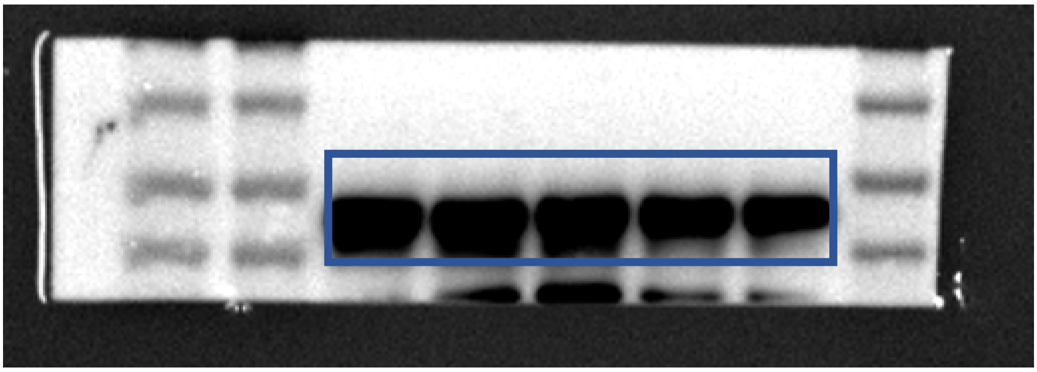

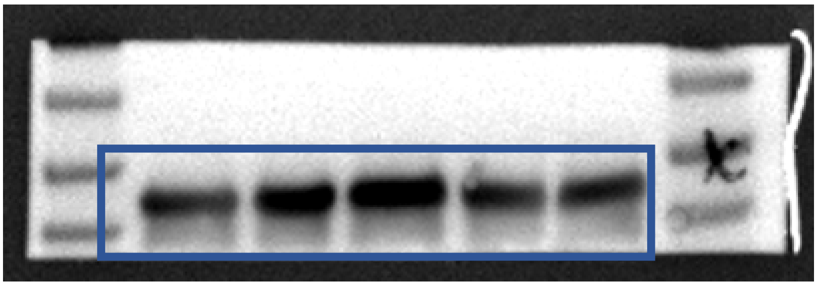

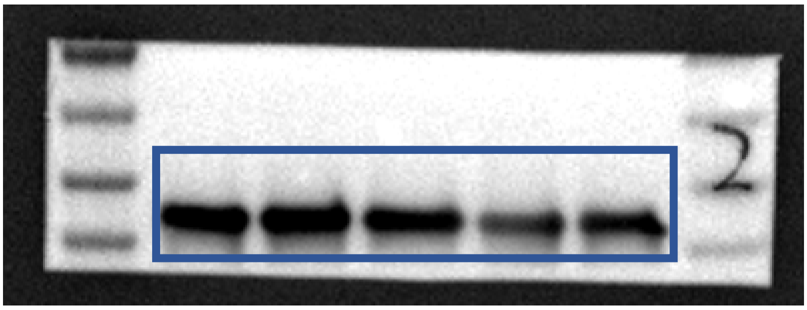
2

Figure 3D

ZO-1

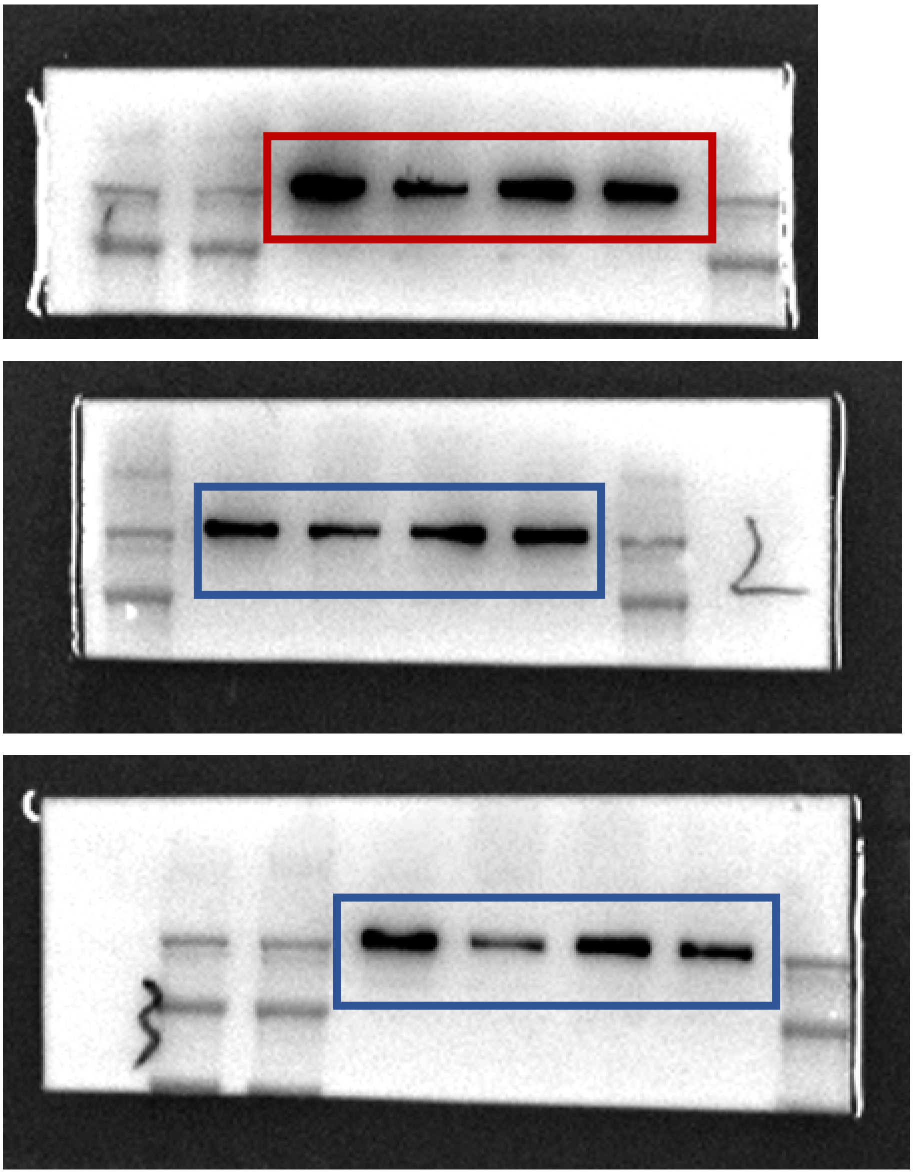

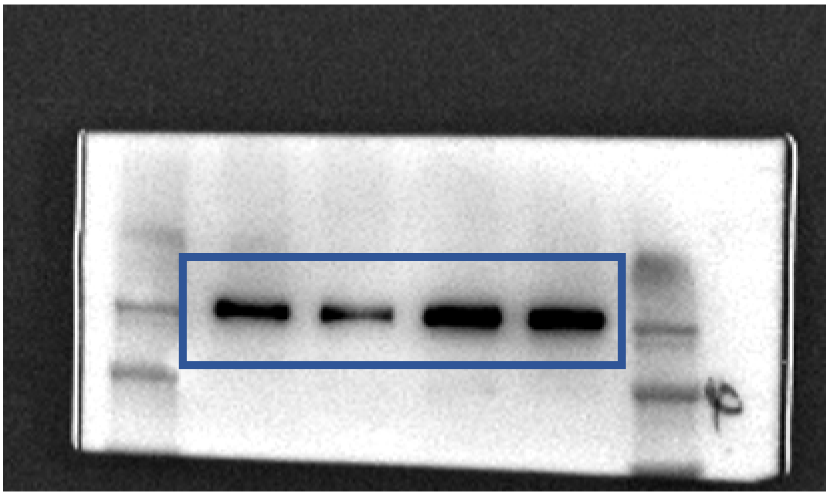

Occludin

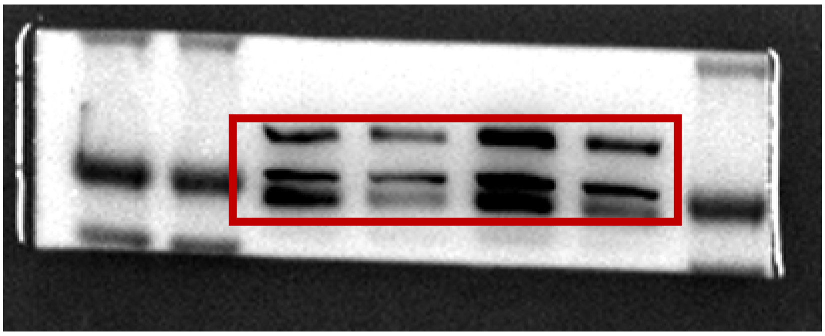

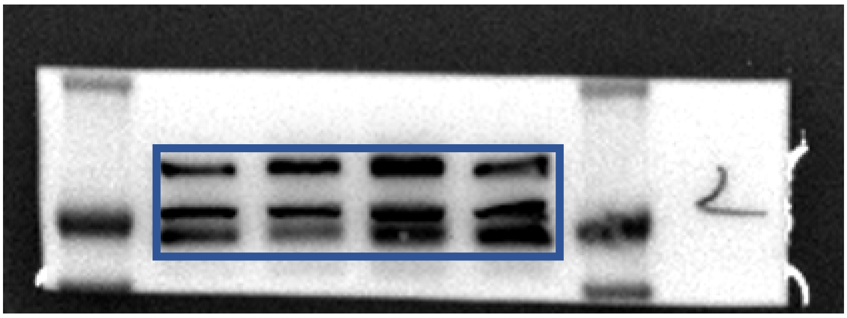

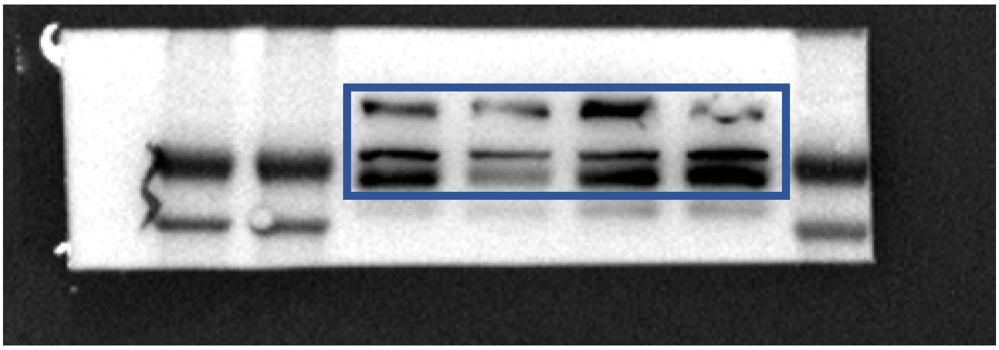

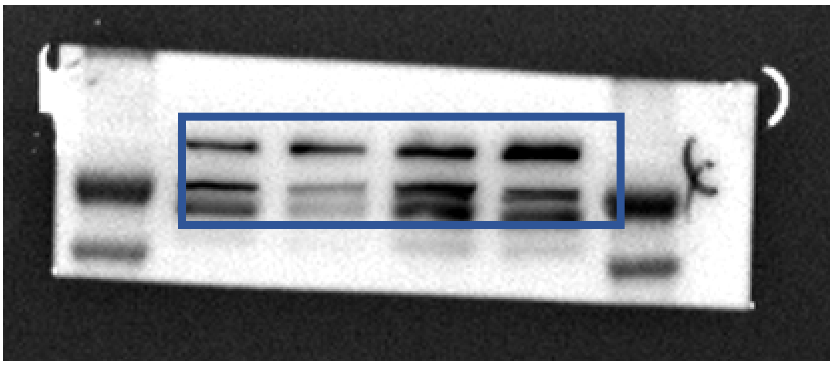

GAPDH (for ZO-1 & Occludin)

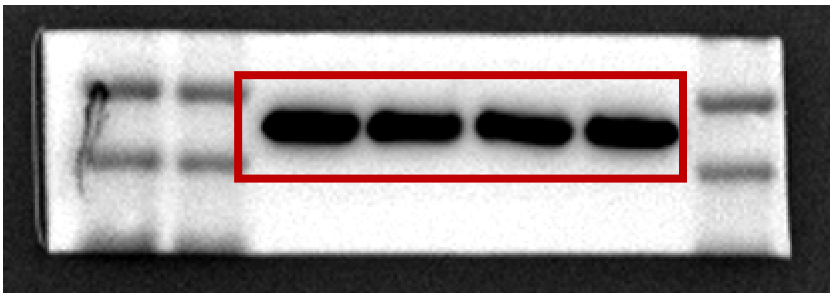

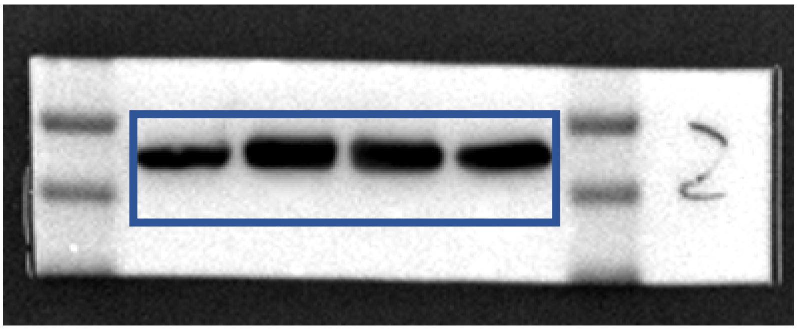

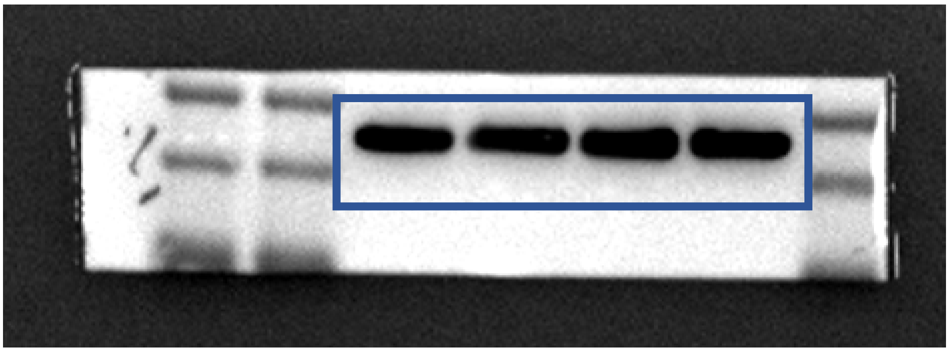

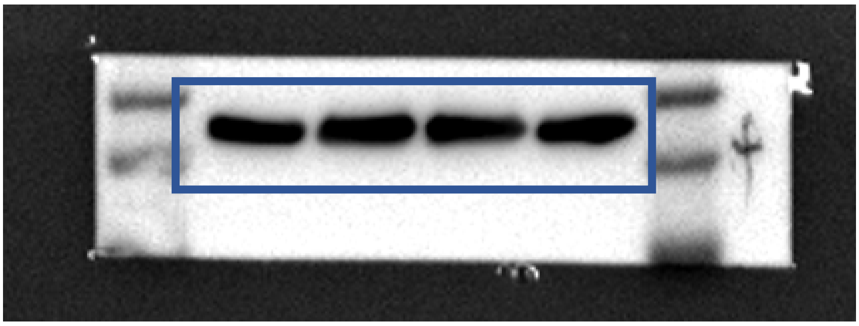

VE-cadherin

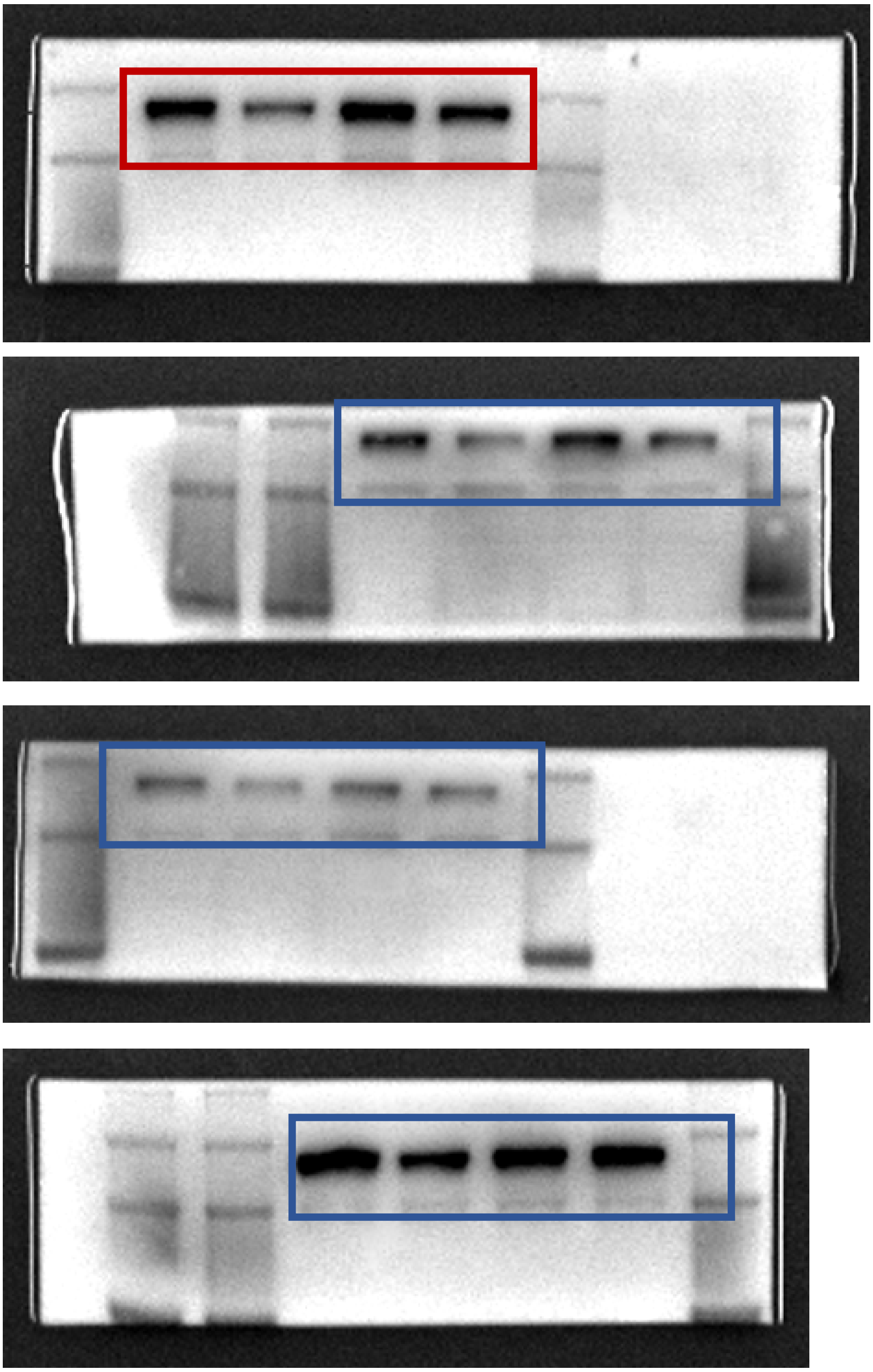

GAPDH (for VE-cadherin)

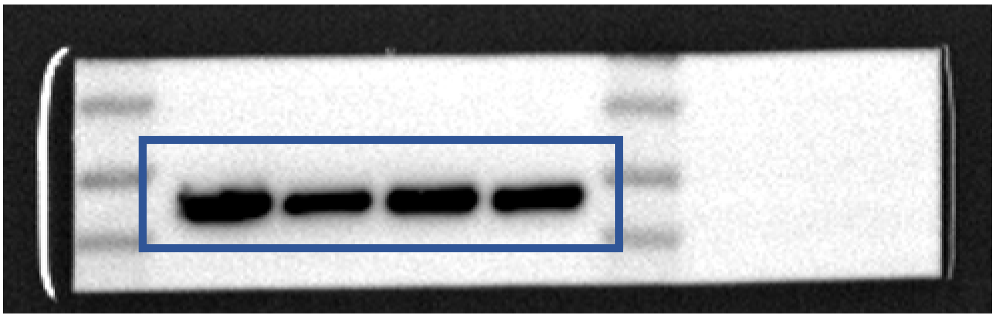

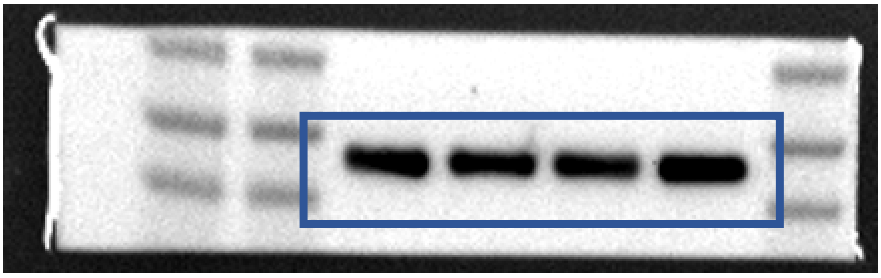

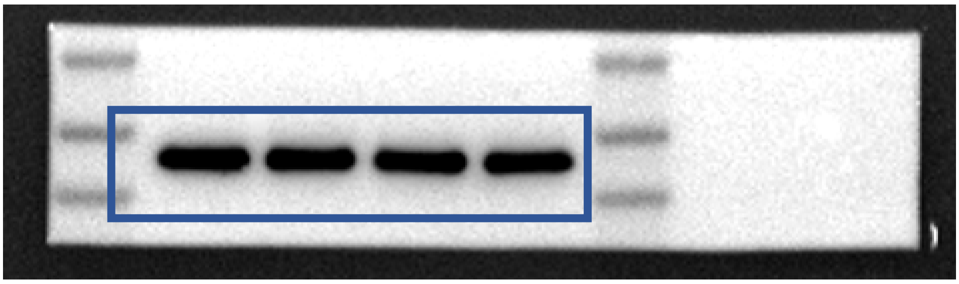

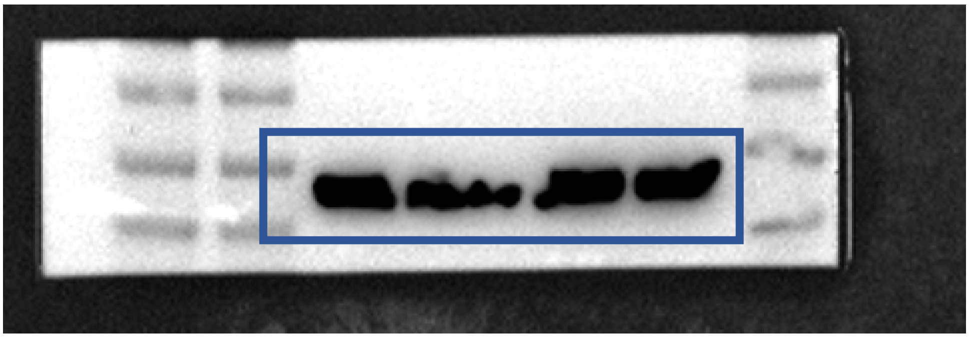

Figure 3E

Piezo1

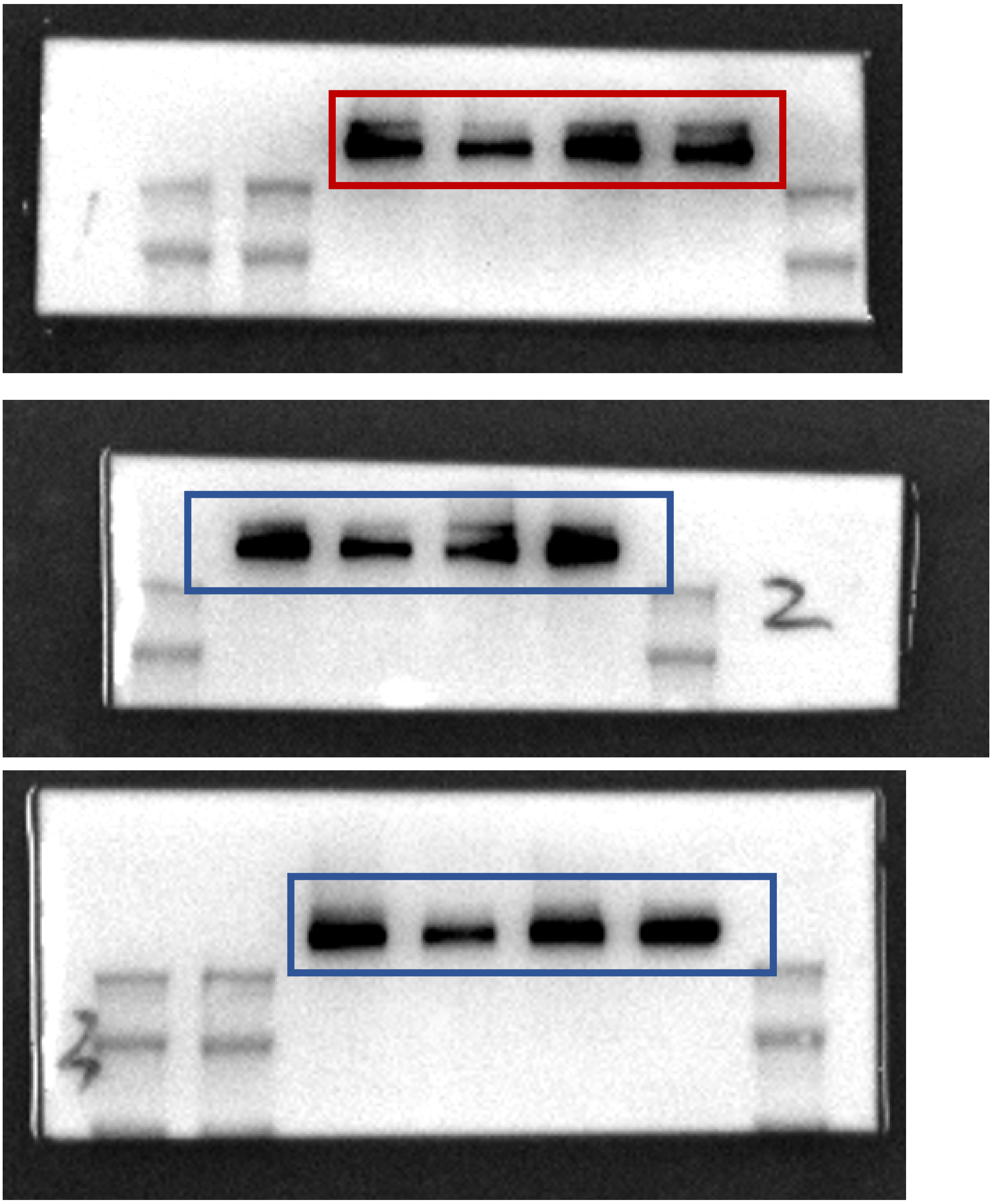

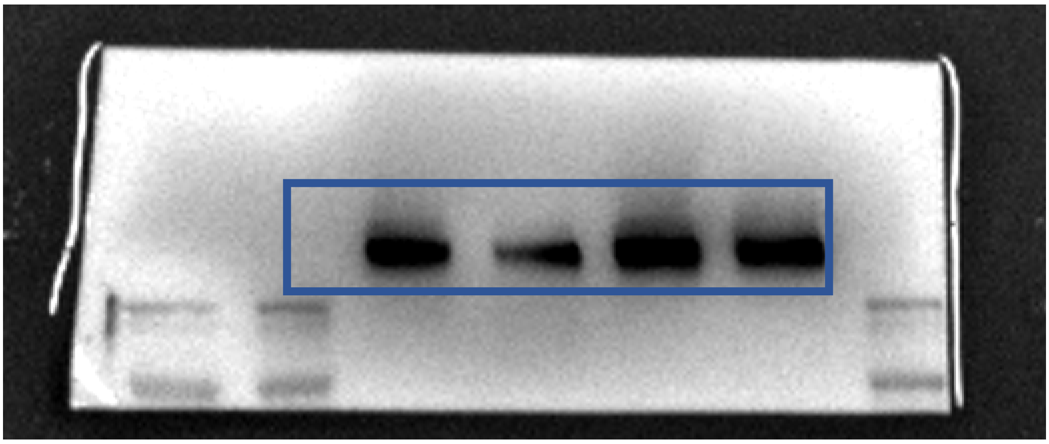

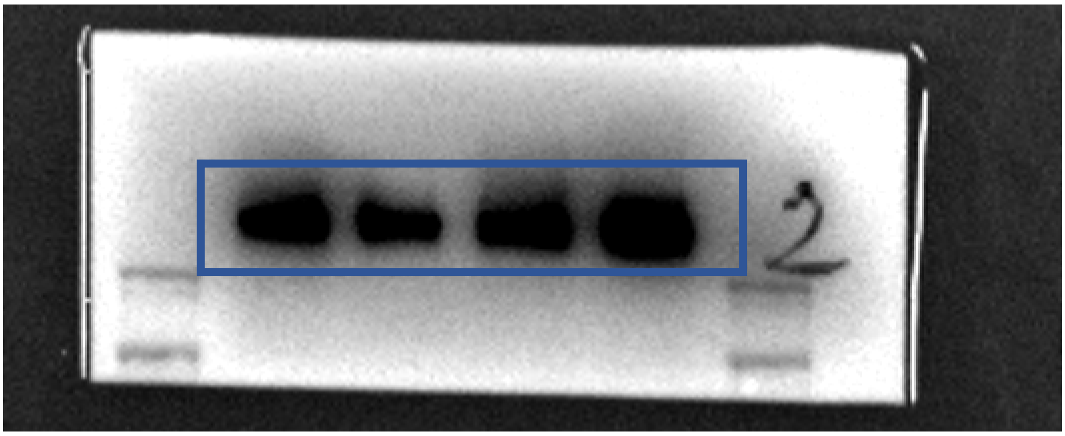

Piezo2

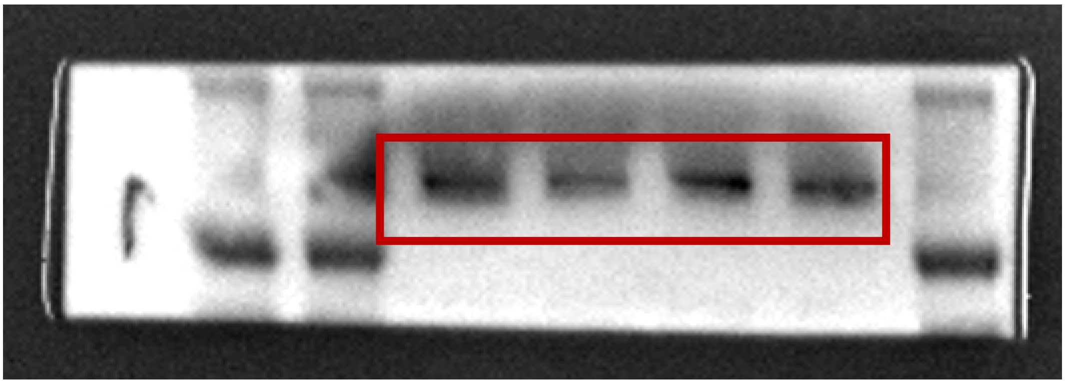

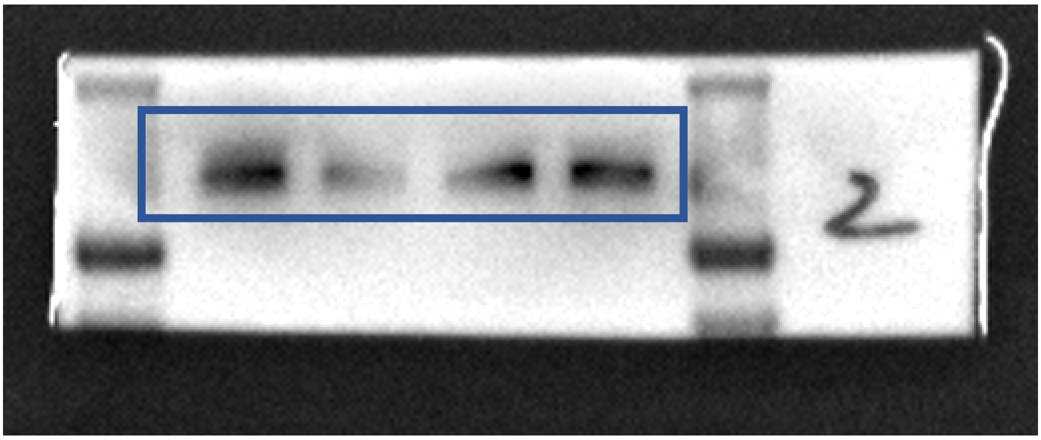

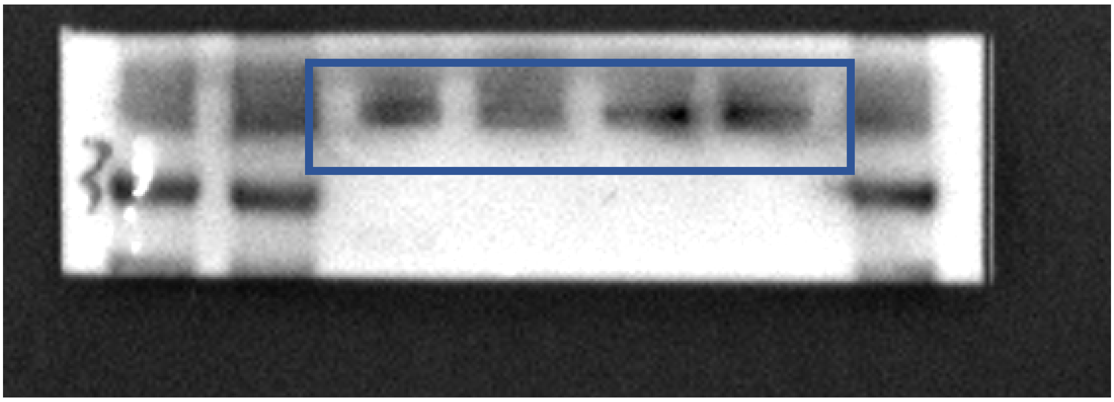

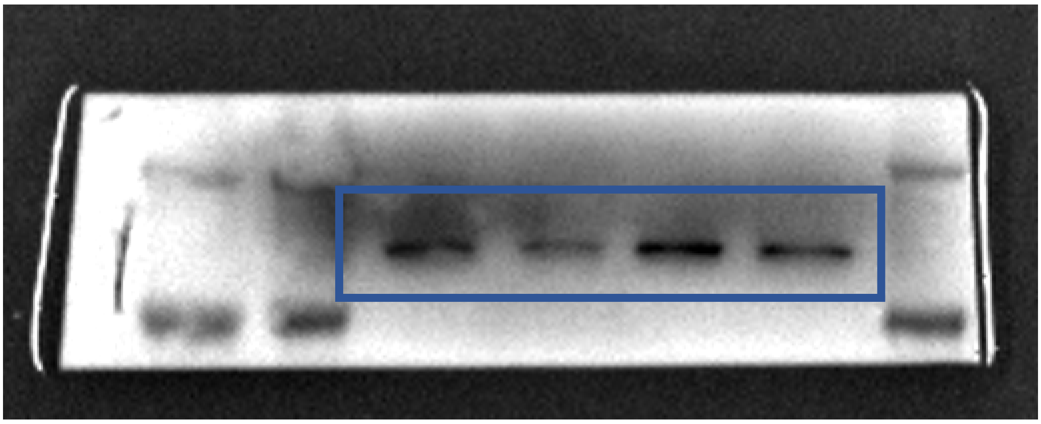

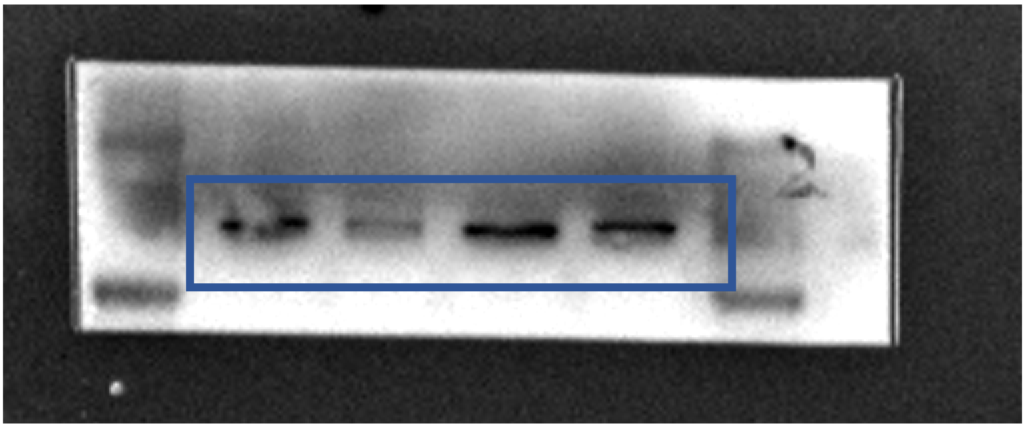

GAPDH

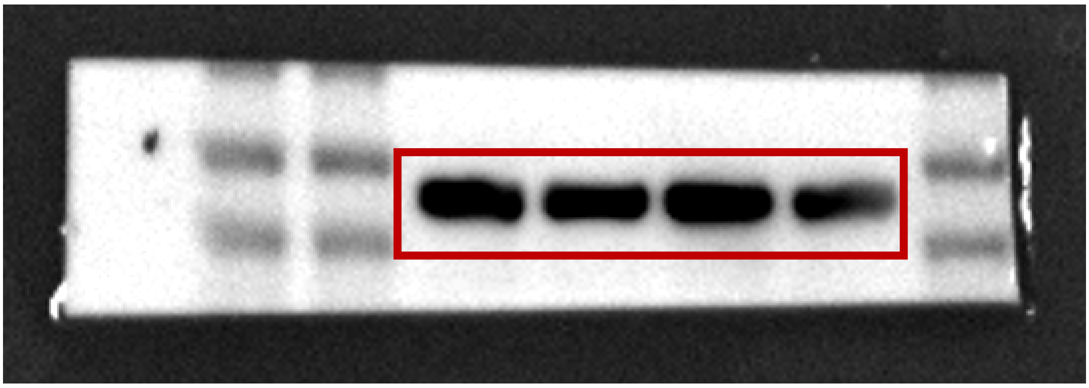

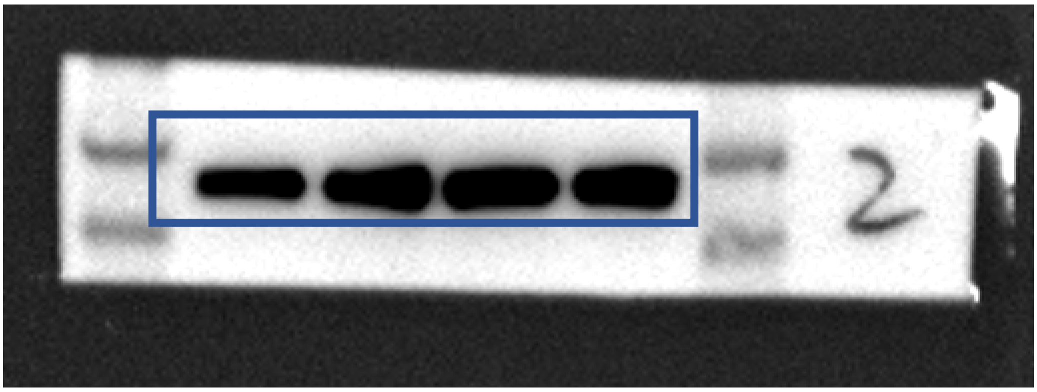
2

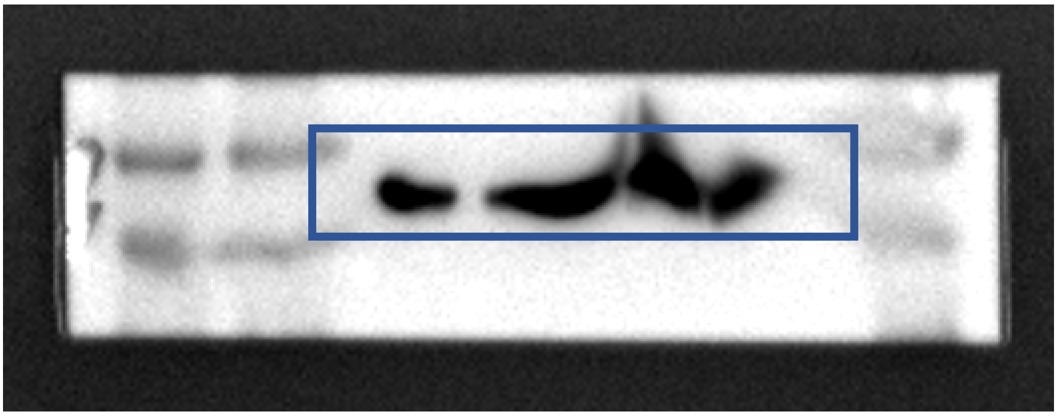

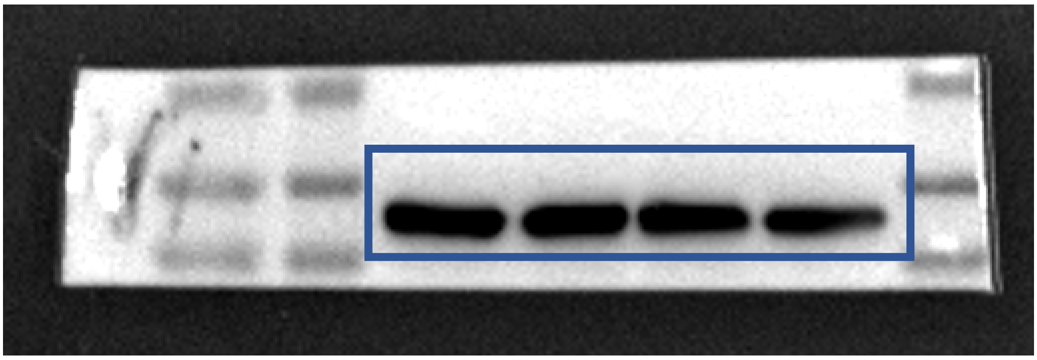

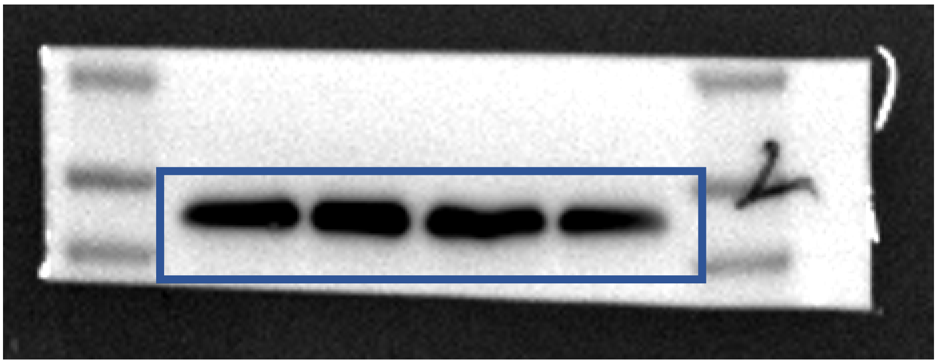

Figure 4C

Flag (FADS1)

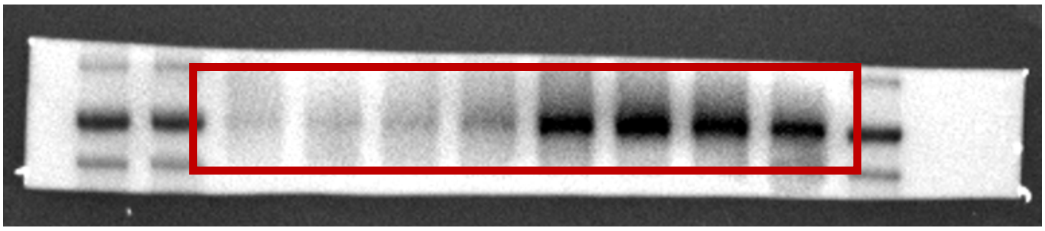

GAPDH

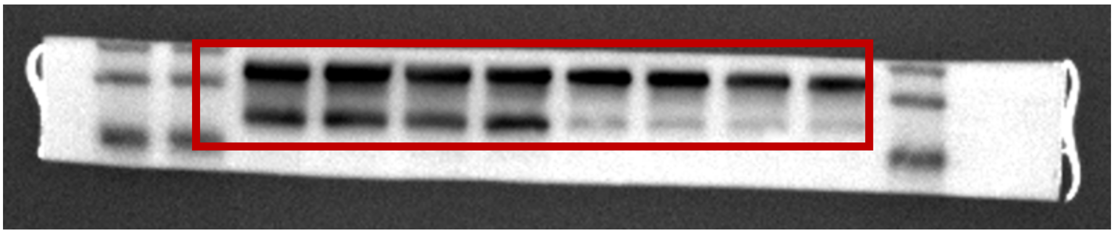

Flag (FADS2)

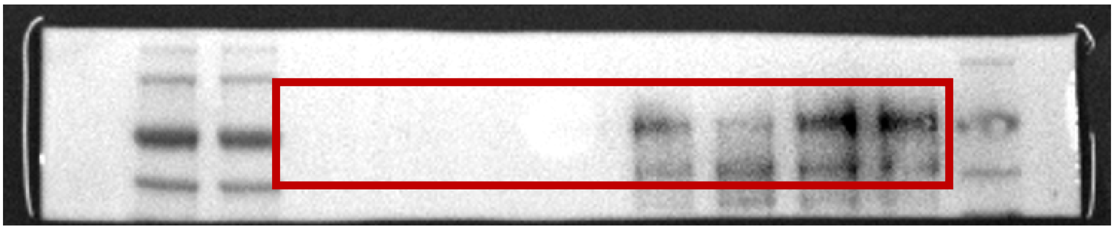

GAPDH

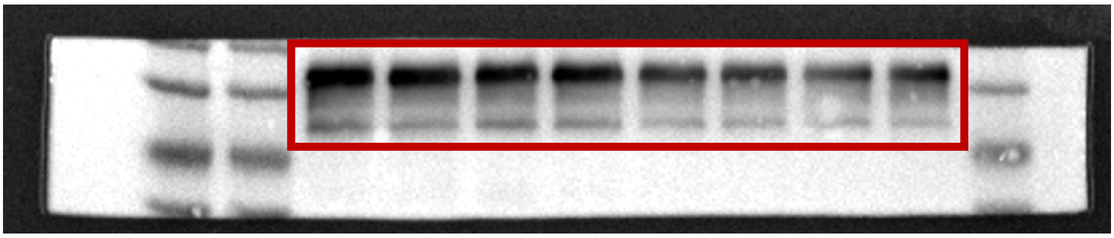

Figure 5C

FADS1

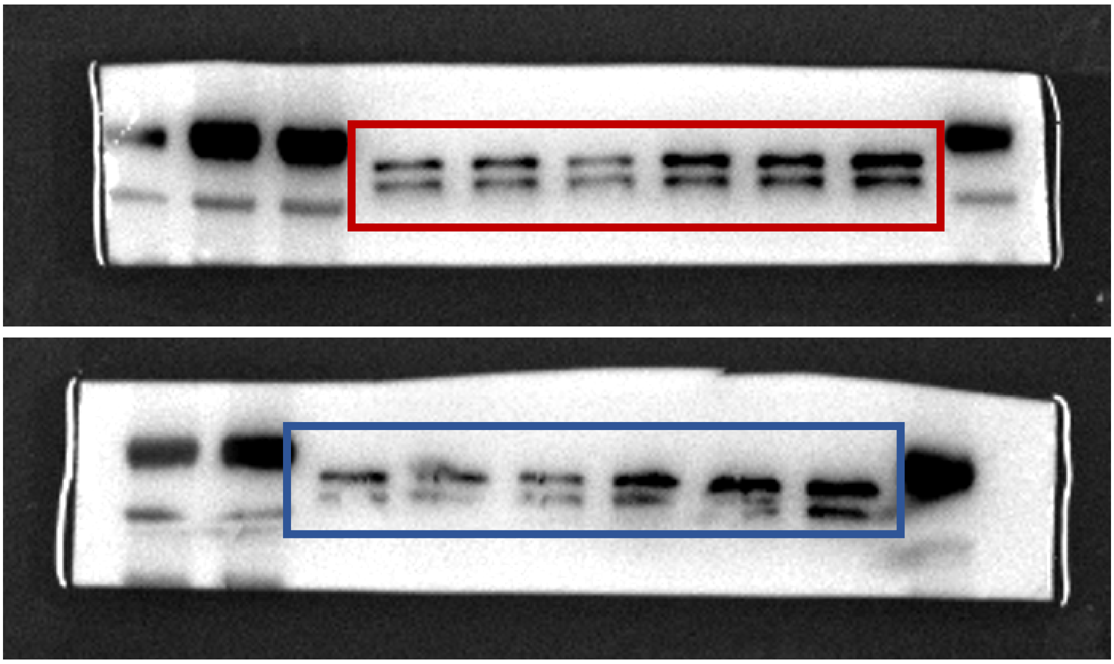

FADS2

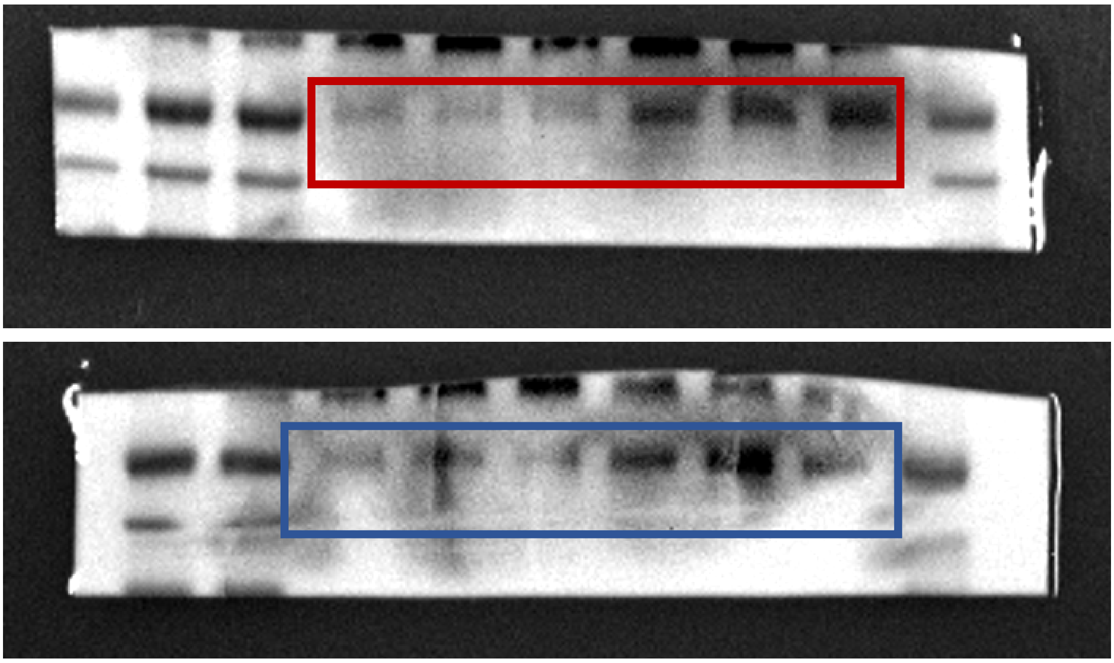

GAPDH

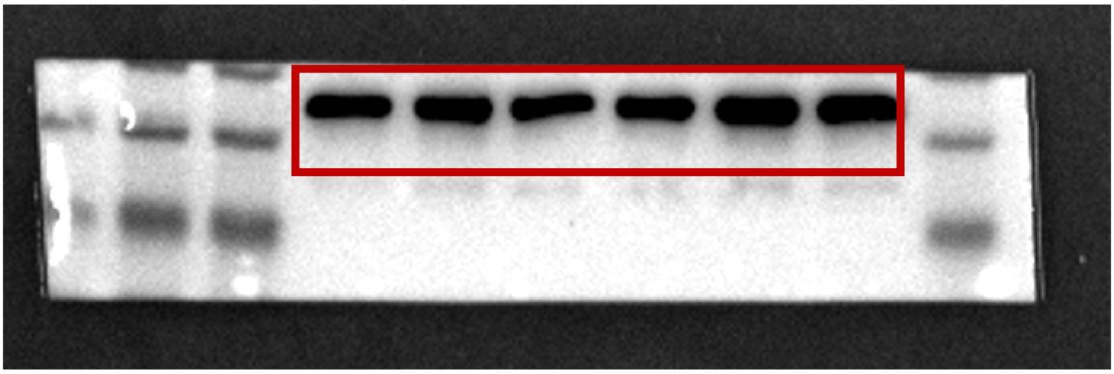

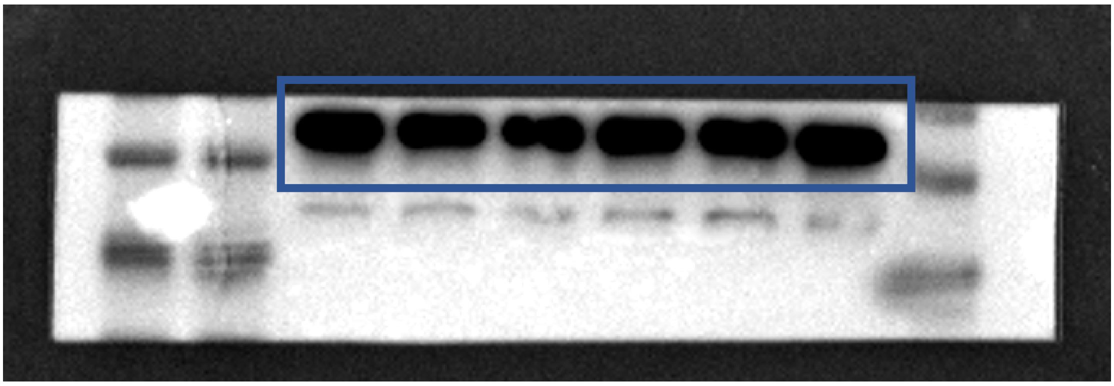

Figure 5S

ZO-1

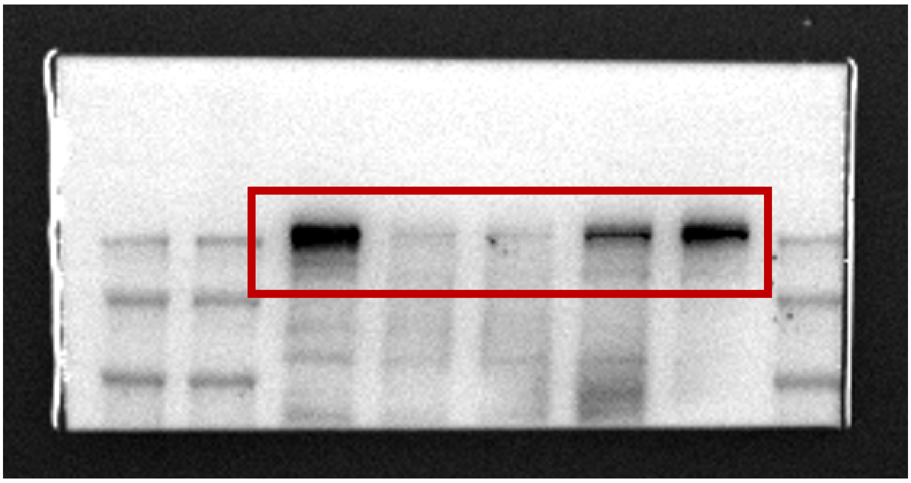

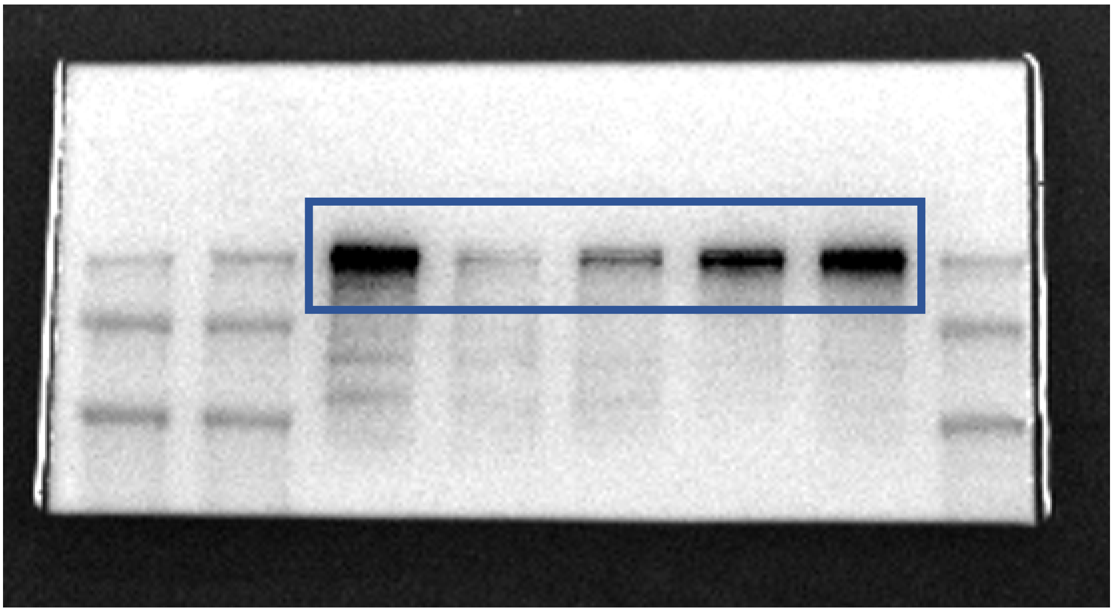

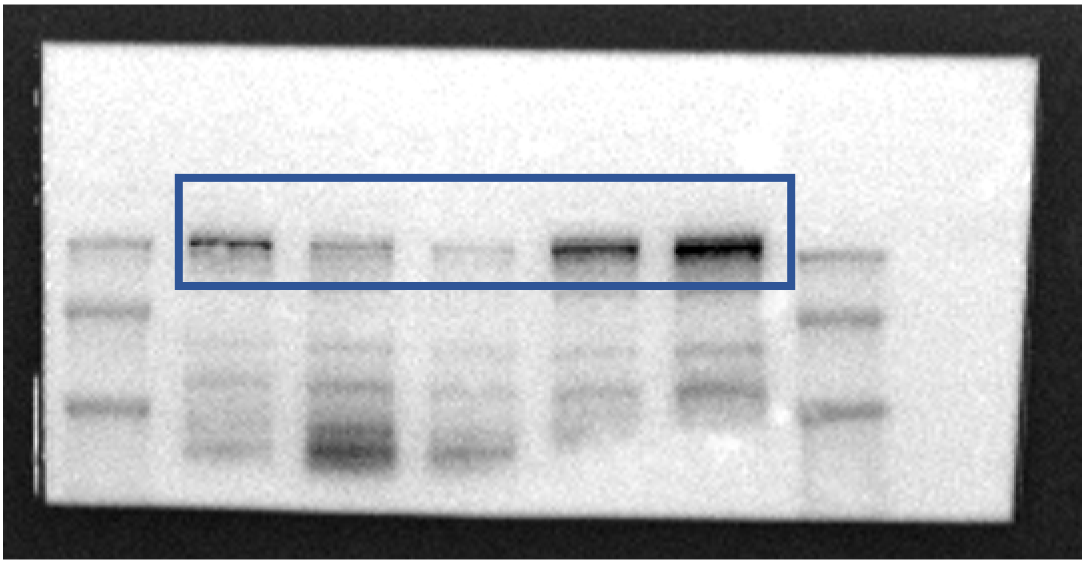

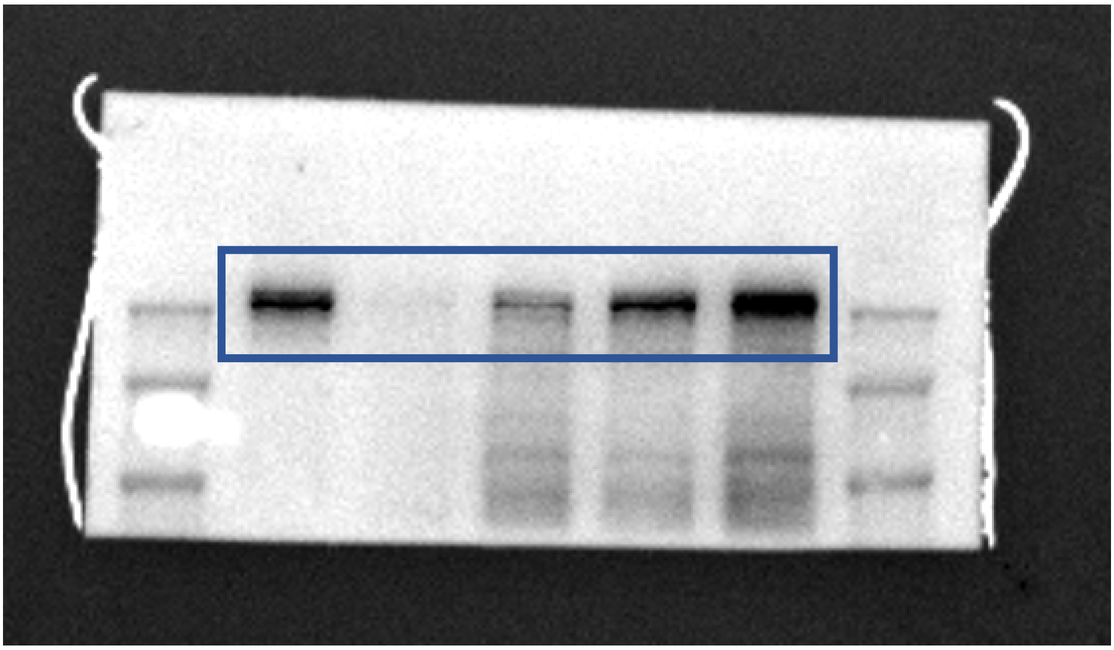

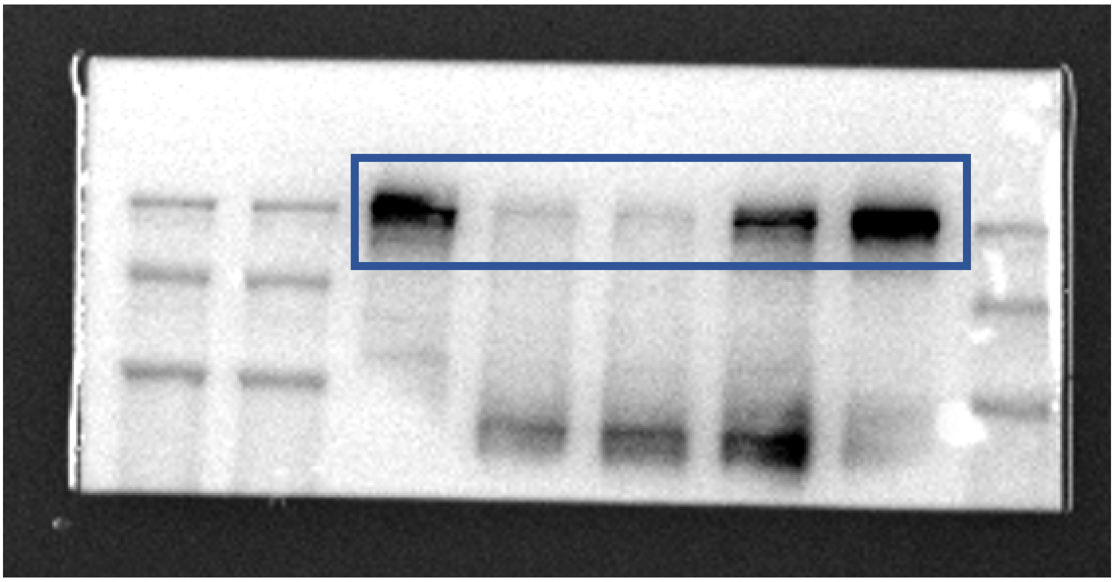

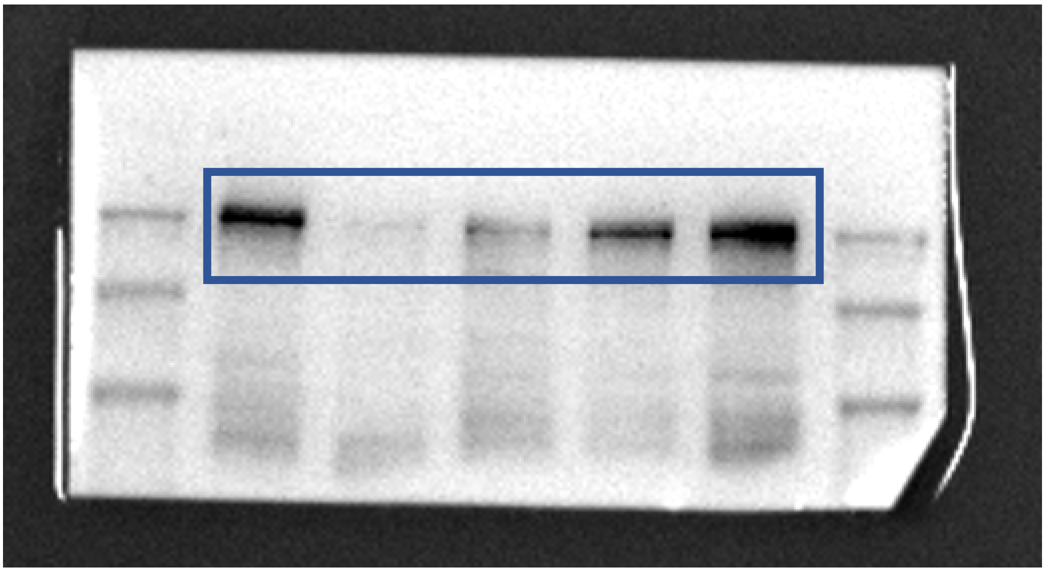

GAPDH (for ZO-1)

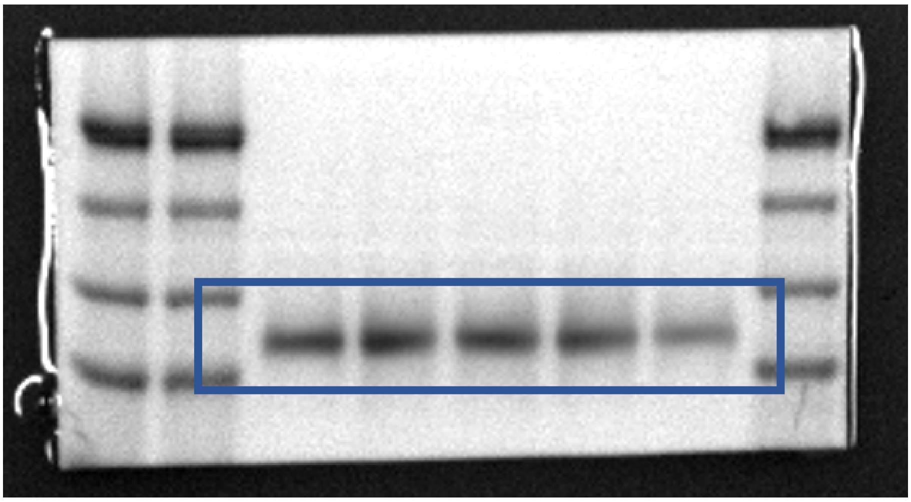

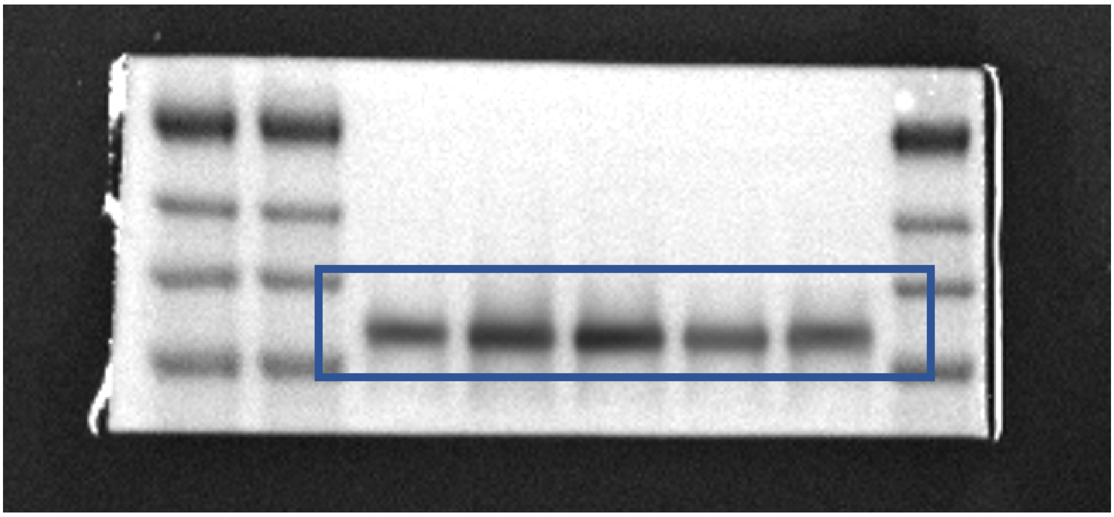

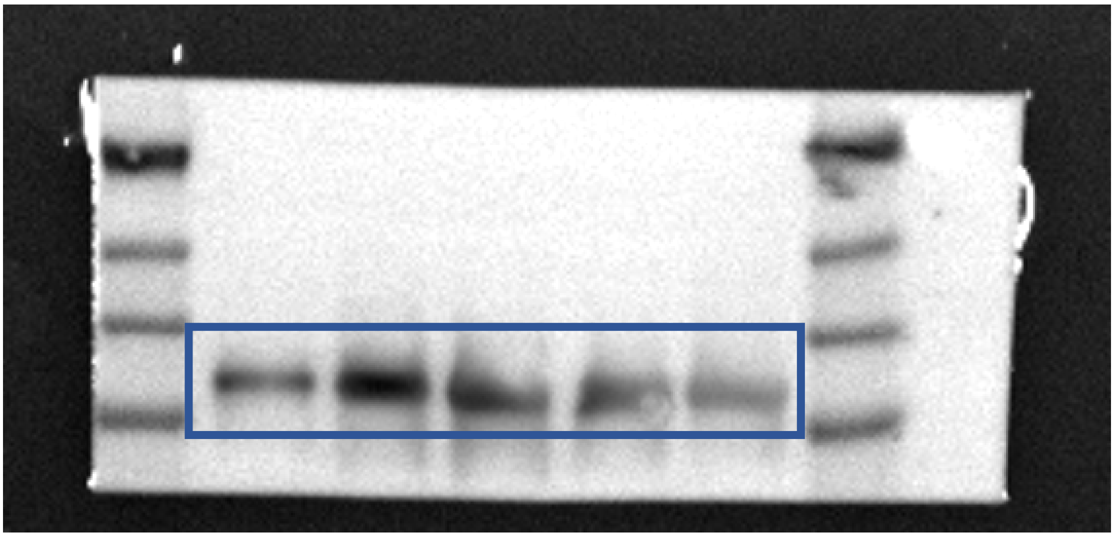

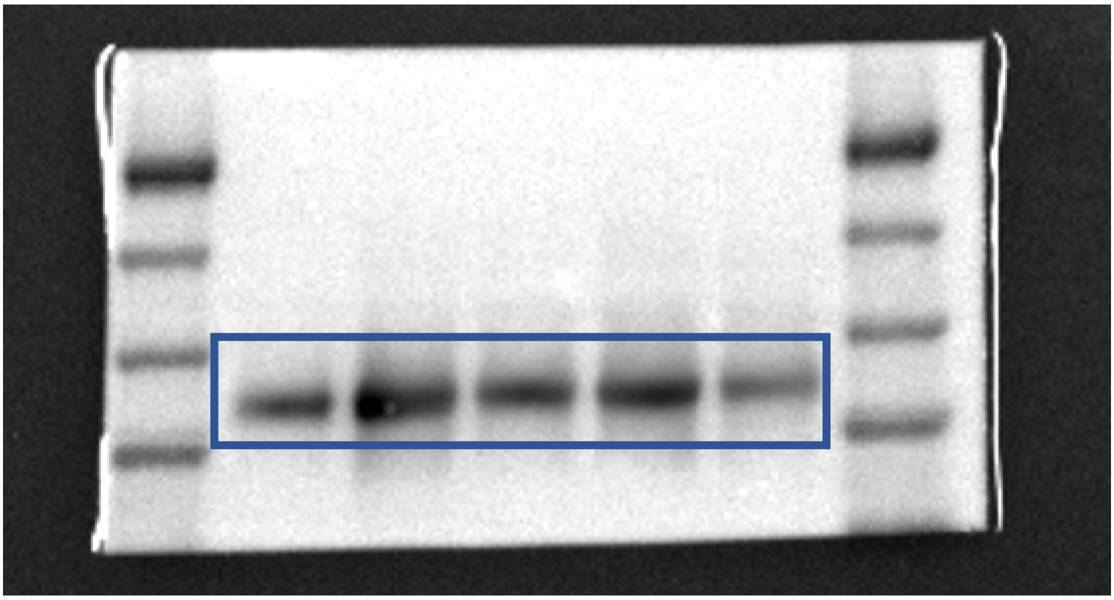

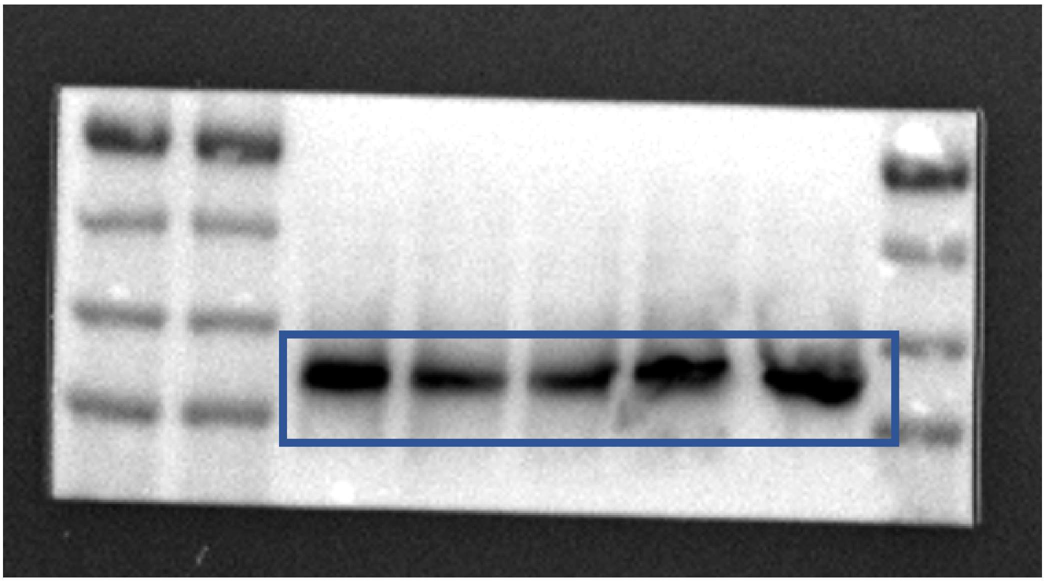

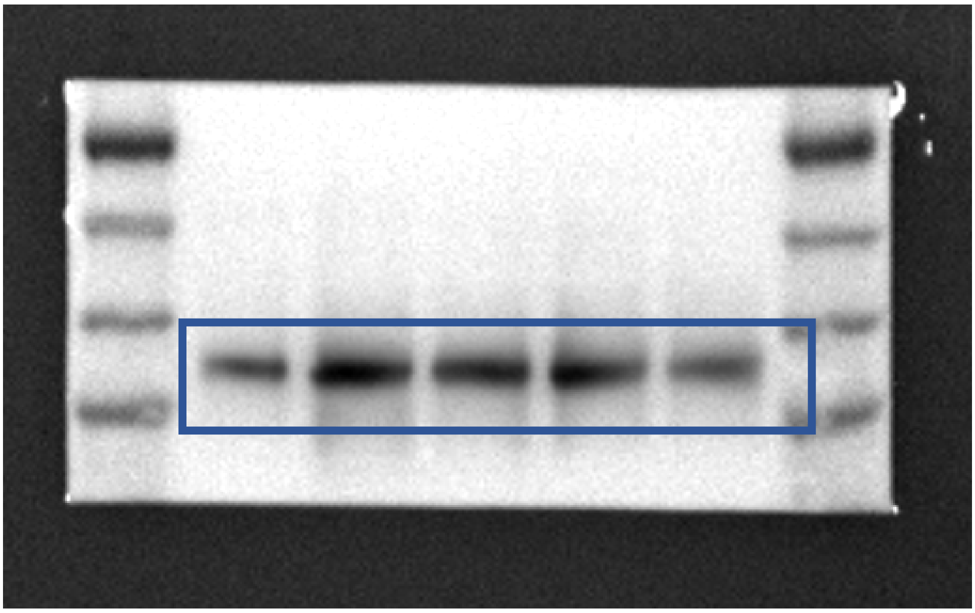

VE-cadherin

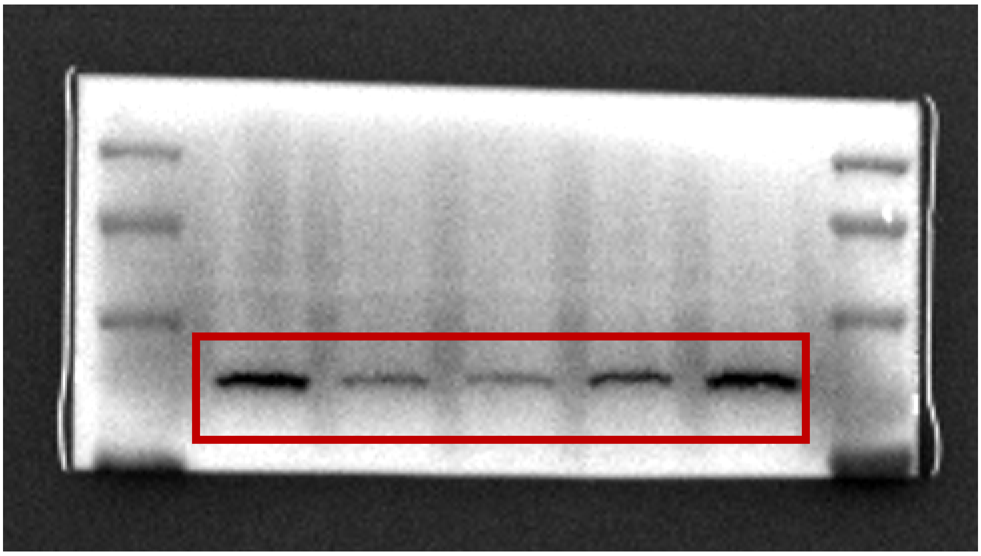

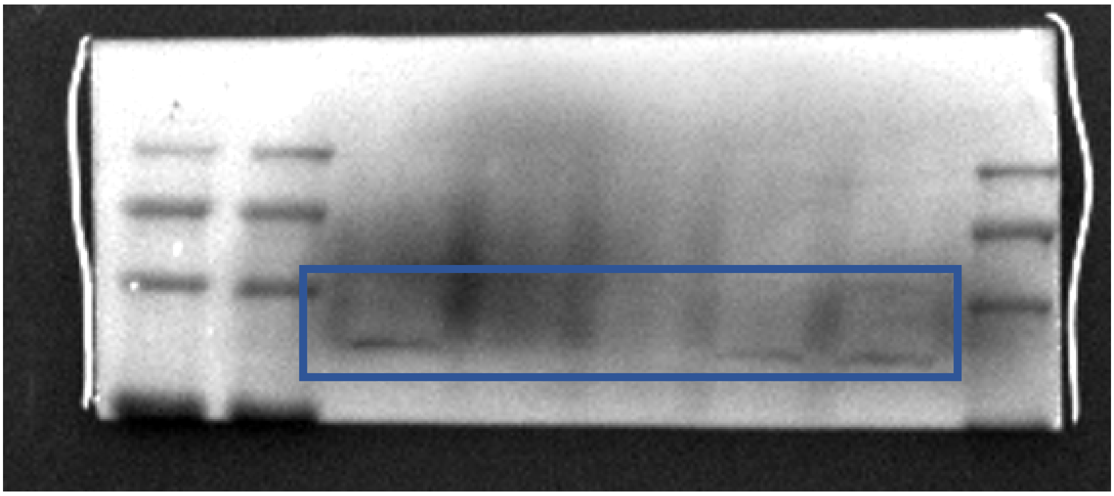

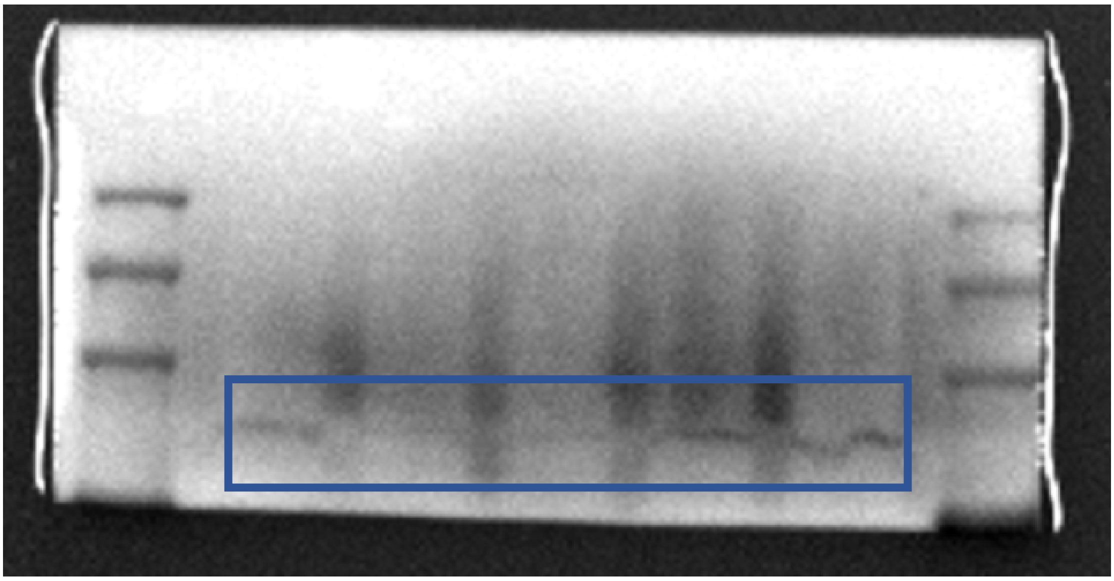

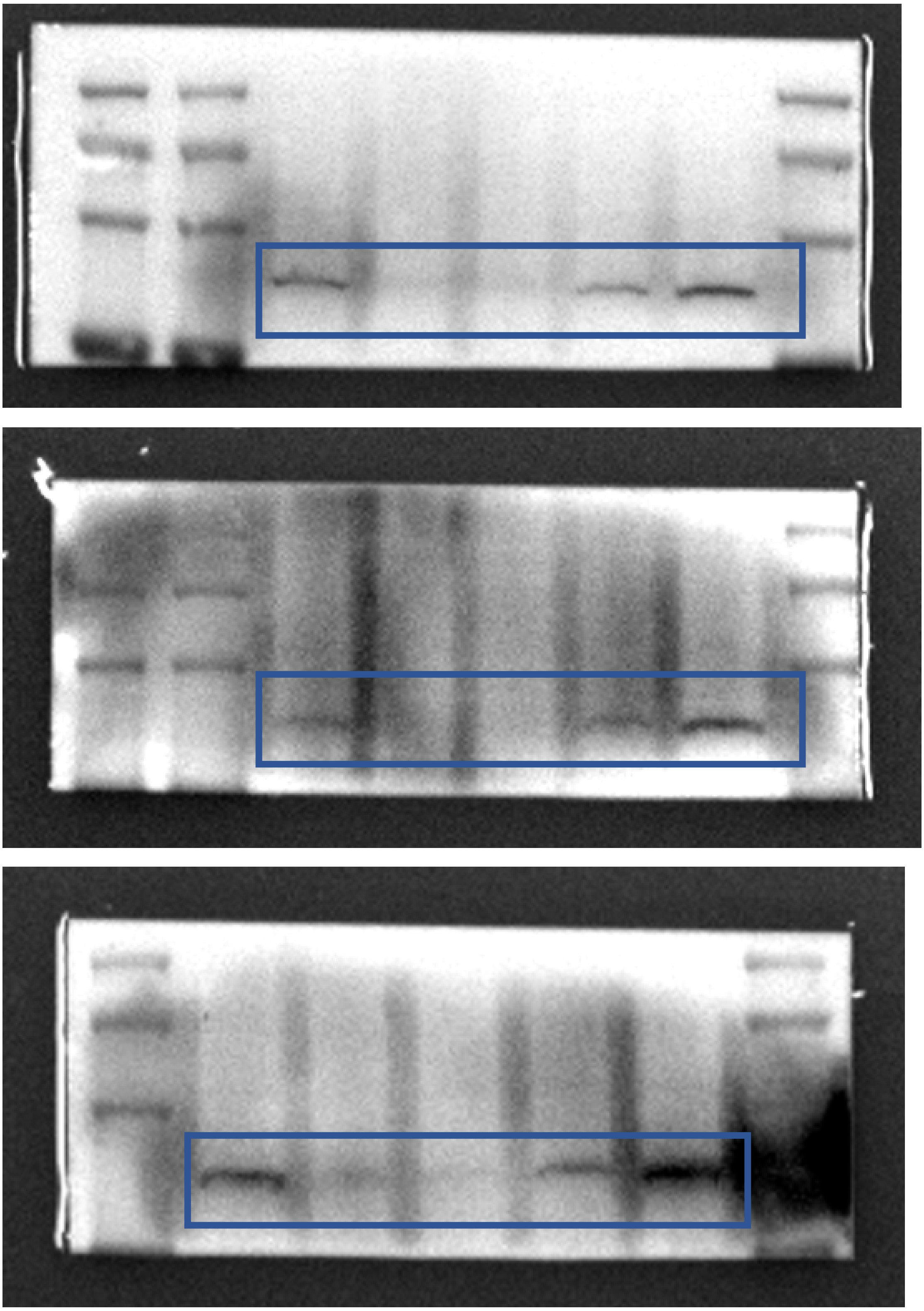

GPX4

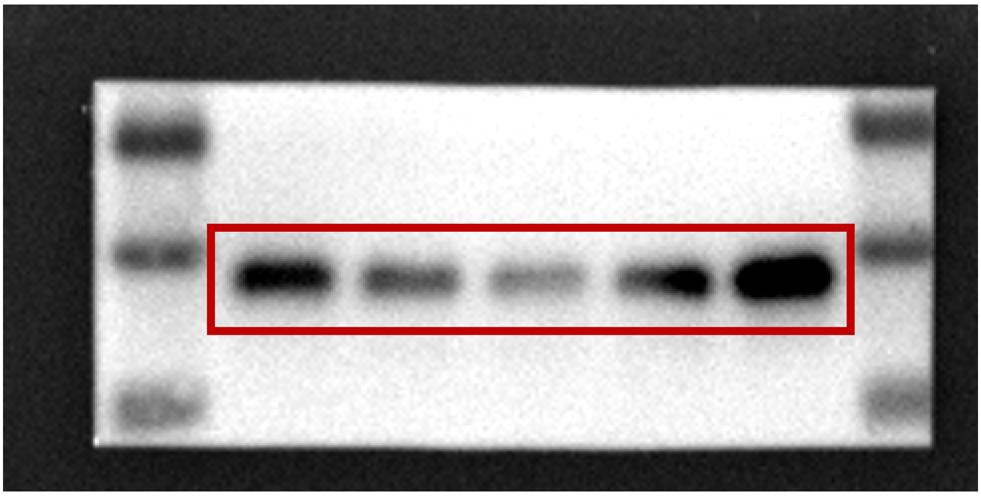

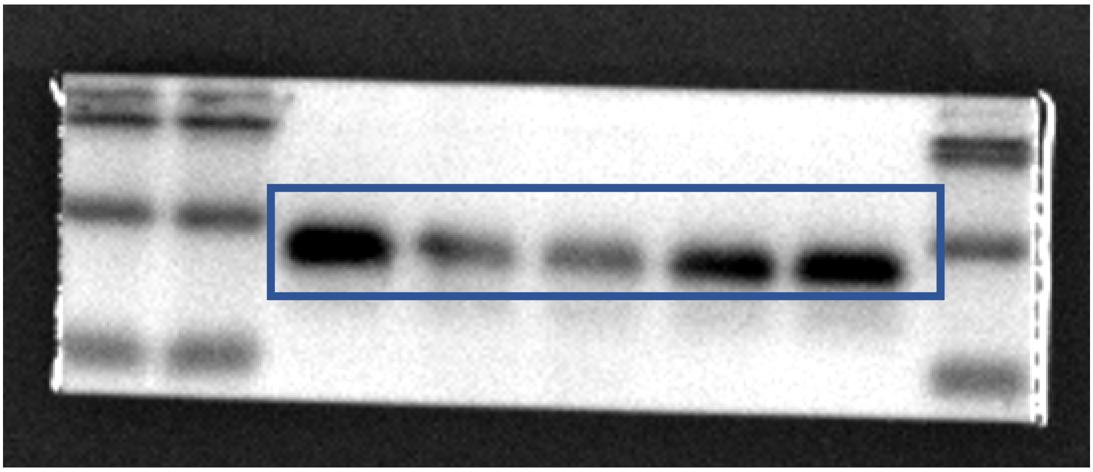

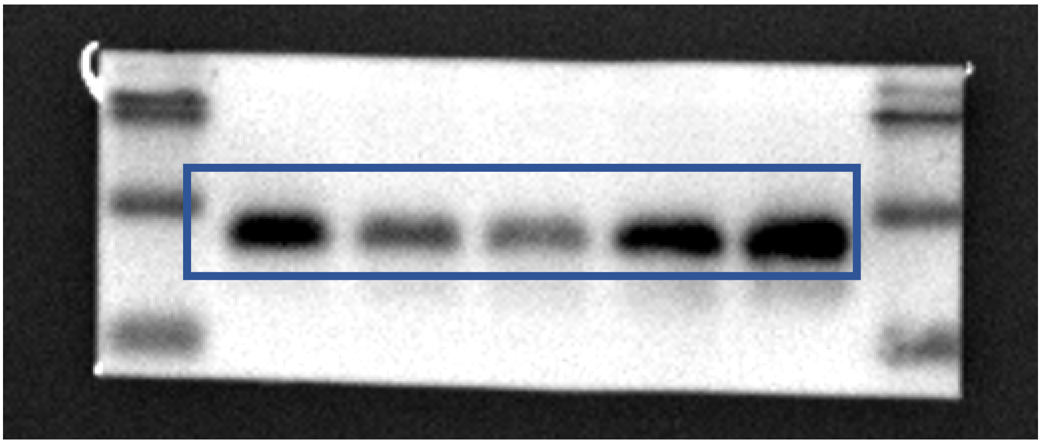

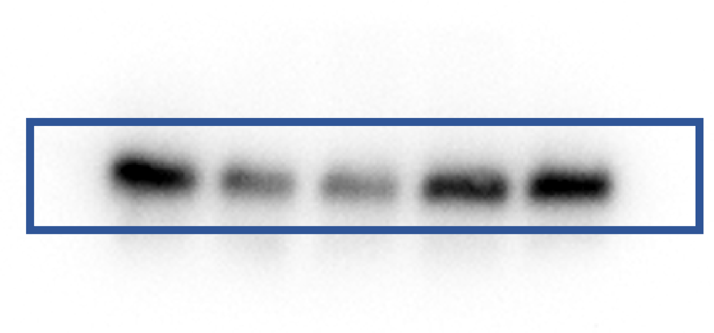

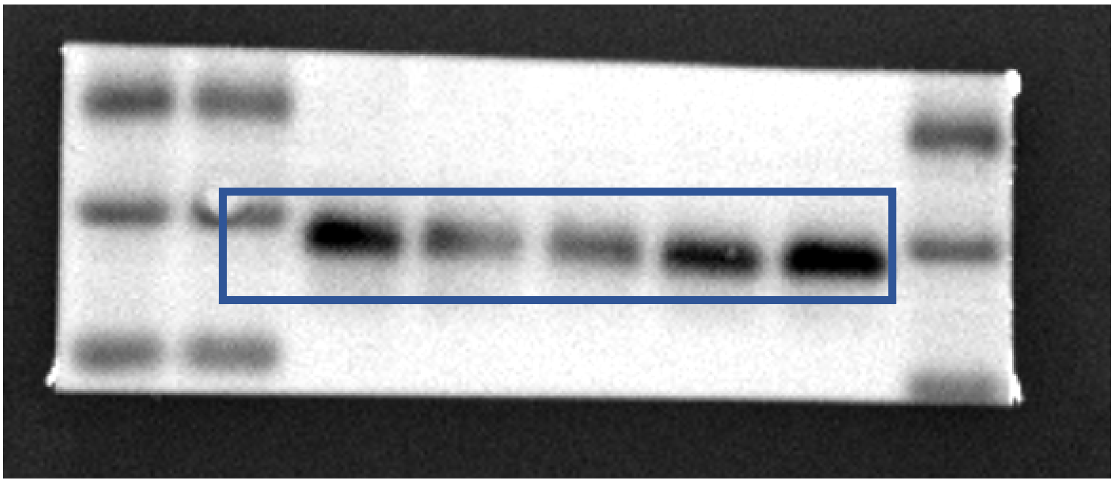

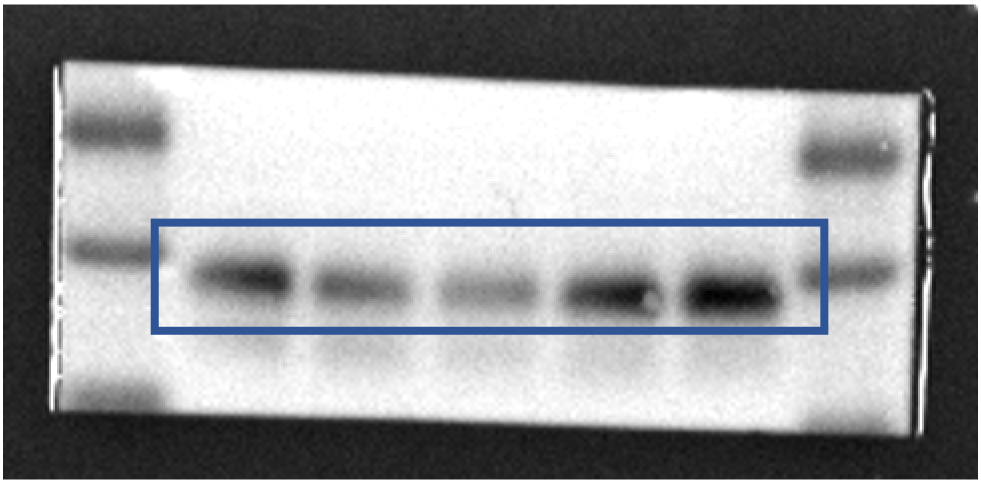

GAPDH

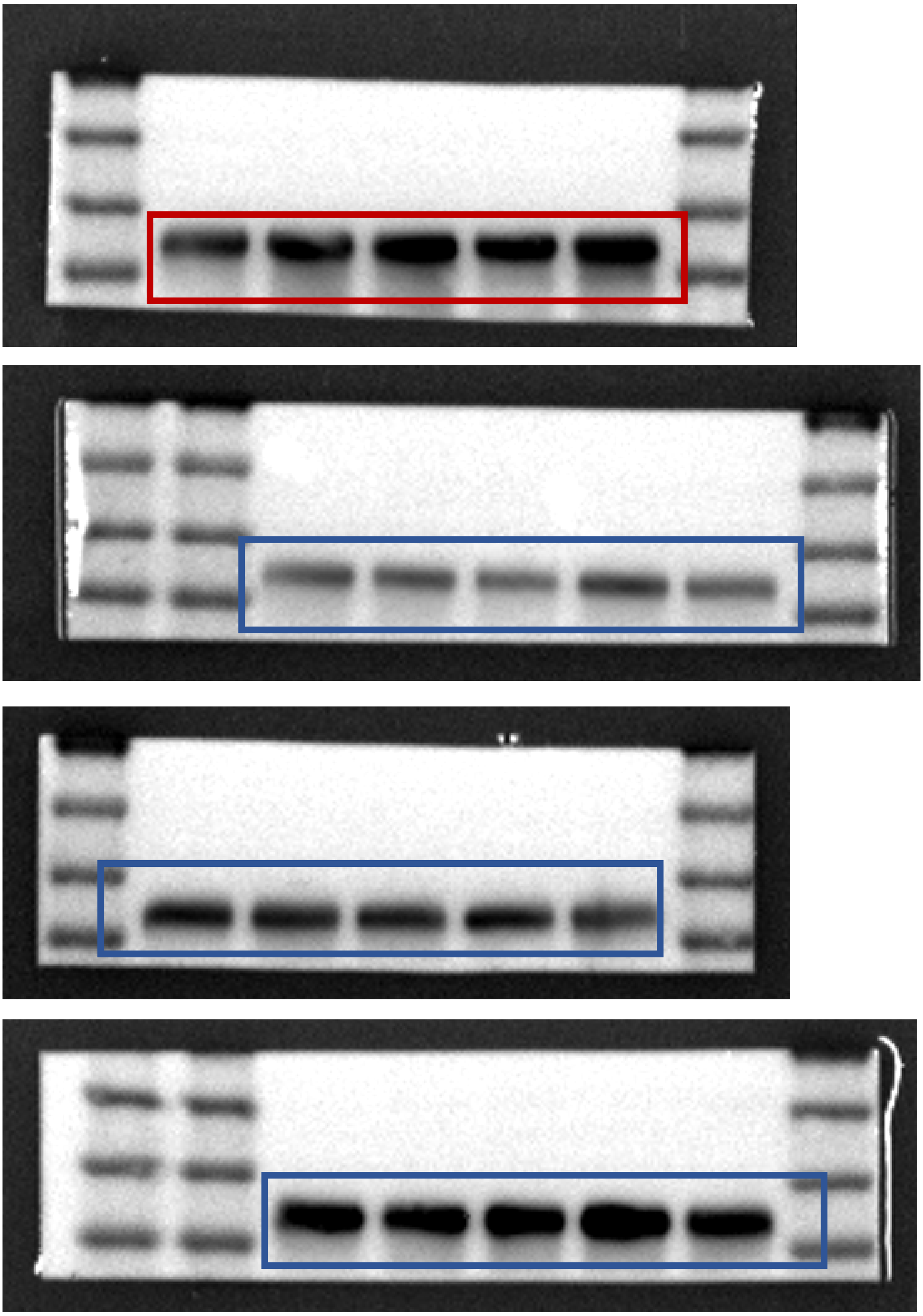

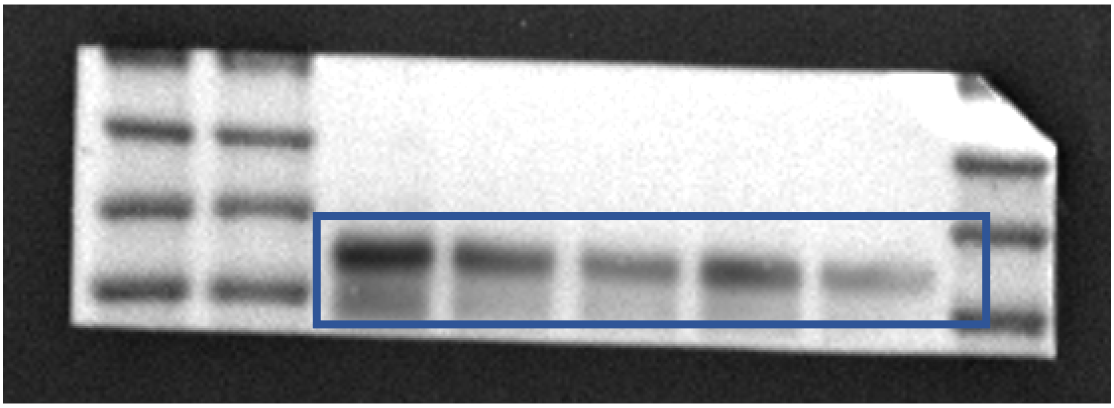

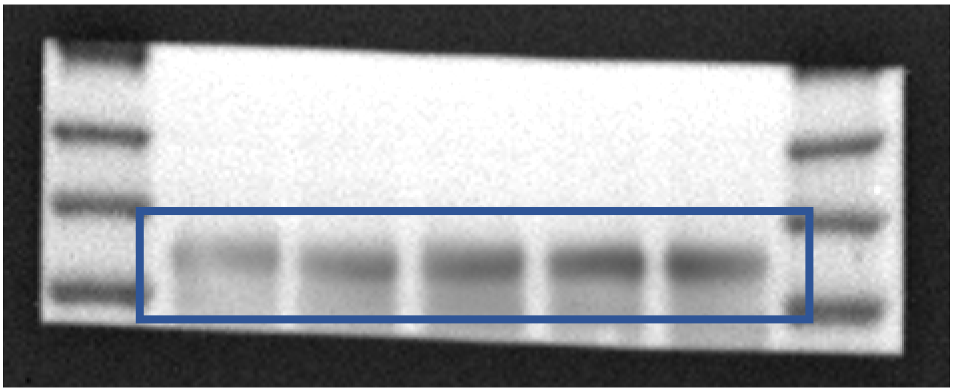

Figure 6B

PARK7

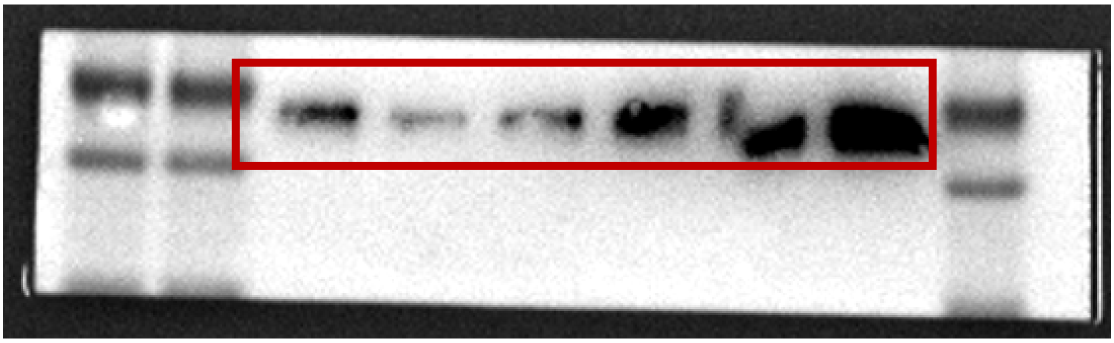

GAPDH

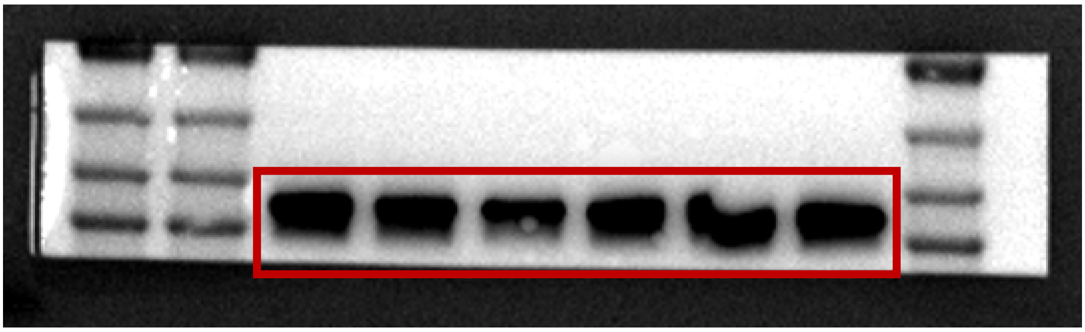

Figure 6C

PARK7

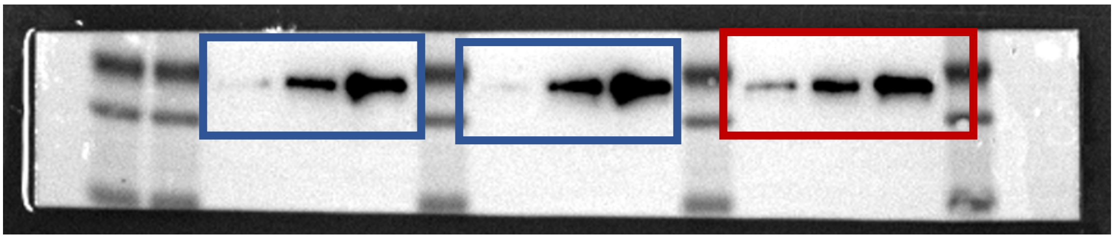

GAPDH

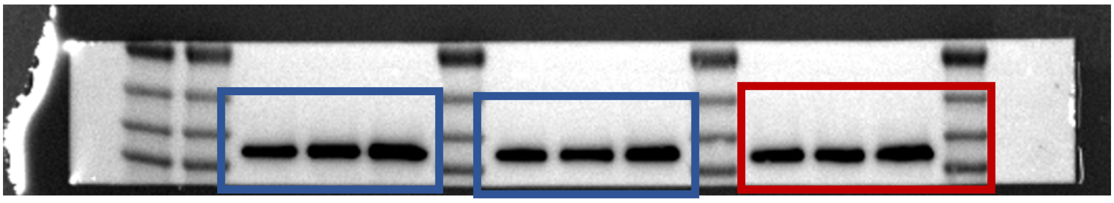

Figure 6M

ZO-1

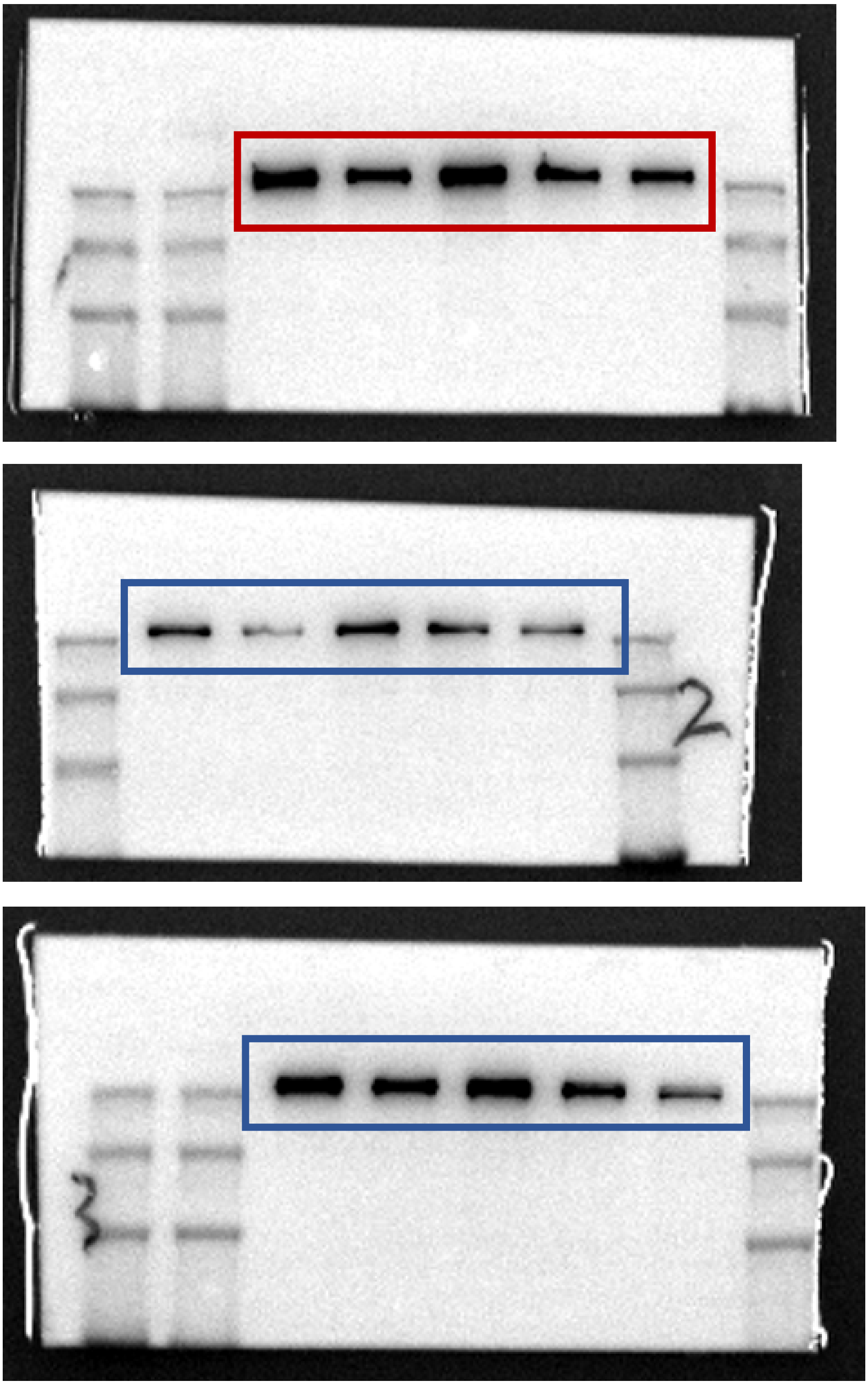

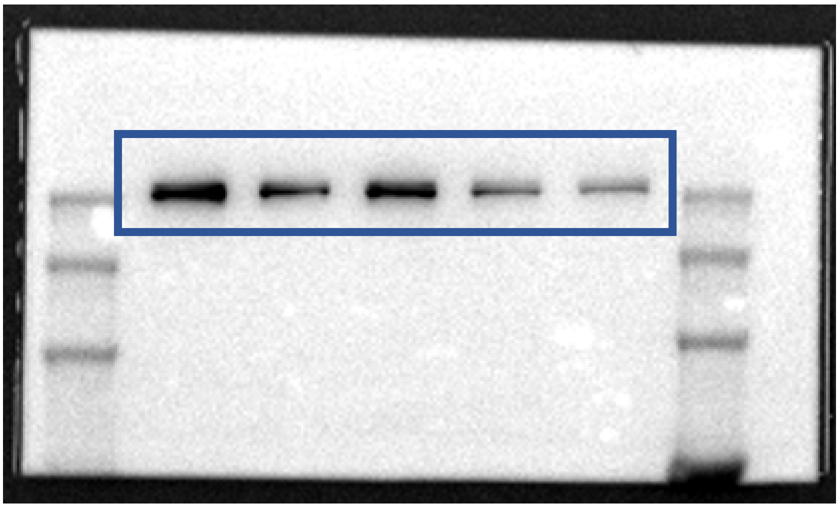

GAPDH (for ZO-1)

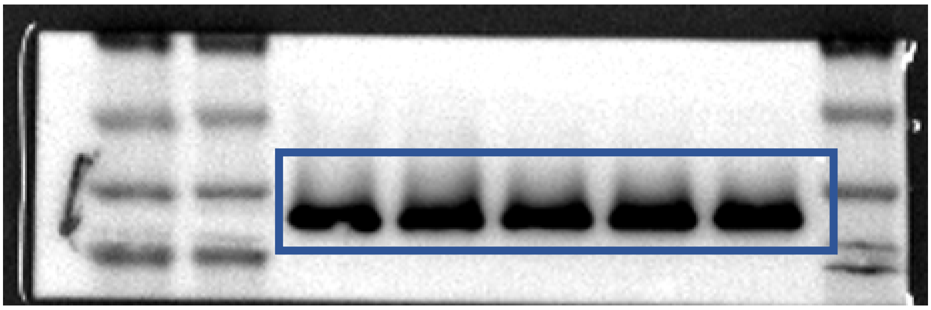

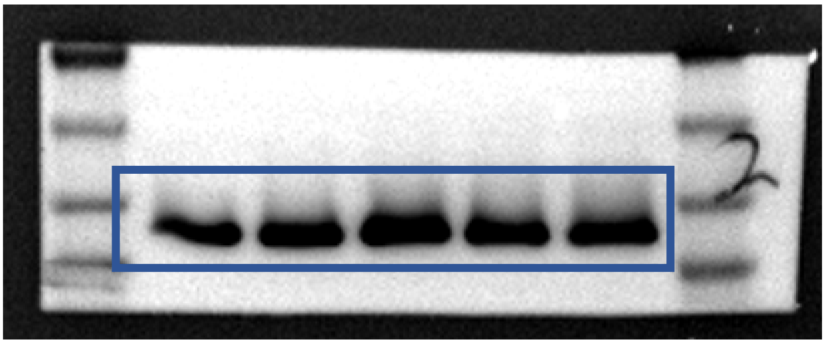

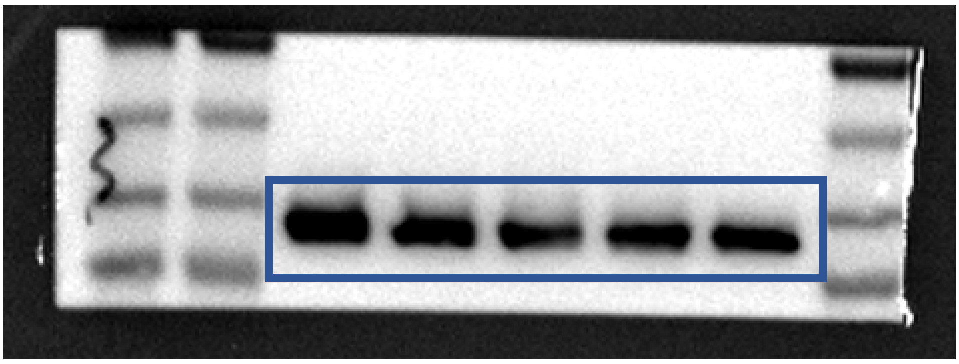

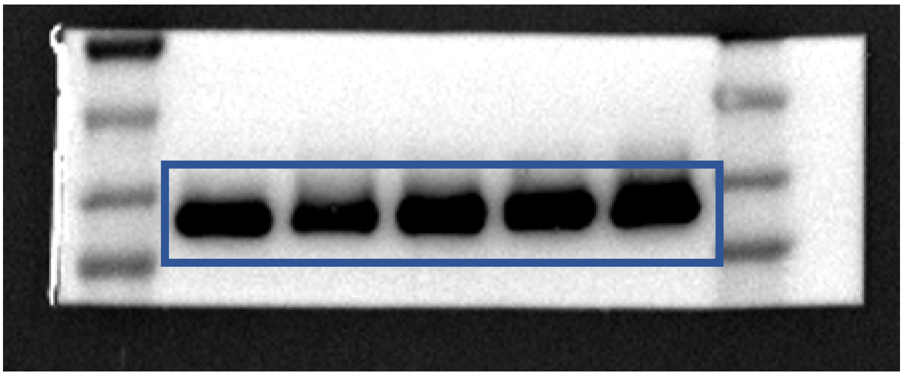

VE-cadherin

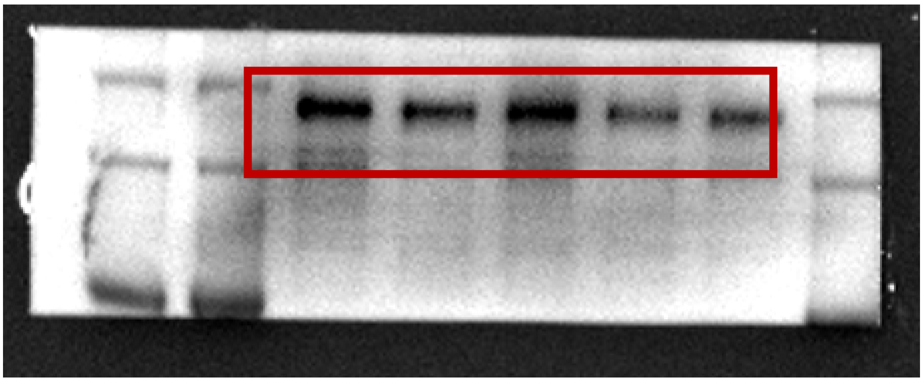

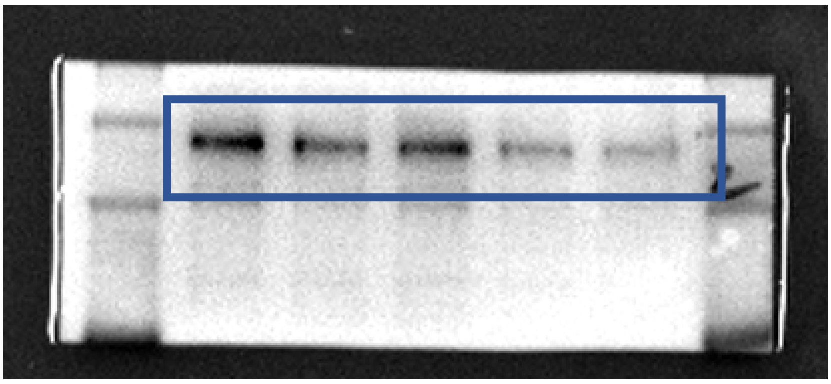

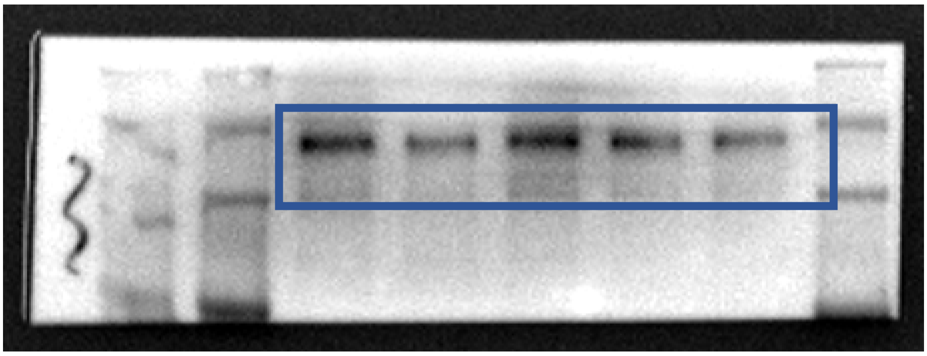

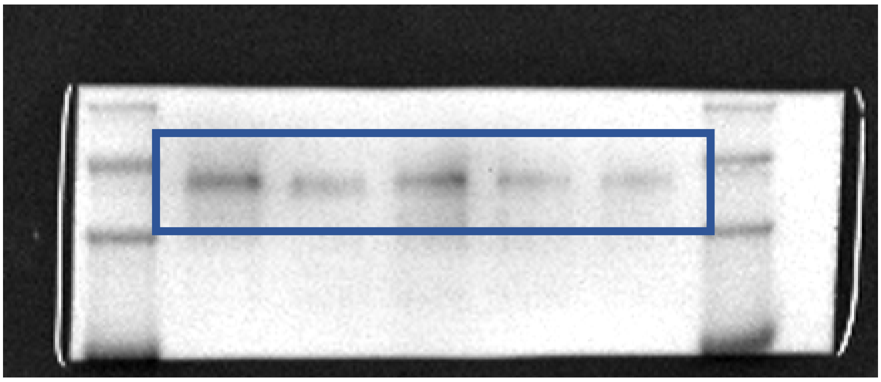

GPX4

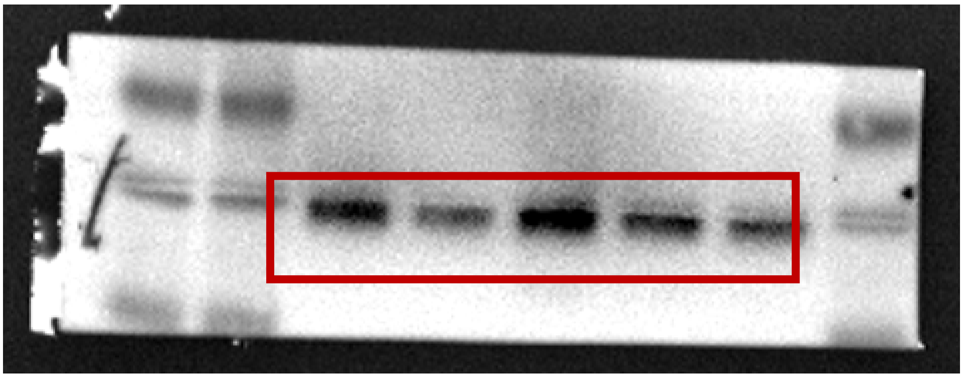

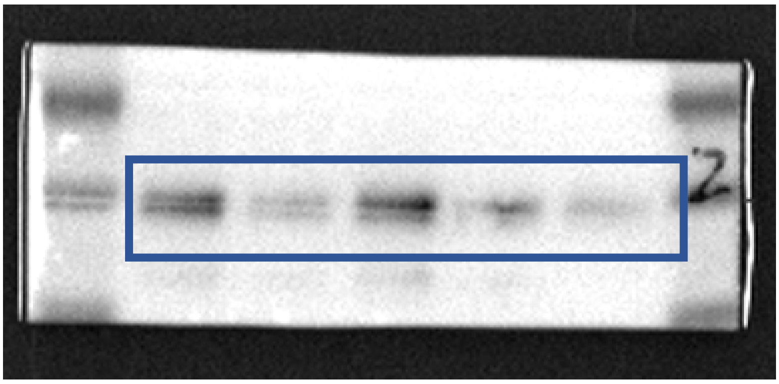

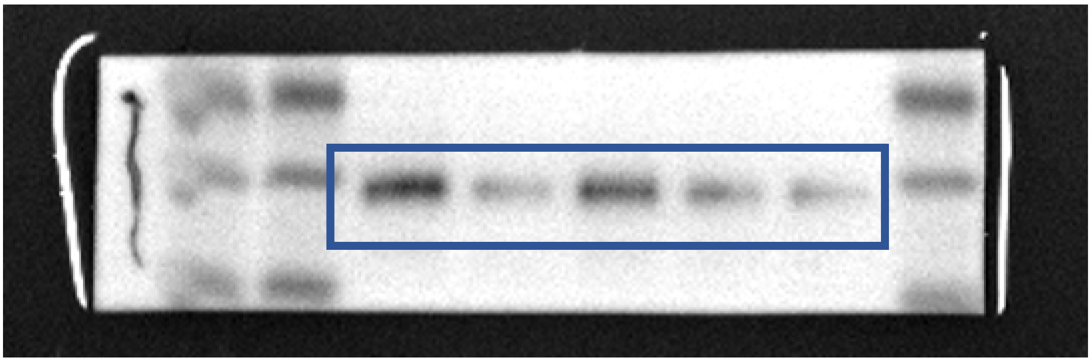

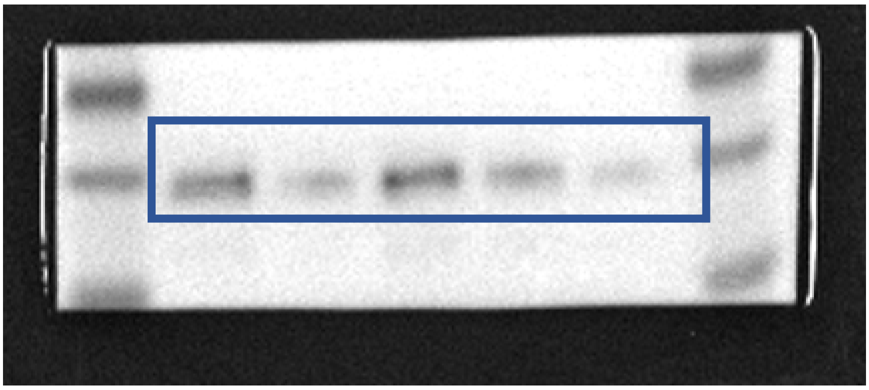

GAPDH (for VE-cadherin & GPX4)

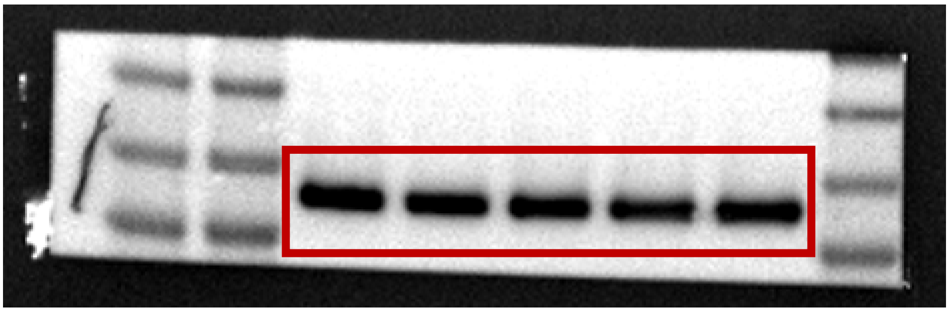

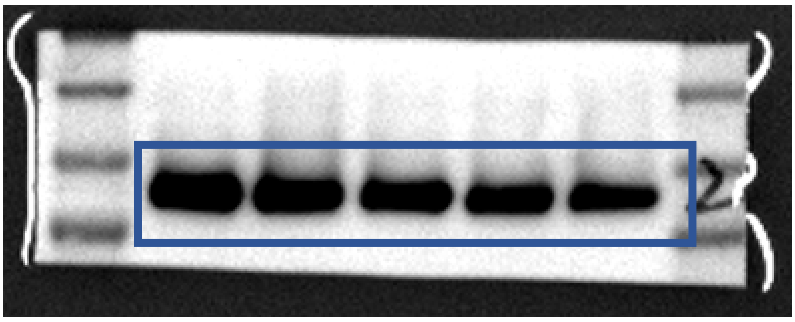

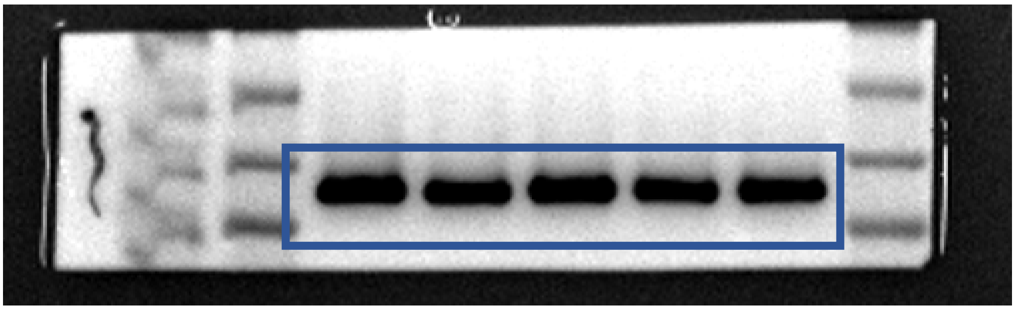

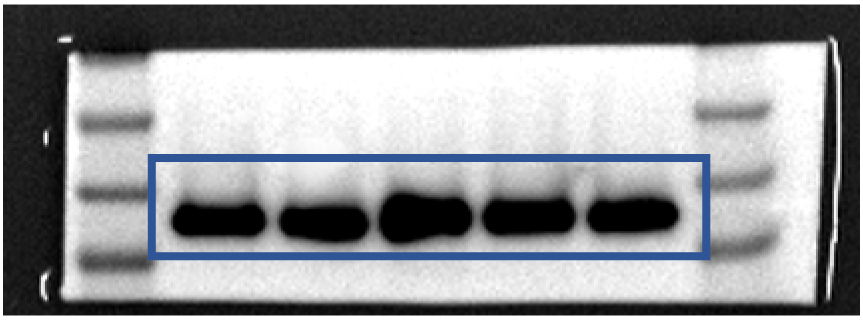

Figure S4C&D

FADS1 validation

CD31

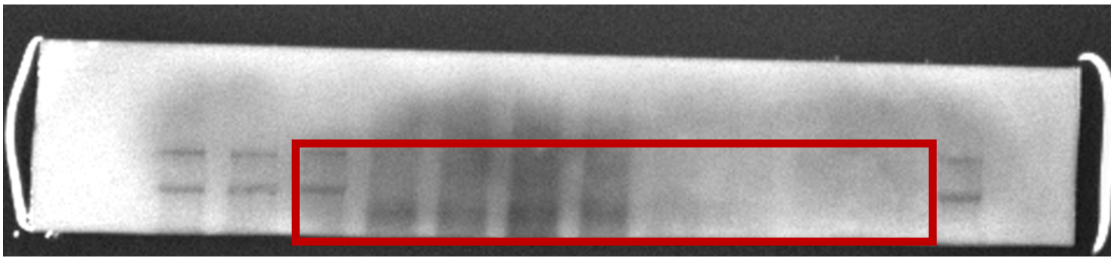

Flag

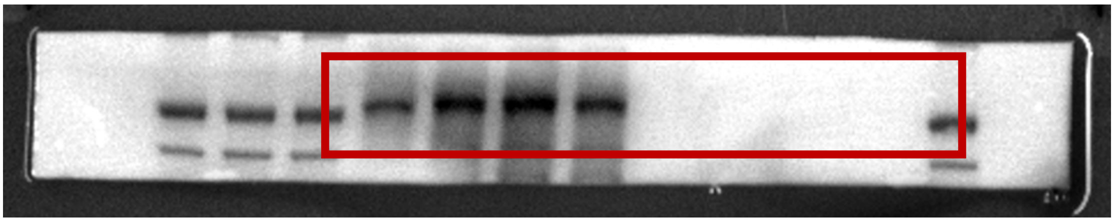

GAPDH

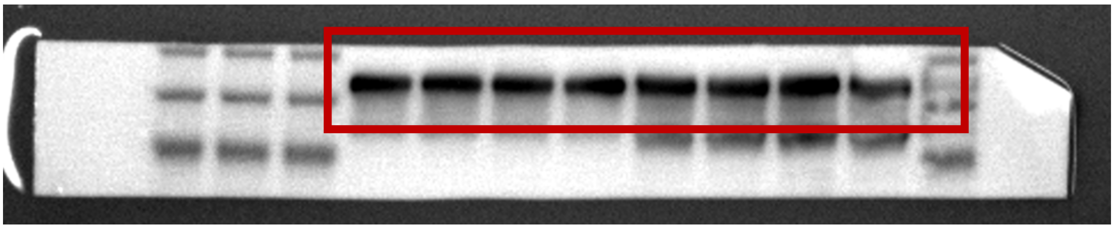

FADS2 validation

CD31

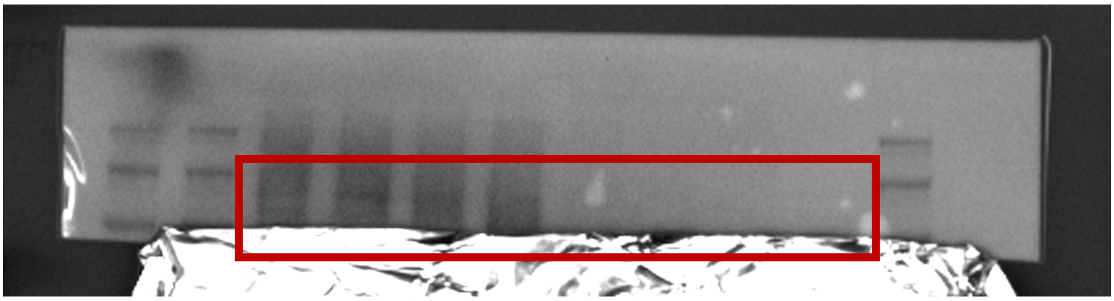

Flag

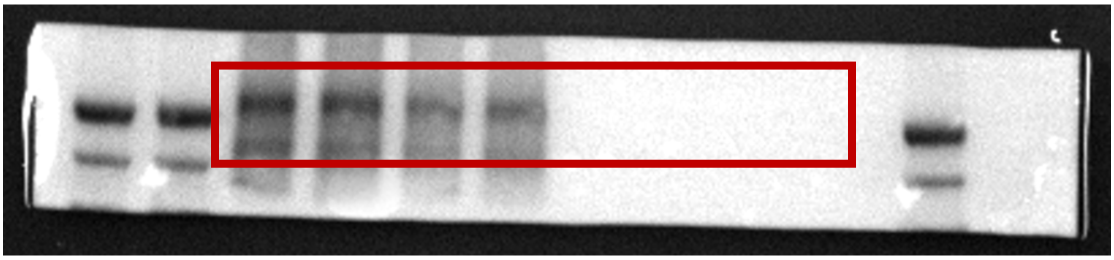

GAPDH

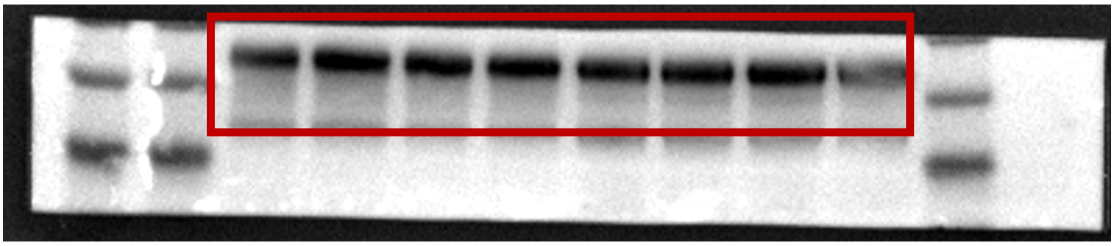

Figure 7F

BMP2

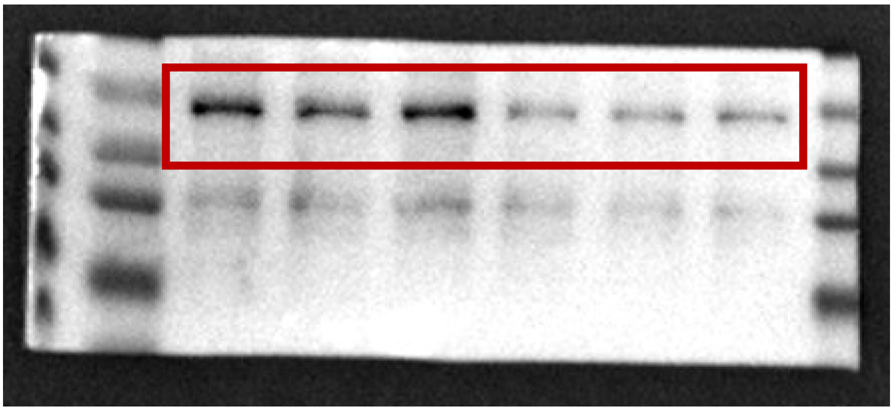

GAPDH for BMP2

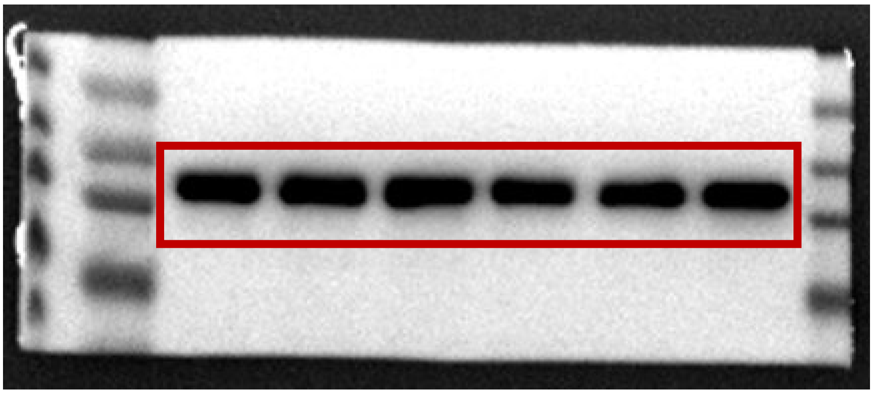

BMP4

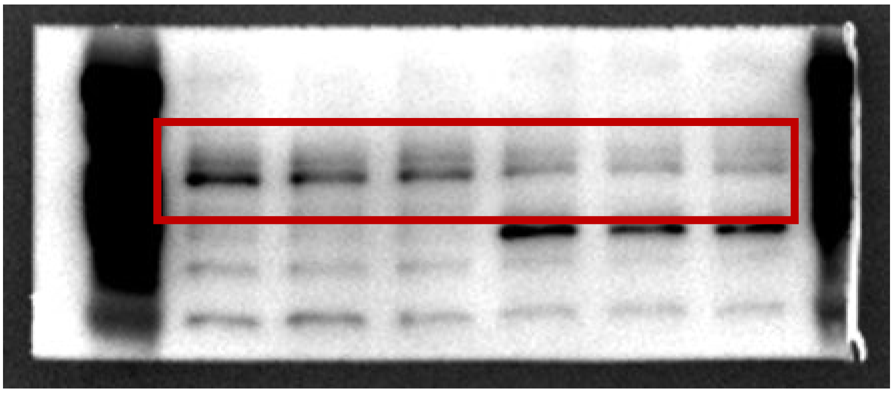

GAPDH for BMP4

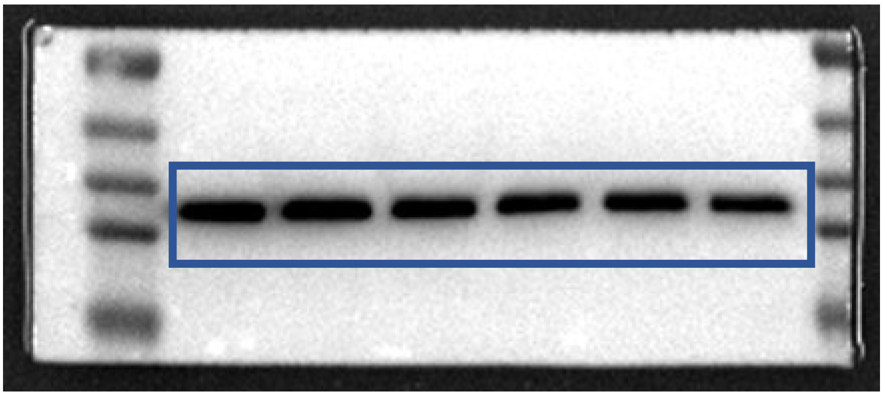

BMPR1A

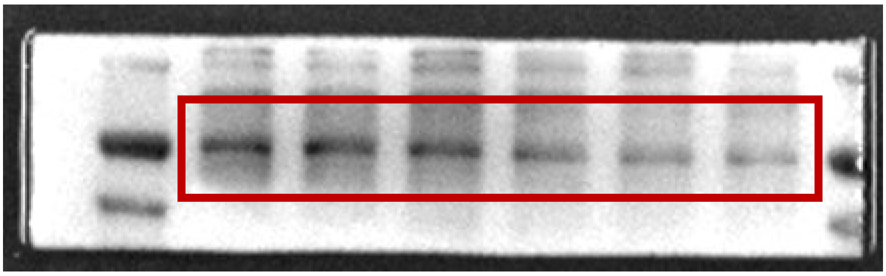

GAPDH for BMPR1A

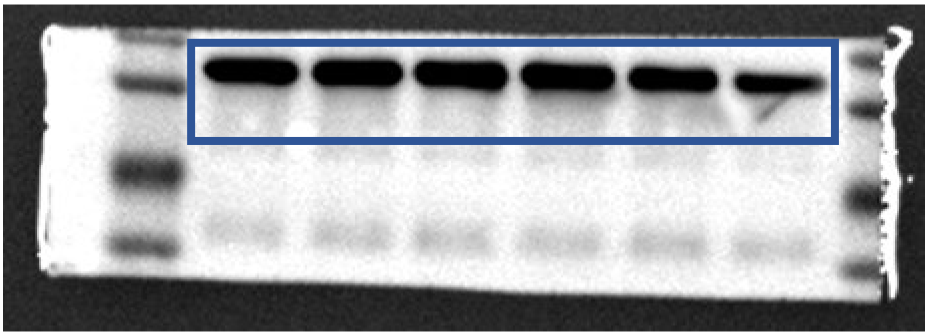

BMPR1B

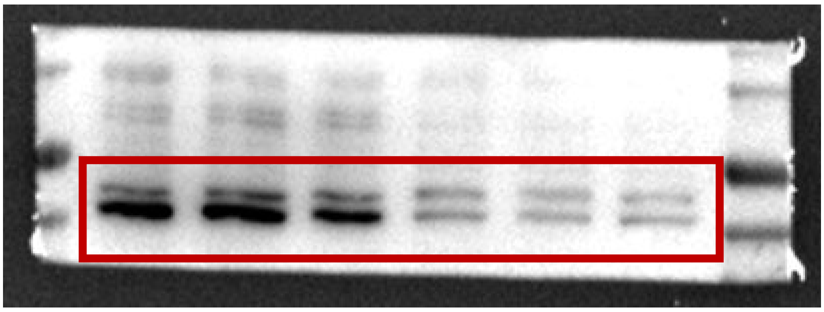

GAPDH for BMPR1B

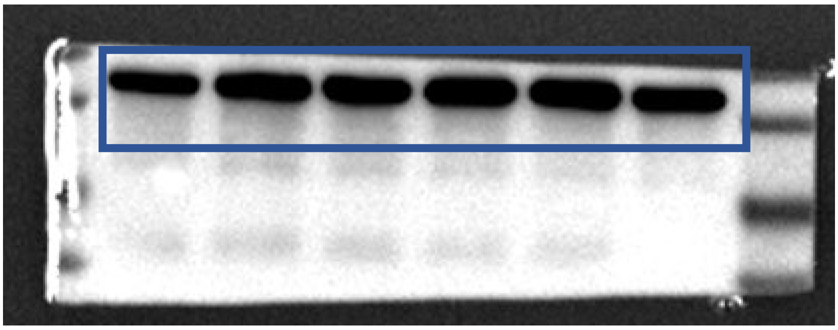

Figure 7H

p-SMAD1/5/9 (cell lysate)

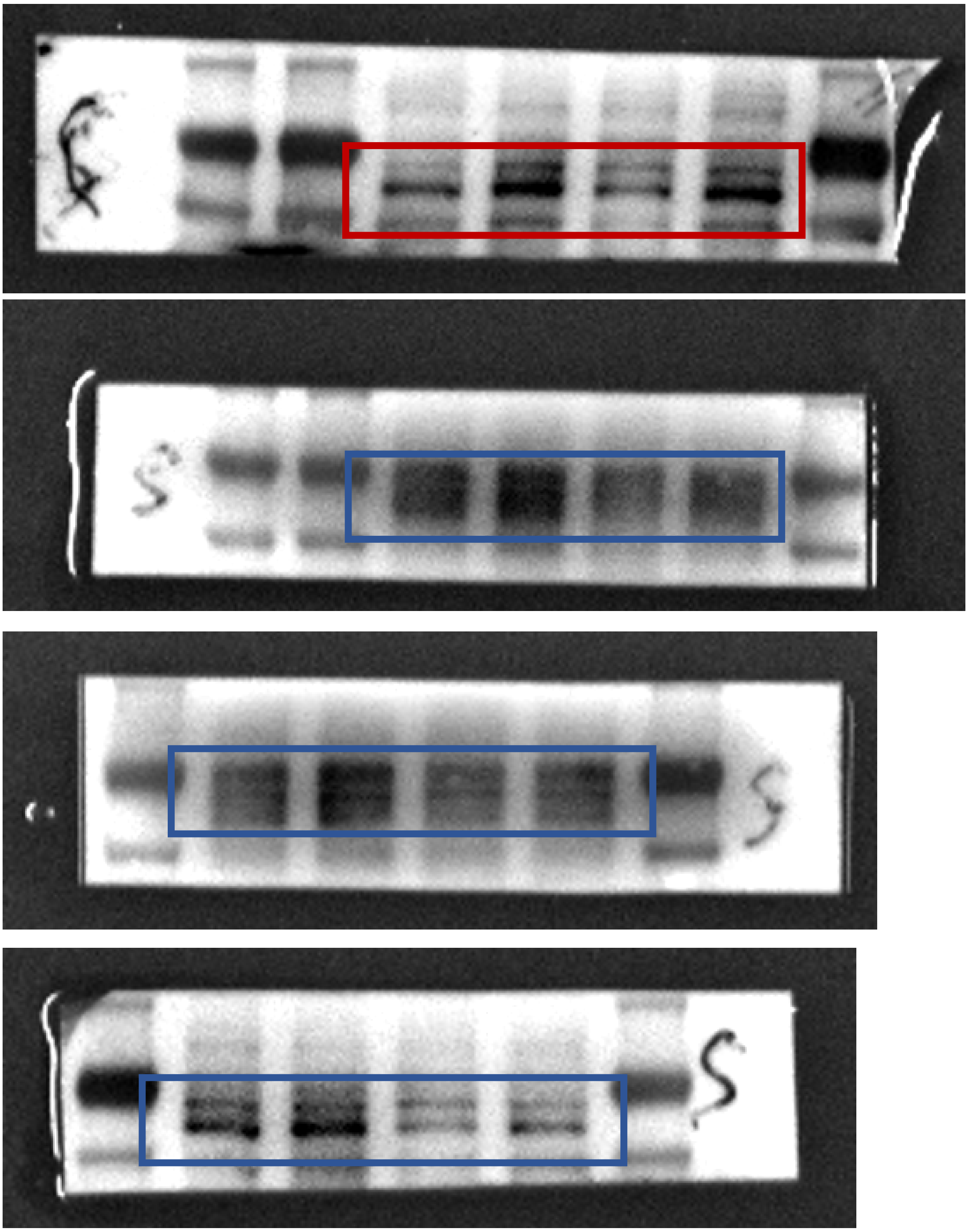

SMAD5 (cell lysate)

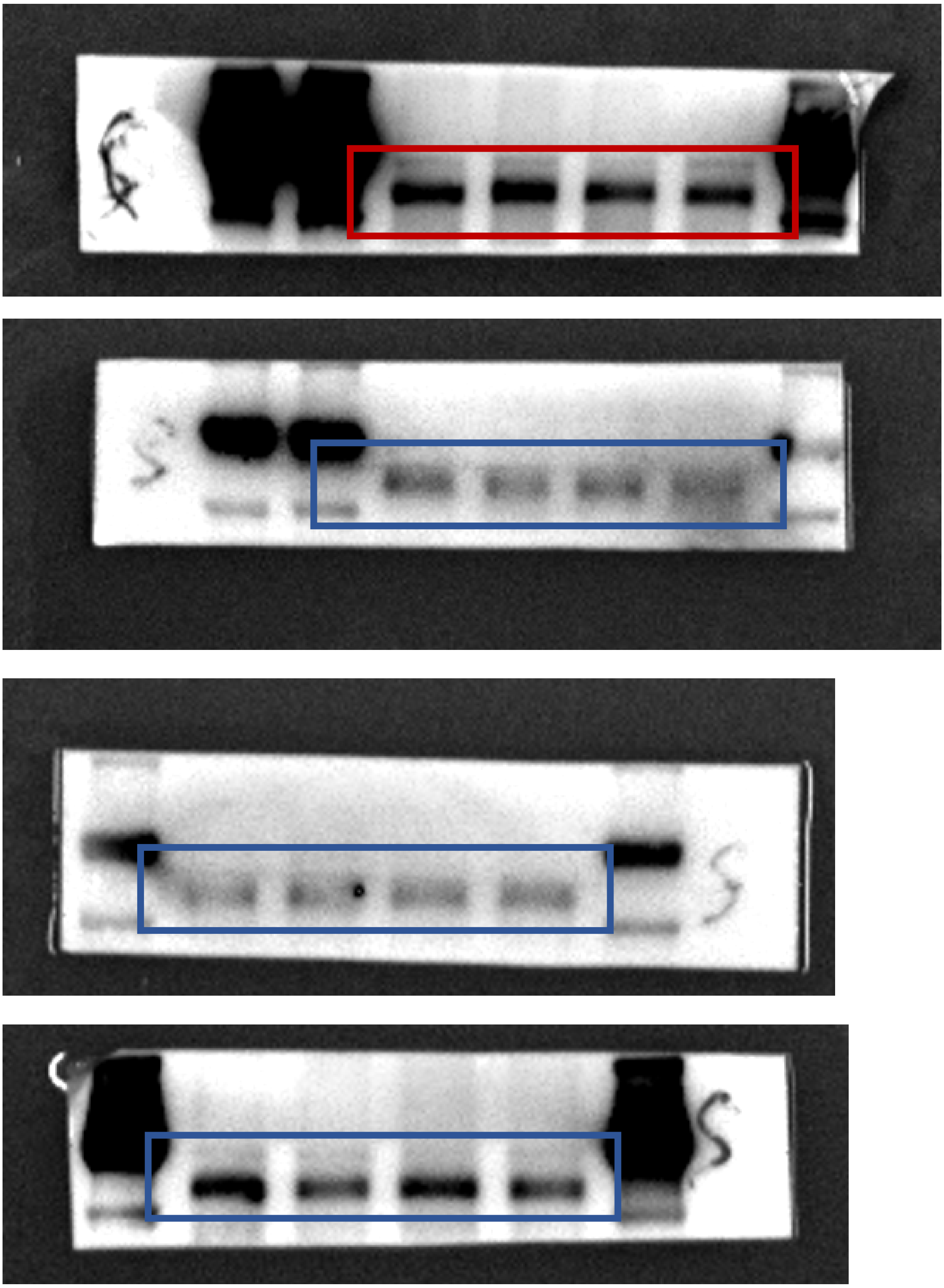

FADS1 (cell lysate)

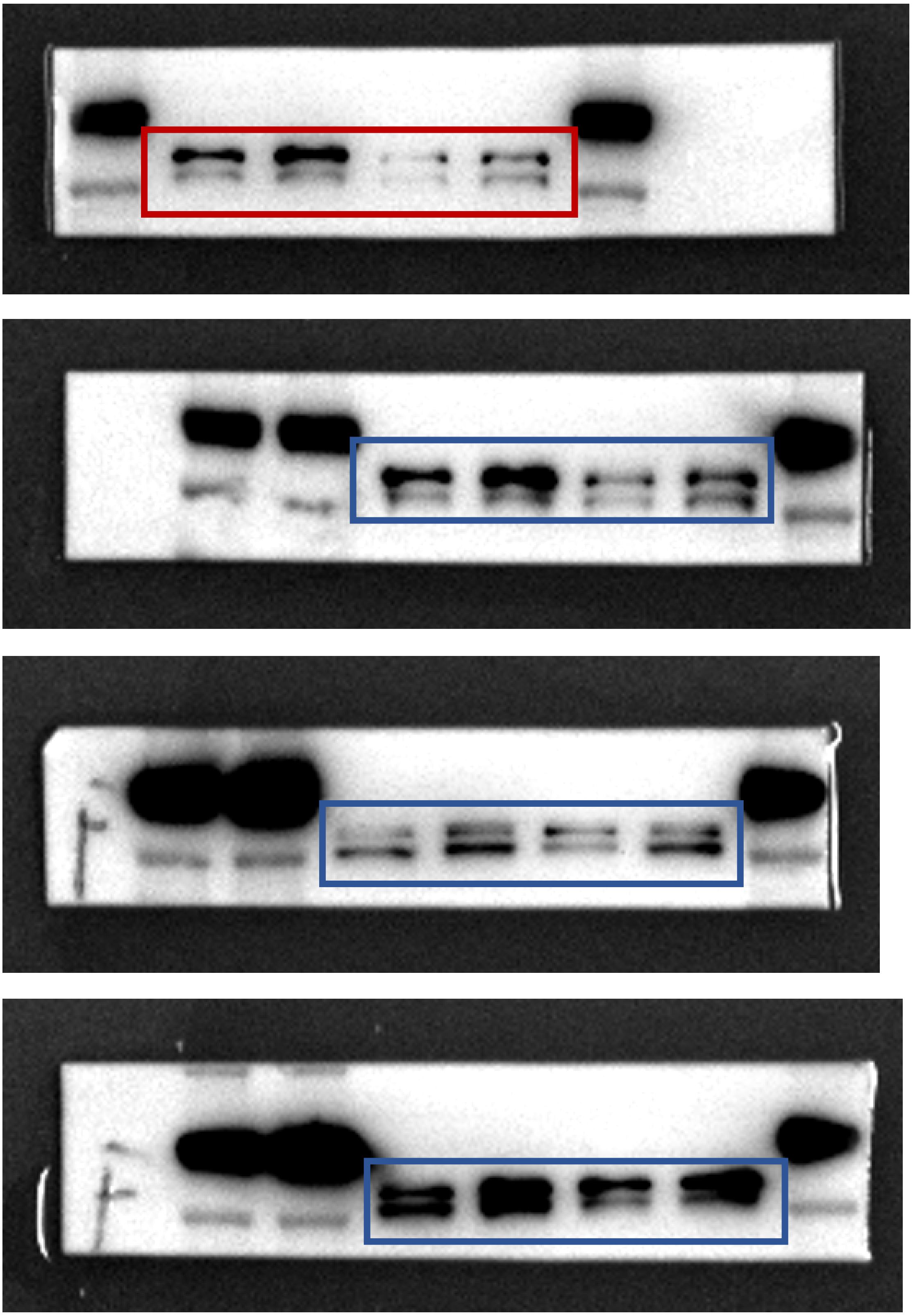

FADS2 (cell lysate)

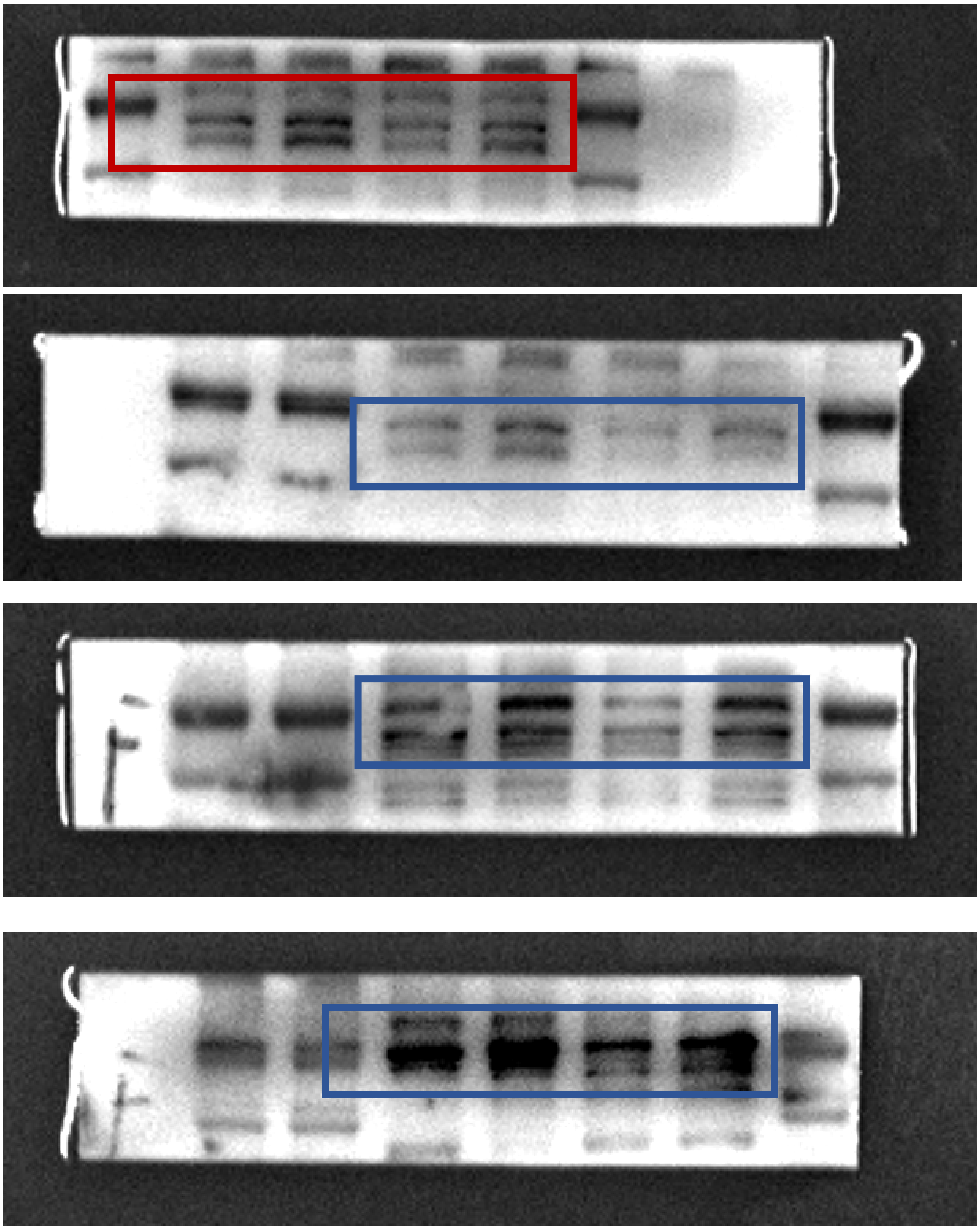

GAPDH (cell lysate)

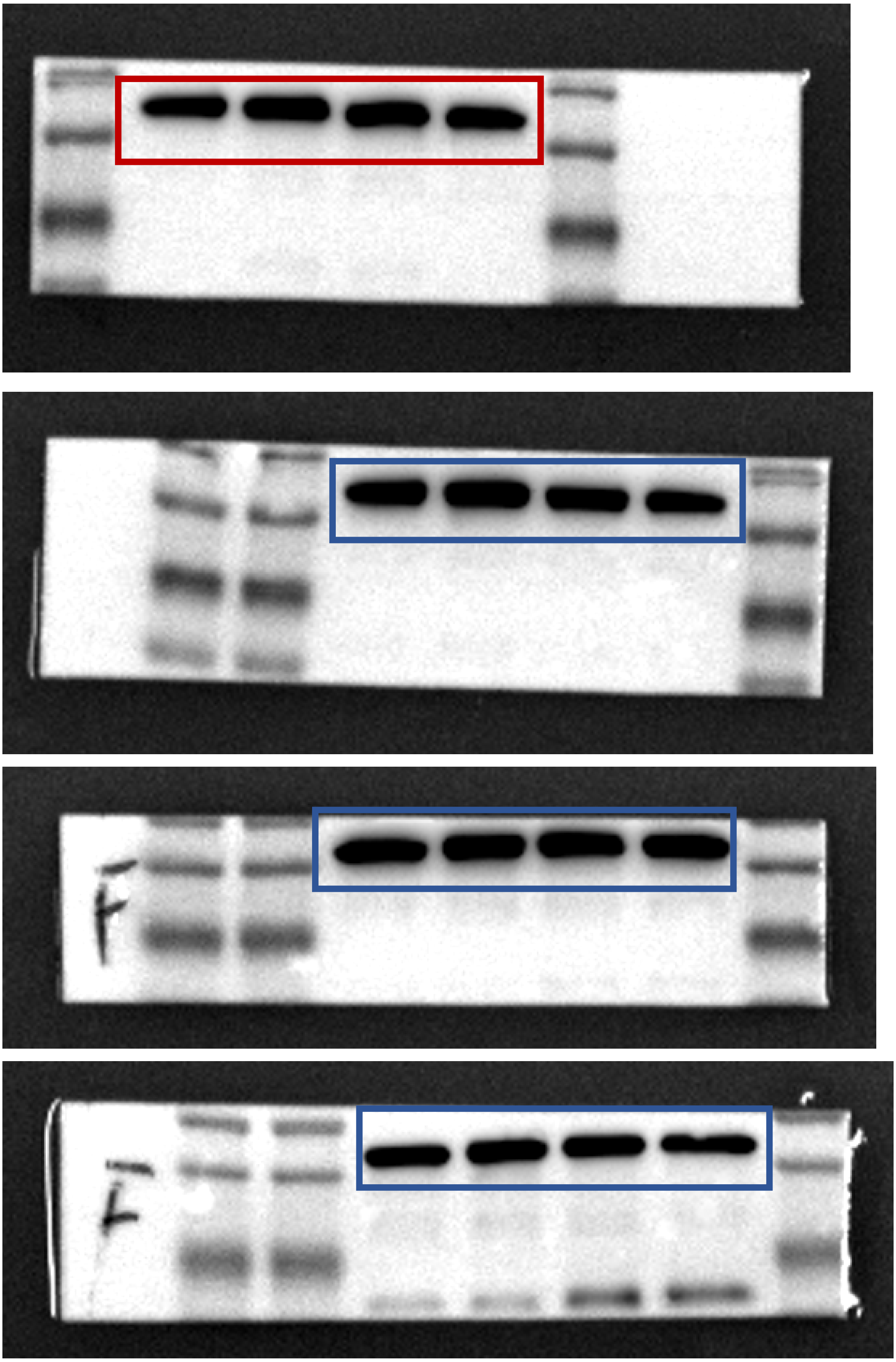

p-SMAD1/5/9

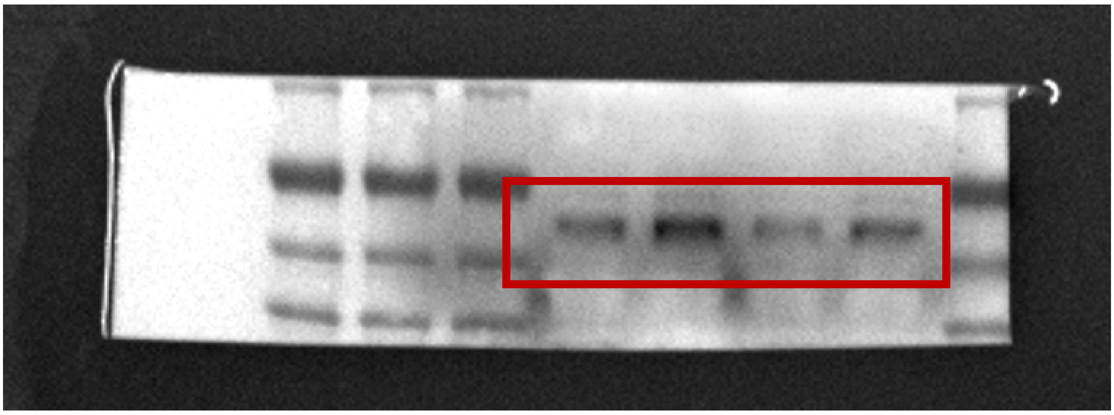
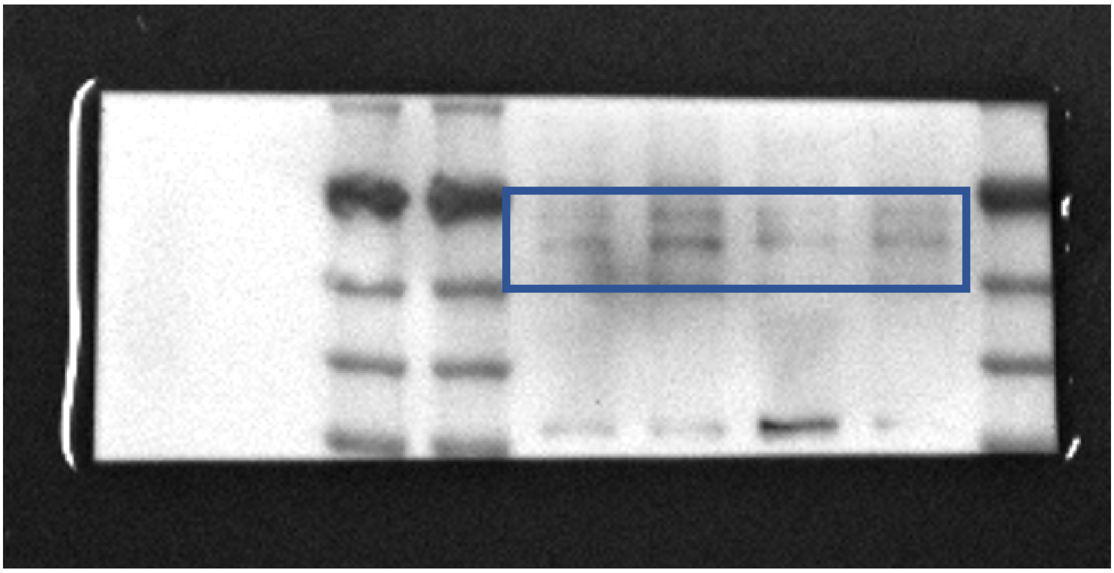
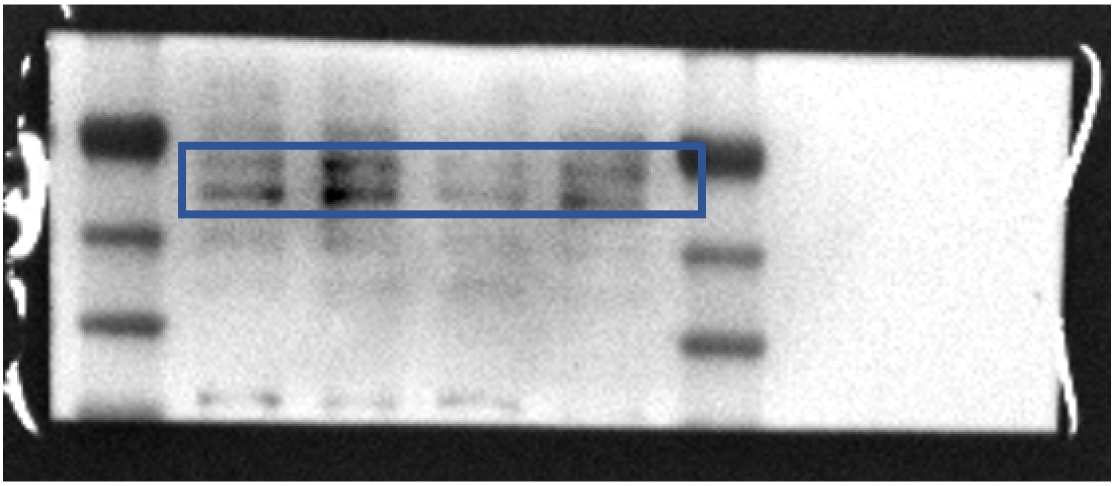

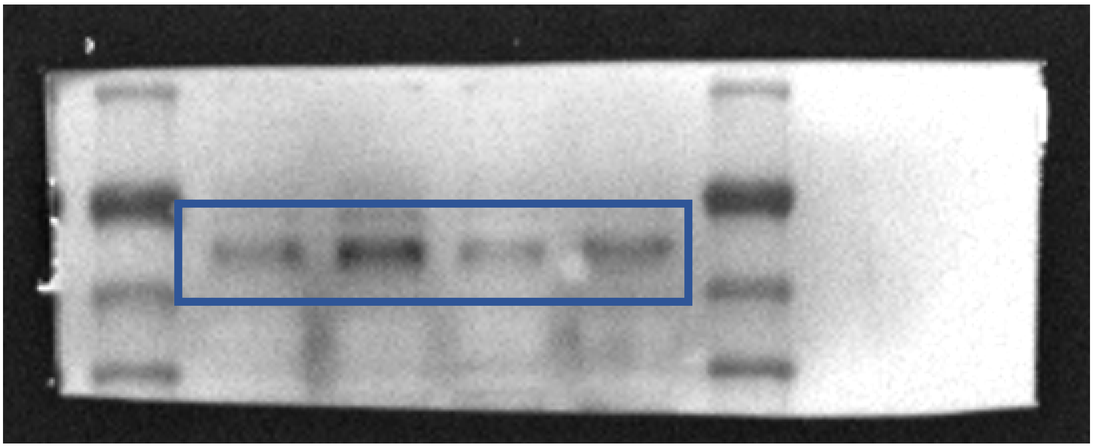

Histone H3

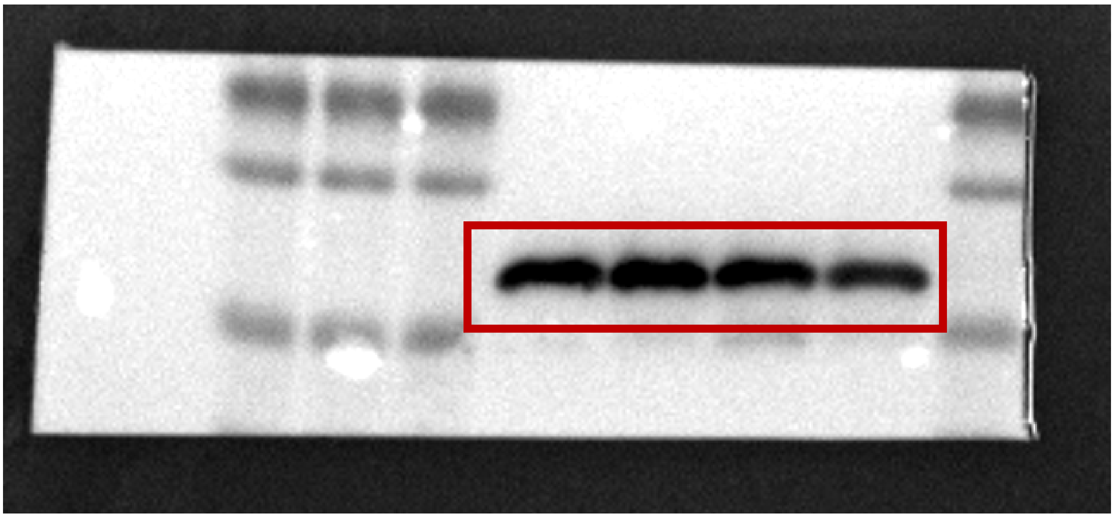

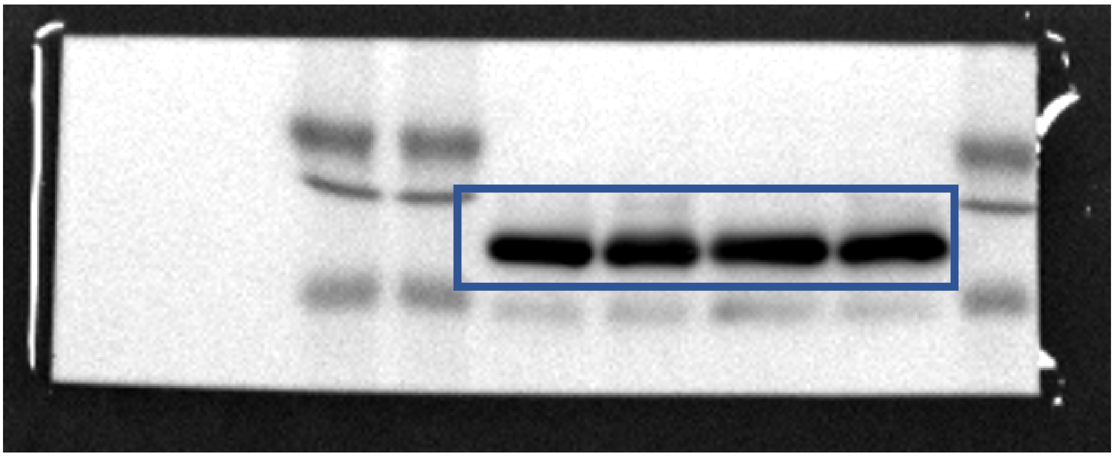

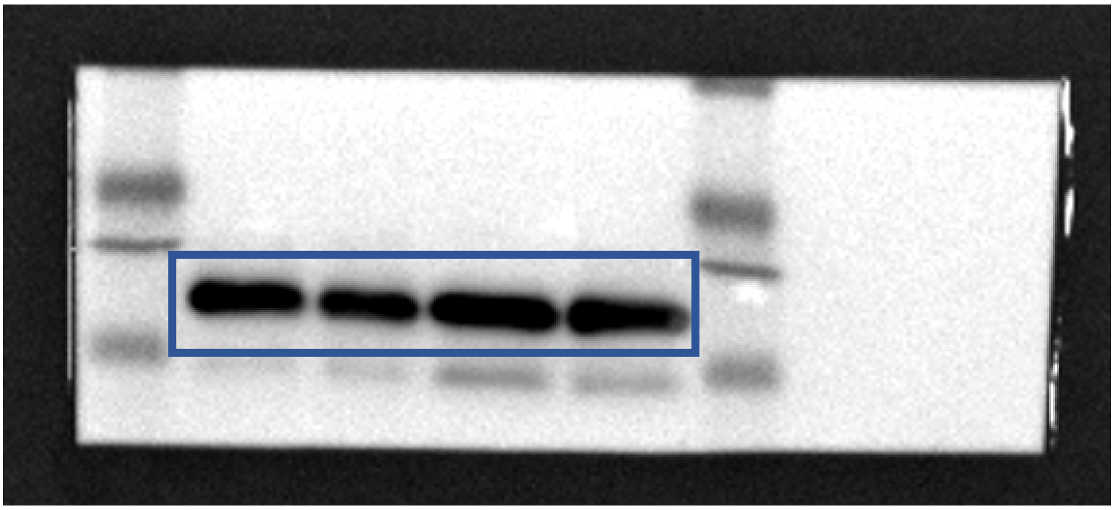
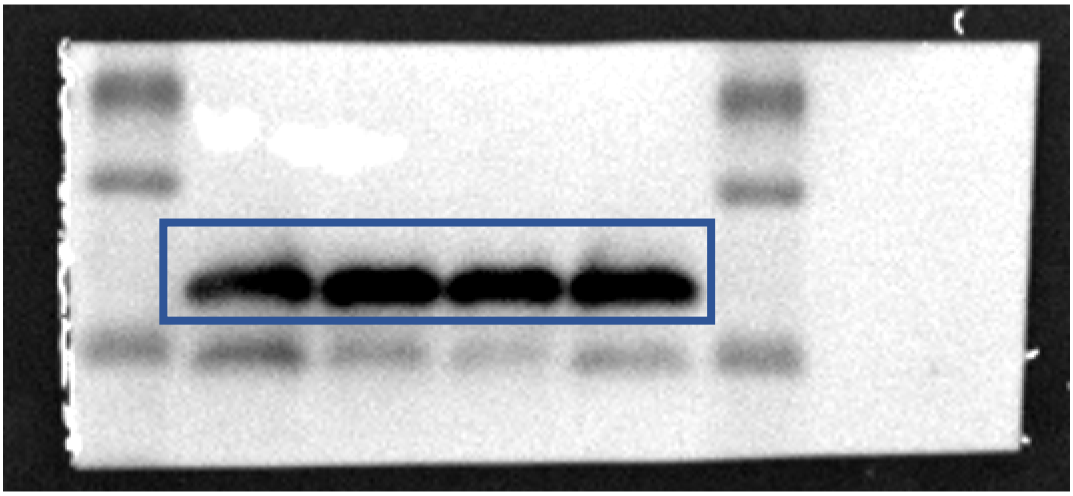

Figure 7N

KEAP1

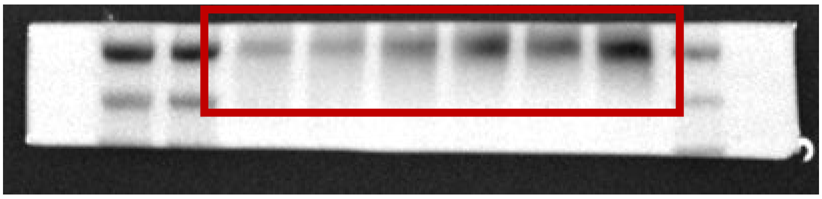

NRF2

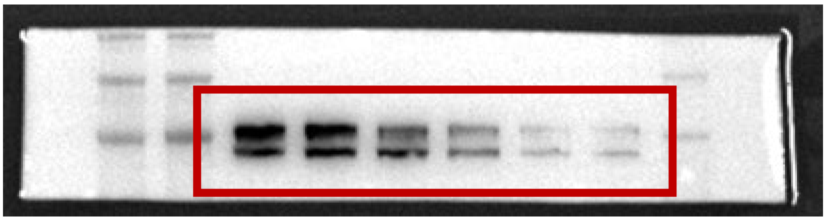

GAPDH

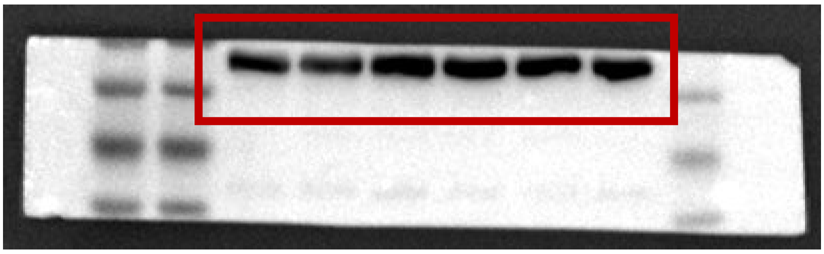

Figure 7O

Ubiquitin

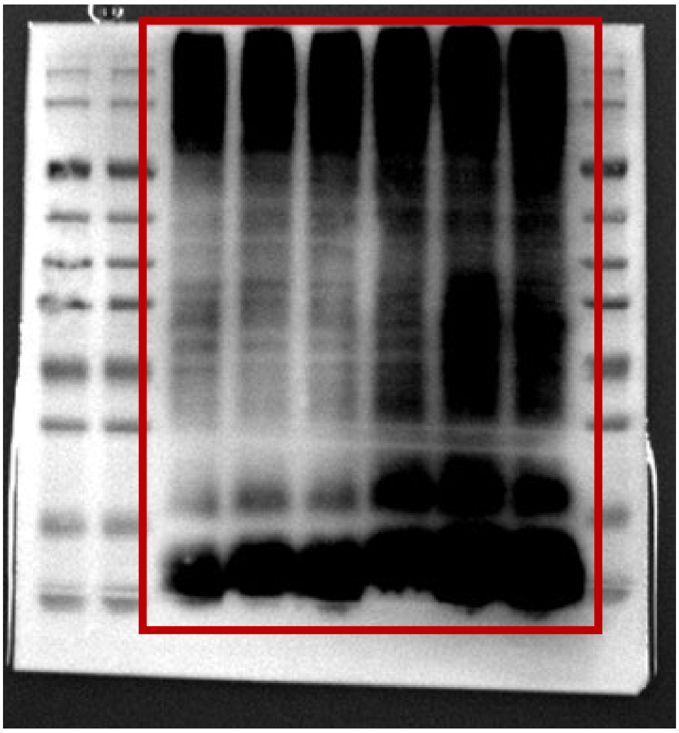

NRF2

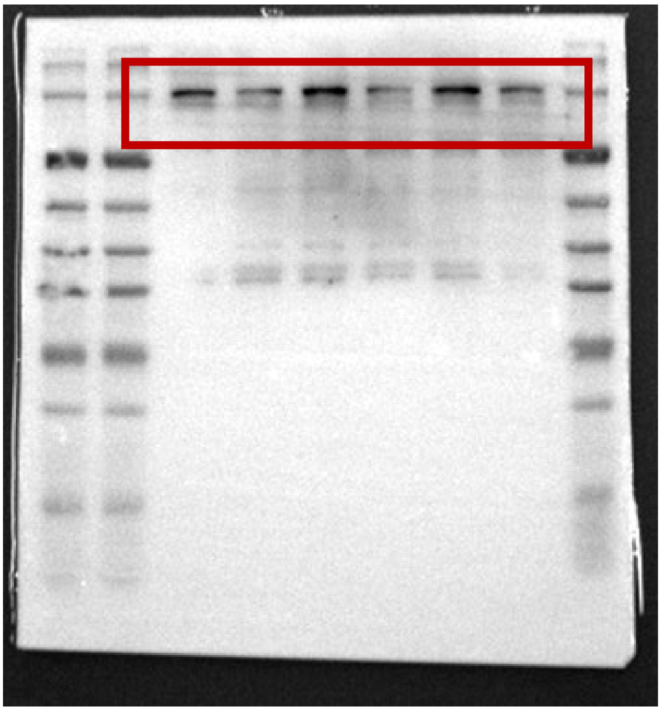

Figure 7T

HuR

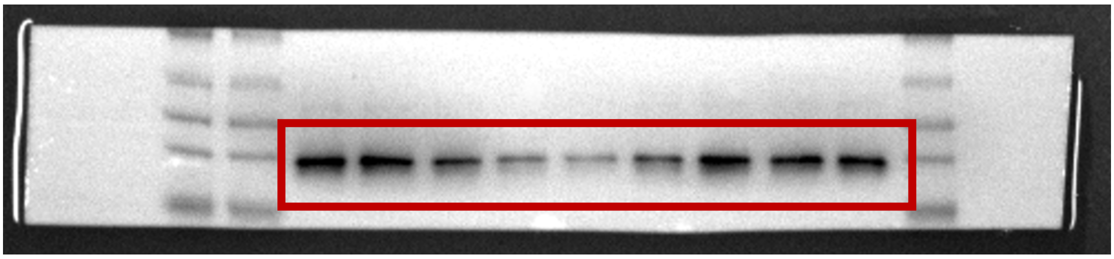

GAPDH

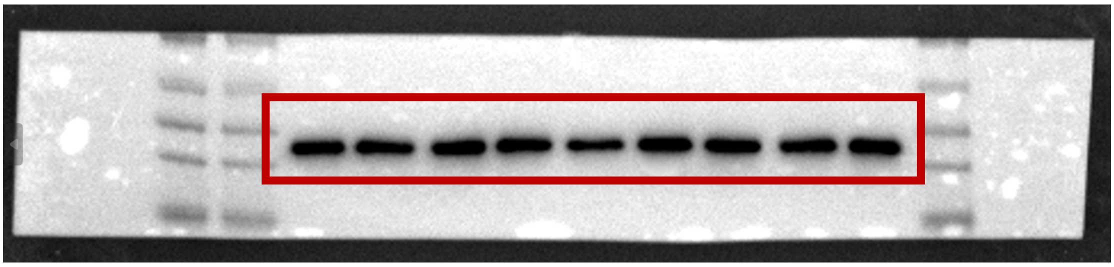

HuR

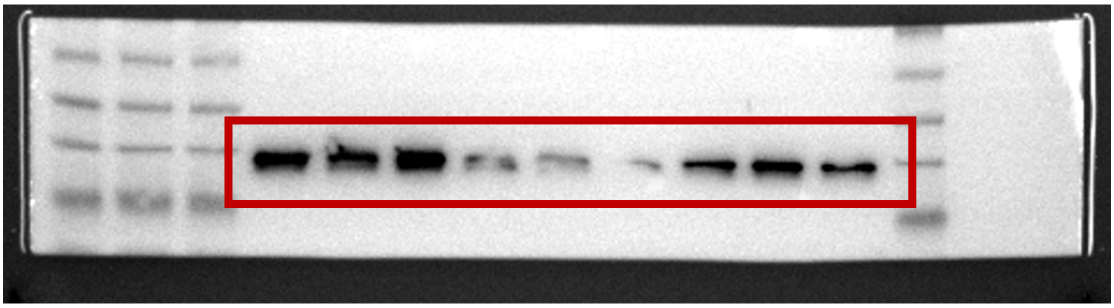

GAPDH

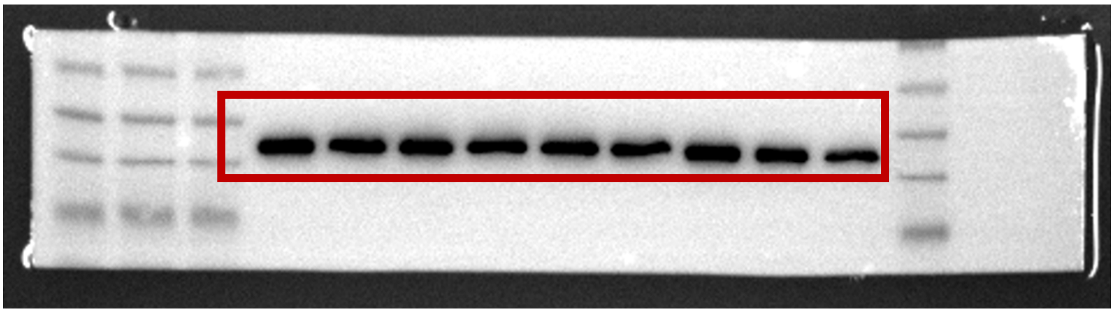

Figure 8D

Pan-kla

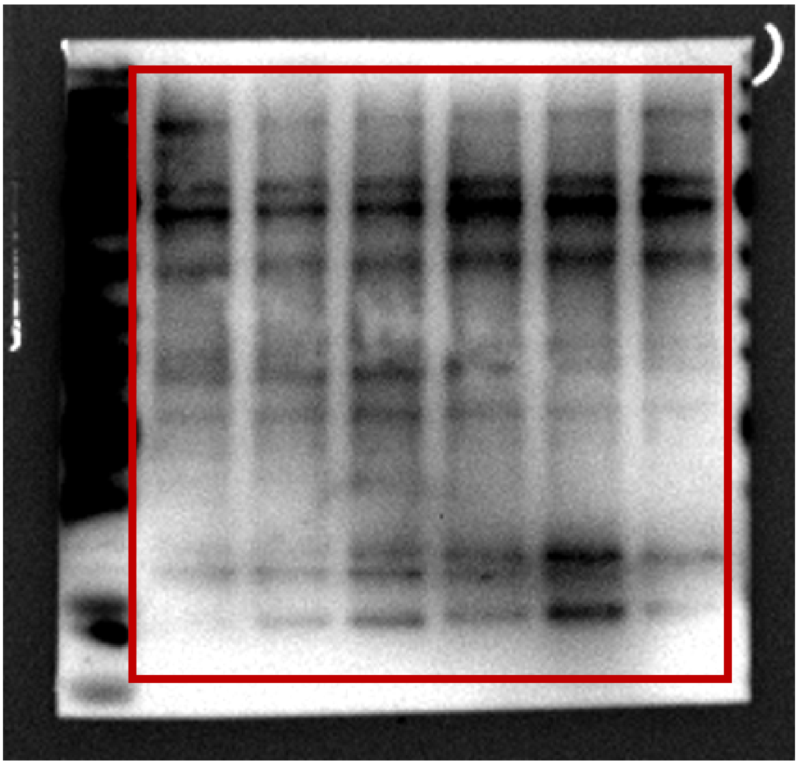

β-actin

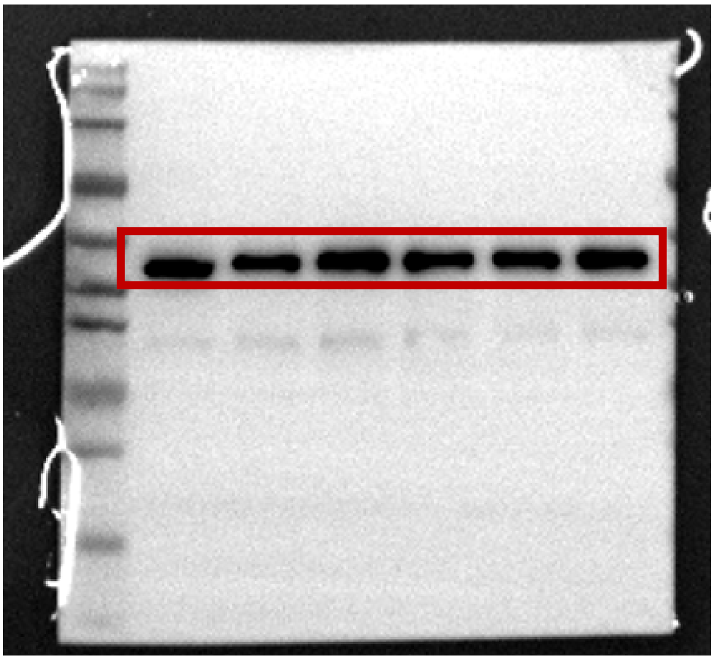

Figure 8E

H3K9la

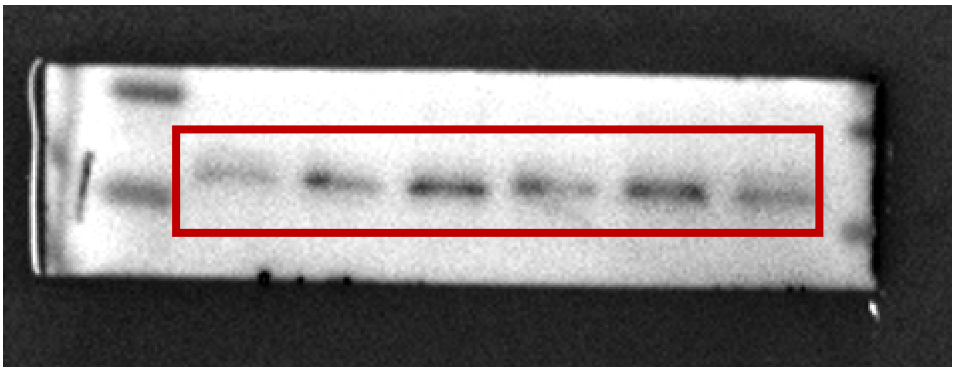

H3K14la

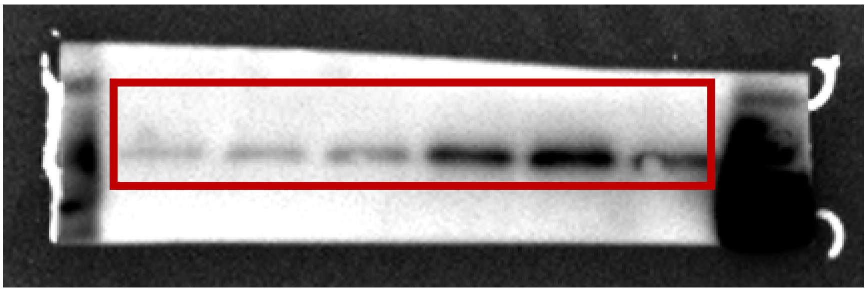

H3K18la

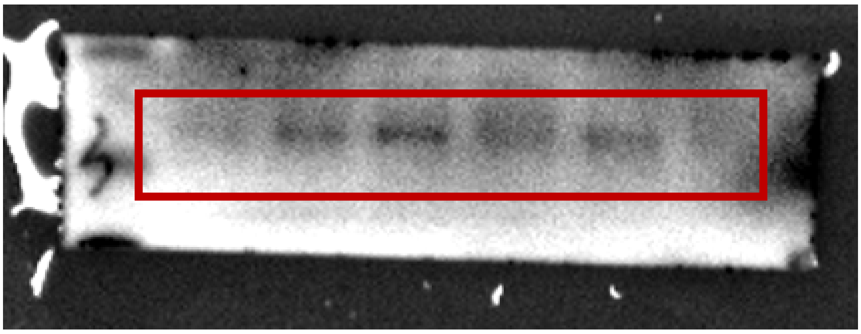

H3K27la

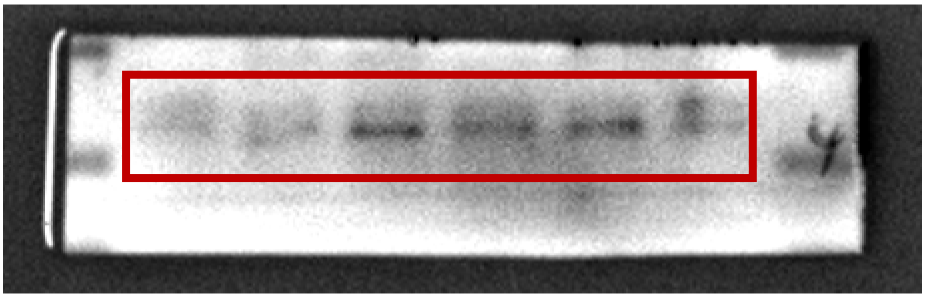

Figure 8H

H3K14la

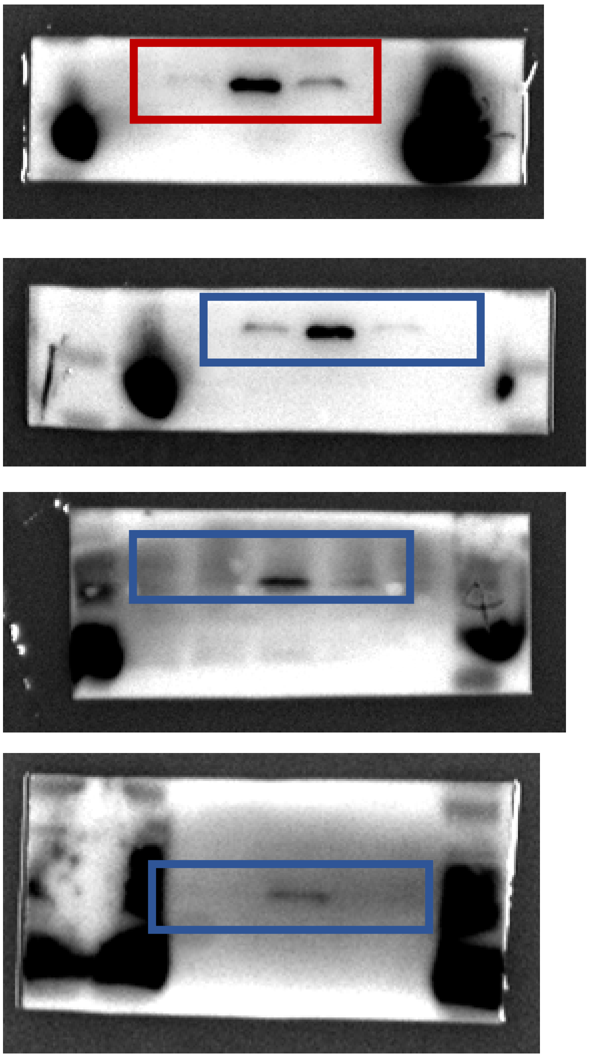

H3

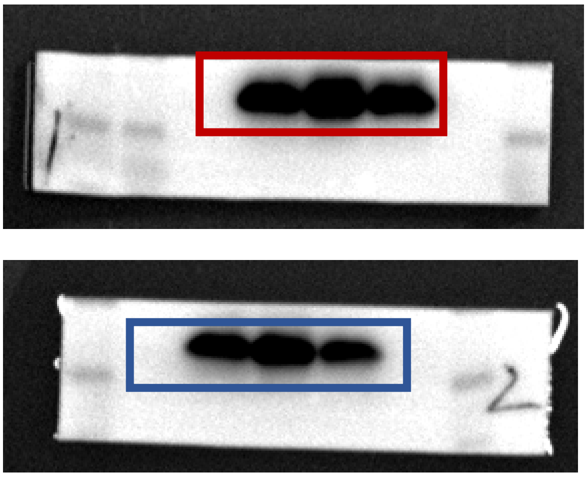

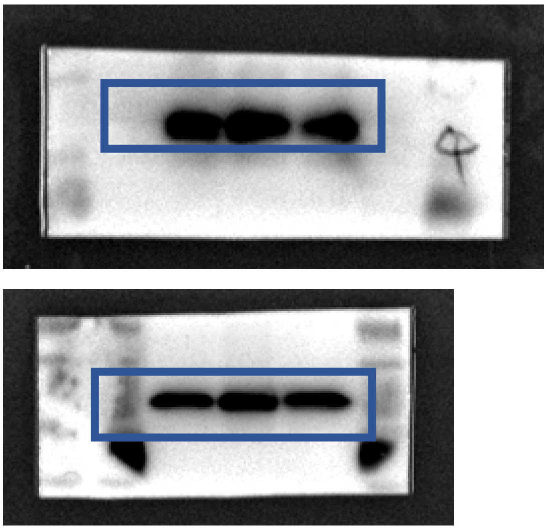

PARK7

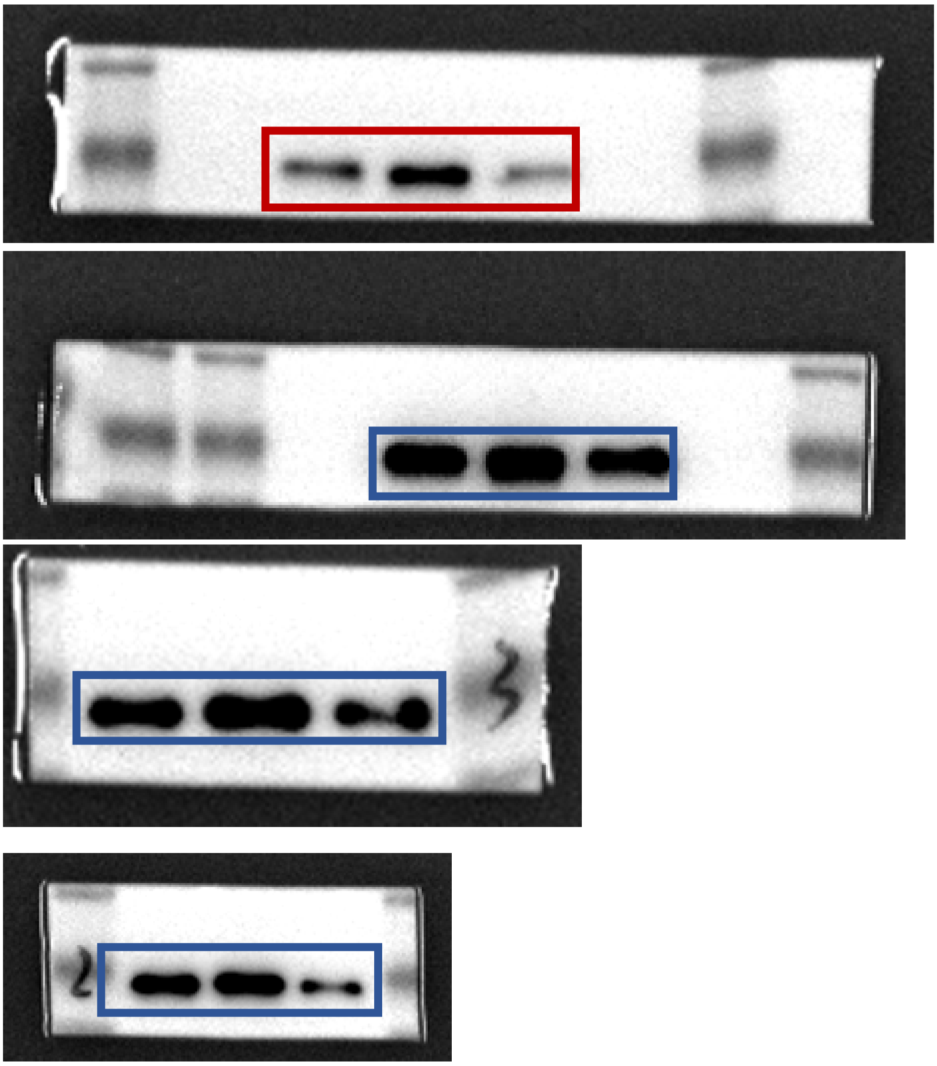

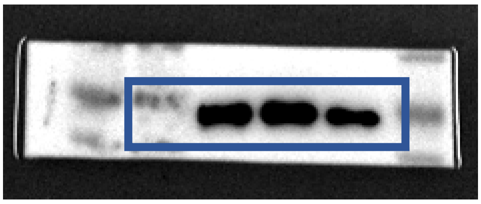

β-actin

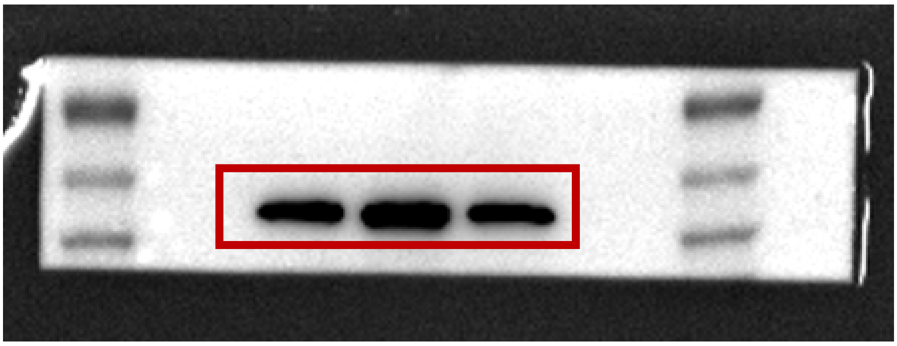

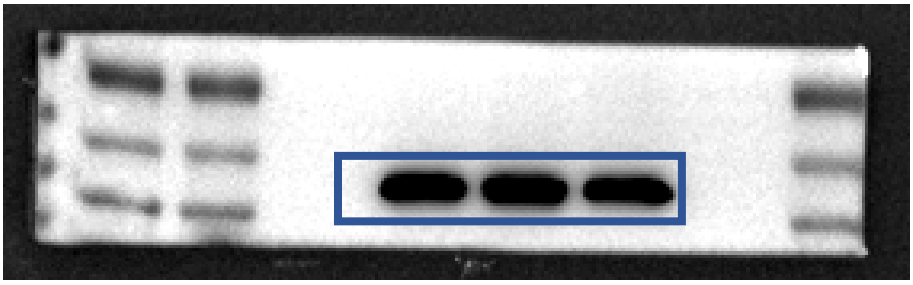

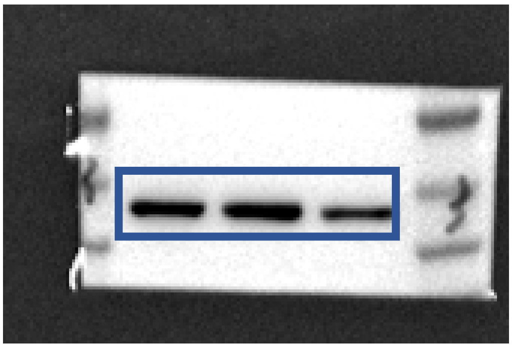

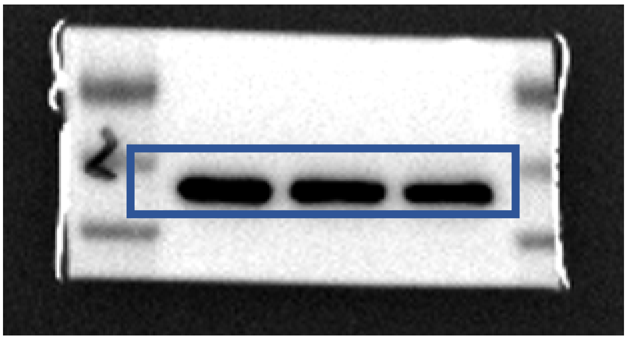

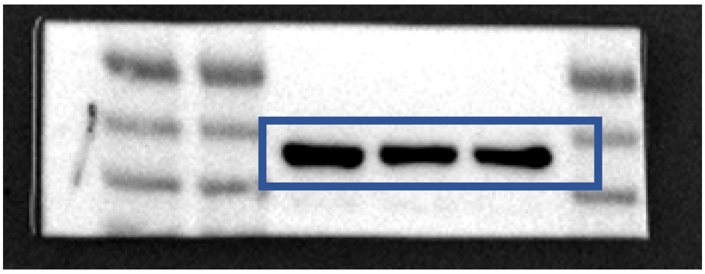

Figure 8J

H3K14la

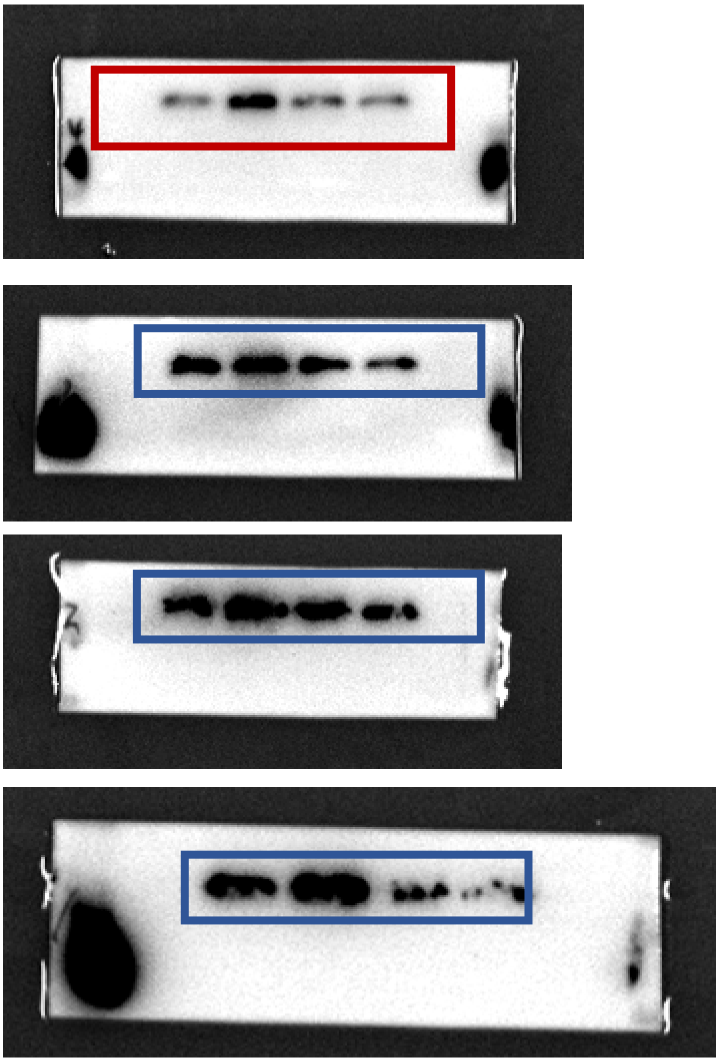

Histone H3

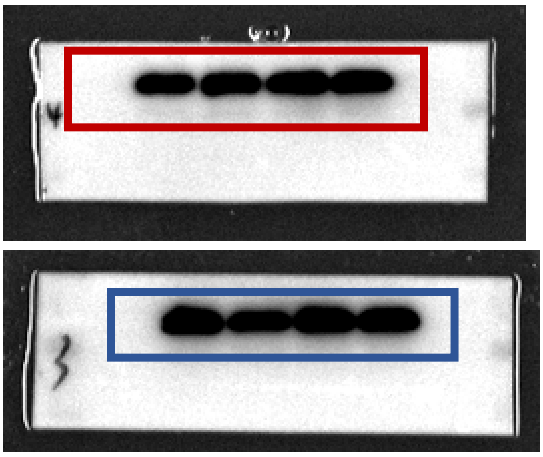

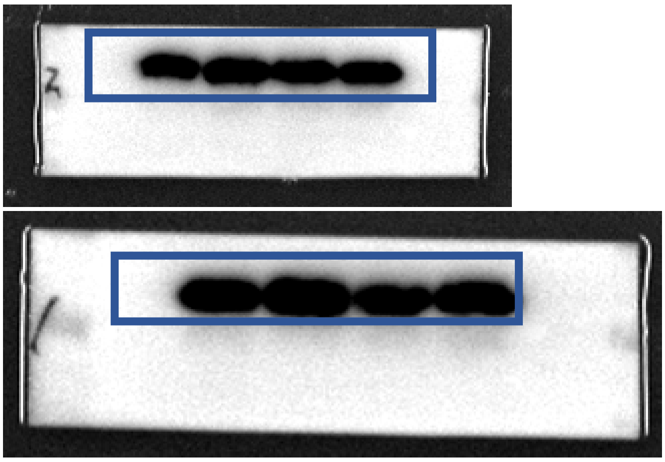

PARK7

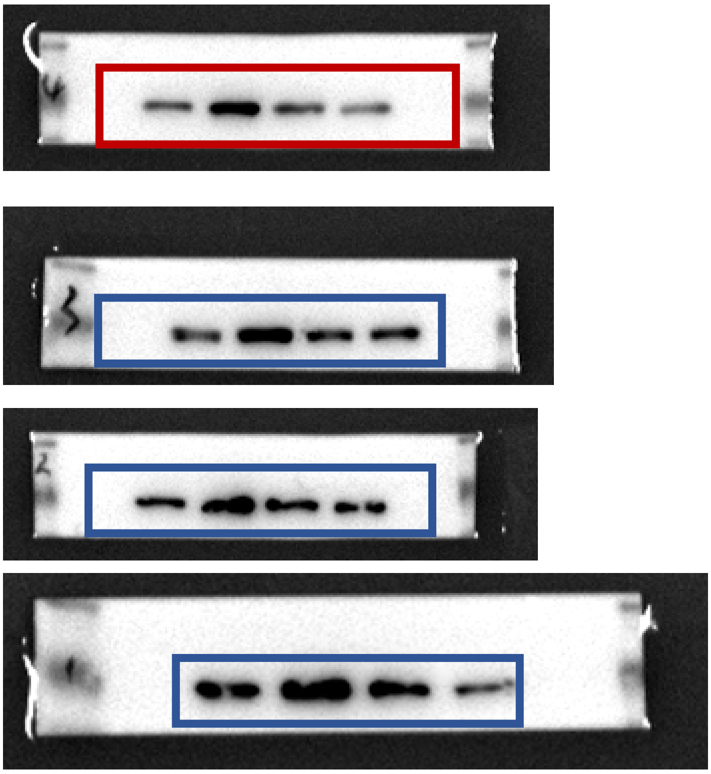

β-actin

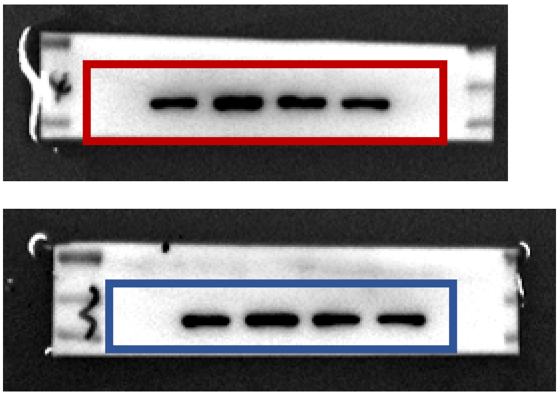

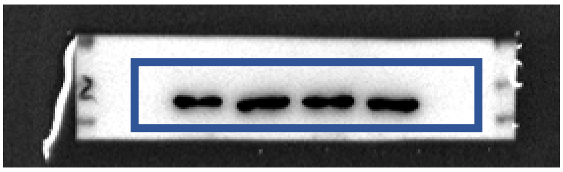
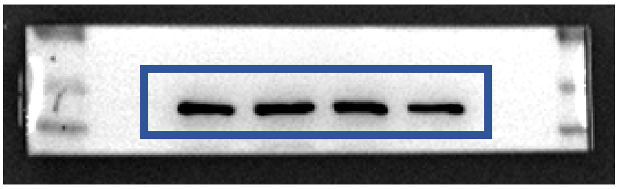

Figure 8N

H3K14la

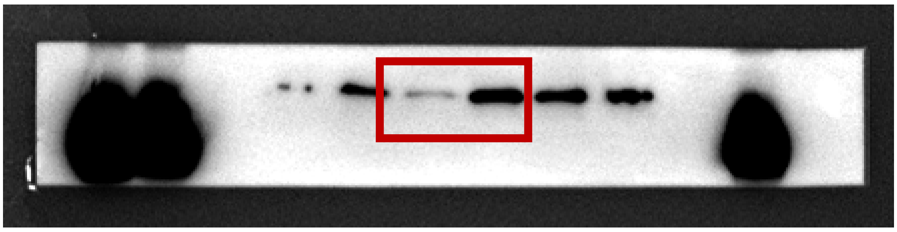

Histone H3

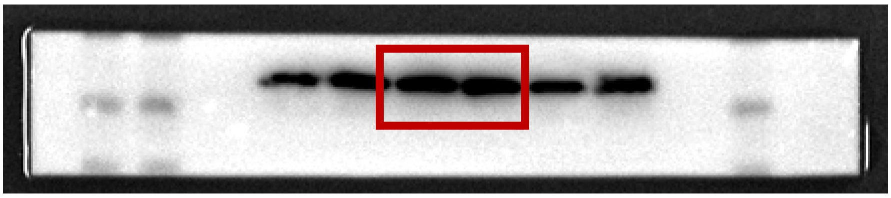

PARK7

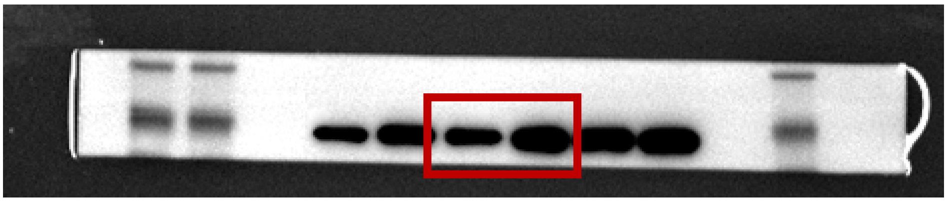

β-actin

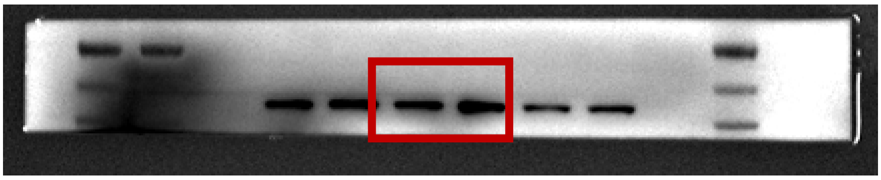

Figure 8P

H3K14la

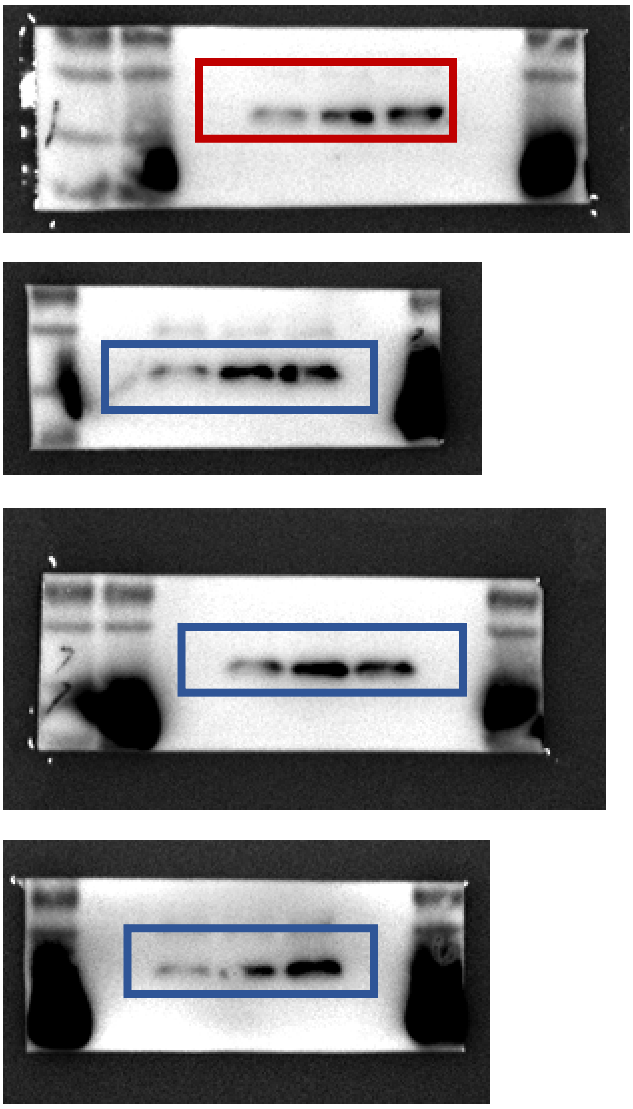

Histone H3

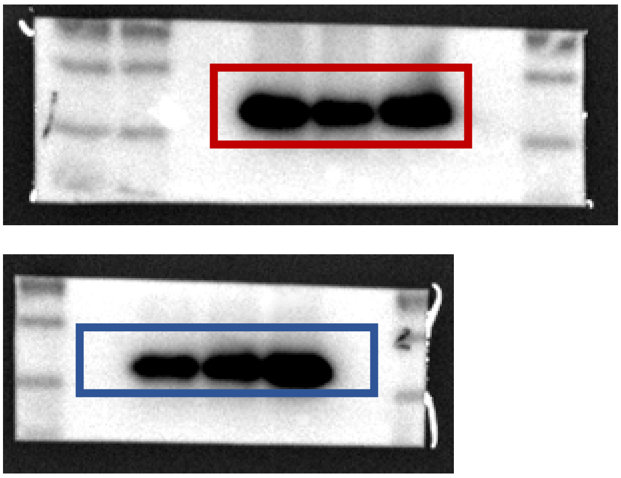

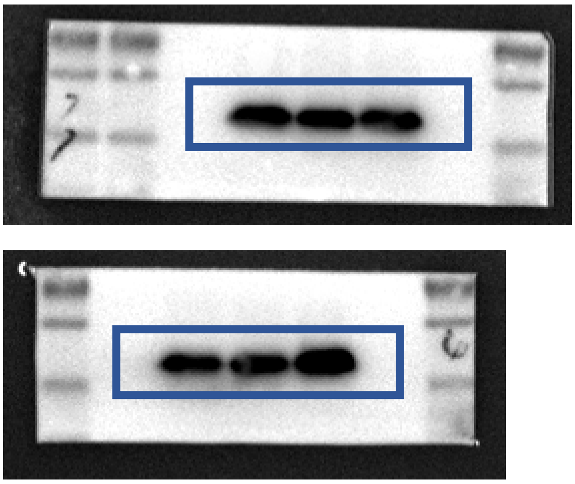

PARK7

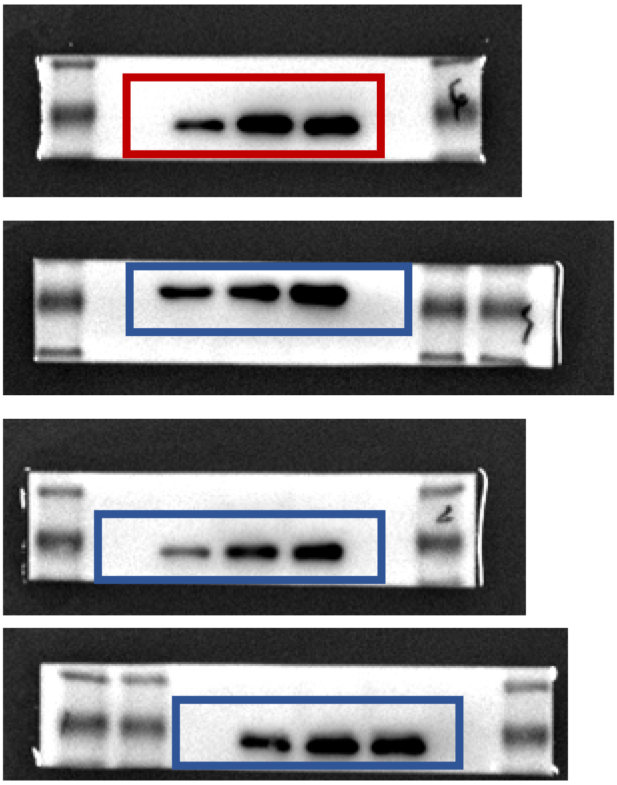

β-actin

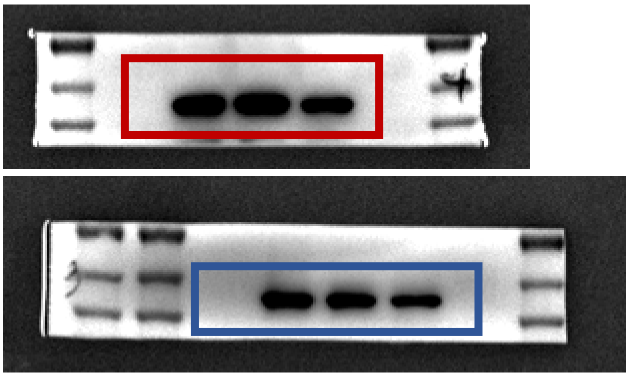

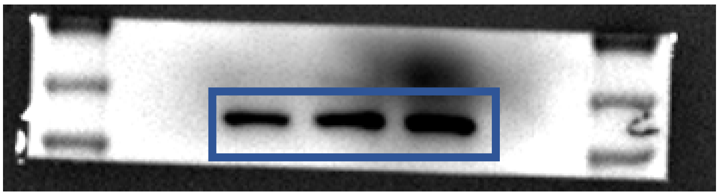

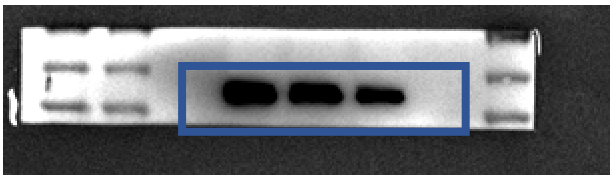

Figure 8Q

P300

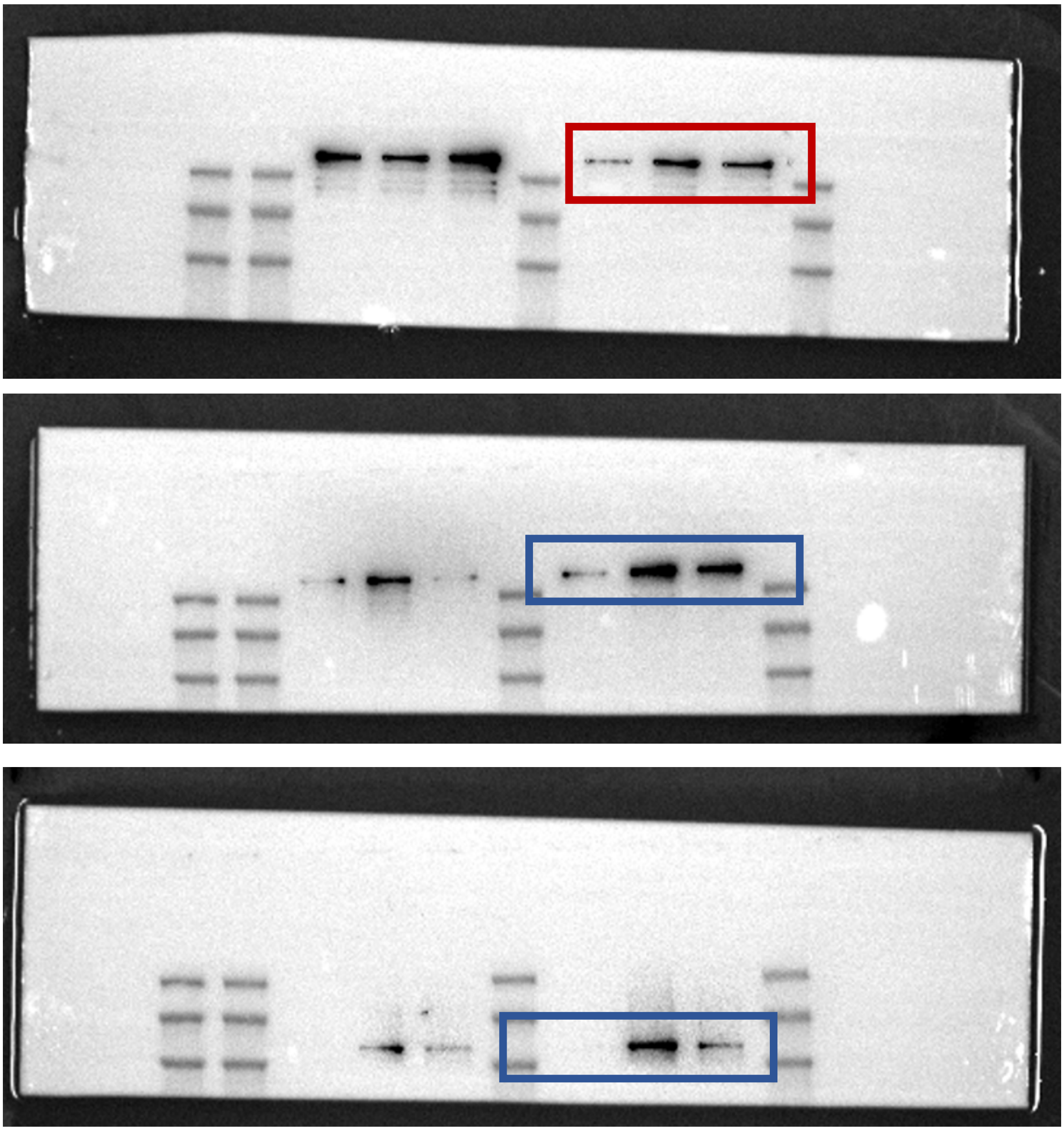

GAPDH

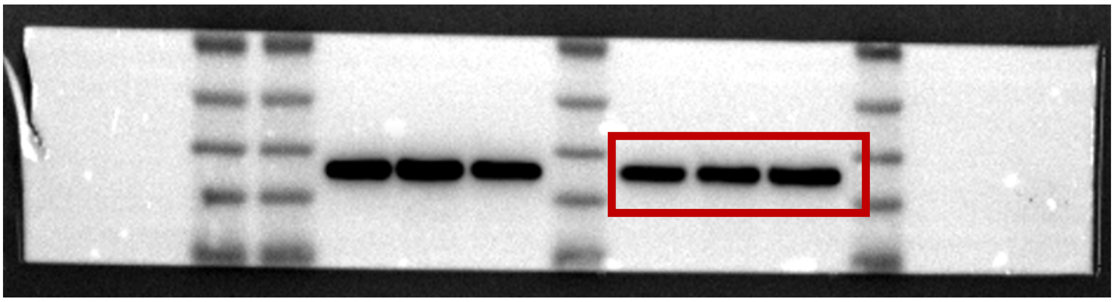

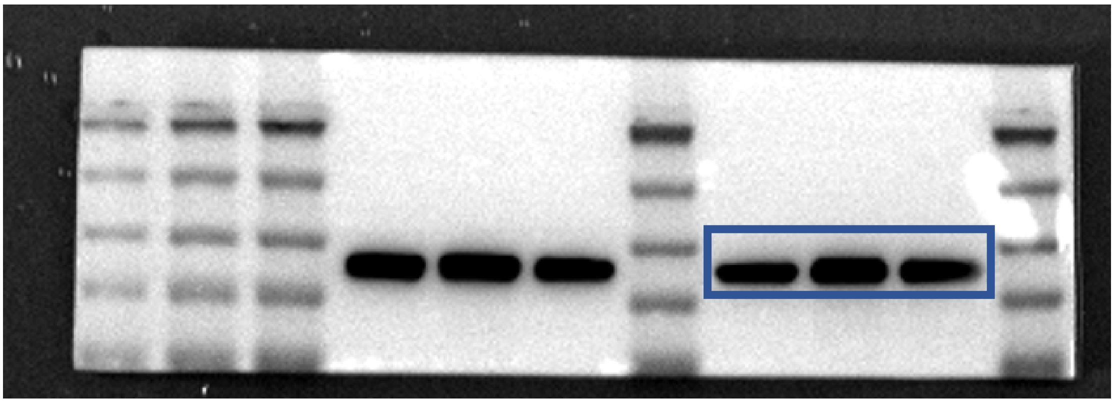
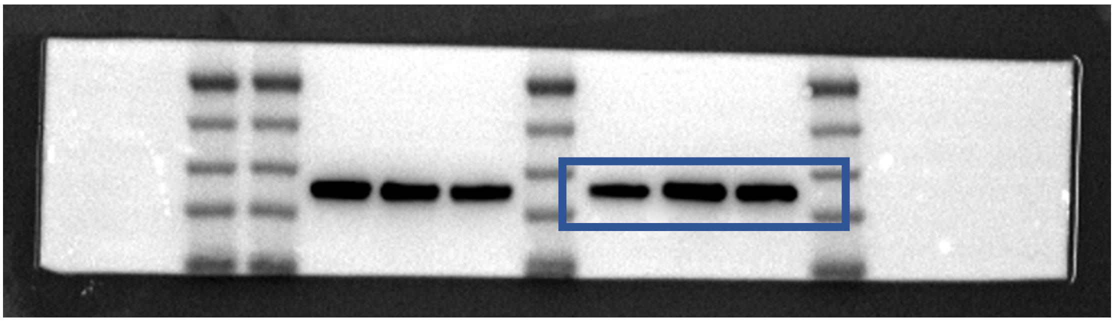

Figure 9K

H3K14la

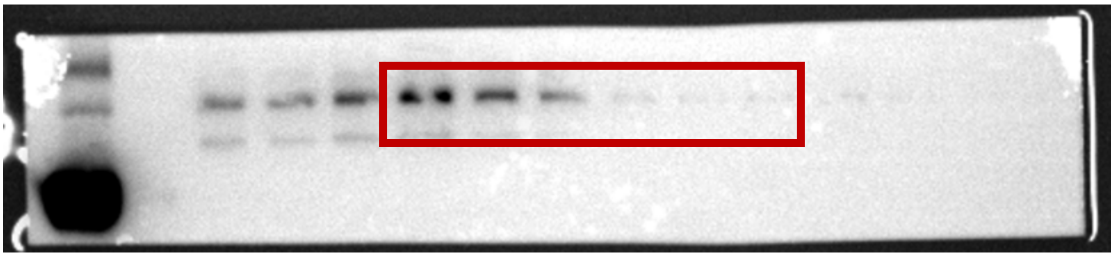

Histone H3

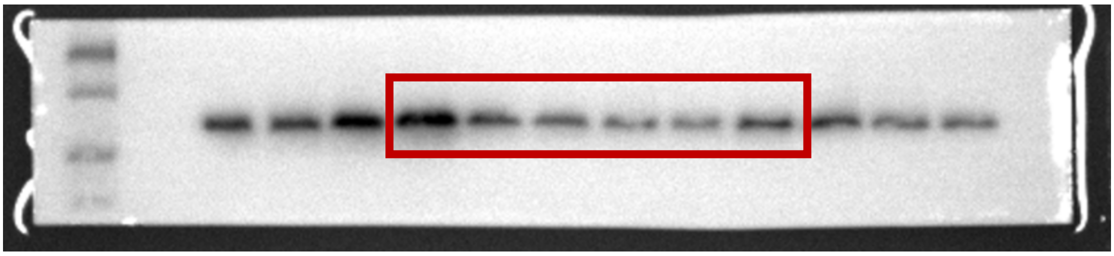

Figure 9N

PARK7

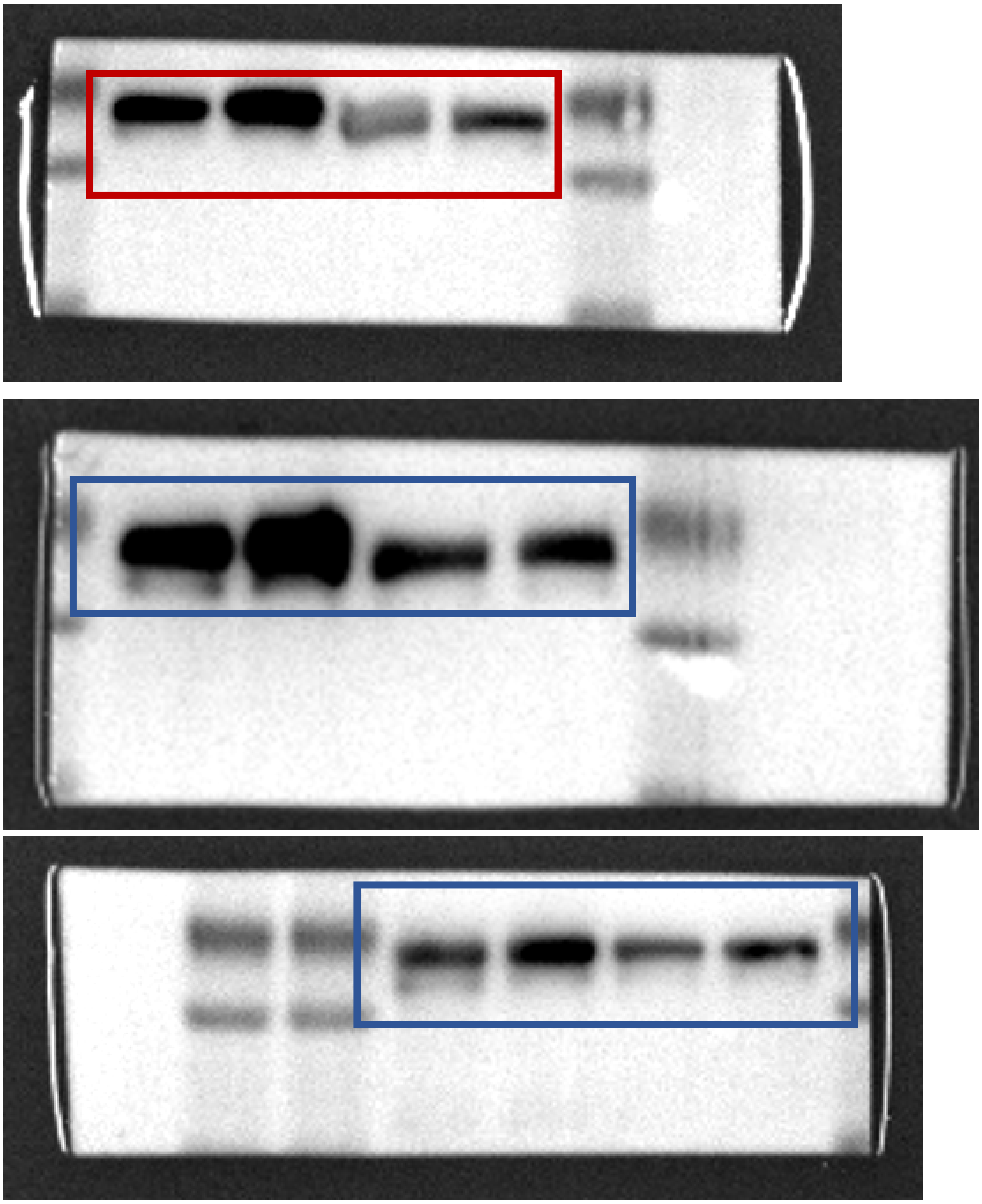

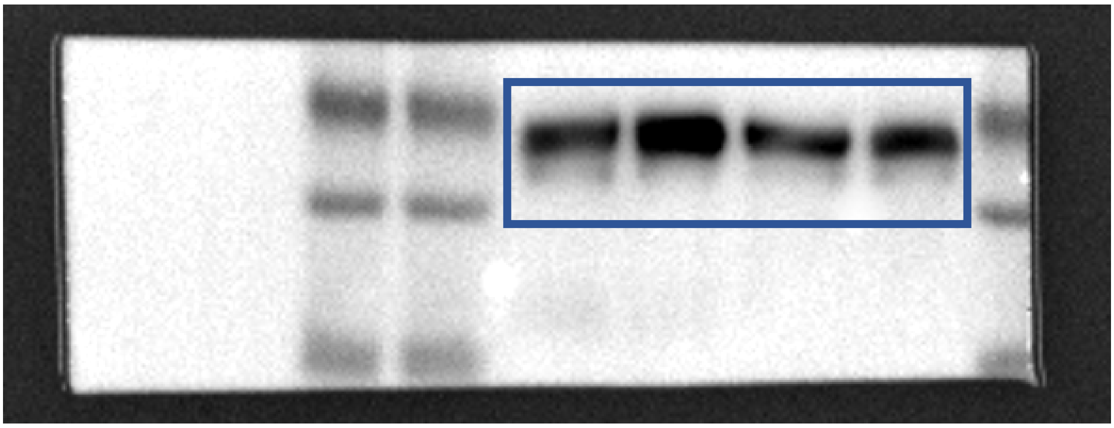

GAPDH

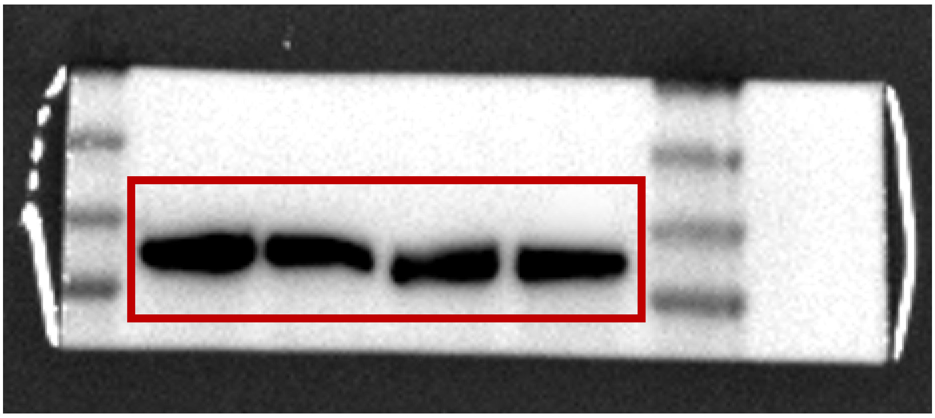

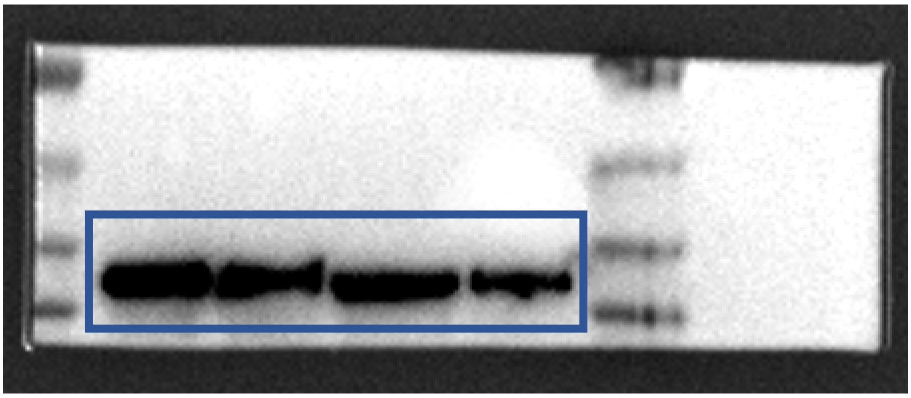

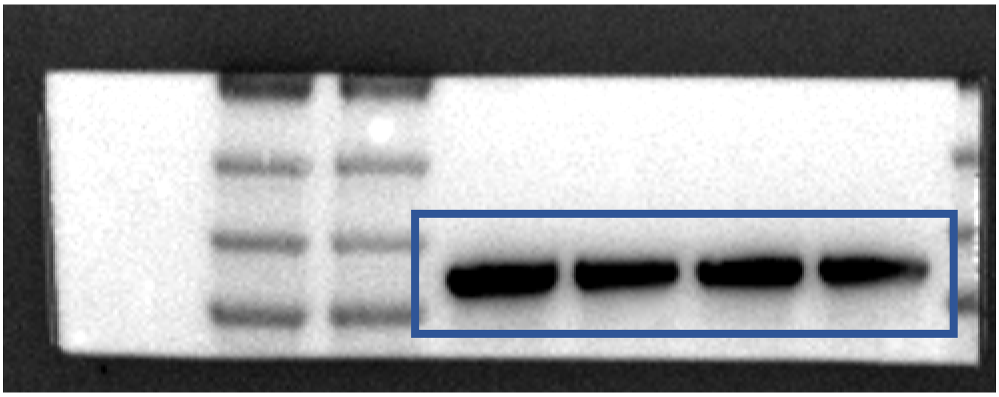

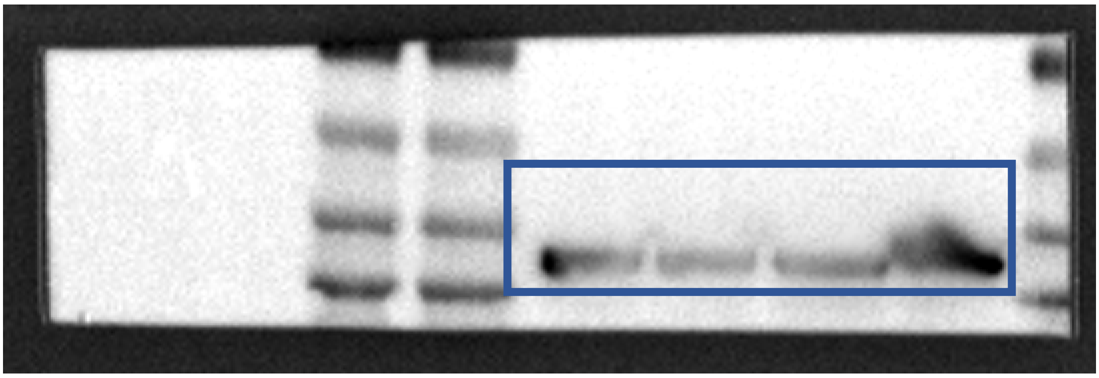

Figure S7F

H3K14la

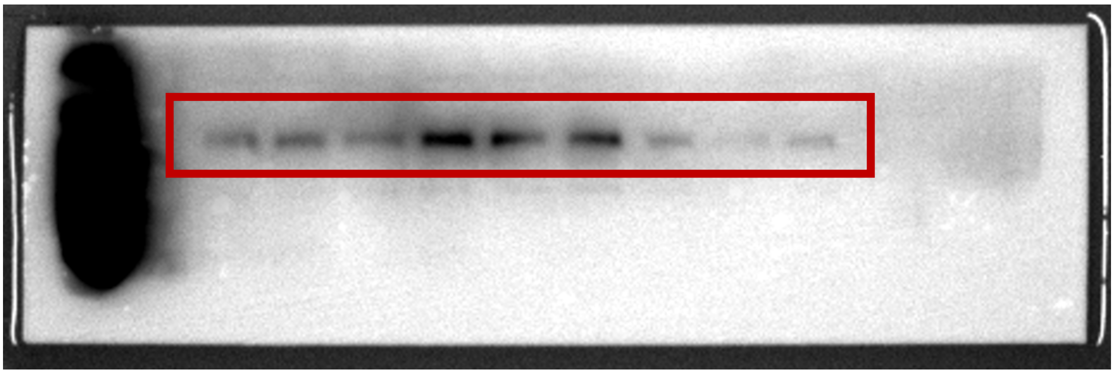

Histone H3

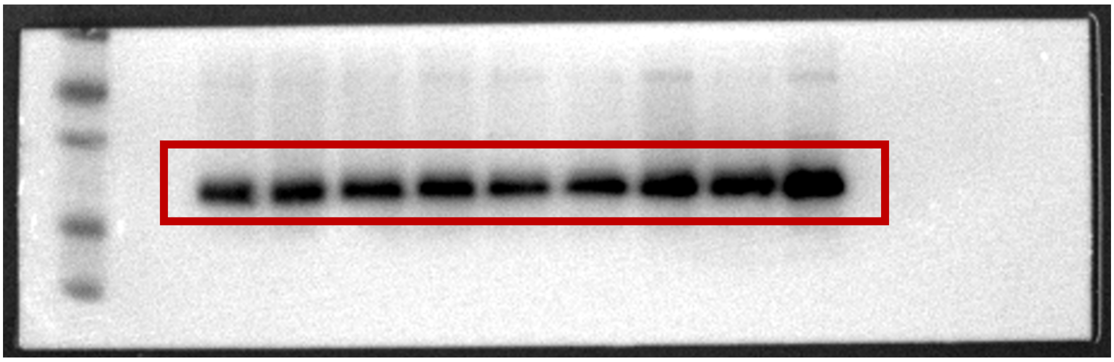

Supplement: Supplementary file 2 — Supporting Information [file ADVS-12-e08725-s001.pdf]
