# Supplementary material for: Metabolic Interplay in Acute Lung Injury: PARK7 Integrates FADS1/2‐Dependent PUFA Metabolism and H3K14 Lactylation to Attenuate Endothelial Ferroptosis and Dysfunction
Source: Adv Sci (Weinh). 2025 Sep 30;12(46):e08725. doi: 10.1002/advs.202508725 (PMC12697827; doi:10.1002/advs.202508725)
Supplement: Supplementary file 3 — Supporting Information [file ADVS-12-e08725-s003.pdf]

Figure 1E

	PBS	LPS
Fads1	63.69876	19.04987
	57.73094	12.71635
	55.24443	19.01933
Fads2	53.11252	14.03725
	44.67654	9.561219
	39.25887	15.26446
Scd1	942.9681	109.7708
	1080.916	72.51589
	1140.569	126.5882

Figure 1G

		PBS			LPS	
1	1850000000	1180000000	1340000000	1150000000	724000000	554000000
2	2100000000	1520000000	1600000000	1420000000	1020000000	776000000
4	44700000000	31100000000	31600000000	31000000000	20700000000	14400000000
5	10600000000	7450000000	7880000000	6480000000	4280000000	2670000000
6	23300000000	19500000000	19700000000	16500000000	10800000000	7500000000

Figure 1H

		PBS			LPS	
FA(14:1)-H	1.042949	0.978373	0.978677	0.316993	0.568404	1.02038
FA(18:1)-H	1.396987	0.648036	0.954977	0.827779	1.067466	0.71773
FA(19:1)-H	0.914087	0.824011	1.261902	0.21828	0.421963	0.752617
FA(20:1)-H	0.915695	0.80872	1.275585	0.395742	0.500972	0.791297
FA(18:3)-H	0.999963	0.850249	1.149788	0.510258	0.72207	0.998044
FA(18:4)-H	0.905196	0.891621	1.203183	0.587701	0.726014	0.893183
FA(20:3)-H	0.862542	0.89944	1.238018	0.419274	0.489028	0.68674
FA(20:4)-H	0.882071	0.868923	1.249006	0.401398	0.578652	0.864839
FA(20:5)-H	0.895994	0.888959	1.215048	0.279532	0.409337	0.705639
FA(22:3)-H	0.833558	0.870107	1.296335	0.31919	0.446103	0.645796
FA(22:4)-H	0.90862	0.792271	1.299109	0.515782	0.352851	0.639511
FA(22:5)-H	0.913842	0.854222	1.231935	0.315864	0.514811	0.758527
FA(22:6)-H	0.943532	0.937617	1.118851	0.359809	0.520063	0.791533

Figure 1J

		PBS				LPS		
Fads1	1.214896	0.871054	1.14671	0.824067	0.059989	0.148737	0.040879	0.04463
Fads2	1.262835	0.89296	1.074253	0.825496	0.046499	0.198425	0.022876	0.039373
Scd1	1.464086	0.827406	0.990801	0.833161	0.034355	0.055681	0.016708	0.021642

Figure 1L

PBS	LPS
0.989691	0.972509
0.869416	0.731959
1.075601	0.635739
1.113402	0.42268
0.986254	0.580756
0.965636	0.814433

Figure 1M

Control	SCAP	ARDS
229.1547898	45.82248993	57.03082
156.5568765	113.137766	110.4985258
182.5979625	140.8274477	134.5235013
253.3366042	138.3742102	95.48738
199.0106013	176.7746807	179.9199801
178.587145	185.4938432	193.4740561
122.8573113	84.10787418	38.6787082
346.2468202	143.1917926	128.639105
99.80784069	133.8630754	161.6337625
244.6983752	164.625254	57.03082
345.6153855	103.1867258	65.11102405
297.5574217	172.3065845	82.26872845
217.0129547	215.1050812	90.32120125
133.0069514	35.34304324	162.9132442
	128.4047139	66.95177005
	44.49448087	96.53292805
	145.3101363	66.95177005
	122.0883844	100.7561613
	64.3560728	101.8222298
	227.0113409	173.2968458
		164.1968301
		99.6941968
		20.9236858
		189.3647402
		86.26213205
		108.3048272
		57.03082
		142.9340282
		85.257625
		49.28304805
		39.57295321
		32.26897865
		110.6598966
		34.68834821
		26.01060659
		60.4847103
		42.37550579
		15.97610513
		119.9891529
		24.86514834
		82.53110714
		80.31102778
		49.32051485
		115.680437
		176.6638704
		107.4447534
		94.14364687

Figure 1Q

eicosapentaenoic acid		arachidonic acid		oleic acid	
Control	ARDS	Control	ARDS	Control	ARDS
0.392304638	0.116180098	0.663453122	0.170011433	0.535239282	1.650690874
0.404525493	0.16061197	0.730207733	0.266738803	0.566782915	1.436252177
0.464867913	0.187308555	0.742405413	0.305716491	0.642487973	2.178583191

0.488528232	0.20070555	0.75307182	0.409701626	0.662176143	1.31148145
0.493190206	0.234669699	0.806102234	0.531987003	0.681980877	1.701654429
0.587946274	0.328218334	0.85666278	0.544188999	0.699646732	1.42353561
0.594830842	0.333243267	0.902263824	0.54870563	0.72125461	1.20137009
0.617289442	0.340754884	0.910028861	0.592878731	0.769098377	1.362155134
0.706505781	0.36909592	0.920416982	0.621543778	0.814355433	0.688837346
0.715359682	0.445218927	0.939137657	0.661262773	0.837538414	3.083046654
0.725957707	0.452383883	0.945877698	0.676230419	0.848214546	1.522634891
0.744346343	0.45399316	0.948542757	0.679424345	0.880059241	0.270501986
0.782312239	0.506008469	0.950222748	0.705404177	0.884529692	1.661733178
0.798499053	0.533169523	0.958295213	0.707331415	0.889905105	0.716920252
0.829479813	0.535407812	0.960958444	0.715267367	0.890934336	1.663104957
0.933974324	0.556646065	0.992776387	0.754378536	0.891551841	1.211468594
0.965758253	0.572969209	1.008459824	0.802077523	0.977322715	1.039425037
1.107663151	0.615201849	1.011630091	0.8068383	1.074918577	1.443105294
1.211984597	0.622921761	1.064964462	0.813074774	1.090054479	0.25002109
1.218329671	0.62301794	1.112394755	0.820954008	1.164601111	1.424390606
1.288525378	0.642098491	1.113445213	0.838051361	1.166131525	2.293148683
1.456315012	0.647343708	1.125768478	0.859400188	1.168031471	1.888429093
1.617079355	0.660505312	1.348201741	0.883059398	1.239244698	1.48150072
1.622395611	0.687389905	1.361819315	0.893780182	1.411264271	0.426158517
1.931529612	0.70820196	1.414876784	0.924373835	1.426513823	0.623350409
1.957139369	0.744785628	1.458015667	0.925600407	1.571767397	1.898413026
2.343362008	0.804141804		0.990401122	1.582467746	2.082772191
	0.948195977		0.990577009	1.91192667	1.595637749
	0.983096777		1.010617186		0.493099147
	1.000603489		1.086202525		1.275215752
	1.039703463		1.225712044		1.436674009
	1.137067867		1.286971953		1.774650564
	1.259609676		1.305092425		1.213199928
	1.518215903		1.328515997		2.066356089
	1.763450169		1.459868276		0.64741979
	1.802844609		1.476434135		2.464831658
	1.810359303		1.648331819		0.994575019
	1.843604943		1.690888642		1.98785421
	0.496831707		1.881038607		0.364104392
	0.494754686		1.897858296		0.946527659
	0.516539745		2.045536972		2.205380845
	1.168854864				

Figure S1G

PBS	LPS
82600000000	56500000000
60800000000	37600000000
62100000000	25900000000

Figure S1H

	PBS				LPS			
FADS1	1.107461657	0.841504427	0.99642	1.054614	0.66813	0.585465	0.579639	0.537389
FADS2	1.04483931	0.935296376	0.82858	1.191284	0.541521	0.463562	0.440098	0.55567
SCD1	1.129695908	0.849701102	0.833192	1.187411	0.329834	0.374023	0.301225	0.362549

Figure S1I

	PBS				LPS			
FADS1	1.049111	1.018068	0.913304	1.025149	0.606747	0.467326	0.560907	0.467326
FADS2	0.843329	1.052753	1.195405	0.94224	0.304427	0.331596	0.45718	0.300236
SCD1	0.930342	0.981119	0.974342	1.124409	0.503187	0.387562	0.51853	0.342102

Figure S1K

	PBS				LPS			
FADS1	1.049111	1.018068	0.913304	1.025149	0.606747	0.467326	0.560907	0.467326
FADS2	0.843329	1.052753	1.195405	0.94224	0.304427	0.331596	0.45718	0.300236
SCD1	0.930342	0.981119	0.974342	1.124409	0.503187	0.387562	0.51853	0.342102

Figure 2A

Vehicle	LPS	LPS+Ferrostatin-1	PS+Ferrostatin-1+SC-261	LPS+DHA
102.06995	51.74875	62.45539	58.60099929	68.02284083
99.50035689	32.69094	78.94361	53.67594575	60.52819415
93.07637402	35.68879	57.3162	32.90506781	72.7337616
103.3547466	41.47038	84.93933	53.24768023	77.44468237
98.8579586	46.8237	70.59243	31.62027123	63.09778729
103.1406138	51.96288	55.60314	49.60742327	77.01641685

Figure 2B

Vehicle	LPS	LPS+Ferrostatin-1	PS+Ferrostatin-1+SC-261	LPS+DHA
2.873227	4.729512	3.201645	3.965471	3.698752
3.152848	4.775849	3.200679	4.047336	3.556257
2.857672	4.022022	3.526199	3.903424	3.498539

Figure 2C

Vehicle	LPS	LPS+Ferrostatin-1	PS+Ferrostatin-1+SC-261	LPS+DHA
1.36124	3.049284	1.690177	2.197217	1.952605
1.297594	2.447094	1.58054	2.199624	1.716259
1.394023	2.315085	1.490536	2.134335	2.024166

Figure 2D

Vehicle	LPS	LPS+Ferrostatin-1	PS+Ferrostatin-1+SC-261	LPS+DHA
48.40497	28.96507	42.41926	37.40073	41.13922
48.90546	32.53132	42.75482	32.39898	36.46537
51.73718	31.2699	47.03913	30.92736	40.71252

Figure 2F

Vehicle	Erastin	E-***FADS1***+LPS+Nigeri	E-***FADS2***+LPS+Nigericin
92.88113	48.15312	82.10208193	82.40429819
108.7979	41.20215	70.71860309	88.44862324
107.589	37.07186	73.13633311	86.13163197
98.92545	28.50907	75.35258563	73.33781061
102.1491	43.4184	72.83411686	79.38213566
89.65749	38.28073	55.40631296	76.05775688

Figure 2G

Vehicle	LPS+Nigericin	E-***FADS1***+LPS+Nigeri	E-***FADS2***+LPS+Nigericin
9.731088	14.34686	11.66527	10.89209
10.1338	14.89459	10.57963	9.91286
9.774169	13.57039	10.21873	10.53027

Figure 2H

Vehicle	LPS+Nigericin	E-***FADS1***+LPS+Nigeri	E-***FADS2***+LPS+Nigericin
1.207815	3.014267	1.527454	1.394565
0.80246	4.437326	1.274171	1.076602
0.99854	3.325043	1.230705	1.34824

Figure 2I

Vehicle	LPS+Nigericin	E-***FADS1***+LPS+Nigeri	E-***FADS2***+LPS+Nigericin
38.02043	20.95873	29.76714	31.41117

43.58119	24.18385	31.94782	33.27944
38.90126	24.47041	29.68831	31.16025

Figure 2K

ASCL4

Vehicle	LPS	LPS+Ferrostatin-1	PS+Ferrostatin-1+SC-261	LPS+DHA
1.115947807	1.353767275	1.088487899	1.667665198	0.903619831
0.998794	1.624289	0.822781	1.37367	1.234107
0.959673	1.953724	1.044216	1.269183	0.975398
0.904744	1.844617	1.076178	1.298538	1.065682
1.020842	1.850424	0.802032	1.675839	0.909095

GPX4

Vehicle	LPS	LPS+Ferrostatin-1	PS+Ferrostatin-1+SC-261	LPS+DHA
1.06353143	0.429563298	1.071220166	0.740608686	1.039399068
1.00362	0.470663	1.268112	0.84606	0.782148
1.078866	0.568123	0.920375	0.670944	1.036943
0.818592	0.53641	0.921843	0.728512	1.253166
1.035392	0.485949	0.889914	0.74251	1.14485

FTH1

Vehicle	LPS	LPS+Ferrostatin-1	PS+Ferrostatin-1+SC-261	LPS+DHA
1.117092336	0.259353408	0.923833797	0.651225703	0.588818519
0.966166	0.358853	1.060631	0.776078	0.604291
1.008991	0.202999	0.81636	0.339823	1.104168
0.974229	0.544421	0.737345	0.475585	0.712129
0.933522	0.52325	0.767366	0.498973	0.67436

Figure 2L

ASCL4

Mock	LPS+Nigericin	E-***FADS1*** +LPS+Nigeri	DE-***FADS2*** +LPS+Nigericin
0.914194114	1.546194084	0.879545809	0.901831784
1.054646	1.381942	1.069834	0.695668
1.031160092	1.211162783	1.068294161	0.911713861

GPX4

Mock	LPS+Nigericin	E-***FADS1*** +LPS+Nigeri	DE-***FADS2*** +LPS+Nigericin
1.092552834	0.637262404	0.853612663	1.067065286
0.995952	0.470499	0.985156	1.241218
0.911495279	0.444059375	1.060602853	1.318297871

FTH1

Mock	LPS+Nigericin	E-***FADS1*** +LPS+Nigeri	DE-***FADS2*** +LPS+Nigericin
1.179945632	0.67542311	0.872524493	0.963548996
0.906814141	0.505283271	1.234189997	1.103761868
0.913240227	0.6738947	1.054278811	1.083452446

Figure 2M

DMSO	DHA
0.706235828	4.184140929
1.356197474	3.089546088
0.945156092	2.944502443
0.992410606	5.980316933

Figure 2N

Vector	OE-*FADS1*	OE-*FADS2*
0.697396	3.794791	4.018276
1.124586	2.828059	3.701696
1.178017	2.418749	3.917531

Figure 2O

Vehicle	LPS	LPS+DHA	LPS+DHA+ML385
0.998551	0.565973	0.981809	0.69387
1.003927	0.754245	0.885324	0.566213
0.85816	0.563739	1.355927	0.619467
1.139361	0.625453	0.879555	0.540383

Figure 2P

Mock	LPS+Nigericin	E-*FADS1*+LPS+Nigeri	DE-*FADS2*+LPS+Nigerici	*FADS1*+LPS+Nigericin+ML	*ADS2*+LPS+Nigericin+ML385
1.037475	0.702181	1.231428	0.958934	0.337563	0.290033
0.992881	0.542861	1.226148	1.177311	0.511316	0.49513
1.009974	0.571613	1.017236	0.914287	0.627775	0.541491
0.95967	0.507309	1.177826	0.97394	0.560489	0.527094

Figure 2Q

MPMEC

DHA(μm)			DMSO						Erastin			
0	103.4862	90.19856	99.9226	98.98431	103.2682	104.1401	53.76657	55.70948	53.68127	55.05552	55.05552	52.82828
5	102.2541	88.84326	105.1353	112.5278	116.3378	90.1796	60.21135	62.95038	51.86156	59.35836	63.32949	51.40664
10	104.9931	106.8507	100.7566	103.6378	77.97242	103.2113	68.39054	67.52808	67.13002	65.63255	61.17807	66.73196
20	107.2014	103.2208	100.7945	106.6233	108.3008	116.0819	73.30943	74.45622	63.98345	68.86442	80.48399	71.68875
50	35.85386	64.71322	27.88317	57.36806	63.17785	58.96978	25.14414	27.7031	39.97662	42.49767	55.84217	53.22634
100	16.56689	22.89794	12.96539	10.79501	10.82345	11.54375	6.46374	3.933214	3.961647	3.525676	4.975753	7.458891

HUVEC

DHA(μm)			DMSO						Erastin			
0	108.9186951	104.5822535	103.8368023	101.5844177	104.7505811	76.32725	47.39092	48.36080904	53.07398	40.67385	52.40869	41.55556
5	137.398135	125.4067919	115.3632404	123.1143292	111.6359847	97.53654	54.08395	59.5185294	62.05948	60.08764	53.93967	49.89179
10	121.863895	122.7616427	124.6132471	129.5829214	120.6134609	119.4672	66.82876	63.55839367	71.15719	70.34761	66.60432	64.39202
20	82.76378016	89.63315254	99.82098485	82.92409223	94.5226708	83.68557	42.37315	44.51331926	48.11233	49.92385	43.12662	38.66193
50	38.91842787	43.87207096	37.17102626	38.78216261	36.65802763	36.16106	24.47431	17.82937452	16.81139	20.62682	23.59259	23.02349
100	21.88526999	37.06682342	20.08977476	18.57482566	18.46260721	17.03583	22.30208	5.076549015	15.53691	15.06399	11.92187	7.425121

LLC

DHA(μm)			DMSO						Erastin			
0	100.7018706	99.70062687	100.161874	99.05188093	98.51938425	101.8644	74.55328	59.42212861	62.90586	67.17333	65.36584	59.93213
5	94.94190661	94.94565659	98.97313142	98.61313367	99.51687802	100.8069	66.15709	63.839601	62.04336	65.85709	70.39831	68.71457
10	96.83939475	98.18188636	96.54314661	97.95688777	95.89440066	97.15439	68.93207	71.3920538	84.93697	65.53834	61.63836	71.11081
20	95.6319023	95.89065068	99.80937619	96.54689658	98.1593865	95.89065	72.5283	69.82831357	63.90335	68.27582	70.08331	67.07958
50	97.99813751	97.94188786	98.46313461	98.65063343	97.31939175	97.23689	69.30332	70.02706233	69.92956	71.15956	73.06829	71.43705
100	87.05195593	82.05698714	68.47082206	83.680727	81.00324373	72.69705	55.6234	60.79087006	60.99337	59.75588	57.75339	58.96838

A549

DHA(μm)			DMSO						Erastin			
0	92.66629192	117.9569046	80.61907439	100.513397	99.042908	109.2014	88.23234	81.16769721	85.42627	86.75286	74.10755	89.82874
5	108.4909125	117.7140716	110.1457748	105.8422335	116.9316095	116.0997	91.68597	91.60502155	92.19412	94.18175	93.94791	91.77141
10	109.4262694	109.9973768	116.3964774	116.8956343	112.2368372	113.9547	62.28068	90.04459434	87.76016	76.96758	74.85404	57.5949
20	115.8478546	109.646618	110.3256511	116.6213228	116.0951846	100.3695	37.46674	64.52913622	74.74162	61.69608	57.23515	44.11767
50	115.9198051	113.5004684	115.0114296	116.9136219	114.2649429	115.5556	41.75679	72.6820311	44.82368	66.88102	58.55274	11.62751

100	105.4510024	96.15139592	99.65448754	93.02154769	77.17444257	59.16882	25.21716	23.64324527	18.44482	21.83549	19.57804	9.756792

Figure S2A

Vector	OE-***FADS1***
0.765779	9.769829
1.155353	5.247632
1.258466	9.883349
0.898132	10.86538

Figure S2B

Vector	OE-***FADS2***
0.878126	7.794735
1.039459	6.960383
1.241858	7.758799
0.882193	8.106992

Figure S2E

NC	siRNA1	siRNA2	siRNA3
1.140764	0.057645	0.092569	0.093428
0.878633	0.065759	0.077482	0.108317
0.997692	0.0965	0.106826	0.137738

Figure S2F

NC	siRNA1	siRNA2	siRNA3
0.886791	0.192999	0.123279	0.122995
0.92445	0.192109	0.149685	0.128514
1.219819	0.212667	0.148308	0.133662

Figure S2I

NC	siRNA1	siRNA2	siRNA3
1.006956	0.127332	0.107818	0.034276
1.009285	0.109576	0.115023	0.028426
0.983957	0.121301	0.120742	0.04133

Figure S3A

Vehicle	LPS	LPS+DHA	LPS+DHA+ML385
112.1969	57.75165	75.60617	67.67083
117.2667	63.04188	79.57384	57.75165
81.99853	59.29464	80.23512	51.57972
99.19177	58.85378	73.62234	73.62234
91.47686	61.93975	74.06319	61.71932
97.86921	60.83762	100.0735	67.22998

Figure S3B

Mock	LPS+Nigericin	E-***FADS1*** +LPS+Nigeric	E-***FADS2*** +LPS+Nigeric	***FADS1*** +LPS+Nigericin+ML	-***FADS2*** +LPS+Nigericin+ML385
105.5245536	51.11607	72.71205	56.47321	40.5692	46.76339
94.97767857	40.5692	64.34152	75.89286	50.78125	49.77679
94.140625	44.92188	59.48661	65.84821	46.59598	46.26116
107.5334821	48.4375	65.34598	62.33259	50.44643	39.22991
98.828125	40.23438	65.17857	67.52232	50.94866	56.3058
98.99553571	34.54241	63.00223	65.84821	44.25223	40.23438

Figure S3D

0μm	5μm	10μm	20μm	50μm	100μm
0.900924815	1.342515673	1.484691698	2.032131443	0.900756551	0.633707737
0.955200064	1.492631877	1.730002529	1.845835782	1.052972342	0.520331704
1.136229175	1.14372578	1.570560397	1.740523124	1.440061096	0.500979847
1.007645946	1.845312151	1.642353785	1.62825386	0.971821239	0.286338582

Figure 3B

ZO-1

Vehicle	LPS	S+Ferrostati	rostatin-1+S	LPS+DHA
1.097025	0.527102	0.788215	0.529483	0.993118
0.935621	0.384487	0.871988	0.562063	1.133531
1.157699	0.574363	0.872825	0.497634	0.841605
0.809655	0.664603	0.810319	0.674912	0.912903

VE-cadherin

Vehicle	LPS	S+Ferrostati	rostatin-1+S	LPS+DHA
1.107451	0.499748	0.888787	0.534149	0.991078
0.712148	0.501248	1.029259	0.481886	1.46212
1.285562	0.406285	0.729724	0.547478	1.136425
0.894839	0.318326	0.994716	0.67249	1.316614

Occludin

Vehicle	LPS	S+Ferrostati	rostatin-1+S	LPS+DHA
1.00574	0.657326	0.820808	0.684599	1.28003
0.994225	0.715887	1.015938	0.462537	1.195238
1.027644	0.723857	0.875659	0.610661	1.156423
0.972391	0.594336	0.948518	0.628083	1.2012

Figure 3D

ZO-1

Mock	PS+Nigerici	***DS1*** +LPS+N	***DS2*** +LPS+Nigericin
0.947389	0.622374	0.734941	0.8386
1.069485	0.487106	0.779984	0.886514
1.139184	0.579175	0.776114	0.671886
0.843942	0.433589	0.914677	0.971759

VE-cadherin

Mock	PS+Nigerici	***DS1*** +LPS+N	***DS2*** +LPS+Nigericin
1.145099	0.523923	0.935337	0.83836
1.062761	0.512245	0.884075	0.998334
1.052965	0.55899	0.822746	1.083949
0.739174	0.682219	1.034299	0.988518

Occludin

Mock	PS+Nigerici	***DS1*** +LPS+N	***DS2*** +LPS+Nigericin
0.968331	0.692491	1.117274	1.094786
1.055018	0.727459	1.114435	1.018513
1.129717	0.741522	1.040026	0.956147
0.846934	0.609529	1.21189	1.234767

Figure 3F

Piezo1

MOCK	PS+Nigerici	***DS1*** +LPS+N	***DS2*** +LPS+Nigericin
0.833845	0.599855	0.985019	1.121129
1.109694	0.676718	0.808198	0.96772
1.240855	0.492964	0.922504	0.958551
0.914067	0.69812	1.053797	0.840496
0.901538	0.516625	0.801463	1.420941

Piezo2

Mock	PS+Nigerici	***DS1*** +LPS+N	***DS2*** +LPS+Nigericin
0.997937	0.559088	0.821371	0.923911
1.201313	0.52235	0.781491	0.86342

0.85298	0.525386	0.948778	1.057085
0.939473	0.643589	0.86782	0.831078
1.008298	0.53444	0.772681	1.049735

Figure 3G

Nodes

Mock	PS+Nigerici	*DS1* +LPS+N	*DS2* +LPS+Nigericin
76	33	102	76
36	12	68	109
49	36	76	58
56	12	79	89
79	45	30	62
58	26	57	82

Lengths

Mock	PS+Nigerici	*DS1* +LPS+N	*DS2* +LPS+Nigericin
3884	2946	4860	3858
3644	2934	3817	4004
3689	3382	4418	4144
3995	1953	4677	4201
4179	3210	3309	4133
3531	3333	4012	4248

Figure 3H

Tie2

Mock	PS+Nigerici	*DS1* +LPS+N	*DS2* +LPS+Nigericin
0.93368	0.790589	1.223488	1.338855
1.014663	0.75054	0.993781	1.054091
0.983502	0.844401	0.901875	1.219255
1.098854	0.901875	1.014663	1.028827
0.976709	0.73255	0.91764	1.165541

VEGFR

Mock	PS+Nigerici	*DS1* +LPS+N	*DS2* +LPS+Nigericin
0.94737	0.681601	0.984184	1.174462
0.896267	0.622869	0.915099	0.819037
1.011853	0.802181	0.883928	1.194991
1.158293	0.780245	0.862741	0.905634
1.004864	0.743291	0.856782	0.96393

Figure 4B

Fads1

AAV-Vector	AAV-Tie2-Fads1
0.772442795	1.663436353
0.715736272	1.675006468
1.360528018	2.23586539
1.329453515	3.075526343

Fads2

AAV-Vector	AAV-Tie2-Fads2
1.093661795	3.488241681
1.137473814	3.107670042
0.819320604	2.322749114
0.981118975	2.661980494

Figure 4E

			Vector-PBS			
Day1	0.897959184	0.886178862	0.930041152	0.926829268	0.882591093	0.930327869
Day2	0.893877551	0.882113821	0.942386831	0.922764228	0.870445344	0.93442623
Day3	0.906122449	0.914634146	0.967078189	0.922764228	0.947368421	0.950819672
			Vector-LPS			
Day1	0.879032258	0.86381323	0.866666667	0.852140078	0.851405622	0.864
Day2	0.798387097	0.789883268	0.796078431	0.785992218	0.803212851	0.796
Day3	0.741935484	0.73540856	0.737254902	0.739299611	0.767068273	0.752
			EC-*Fads1*-LPS			
Day1	0.897540984	0.909465021	0.920833333	0.933609959	0.905349794	0.912133891
Day2	0.844262295	0.839506173	0.870833333	0.863070539	0.835390947	0.841004184
Day3	0.823770492	0.802469136	0.825	0.804979253	0.802469136	0.807531381
			EC-*Fads2*-LPS			
Day1	0.886554622	0.9125	0.897959184	0.914979757	0.91322314	0.892561983
Day2	0.848739496	0.85	0.848979592	0.854251012	0.867768595	0.847107438
Day3	0.827731092	0.8125	0.820408163	0.829959514	0.838842975	0.818181818

Figure 4G

Vector-PBS	Vector-LPS	EC-*Fads1*-LPS	EC-*Fads2*-LPS
1	6.666666667	4	3.666666667
0.333333333	5	3.333333333	4.333333333
0.666666667	5.333333333	3.666666667	3
1	6	3	3.666666667
0.666666667	5.333333333	3.333333333	3.333333333
2	6	2.333333333	3.666666667

Figure 4H

Vector-PBS	Vector-LPS	EC-*Fads1*-LPS	EC-*Fads2*-LPS
3.846153846	6.316666667	4.939393939	5.761904762
4.196428571	5.862745098	4.414634146	5.85
3.288888889	6.351351351	4.702702703	4.684210526
4.62	5.653061224	5.645833333	4.089552239
4.246376812	6.338709677	4.703125	4.879310345
4.711864407	6.279069767	5.375	5.795454545

Figure 4I

Vector-PBS	Vector-LPS	EC-*Fads1*-LPS	EC-*Fads2*-LPS
177000	8540000	1730000	1540000
151000	6580000	1550000	2110000
135000	7480000	1960000	1730000
201000	4270000	2010000	2150000
166000	5750000	1700000	902000
173000	5610000	1810000	1930000

Figure 4J

Vector-PBS	Vector-LPS	EC-*Fads1*-LPS	EC-*Fads2*-LPS
0.066025	2.6569	1.292725	1.588825
0.827425	3.22795	1.53595	1.483075
0.425575	3.10105	1.165825	1.45135
0.56305	2.2339	1.081225	1.250425
0.7111	3.18565	1.567675	1.1764
0.573625	3.27025	1.28215	1.419625

Figure 4K

TNF-α

Vector-PBS	Vector-LPS	EC-*Fads1*-LPS	EC-*Fads2*-LPS
101.5185957	178.0791524	160.3946622	125.7638669
116.563708	178.4118911	120.9646767	137.4318439
122.7014842	140.9763569	141.8237823	117.0965817
152.5079456	177.5391417	128.9013643	130.8388747
115.0058733	177.2072519	135.1911876	132.7203932
96.18951252	172.2677163	126.2492336	123.043576

IL-6

Vector-PBS	Vector-LPS	EC-*Fads1*-LPS	EC-*Fads2*-LPS
149.1545	175.1702375	163.30955	157.8264125
145.1490875	191.5029875	174.4702625	170.4259625
150.9822125	183.84215	163.853975	163.1928875
96.5397125	178.9812125	149.387825	178.009025
136.3994	213.3577625	152.2655	158.7597125
135.2716625	205.93025	159.3041375	142.9325

Figure 4L

IL-1β

Vector-PBS	Vector-LPS	EC-*Fads1*-LPS	EC-*Fads2*-LPS
66.07372	250.0687	152.7013	152.7013
65.07508	227.5993	125.2387	170.1775
68.071	270.0415	172.6741	200.1367
75.06148	364.9123	207.6265	135.2251
81.05332	230.0959	145.2115	130.2319
69.06964	250.0687	130.2319	200.1367

TNF-α

Vector-PBS	Vector-LPS	EC-*Fads1*-LPS	EC-*Fads2*-LPS
291.976903	532.4989855	406.7156495	338.247898
271.9509268	561.6407223	420.0132926	385.4992615
179.6625419	496.8698859	352.4408923	356.777256
179.4120479	516.206778	381.9410867	364.5968638

215.1034601	504.8249674	379.6423293	332.0682969
229.1339721	461.8824997	421.3250517	373.642714

IL-6

Vector-PBS	Vector-LPS	EC-*Fads1*-LPS	EC-*Fads2*-LPS
259.9449875	569.8514375	501.5066563	351.2064688
231.4015625	546.713375	455.2305313	342.165125
174.3536	515.8950313	443.3698438	394.5660313
224.59625	647.3347813	452.3139688	431.8980313
190.608575	575.2956875	378.9138125	333.7070938
163.9706375	520.7559688	416.3430313	434.1340625

Figure 4N

Vector-PBS	Vector-LPS	EC-*Fads1*-LPS	EC-*Fads2*-LPS
12.57666667	39.24	24.28333333	21.13666667
12.96	31.77333333	19.1	22.00333333
9.61	28.19	22.08	22.04666667
12.64333333	32.3	16.94	18.36666667
14.36333333	35.67666667	21.2	21.75666667
12.61333333	37.52	14.58666667	18.32

Figure 5B

Fads1

MOCK	FADS-UP
0.838956	6.025626
0.747425	2.072125
1.238753	1.580083
1.341022	2.248385
0.772592	3.694952
1.242575	1.850324

Fads2

MOCK	FADS-UP
1.09767	1.557129
0.986993	1.627009
0.868541	1.726413
1.181903	1.628263
0.857904	1.484524
1.048101	1.56796

Figure 5G

0	4	8	12	24
12.38255172	20.441	28.24307	18.87017241	16.02751724
11.19034483	23.969	40.9479	16.29317241	10.78141379
11.69593103	22.332	30.19297	20.38106897	17.96810345
10.89648276	22.975	32.1719	15.90810345	7.387
10.84793103	22.479	30.92962	14.74955172	7.822448276
9.629206897	46.438	43.99828	16.09413793	11.20096552
9.629413793	48.729	44.95172	16.68458621	11.64665517
12.01917241	23.402	41.68969	16.27534483	10.21517241

Figure 5H

Mock	LPS	LPS+ALA	FADS-UP+LPS	FADS-UP+LPS+ALA
2	7.333333	7	5.666667	3.333333
1.666667	7.666667	6.333333	5.666667	2.666667
2.666667	7	9.333333	5	4
2	8.666667	6.666667	5.666667	4.333333
1	8.666667	6.666667	6	4.666667
1.333333	10	7.333333	4.666667	4.333333

Figure 5I

Mock	LPS	LPS+ALA	FADS-UP+LPS	FADS-UP+LPS+ALA
4.22449	6.315789	6.560976	5.478261	4.4
4.207547	6.57377	5.736842	5.857143	4.87037
4.230769	7.457627	5.061538	5.765957	5
3.571429	6.444444	6.333333	5.235294	4.480769
3.877193	7.74359	6.55102	4.942308	4.259259
3.86	6.93617	7.784946	5.982456	4.701754

Figure 5J

Mock	LPS	LPS+ALA	FADS-UP+LPS	FADS-UP+LPS+ALA
156000	2340000	2540000	1120000	790000
169000	1670000	2360000	920000	760000
239000	2140000	1980000	1810000	614000
96200	2540000	1990000	1550000	555000

59800	2250000	2210000	1170000	749000
212000	2840000	1640000	829000	811000

Figure 5K

Mock	LPS	LPS+ALA	FADS-UP+LPS	FADS-UP+LPS+ALA
0.2648837	4.000206	3.908203	2.0681425	1.0009077
0.550093	4.625826	4.625826	1.654129	0.7985011
0.550093	3.917403	2.877769	2.114144	1.3505191
0.826102	4.690228	4.690228	2.2889497	1.5713263
0.642096	4.598225	4.598225	1.4149212	1.1573128
0.3476864	4.009406	3.089376	2.758165	1.2125146

Figure 5L

Mock	LPS	LPS+ALA	FADS-UP+LPS	FADS-UP+LPS+ALA
220.5435195	561.5348	511.8413	434.6807368	394.7667259
223.5211952	547.3402	613.0559	455.8485482	393.3254646
212.1048319	588.1626	479.2286	418.0484869	365.5801059
228.4807228	513.0909	507.4916	427.8455317	351.1634636
219.4705912	522.5606	444.8809	454.7383612	346.9080722
215.2308219	544.6774	542.0276	403.5258795	385.727138

Figure 5M

Mock	LPS	LPS+ALA	FADS-UP+LPS	FADS-UP+LPS+ALA
120.1942	331.9113	289.0102	244.1028	230.0438
142.5102	271.2785	320.6724	254.3431	223.5897
130.4956	259.1915	318.4754	266.9069	223.5897
138.8621	298.957	314.1324	270.6186	204.3862
133.684	315.7531	355.66	251.3059	240.7978
127.3902	288.524	294.9275	278.686	225.0863

Figure 5N

Mock	LPS	LPS+ALA	FADS-UP+LPS	FADS-UP+LPS+ALA
316.7965	722.5774	471.6688	1009.245	540.1493
326.5062	968.6083	459.1596	845.8292	533.3434
355.0332	780.0673	425.0276	963.3944	527.0901
349.8303	651.8728	497.0222	909.0224	671.2511
324.6684	811.6709	454.7974	807.0962	492.2051
319.2027	706.0229	490.2938	889.2188	578.2123

Figure 5O

Mock	LPS	LPS+ALA	FADS-UP+LPS	FADS-UP+LPS+ALA
5.048761	9.719779	8.605479	13.48416	8.551058
5.372926	11.19935	6.874386	13.27762	8.461058
6.490462	11.45068	7.641752	12.25441	9.96508
4.534366	10.99688	7.481252	11.51997	10.30002
4.443604	11.63614	8.551058	12.77112	10.34252
4.354248	8.733692	7.386638	10.99688	8.938648

Figure 5P

Mock	LPS	LPS+ALA	FADS-UP+LPS	FADS-UP+LPS+ALA
1.098526	0.693561	0.828549	0.851823	1.070597
1.01474	0.688906	0.730799	0.90768	1.200931

0.903026	0.726144	0.633049	0.977502	1.200931
0.912335	0.60512	0.777347	0.758728	1.186967
0.884407	0.40962	0.535299	0.949573	0.889061
1.186967	0.470132	0.591156	0.856478	1.335919

Figure 5Q

Mock	LPS	LPS+ALA	FADS-UP+LPS	FADS-UP+LPS+ALA
2.534507	5.741362	6.260069	2.889544	3.289288
1.953071	5.37899	5.910291	3.585485	2.519332
2.020445	5.737189	3.329244	4.151426	2.637669
2.431657	4.881373	6.610418	3.725228	2.91769
1.602979	4.666906	4.318077	3.073771	2.071965
2.539149	4.168732	4.626374	3.492744	2.224075

Figure 5R

Mock	LPS	LPS+ALA	FADS-UP+LPS	FADS-UP+LPS+ALA
1.033123	0.386056	0.494615	0.833036	1.230333
1.231701	0.470194	0.576003	0.665179	0.975587
0.91081	0.339175	0.279633	0.591568	0.874917
1.310773	0.103499	0.593334	0.738735	0.919461
0.957757	0.507911	0.136666	0.701046	0.913784
0.976962	0.571995	0.635156	0.733481	1.03166

Figure 5S

ZO-1

Mock	LPS	LPS+ALA	FADS-UP+LPS	FADS-UP+LPS+ALA
1.067573	0.187141	0.263358	0.716292	1.04256
1.224168	0.222269	0.243049	0.772352	0.818031
0.824388	0.35218	0.234736	0.773527	0.97085
0.856553	0.166595	0.46375	0.644873	1.047082
0.791173	0.247209	0.223372	0.670098	0.90519
1.236144	0.188396	0.306345	0.590637	1.085654

VE-cadherin

Mock	LPS	LPS+ALA	FADS-UP+LPS	FADS-UP+LPS+ALA
1.046576	0.437076	0.491793	0.714265	0.991083
0.843212	0.409443	0.49071	0.663835	1.134611
0.910143	0.43963	0.497172	0.635282	1.048213
1.255588	0.604639	0.364428	0.462244	0.861073
0.852881	0.392297	0.454257	0.760803	1.199116
1.0916	0.472705	0.309534	0.698286	1.031392

GPX4

Mock	LPS	LPS+ALA	FADS-UP+LPS	FADS-UP+LPS+ALA
1.170194	0.495047	0.33023	1.024247	1.307991
1.023458	0.542826	0.554265	0.835969	1.1338
0.824379	0.551698	0.677592	0.87371	1.173012
1.056114	0.517504	0.543804	0.879887	1.077358
0.839887	0.565173	0.573351	0.779775	1.558259
1.085968	0.665623	0.495871	0.835673	1.069155

Figure 6A

Control	SCAP	ARDS
17.59586	56.82744	90.9343
20.96711	44.22934	82.17125
18.10292	41.1875	74.02381
20.8641	34.68898	28.84562
23.35121	37.4683	25.0264
18.20449	32.96545	23.76872
53.33495	57.37286	28.9527
25.65812	34.14163	22.41493
26.35597	34.59755	21.27648
28.2224	47.8569	56.67828
14.93663	42.6499	37.02492
31.98062	42.16046	67.45884
20.82827	29.08339	42.98634
27.28441	61.35807	27.13963
	34.41495	40.94486
	50.95792	61.5272
	26.69248	27.13963
	34.59755	34.92438
	51.37675	47.585
	48.06143	29.05983
		50.62
		29.05983
		27.77777
		22.93455
		33.60767
		33.06131
		116.4502
		73.64113
		94.85839
		101.0365
		60.12953
		67.06262
		68.69522
		79.18706
		64.52833
		59.79613
		59.57425
		76.11106
		63.84379
		110.7956
		44.32873
		60.57517
		35.79219
		33.41625
		44.82684
		64.6427
		61.24599

Figure 6C

Vehicle	Erastin	PS+Nigericin
0.674957	3.787602	7.091011
0.669953	6.640429	8.423817
1.65509	5.351844	8.102935

Figure 6D

NC	si*PARK7*
1.055188	0.007826
1.047899	0.009084
1.123111	0.009736
0.805245	0.009769

Figure 5H

PARK7

NC	si*PARK7*
416.12	8.81
430.76	7.68
419.6	10.67

FADS1

NC	si*PARK7*
35.07	29.05
36.6	27.89
36.64	26.31

FADS2

NC	si*PARK7*
76.17	53.66
76.63	55.83
79.25	50.58

Figure 6I

PARK7

Vector	OE-PARK7
0.902814	2.607198
1.270885	2.583213
0.921784	2.212739
0.945511	2.518387

FADS1

Vector	OE-PARK7
1.012204	1.718123
0.948794	1.726081
0.989085	1.599366
1.052753	1.35113

FADS2

Vector	OE-PARK7
1.042466	1.271619
1.079228	1.674039
0.911722	1.781797
0.974905	1.848899

Figure 6J

PARK7

NC	SiPARK7
1.294594	0.027632
0.840411	0.02158
0.921784	0.026629
0.997116	0.027696

FADS1

NC	SiPARK7

0.991373	0.507273
1.067449	0.655575
0.950989	0.727406
0.993666	0.62887

FADS2

NC	SiPARK7
0.812252	0.468677
1.112136	0.423373
0.961483	0.511687
1.151355	0.549681

Figure 6K

Mock	LPS+Niger	OE-PARK7	siFADS1+C	siFADS2+OE-PARK7+LPS+Nigericin
32.64469	18.95046	40.0281	24.3902	28.8855
33.60419	20.26962	51.32257	31.36271	27.11571
35.44972	24.46811	44.02649	23.63887	20.91898

Figure 6L

Mock	LPS+Niger	OE-PARK7	siFADS1+C	siFADS2+OE-PARK7+LPS+Nigericin
1.314051	3.237346	0.669707	3.328536	3.781484
1.351669	2.851389	1.665122	2.792993	3.059628
0.881728	3.121932	1.267622	2.358105	3.330988

Figure 6M

zo-1

Mock	LPS+Niger	OE-PARK7	siFADS1+C	siFADS2+OE-PARK7+LPS+Nigericin
0.914892	0.508587	0.898982	0.512098	0.525524
1.084382	0.329205	0.873463	0.580475	0.536721
0.867183	0.571592	0.974858	0.593572	0.375417
1.133543	0.676941	0.849508	0.385453	0.329162

VE-cadherin

Mock	LPS+Niger	OE-PARK7	siFADS1+C	siFADS2+OE-PARK7+LPS+Nigericin
0.968467	0.582627	1.000504	0.555447	0.497238
0.924345	0.62859	0.985563	0.49264	0.493964
0.999234	0.446617	0.961225	0.639834	0.521176
1.107954	0.488233	0.986689	0.618407	0.401722

GPX4

Mock	LPS+Niger	OE-PARK7	siFADS1+C	siFADS2+OE-PARK7+LPS+Nigericin
0.871132	0.528823	1.06957	0.741776	0.565777
0.90918	0.409871	1.266119	0.60393	0.539252
1.02828	0.434547	1.140749	0.67209	0.431418
1.191408	0.503231	1.155561	0.574891	0.308223

Figure 7A

NC	si***PARK7***
0.824861	0.172407
1.390454	0.484832
0.871893	0.241577

Figure 7E

BMP2

NC	si***PARK7***
0.934111	0.612027
1.138131	0.627056
0.940609	0.612027

BMP4

NC	si***PARK7***
0.895025	0.406126
1.160704	0.463294
0.962594	0.488016

BMPR1A

NC	si***PARK7***
0.911722	0.547779
0.807574	0.398228
1.358172	0.262733

BMPR1B

NC	si***PARK7***
1.009285	0.23138
1.35035	0.217386
0.733736	0.250578

Figure 7I

Mock	OE-***PARK7***	si***PARK7***	***RK7***+BMP agonist
1.057617	1.209883498	0.726560917	1.244773492
0.918636	1.692312415	0.694224472	1.097122585
1.02488	1.695645	0.644412	0.996159
0.998867	1.540226	0.734286	1.076889

Figure 7J

Mock	OE-***PARK7***	si***PARK7***	***RK7***+BMP agonist
1.045286	1.24663735	0.823299595	1.133998786
0.971686	1.557205294	0.628530065	1.253160474
0.9839	1.834341	0.421302	1.252171
0.999128	1.386817	0.562874	1.249225

Figure 7K

FADS1

Mock	OE-***PARK7***	si***PARK7***	***RK7***+BMP agonist
1.190368	1.378733138	0.285606775	1.013778695
0.889376	1.220901818	0.578374868	1.105626489
1.002528	1.251991	0.612933	1.042408
0.917728	1.185383	0.676363	1.106095

FADS2

Mock	OE-***PARK7***	si***PARK7***	***RK7***+BMP agonist
1.044118	1.269384815	0.653316237	1.144564033
1.005216	1.153462836	0.760407103	1.154941298

1.033281	1.298919	0.663966	1.111298
0.917385	1.28751	0.675908	1.214072

Figure 7L

FADS1

IgG	nti-pSAMD1/5/9
0.006992	0.007678
0.003734	0.008032
0.002108	0.007705
0.002197	0.005921

FADS2

IgG	nti-pSAMD1/5/9
0.015303	0.046552
0.009355	0.030081
0.003484	0.040526
0.006848	0.078836

Figure 7M

	NC				siPARK7			
preBMP2	0.852634892	0.989656656	1.049716684	1.128964405	0.574349177	0.491410299	0.547146851	0.510506063
preBMP4	0.946057647	0.952637998	0.996540263	1.113421618	0.512278412	0.404721108	0.539614118	0.514056913
preBMPR1/	1.411765039	0.977724561	0.82597308	0.877112215	0.875087995	1.102541785	1.231855756	1.305105711
preBMPR1I	1.1076484	1.022782939	0.934651216	0.944419673	1.096191612	0.608150374	0.947698461	0.785944857

Figure 7P

	Mock				siPARK7				siPARK7+OE-NFE2L2			
BMP2	1.001155913	0.986232704	0.910669834	1.112136086	0.565134695	0.468136124	0.341510064	0.422395587	0.627056205	0.938437997	0.716149817	0.810377861
BMP4	1.090507733	0.948246031	0.931955732	1.037659659	0.578344092	0.507565764	0.50697974	0.440332937	0.764894847	1.327151742	0.863539055	0.87458267

Figure 7Q

	IgG			Anti-NRF2		
BMP2	0.001816025	0.004100456	0.00191293	0.01903766	0.0238478	0.019572882
BMP4	0.007365504	0.002115186	0.00364466	0.009551877	0.009005362	0.01457864

Figure 7R

BMPR1A

	NC				si*PARK7*			
0	1.02663	0.816722	1.20546	0.989371	1.0811	0.952088	0.941152	1.032279
1	0.636361	0.677323	0.570876		0.400766	0.402158	0.382668	
2	0.390822	0.443268	0.367186		0.301278	0.415379	0.380025	
4	0.441224	0.29211	0.299629		0.380464	0.354986	0.337783	
8	0.298248	0.293802	0.293802		0.274682	0.278195	0.215511	

BMPR1B

	NC				si*PARK7*			
0	1.062527367	0.851159	1.079852	1.023965	1	1.049717	0.933033	1.021012
1	0.437543997	0.548729	0.589474		0.289506304	0.383332	0.311003	
2	0.472209837	0.377836	0.330068		0.251157919	0.321599	0.29287	
4	0.362444835	0.360357	0.362864		0.341510064	0.233258	0.20805	
8	0.335449307	0.381344	0.303373		0.180908655	0.184071	0.241484	

Figure 7S

Mock	siPARK7	siPARK7+NAC

BMPR1A	1.082037126	1.078293561	0.852881178	1.004921865	1.21314332	1.444346279	1.535544157	1.615614874	0.928999677	0.737347532	0.794847502	1.078293561
BMPR1B	1.127986655	1.158359714	0.820030798	0.933302501	1.436027456	1.717626871	1.649562377	1.993656233	1.1978246	1.120195082	0.921517414	1.227239367

Figure 7U

	NC						si*PARK7*					
BMPR1A	494.5594004	576.0299442	80.44885597	61.39290364	218.2745323	78.24897777	5.617779503	44.63179732	40.50421101	57.68002961	49.52207979	24.42014734
BMPR1B	354.5880238	210.8393004	119.4282229	335.4607114	168.8970126	135.2983092	16.56423878	23.42537114	36.00187151	55.71523605	86.82267696	64

Figure S6B

	NC				OE-NFE2L2			
BMP2	0.825019429	0.99826863	1.096191612	1.1076484	3.057812615	2.544707789	2.643592852	2.853040052
BMP4	1.04336938	0.85042151	1.126033696	1.000866809	1.654333386	1.306991719	1.297963667	1.445180807
BMPR1A	0.738413073	0.83364208	1.077359696	1.507857403	1.982746175	1.022782939	0.75917269	1.1076484
BMPR1B	0.676541341	1.52229715	1.165743189	0.832920092	0.892701651	0.758515202	0.880411564	0.620391145

Figure S6C

	NC				OE-NFE2L2			
FADS1	1.157356506	1.06744865	0.722381741	1.120518654	1.02870786	1.146709536	1.031087428	1.187148161
FADS2	1.293099539	0.86904332	0.903857138	0.984525173	0.851158668	1.257739664	1.047899238	1.266487935

Figure S6E

	DMSO				CMLD-2			
BMPR1A	1.393938263	1.14538557	0.825019429	0.75917269	0.411557715	0.385775475	0.433018329	0.454546565
BMPR1B	1.660556334	1.32408891	0.873572896	0.520631076	0.129857388	0.119769572	0.130760617	0.135059466

Figure 8A

Control	SCAP	ARDS
1.0783904	6.838812	8.9186368
1.2200816	5.46202	7.1778592
0.828744	4.24362	4.5936816
2.616752	3.128784	5.9836048
1.9555264	5.992024	5.7474528
1.0244128	4.895464	2.009504
2.920376	5.596044	5.8958912
0.5723504	3.701432	3.73004
1.946936	3.08614	2.8124208
2.677976	4.54822	7.3465392
3.061772	3.427292	6.76628
1.77636	3.433384	9.2829856
3.975572	2.982576	5.720464
1.843372	3.689248	2.3401168
	3.006944	4.8028448
	7.819624	4.5801872
	3.896376	2.8461568
	3.902468	5.7744416
	7.551576	1.9217904
	4.054768	5.1064688
		6.0038464
		2.0229984
		2.1849312
		3.8177536
		8.7432096
		7.5152192
		9.3572048
		2.4075888
		7.8525792
		9.3572048
		8.7517
		5.833632
		10.993556
		8.794344
		9.434004
		8.69078
		8.404456
		5.61432
		3.427292
		7.636864
		5.163512
		7.338356
		7.56376
		3.96948
		2.93384
		7.021572
		5.547308

Figure 8G

PBS	LPS	2-DG+LPS
0.929161	1.395388	0.979873
1.007421	1.702121	0.968618
0.887201	1.181539	1.028589
1.069794	1.154552	1.002776
1.125578	1.41814	1.012087

Figure 8H

PARK7

PBS	LPS	2-DG+LPS
1.090989	1.430798	0.61231
1.030508	1.192959	0.973645
0.90874	1.303734	0.944035
1.108387	1.39083	0.673007
0.861376	1.528554	0.930394

H3K14la

PBS	LPS	2-DG+LPS
0.93482	3.275333	0.321562
0.7205	2.427278	1.307382
1.297444	1.991795	1.108638
1.047236	2.305992	1.199248

Figure 8I

Mock	PS+Nigerici	G+LPS+Nige	A+LPS+Nigericin
0.898478	3.172358	2.186902	1.952815
1.068477	2.258798	1.822041	1.33999
1.041663	2.872331	1.541024	1.557129

Figure 8J

PARK7

Mock	PS+Nigerici	G+LPS+Nige	A+LPS+Nigericin
0.922561	1.636554	1.085593	0.827497
0.966432	1.392797	1.082038	1.074144
0.962963	1.669999	1.077974	0.863195

H3K14la

Mock	PS+Nigerici	G+LPS+Nige	A+LPS+Nigericin
0.972102	1.894778	1.105506	0.689993
1.315668	1.538301	0.92392	0.822708
1.019845	1.582338	0.973948	0.913789
1.308293	1.690747	0.837611	0.568844

Figure 8M

DMSO	Erastin
1.016305	1.450617
1.016305	1.180993
1.057018	1.286395
0.915945	1.613284

Figure 8N

PARK7

DMSO	Erastin
1.081971	1.254674
0.948042	2.181703
0.969987	2.026764

H3K14la

DMSO	Erastin
0.977599	2.834693
1.494286	2.85246

0.528115	2.840942

Figure 8O

NC	siFADS1	siFADS2
1.048101	1.373425	1.52391
0.885426	1.618261	1.592299
1.077567	1.52391	1.607083
1.010062	1.249292	2.110782

Figure 8P

PARK7

NC	siFADS1	siFADS2
0.83129	1.864941	2.11201
1.114809	1.477905	2.137304
1.033951	1.901451	1.629153
1.01995	1.773022	2.059031

H3K14la

NC	siFADS1	siFADS2
0.937552	2.275709	1.773367
0.972119	1.974003	1.867509
1.141081	1.98992	1.674737
0.949248	2.119191	1.826927

Figure 8Q

NC	si***FADS1***	si***FADS2***
1.180505	4.562206	3.492812
0.404203	5.245212	2.998776
1.415292	4.144917	3.485764

Figure 9F

	IgG				Anti-H3K14la			
-1000~TSS	1.460663888	2.123776156	0.147290579	0.268269377	2.773372478	4.696680561	3.841409204	2.534406115
TSS~+100(	1.473714104	0.721692753	0.852313845	0.952279297	4.390717376	5.874461763	7.282615481	6.563460571

Figure 9G

	PBS					Sodium Lactate					2-DG				
WT	0.929050512	0.926447929	1.067257613	1.095486581	0.981757365	1.187568665	1.0604825	1.370604468	1.220206485	1.173342276	0.742265768	0.760746646	0.702333943	0.850059755	0.756360281
MUT1	0.711070012	0.731466702	0.645660232	0.784432108	0.654275604	0.962486285	0.960491275	1.036911666	0.908330044	0.930920146	0.498681753	0.589211518	0.602872432	0.611688255	0.652390402
MUT2	0.780993708	0.611297907	0.603626297	0.729514924	0.606939799	0.872779339	0.919526708	0.871405355	0.866840436	0.951659335	0.46103942	0.551413729	0.578719796	0.625314828	0.606070748
MUT3	0.440767915	0.490926649	0.570275501	0.539240867	0.49389915	0.513390315	0.586916715	0.574202542	0.591130674	0.571946782	0.41218347	0.4031727	0.474115008	0.478643954	0.466056376

Figure 9H

H3f3a	H3f3b
679.1762	586.1499
722.0155	570.0249
710.754	549.0187

Figure 9L

	PBS					Sodium Lactate					2-DG				
WT	0.947970115	1.154349839	0.85630309	1.236173897	0.80520306	1.400799934	1.708542044	1.484071889	1.420035515	1.693430974	0.600846068	0.608045325	0.909542996	0.430203703	0.628675055
H3.3A-K14I	0.52772878	0.71973837	0.661817538	0.959498986	0.654086355	0.916207319	1.001566748	0.90184308	0.879696532	0.646006333	0.657923345	0.501640405	0.638680861	0.67895096	0.64816477

Figure 9M

	Vehicle			LPS+Nigericin		
WT	1.109569472	0.841868418	1.070536016	1.36761928	1.641483218	1.482809572
H3.3A-K14I	0.602207814	0.629233186	0.543995517	0.588453369	0.654440226	0.490276211

Figure 9N

	Vehicle				LPS+Nigericin			
WT	0.922433844	0.977328603	1.168577186	0.931660366	1.351271803	1.50282319	1.590492264	1.440991342
H3.3A-K14I	0.621141625	0.630364517	0.781783921	0.588194757	0.881373489	0.688762273	0.593674914	0.899179658
